# Structure-Based Design and Optimization of FPPQ, a Dual-Acting 5-HT_3_ and 5-HT_6_ Receptor Antagonist with Antipsychotic and Procognitive Properties

**DOI:** 10.1021/acs.jmedchem.1c00224

**Published:** 2021-09-01

**Authors:** Paweł Zajdel, Katarzyna Grychowska, Szczepan Mogilski, Rafał Kurczab, Grzegorz Satała, Ryszard Bugno, Tomasz Kos, Joanna Gołębiowska, Natalia Malikowska-Racia, Agnieszka Nikiforuk, Séverine Chaumont-Dubel, Xavier Bantreil, Maciej Pawłowski, Jean Martinez, Gilles Subra, Frédéric Lamaty, Philippe Marin, Andrzej J. Bojarski, Piotr Popik

**Affiliations:** † Faculty of Pharmacy, Jagiellonian University Medical College, 9 Medyczna Str., 30-688 Kraków, Poland; ‡ Maj Institute of Pharmacology, Polish Academy of Sciences, 12 Smętna Str., 31-343 Kraków, Poland; § Institut de Génomique Fonctionelle, Université de Montpellier, CNRS, INSERM, 34094 Montpellier, France; ∥ IBMM, Université de Montpellier, CNRS, ENSCM, 34095 Montpellier, France

## Abstract

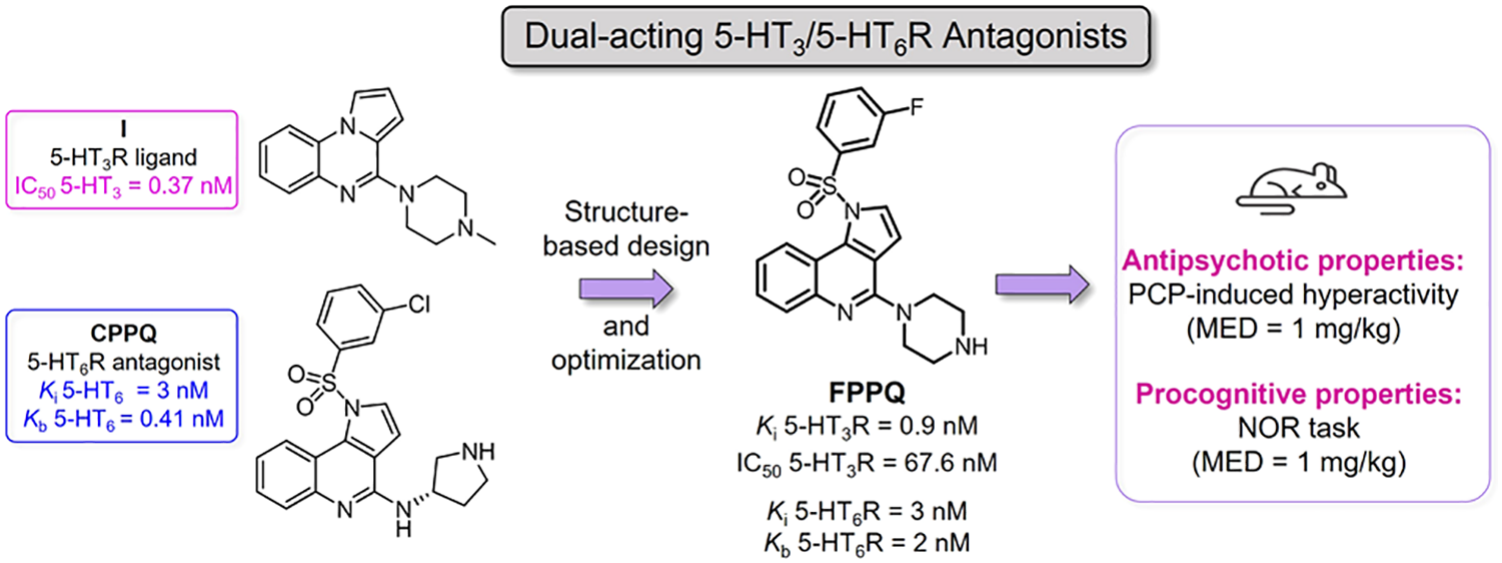

In line with recent clinical trials demonstrating that ondansetron, a 5-HT_3_ receptor (5-HT_3_R) antagonist, ameliorates cognitive deficits of schizophrenia and the known procognitive effects of 5-HT_6_ receptor (5-HT_6_R) antagonists, we applied the hybridization strategy to design dual-acting 5-HT_3_/5-HT_6_R antagonists. We identified the first-in-class compound **FPPQ**, which behaves as a 5-HT_3_R antagonist and a neutral antagonist 5-HT_6_R of the Gs pathway. **FPPQ** shows selectivity over 87 targets and decent brain penetration. Likewise, **FPPQ** inhibits phencyclidine (PCP)-induced hyperactivity and displays procognitive properties in the novel object recognition task. In contrast to **FPPQ**, neither 5-HT_6_R inverse agonist SB399885 nor neutral 5-HT_6_R antagonist CPPQ reversed (PCP)-induced hyperactivity. Thus, combination of 5-HT_3_R antagonism and 5-HT_6_R antagonism, exemplified by **FPPQ**, contributes to alleviating the positive-like symptoms. Present findings reveal critical structural features useful in a rational polypharmacological approach to target 5-HT_3_/5-HT_6_ receptors and encourage further studies on dual-acting 5-HT_3_/5-HT_6_R antagonists for the treatment of psychiatric disorders.

## Introduction

Schizophrenia is a debilitating mental disorder characterized by the presence of positive (hallucinations, delusions) and negative (social withdrawal, flat affect, low motivation) symptoms that are usually accompanied by cognitive impairment (e.g., learning and attention deficits). Despite the steady stream of antipsychotic drugs acting at a variety of monoamine receptors, the clinical management of schizophrenia is far from optimal. A significant number of patients under antipsychotic treatment experience persistent symptoms and an impaired quality of life. Approximately 30% of patients diagnosed with schizophrenia do not respond or only partially respond to existing drugs,^1^ with inadequate control of the core positive symptoms and relative inefficacy in treating the negative and cognitive symptoms.

A detailed analysis of the receptor profile of clozapine, the only antipsychotic used in treatment-resistant schizophrenia,^2,3^ in addition to well-known blockade of serotonin type 2A (5-HT_2A_) receptor, revealed the antagonistic properties at the serotonin type 3 receptor (5-HT_3_R)^4^ and serotonin type 6 receptor (5-HT_6_R).^5^ Although concurrent blockade of the muscarinic, histamine, and dopamine receptors hampers procognitive properties of clozapine,^1,6^ its high affinity for both 5-HT_3_R and 5-HT_6_R has triggered academic and industrial research.

Among the 14 serotonin receptor subtypes,^7^ 5-HT_3_R is a unique ionotropic receptor that belongs to the pentameric ligand-gated ion channel (LGIC) superfamily. 5-HT_3_R is located in both the CNS and periphery (including the small intestine and colon). Presynaptic 5-HT_3_Rs regulate calcium influx into nerve terminals, thus modulating the release of neurotransmitters in different brain areas (hippocampus, putamen, caudate nucleus, amygdala), while postsynaptic receptors located on GABA interneurons are associated with fast excitatory sodium and potassium depolarization.^8−10^ Blockade of presynaptic 5-HT_3_R inhibits overactive mesolimbic dopamine activity and GABA release and increases acetylcholine neurotransmission in the hippocampus and cortex. At the same time, blockade of 5-HT_3_R located in GABAergic interneurons enhances glutamatergic transmission. Ondansetron and granisetron, which behave as 5-HT_3_R antagonists, failed to alleviate the positive symptoms of psychosis. Still, they reduced the negative symptoms and improved cognitive symptoms when administered as adjuvant therapy to antipsychotics.^11−13^ Finally, 5-HT_3_R antagonists reduce haloperidol- and 5-hydroxytryptophan-induced extrapyramidal side effects, i.e., catalepsy and tardive dyskinesia.^12,14,15^

5-HT_6_R is a Gs-coupled receptor (GPCR) that is almost exclusively expressed in the CNS and is abundant in brain regions involved in cognitive functions such as the prefrontal cortex, hippocampus, and striatum. It is located postsynaptically to serotonergic neurons and is primarily localized in the primary cilium, a sensory organelle that participates in neurodevelopmental processes.

Recent studies on the 5-HT_6_R interactome identified additional signaling pathways, including the Fyn tyrosine kinase,^16^ mechanistic target of rapamycin (mTOR, involved in synaptic plasticity and cognition),^17^ and cyclin-dependent kinase 5 (Cdk5) pathway,^18^ which is critical for neuron migration and neurite growth. 5-HT_6_R antagonists improve cognitive performance in a wide range of preclinical models of cognitive impairment.^19−21^ The beneficial effects of 5-HT_6_R antagonists on cognition have been attributed to the enhanced release of acetylcholine and glutamate in the frontal cortex and hippocampus.^22,23^ Finally, the selective 5-HT_6_R antagonists—idalopirdine and AVN-211—have advanced to phase II and phase IIa clinical trials, respectively, as add-on therapies against schizophrenia, but the results were not conclusive.^24,25^

**Figure 1:**
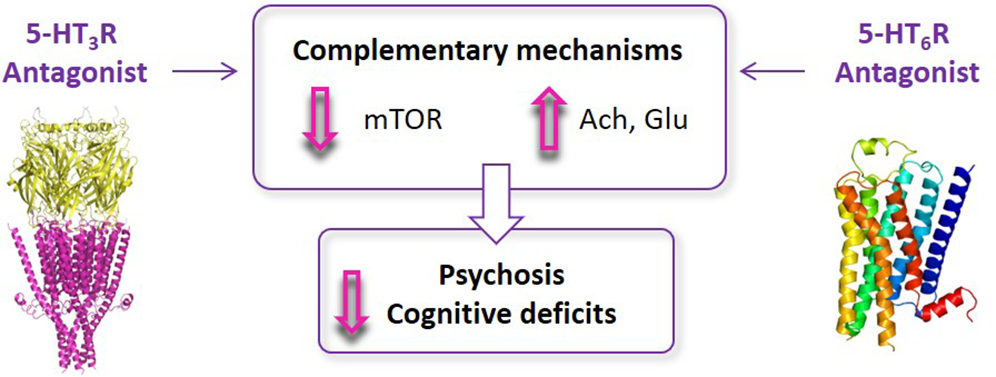
Schematic representation of the hypothetical influence of dual 5-HT_3_/5-HT_6_Rs antagonists on mTOR activity and neurotransmitters release.

**Figure 2:**
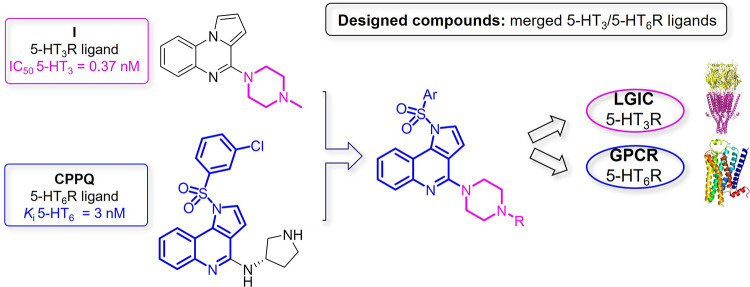
Strategy for the design of dual-acting 5-HT_3_/5-HT_6_Rs antagonists.

Given their role in different paradigms of cognitive impairment, 5-HT_3_R and 5-HT_6_R are promising targets for the development of dual-acting compounds with presumably more efficient therapeutic effects than selective agents (Figure 1).^26,27^ The molecular framework for developing compounds that target both receptors arises from the structural similarity of pyrroloquinoxaline **I**, a 5-HT_3_R ligand,^28^ and CPPQ, a pyrroloquinoline-based 5-HT_6_R antagonist (Figure 2).^29^ The rationale toward “selective unselective” compounds was achieved using a hybridization strategy, which involved merging the pharmacophores of **I** and CPPQ into a unique molecular entity.^30−32^

Based on a combination of rational design and *in silico* analysis, we evaluated the structure–activity relationships of dual-acting 5-HT_3_/5-HT_6_R antagonists. Structural modifications comprised diversification of the amine fragment at position 4 of the 1*H*-pyrrolo[3,2-*c*]quinoline core and functionalization of the *N*^1^ atom of the tricyclic scaffold with various arylsulfonyl moieties. We selected a lead compound 1-[(3-**f**luorophenyl)sulfonyl]-4-(**p**iperazin-1-yl)-1*H*-**p**yrrolo[3,2-*c*]**q**uinoline (**FPPQ**) with balanced target activity (leaving 87 targets unaffected) and favorable oral absorption and CNS penetration. Similar to the reference drug clozapine, **FPPQ** attenuated phencyclidine (PCP)-induced hyperlocomotion, and enhanced novelty discrimination of PCP-treated rats in the novel object recognition (NOR) test. These data might support the potential antipsychotic activity of **FPPQ** that relies on its dual 5-HT_3_/5-HT_6_R antagonism.

## Results and Discussion

### Synthesis

**Scheme 1:**
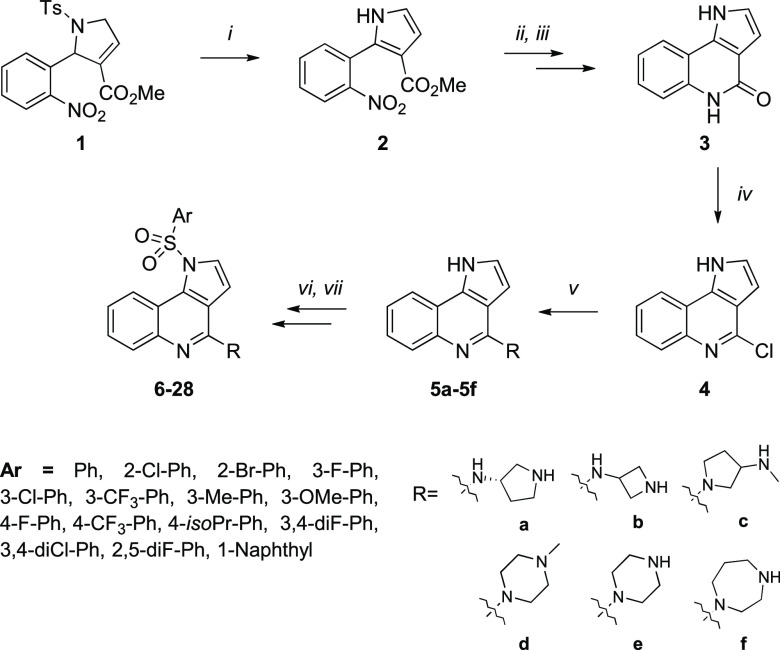
Synthetic Pathway Leading to Compounds **6**–**28**: (i) Na-OtBu, DMF, rt, 2 h; (ii) H_2_, Pd/C, MeOH, rt, 2 h; (iii) AcOH, *sec*-BuOH, 60°C, 3 h; (iv) POCl_3_, 105°C, 4 h; (v) primary amine, MeCN, MW 140°C, 7 h or secondary amine, TEA, toluene, 114 °C, o/n; (vi) arylsulfonyl chloride, BTPP, CH_2_Cl_2_, 0°C → rt, 3 h; (vii) 1M HCl/MeOH, rt, 5 h

Designed compounds **6**–**28** were synthesized in a multistep synthetic pathway starting from pyrroline **1** obtained according to our previously reported method (Scheme 1).^33,34^ Subsequent aromatization to pyrrole derivative **2**, followed with reduction of nitro group, then cyclization of arylpyrrole derivative to lactam **3**, and chlorination of the latter afforded 1*H*-pyrrolo[3,2-*c*]quinoline **4**. Stirring of key synthon **4** with the respective primary amines required prolonged heating in acetonitrile under microwave-assisted conditions to yield amino derivatives **5a** and **5b**. On the other hand, the reaction with secondary amines proceeded smoothly in the presence of triethylamine (TEA) in refluxing toluene to furnish amino derivatives **5c**–**5f**. Subsequent coupling with selected sulfonyl chlorides in the presence of phosphazene base P1-*t*-Bu-tris(tetramethylene) (BTPP) provided sulfonamide derivatives **6**–**28**.^35^ The Boc-protected products were finally converted into the HCl salts of secondary amines upon treatment with 1 M HCl solution in methanol.

### Structure–Activity Relationship Studies

**Figure 3:**
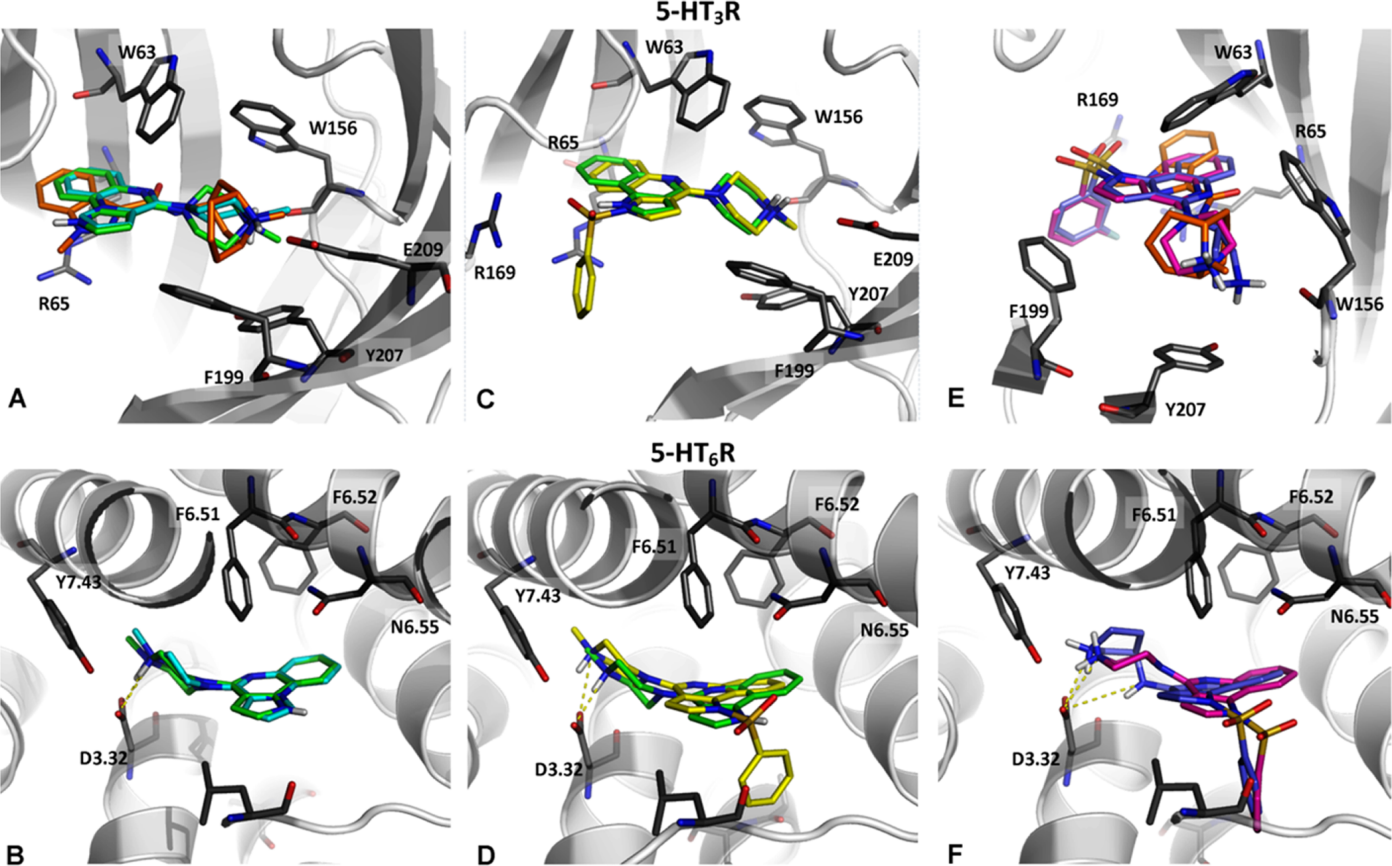
Illustration of binding modes of selected compounds in the orthosteric binding site of 5-HT_3_R (PDB ID: 6NP0) and 5-HT_6_R (a homology model built on a β2 adrenergic template; PDB ID: 4LDE). Comparison of binding modes of compound **I** (cyan), **5d** (green) vs granisetron (orange) in 5-HT_3_R (A), and **I** vs **5d** in 5-HT_6_R (B). (C, D) Binding modes of **5d** (green) and **6** (yellow) in 5-HT_3_R and 5-HT_6_R, respectively. (E, F) Illustration of the binding modes for analogues with five-membered (**8**; violet) and six-membered (**17**; magenta) aliphatic ring containing nitrogen atom in 5-HT_3_ and 5-HT_6_Rs, respectively.

To initiate the quest for dual-acting 5-HT_3_/5-HT_6_R antagonists, the approach entailed identification of the common structural features of known 5-HT_3_R and 5-HT_6_R ligands (Figure 2). Molecular docking analysis indicated that pyrroloquinoxaline **I**, a 5-HT_3_R ligand, shows coherent binding mode with that of granisetron – a 5-HT_3_R antagonist (Figure 3A).^36^ Further analysis of pyrroloquinoxaline **I**, suggested that pyrroloquinoline **5d**, with the bridgehead nitrogen shifted to position 1 of the pyrrole ring, would occupy the same binding site in 5-HT_3_R. The pyrroloquinoline moiety is constrained by the CH−π interaction with W63, and cation−π interaction with R65 in the 5-HT_3_R binding site, whereas the positively charged methyl piperazine moiety is located in the pocket formed by W156, Y207, F199, W63, and E209. Mutual spatial relationships enable the creation of cation−π interactions, trapping the charged methyl piperazine fragment between the side chains of W156 on one side and F199/Y207 on the other side (Figure 3A).

As revealed by the functional ex vivo assays, which measured the effects of the compounds on guinea pig ileum contractions induced by serotonin (5-HT), pyrroloquinoline **5d** similarly to 5-HT induced contraction of ileum and was classified as an agonist at 5-HT_3_R (100% response of serotonin used as control agonist at 100 nM) (Table 1). Its demethylated analogue **5e** behaved as a partial agonist in this assay (43% response at 300 nM).

**Table 1:**
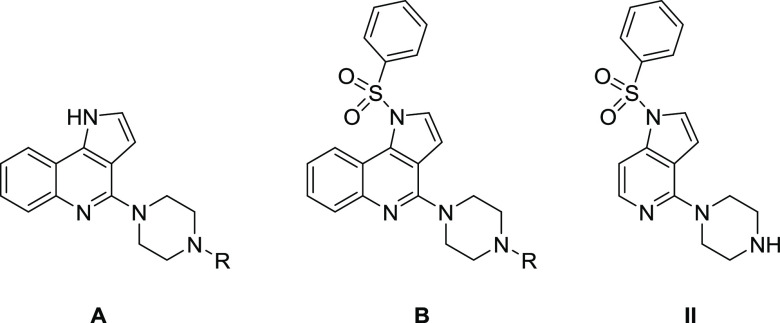
Agonist/Antagonist Properties of Compounds **5d**, **5e**, **6**, **7**, **II**, and Ondansetron for 5-HT_3_Rs, and Antagonist Properties and Binding Data of Compounds **5d**, **5e**, **6**, **7**, **II**, Ondansetron, Intepirdine, and SB399885 for 5-HT_6_Rs

			5-HT_3_R			5-HT_6_R	
compound	core	R	agonist effect^b^	antagonist effect^c^	pD_2_′^d^	*K*_b_ [nM]^e^	*K*_i_ [nM]^f^
5d	A	CH_3_	100 (100 nM)	NT	NT	>10 000	245
5e	A	H	9 (100 nM) 43 (300 nM)	NT	NT	>10 000	757
6	B	CH_3_	NT	28 (100 nM) 48 (300 nM)	NT	4	2
7	B	H	NT	26 (100 nM) 60 (300 nM)	6.43	17	11
II^a^			NT	7 (300 nM)	NT	1	6
ondansetron			NT	pA_2_ = 7.11 ± 0.12	NT	>10 000	NT
intepirdine			NT	NT	NT	1.2	1.4
SB399885			NT	NT	NT	1.6	0.7

a Compound reported in ref (37). For synthesis, see Supporting Information Scheme S1.

b The effect induced by the tested compounds at the concentration of 100 or 300 nM expressed as a percent of maximal contraction of guinea pig ileum induced by control agonist (5-HT).

c Percent inhibition of response to stimulation by 5-HT (contraction of guinea pig ileum) at the concentration of 3 μM induced by different concentrations of tested compounds shown in brackets (*N* = 6–8, SEM ≤ 12%).

d Antagonist potency expressed as pD_2_′ (*N* = 6–8, SEM ≤ 14%).

e Mean *K*_b_ values based on two independent experiments in 1321N1 cells (SEM ≤ 22%).

f Mean *K*_i_ values based on three independent binding experiments in HEK cells stably expressing *h*5-HT_6_R (SEM ≤ 15%).

Closer inspection of the binding mode of **5d** in 5-HT_3_R showed that the binding pocket filled with the tricyclic scaffold leaves some space for structural modifications. Extension of the pyrroloquinoline core at the *N*^1^ atom with a phenylsulfonyl fragment enabled a distinct cation−π interaction with R169, which stabilized the ligand–receptor (L–R) complex (Figure 3C). Of note, compounds **6** and **7**, bearing phenylsulfonyl fragment, did not exert any contractile effect on the guinea pig ileum, but efficiently inhibited serotonin-induced contraction of the tissue. Thus, the introduction of a phenylsulfonyl fragment switched the initial agonist activity at 5-HT_3_R (**5d**, **5e**) into antagonistic properties (**6**, **7**), leading the functional properties in the desired direction (Table 1).

Introducing a hydrophobic fragment, linked via a double electron–acceptor sulfonyl group to the pyrroloquinoline core, was also advantageous for interaction with 5-HT_6_R. This modification allowed us to construct the framework required for 5-HT_6_R antagonism, as revealed by the inhibitory activity of arylsulfonyl derivatives **6** and **7** in the cAMP assay performed in 1321N1 cells (Table 1). A similar trend was observed for the 5-HT_6_R binding data (**6***K*_i_ = 2 nM vs **5d***K*_i_ = 245 nM; **7***K*_i_ = 11 nM vs **5e***K*_i_ = 757 nM, Table 1). The docking analysis results showed that the introduction of phenylsulfonyl fragment (**6**) did not significantly change the binding mode compared with the reference analogue **5d** (Figure 3B,D). Nevertheless, the phenylsulfonyl fragment interacts with the hydrophobic pocket formed by helixes 3–5 in 5-HT_6_R and additionally stabilizes L–R complex.

**Figure 4:**
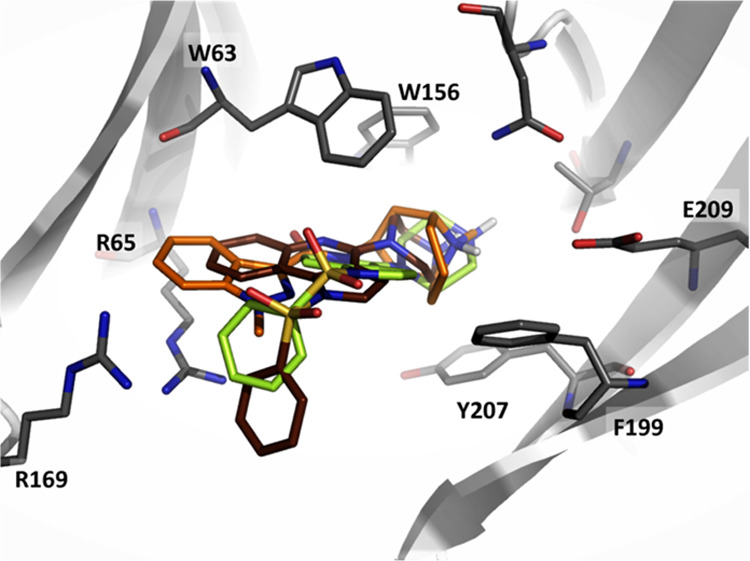
Binding modes of compounds **7** (brown), **II** (lemon), and granisetron (orange) in the active site of 5-HT_3_ (PDB ID: 6NP0).

Next, we focused on the planar pyrroloquinoline skeleton’s role in the interactions with 5-HT_3_R and 5-HT_6_R. Consistent with the binding model, the fused benzene ring in the pyrroloquinoline core forms an additional cation−π interaction with R65 (Figure 4). Its deletion, which resulted in the degradation of the pyrroloquinoline to the azaindole core, was detrimental for targeting the 5-HT_3_ site (**II** vs **7**). This observation is in line with data reported for pyrroloquinoxaline and imidazoquinoxaline series, where removal of the fused benzene ring led to a loss of antagonistic activity at 5-HT_3_R.^38^

Further considerations, employing combined medicinal chemistry and docking approaches, functionalized the *C*^4^ position of the pyrroloquinoline core with various alicyclic amines (Table 2, Supporting Information Table S1). Based on the geometry of the interactions between the protonated basic group and R65/Y207 for 5-HT_3_R (cation−π) (Figure 3E), and D3.32 (salt bridge) for 5-HT_6_R (Figure 3F), the designed structures were scored and subsequently selected for synthesis (Table 2).

**Table 2:**
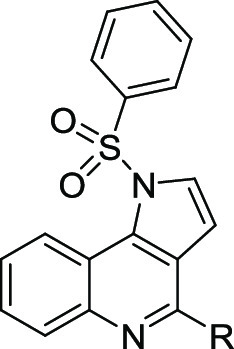

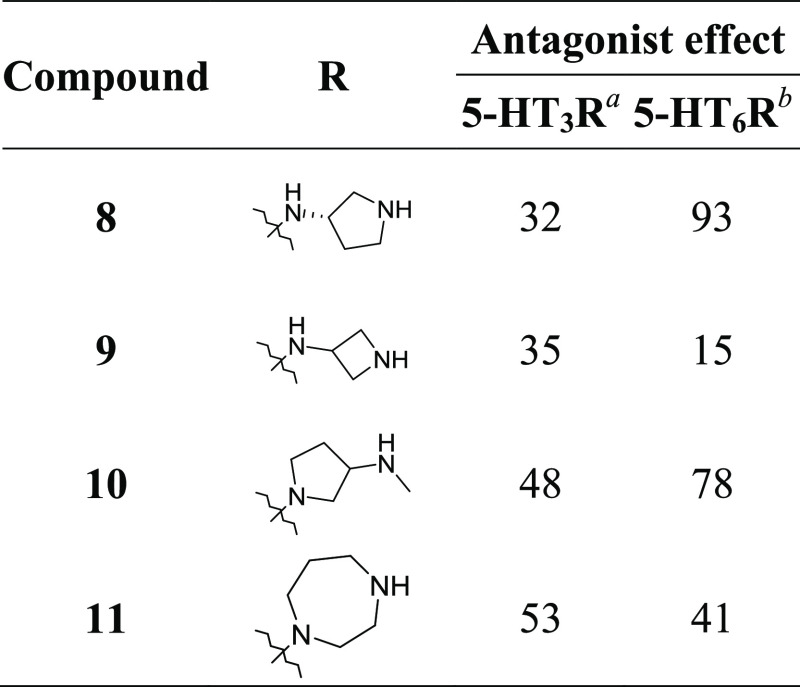
Antagonist Properties of Compounds **8**–**11** at 5-HT_3_ and 5-HT_6_ Receptors^a^^,^^b^

a Percent inhibition of response to stimulation by 5-HT (contraction of guinea pig ileum) at the concentration of 3 μM induced by tested compounds (300 nM).

b Percent inhibition of control agonist response at 10^–6^ M; performed in duplicate in 1321N1 cells.

Replacement of the 3-aminopyrrolidine fragment present in CPPQ with 3-aminoazetidine, connected to a pyrroloquinoline moiety by the exocyclic nitrogen atom, did not significantly influence the antagonist properties for 5-HT_3_R (**9** vs **8**). In contrast, introduction of secondary amines, connected to the pyrroloquinoline core by endocyclic nitrogen, was beneficial in terms of antagonist potency for this target (**6**, **7**, **10**, **11** vs **8**) (Tables 1 and 2).

In turn, the antagonist properties at 5-HT_6_R were strongly affected by the alicyclic ring’s size, since four- and seven-membered rings reduced antagonist activity at this site. These observations indicate that only the six-membered piperazine ring in the *C*^4^ position of the pyrroloquinoline core ensures the desired pharmacological profile at both targets (Table 2, Figure 3E,F).

We next explored the optimal substituents in the arylsulfonyl part (Table 3). Among the methyl piperazine derivatives (**12–14**), no substantial difference in antagonist potency at 5-HT_6_R was observed between compounds bearing halogen atoms in position 3 and their unsubstituted congener (**12**, **13** vs **6**). On the other hand, an introduction of a fluorine atom in position 3 of the arylsulfonyl moiety was highly favorable in terms of antagonist properties at 5-HT_3_R (**12**, 74% at 100 nM vs **6**, 28% at 100 nM).

**Table 3:**
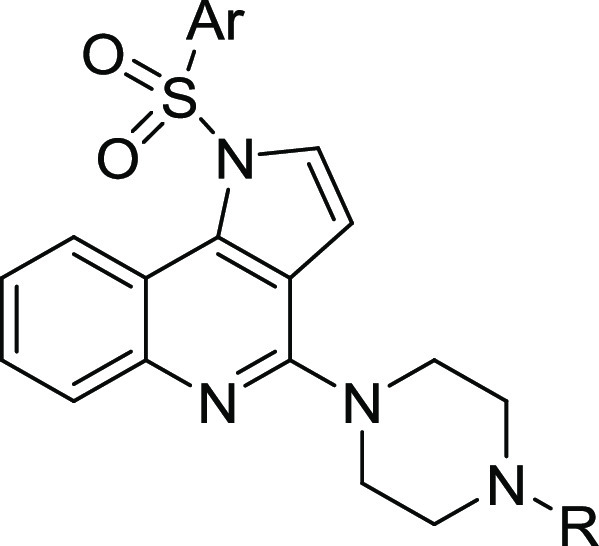
Antagonist Properties and Binding Data of Compounds **12**–**28**, Ondansetron, and Intepirdine at 5-HT_3_ and 5-HT_6_ Receptors Suggest That Dual-Acting 5-HT_3_/5-HT_6_R Antagonists (**17**, **18**, **20**) Display the Most Favorable Profile

			5-HT_3_R		5-HT_6_R		
compound	Ar	R	antagonist effect^a^	pD_2_′^b^	antagonist effect^c^	*K*_b_ [nM]^d^	*K*_i_ [nM]^e^
12	3-F-Ph	CH_3_	30 (30 nM) 74 (100 nM)	7.33	83	6	2
13	3-Cl-Ph	CH_3_	10 (100 nM) 44 (300 nM)	NT	90	5	2
14	4-F-Ph	CH_3_	NT	NT	75	7	10
15	2-Br-Ph	H	NT	NT	69	NT	13
16	2-Cl-Ph	H	NT	NT	75	50	5
17 FPPQ	3-F-Ph	H	32 (100 nM) 78 (300 nM)	7.43	92	2	3
18	3-Cl-Ph	H	11 (100 nM) 72	6.71	100	32	3
19	3-CF_3_-Ph	H	21 (100 nM) 37 (300 nM)	6.09	82	17	3
20	3-Me-Ph	H	20 (30 nM) 40 (100 nM)	6.74	89	38	3
21	3-OMe-Ph	H	28 (100 nM) 73 (300 nM)	6.38	82	32	7
22	4-F-Ph	H	48 (300 nM)	NT	72	NT	18
23	4-CF_3_-Ph	H	NT	NT	63	NT	34
24	4-*i*Pr-Ph	H	38 (300 nM)	NT	65	NT	14
25	3,4-diF-Ph	H	NT	NT	56	124	18
26	3,4-diCl-Ph	H	NT	NT	70	58	12
27	2,5-diF-Ph	H	32 (300 nM)	NT	88	9	4
28	1-naphthyl	H	NT	NT	95	18	14
ondansetron			NT	pA_2_ = 7.11	1	58 220	NT
intepirdine			NT	NT	NT	1.2	1.4

a Percent inhibition of response to stimulation by 5-HT (contraction of guinea pig ileum) at the concentration of 3 μM induced by different concentrations of test compounds shown in brackets (*N* = 6–8, SEM ≤ 12%).

b Antagonist potency expressed as pD_2_′ or pA_2_(*N* = 6–8, SEM ≤ 0.19).

c Percent inhibition of control agonist (5-HT) response at 10^–6^ M; performed in duplicate in 1321N1 cells.

d Mean *K*_b_ values based on two independent experiments in 1321N1 cells (SEM ≤ 22%).

e Mean *K*_i_ values based on three independent binding experiments (SEM ≤ 15%).

Because metabolic stability experiments using rat liver microsomes revealed higher susceptibility of *N*-methylated derivatives to metabolic enzymes (**12**, Cl_int_ = 32.65 μL/min/mg vs **17**, Cl_int_ = 12.8 μL/min/mg; **6**, Cl_int_ = 38.57 μL/min/mg vs **7**, Cl_int_ = 4.48 μL/min/mg), only unsubstituted derivatives were submitted for further investigation.

Among the desmethyl analogues, the introduction of a fluorine atom in position 3 improved antagonist activity at 5-HT_3_R (**17**, pD_2_′ = 7.43) and increased the antagonist properties for 5-HT_6_R (*K*_b_ = 2 nM) up to 5-fold compared with the unsubstituted compound **7** (Tables 1 and 3). Regardless of the substituents’ electronic properties, the presence of chlorine atom, methyl or methoxy group did not significantly affect antagonist activity at either receptor (**18**, **20**, **21** vs **7**).

Shifting of the halogen atom from position 3 to 2 of the arylsulfonyl fragment slightly decreased antagonist properties at 5-HT_6_R (**18** vs **16**). Substitution at position 4 (**22**–**24**) afforded a drop in antagonist effects at both targets, regardless of the substituent’s volume and electronic properties.

3,4-Difluoro and 3,4-dichloro derivatives (**25**, **26** vs **18**) revealed an unfavorable effect of the 3,4-disubstitution pattern on the antagonist properties at the 5-HT_6_R. On the other hand, the introduction of fluorine atoms at positions 2 and 5 (**27**) maintained activity at 5-HT_6_R but decreased antagonist potency at the 5-HT_3_ sites. Subsequently, expansion of the aromatic ring system by introduction of a naphthyl moiety reduced antagonist potency at 5-HT_6_R (**28**).

In summary, in the desmethylpiperazine series, monosubstitution of the arylsulfonyl fragment with halogen atoms (**17**, **18**) or small electron-donating substituents (**20**) in position 3, was the most favorable modifications to ensure antagonist properties at both targets.

Based on its highest antagonistic potency at both 5-HT_3_R and 5-HT_6_R (pD_2_′ (5-HT_3_R) = 7.43, *K*_b_ (5-HT_6_R) = 2 nM, Table 3) and metabolic stability (Cl_int_ = 12.8 μL/min/mg), **FPPQ** was selected for a more detailed evaluation. Additionally, compounds **18** and **20** were chosen for in vitro evaluation for their selectivity over selected GPCRs (Table 4). These experiments confirmed a class-effect selectivity over 5-HT_1A_, 5-HT_2A_, and 5-HT_7_ receptors. Importantly, evaluated derivatives did not bind to dopaminergic D_2_Rs. Therefore, these compounds might be devoid of the side effects associated with D_2_R blockade, such as extrapyramidal symptoms and prolactin release.

**Table 4:**
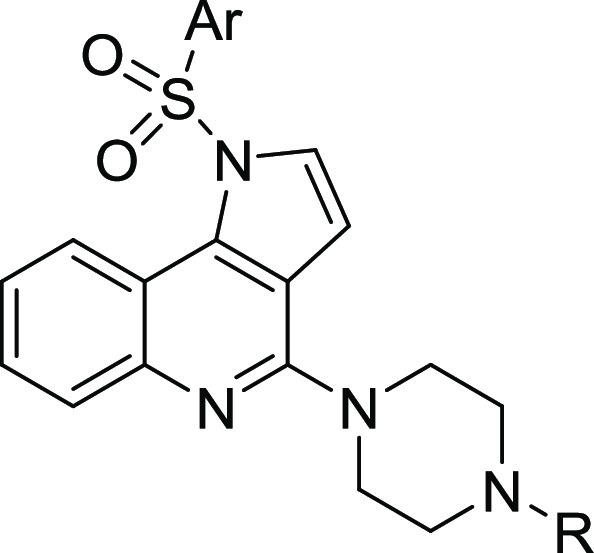
Binding Data of Compounds Selected from the Synthesized Library for 5-HT_6_, 5-HT_3_, 5-HT_1A_, 5-HT_2A_, 5-HT_7_, and D_2_Rs Suggest Marginal Affinity toward 5-HT_1A_, 5-HT_2A_, 5-HT_7_, and Dopamine D_2_ Receptors

			*K*_i_ [nM]^a^					
compound	Ar	R	5-HT_6_R	5-HT_3_R	5-HT_1A_R	5-HT_2A_R	5-HT_7_R	D_2_R
17 (FPPQ)	3-F-Ph	H	3	0.93^b^	437	3005	2997	4392
18	3-Cl-Ph	H	3	NT	773	1666	1794	1345
20	3-Me-Ph	H	3	NT	760	4631	4139	2156

a Mean *K*_i_ values (SEM ≤ 22%) based on at least three independent binding experiments.

b Performed at Eurofins.

In addition to ex vivo functional evaluation of the series of pyrroloquinolines at 5-HT_3_R in guinea pig ileum, **FPPQ** was profiled in the electrophysiological method using *h*5-HT_3_R ion channel cell-based antagonist IonFlux assay. In this cellular model, FPPQ inhibited inward currents in response to the 5-HT addition, and behaved as an antagonist (IC_50_ = 0.0676 μM). A similar effect at *h*5-HT_3_R was observed for palonosetron (IC_50_ = 0.0017 μM) used as a reference 5-HT_3_R antagonist.

To further assess the selectivity of **FPPQ**, SafetyScreen profiling was conducted at Eurofins (Table 5, Supporting Information Table S2). This experimental panel of 87 receptors, ion channels, transporters, and enzymes assesses interactions with proteins that are distinct from the intended molecular target and predicts potential clinical adverse effects. **FPPQ** displayed > 1–3 orders of magnitude higher affinity for 5-HT_3_R and 5-HT_6_R than for an array of receptors and enzymes expressed in the brain. Cardiac safety assessment of **FPPQ** was based on its lack of agonistic effect at 5-HT_2B_R (3.6% inhibition at 1 μM) which is indicative of valvulopathy and reasonably high selectivity (1000-fold) over *h*ERG channels (*K*_i_ = 0.94 μM) which are responsible for prolongation of the QT interval.

**Table 5:** Affinity of **FPPQ** for Receptors, Transporters, and Ion Channels Selected from Selectivity Profiling Panel, Compared with Its Affinities at 5-HT_3_R and 5-HT_6_R Main Targets, Suggest Decent Selectivity of **FPPQ** Compound

assay^a^	*K*_i_ [μM]
5-HT_3_	0.00093^b^
5-HT_6_	0.003^b^
α_2A_	0.11
5-HT_2B_	0.17
β_1_	0.17
D_3_	0.21
H_1_	0.22
Ca^+2^ channel L-type, dihydropyridine	0.50
Na^+^ channel, site 2	0.71
5-HT_2C_	0.83
5-HT_1B_	0.89
*h*ERG	0.94
Ca^+2^ channel L-type, benzothiazepine	1.08
DAT	1.09
NET	1.24
Ca^+2^ channel L-Type, phenylalkylamine	1.32
M_1_	1.94
5-HT_5A_	2.87
μ (OP3, MOP)	2.88
κ (OP2, KOP)	3.27
σ_1_	3.50
α_2B_	4.61

a Items meeting criteria of significance (≥50% stimulation or inhibition at 10 μM). For the results of all enzyme and radioligand binding assays, see Supporting Information Table S2.

b See Table 4.

The lack of off-target-related adverse effects observed with the currently available antipsychotics, such as sedation, hyperprolactinemia, obesity, and a propensity to induce tardive dyskinesia, might be an additional benefit of dual-acting 5-HT_3_R/5-HT_6_R antagonists.

**Figure 5:**
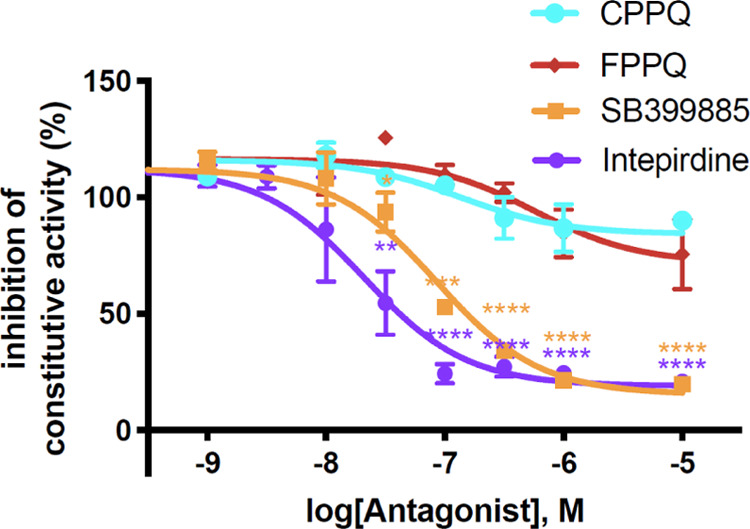
Influence of **FPPQ**, CPPQ, SB399885, and intepirdine on 5-HT_6_R constitutive activity at Gs signaling in NG108-15 cells. NG108-15 cells transiently expressing the 5-HT_6_R and the cAMP BRET sensor CAMYEL were exposed to increasing concentrations of SB399885, intepirdine, CPPQ, or **FPPQ** for 5 min. Cyclic AMP levels were estimated by measuring the CAMYEL BRET signal. Data are mean ± SEM of the values obtained in three independent experiments performed in quadruplicate using different sets of cultured cells. The BRET observed with the highest concentration of FPPQ is not significantly different from basal BRET (*p* = 0.1226, unpaired t test. **p < 0.01, ****p < 0.0001 vs vehicle (ANOVA followed by Dunnett’s multiple comparison test).

Since 5-HT_6_R displays a high level of constitutive activity, defined as the ability of the receptor to adopt an active conformation that enables signal transduction in the absence of an agonist, 5-HT_6_R ligands can be classified as inverse agonists or neutral antagonists.^39^ Evaluation of the impact of **FPPQ** on agonist-independent 5-HT_6_R-operated Gs signaling was performed in NG108-15 cells transiently expressing 5-HT_6_Rs, a cellular model in which 5-HT_6_R exhibits a high level of constitutive activity.^18,40^**FPPQ** did not significantly change the level of cAMP, which indicates its neutral antagonist properties toward this signaling pathway (Figure 5). Thus, FPPQ behaves similarly to CPPQ, a reference neutral antagonist of 5-HT_6_R.^29^ On the other hand, SB399885 and intepirdine, the reference 5-HT_6_R antagonists, strongly decreased basal cAMP level in a concentration-dependent manner and thus behaved as inverse agonists at Gs signaling (IC_50_ equals 97 nM and 2.8 nM for SB399885 and intepirdine, respectively).

**Figure 6:**
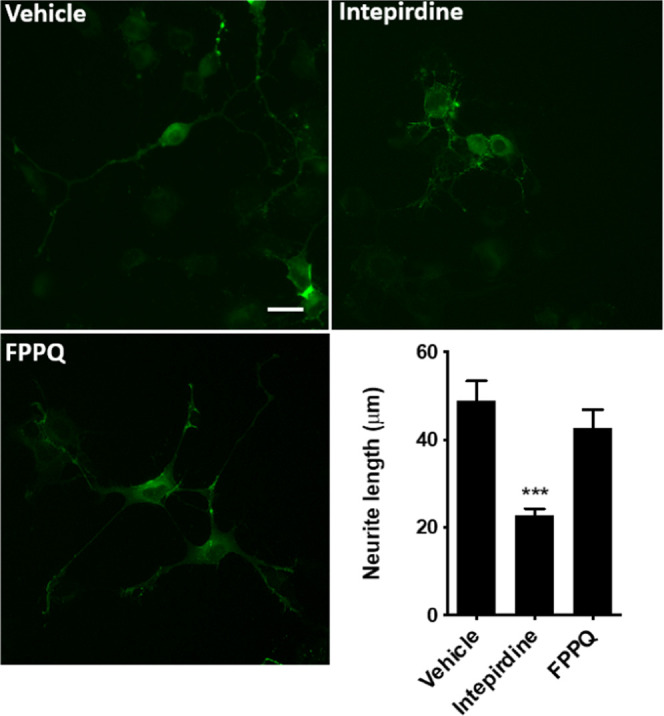
Effects of **FPPQ** (1 μM), intepirdine (1 μM), and DMSO (Vehicle) on neurite length in NG108-15 cells transfected with a plasmid encoding a GFP-tagged 5-HT_6_R. The histogram shows the mean + SEM of neurite length in each condition measured from three independent experiments. Symbols: ***p < 0.001 vs cells expressing 5-HT_6_R and treated with DMSO (Vehicle). Scale bar, 10 μm.

Moreover, **FPPQ** did not prevent neurite growth elicited by 5-HT_6_R expression in NG108-15 neuroblastoma cells, a process that is mediated by agonist-independent activation of Cdk5 signaling. In contrast, intepirdine reduced NG108-15 cell neurite length and thus behaved as an inverse agonist at Cdk5 signaling elicited by constitutively active 5-HT_6_R (Figure 6).

Both neutral antagonists and inverse agonists of 5-HT_6_R have been in clinical trial for alleviating cognitive symptoms of schizophrenia. Post-mortem analysis of brains of patients with schizophrenia showed decreased levels of Cdk5 and its activator p35, suggesting a reduced Cdk5 activity in the disease.^41^ Accordingly, developing neutral 5-HT_6_R antagonists such as **FPPQ** that do not inhibit Cdk5 signaling might be of greater interest than inverse agonists due to presumably less pronounced side effects linked to reduction of agonist-independent, 5-HT_6_R-operated Cdk5 signaling.

### Preliminary Assessment ADME Properties as well as Safety of FPPQ

With a molecular weight of 410.47 Da, a clogP of 4.20, a PSA of 67.23 Å^2^, one hydrogen bond donor, three H-bond acceptors, and two rotatable bonds, the calculated descriptors confirmed the CNS druglike properties of compound **FPPQ**.^42,43^ Its physicochemical properties include a basic p*K*_a_ of 8.78, indicating that this compound would be partially protonated at physiological pH. The aqueous solubility of **FPPQ** is high (1.4. mmol/mL at pH 7.0). **FPPQ** is chemically stable at both pH 1.2 and 8.0, which reflects the pH range along the gastrointestinal tract. FPPQ shows metabolic stability in both rat liver microsomes (12.8 μL/min/mg) and in human microsomes (8.2 μL/min/mg, Table 6).

**Table 6:** **FPPQ** Displays Decent Metabolic Stability and Weakly Interacts with Cytochrome P450 Isozymes

assay type		FPPQ
solubility [mmol/ml]		1.4
microsomal stability [CL_int_ μL/min/mg]	rat	12.8
	human	8.2
cytochrome P450 inhibition	1A2	12 μM
	2C19	10 μM
	3A4	57 μM
	2C9	<25% inh at 10 μM
	2D6	<25% inh at 10 μM

To examine the propensity of potential drug–drug interactions, the inhibitory activity of **FPPQ** against cytochrome P450 (CYP) isoenzymes predominantly engaged in drug biotransformation was tested. **FPPQ** did not inhibit CYP2C9 and CYP2D6 (below 25% inhibition at 10 μM), and had moderate inhibitory activity against CYP1A2, CYP2C19, and CYP3A4 (Table 5). Moreover, **FPPQ** showed no mutagenic potential in the Salmonella mutagenic test, which further confirmed its safety profile (Supporting Information Table S3).

### Preliminary In Vivo Pharmacokinetics of FPPQ

**Figure 7:**
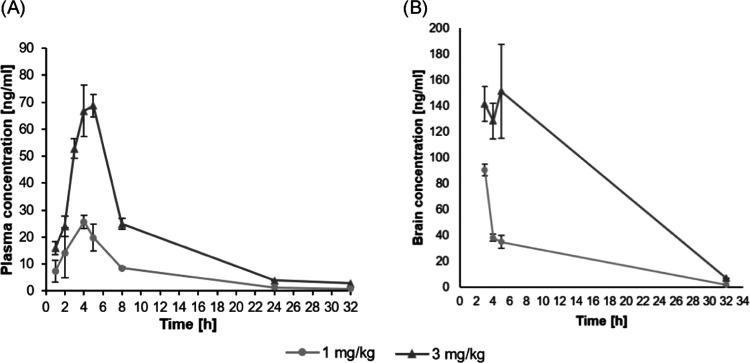
Pharmacokinetic study displays sufficient plasma and brain concentration following oral administration in rats. Data show the concentration (ng/mL) of **FPPQ** in the plasma (A) and brain (B) of rats at different time points (in hours, h) after the administration of the drug at two doses and are expressed as mean ± SEM (*N* = 3 per each time point).

To investigate the pharmacokinetics of **FPPQ** in vivo, we determined its plasma and brain concentrations at various time points after oral administration (1, and 3 mg/kg) in male Lister hooded rats. **FPPQ** reached its maximal concentration (C_max_ = 0.22 μM, and C_max_ = 0.37 μM, for doses 1 and 3 mg/kg, respectively) in plasma and brain between 3 and 5 h after drug administration, regardless of the dose used, suggesting that at these doses, the compound could easily affect its main (5-HT_3_R and 5-HT_6_R) brain targets. **FPPQ** showed good brain penetration, with the concentration varying proportionally to the injected dose. The brain/plasma ratio was 2.1 ± 0.1 considering all analyzed samples (mean ± SEM, *n* = 36). At both doses, the levels of **FPPQ** in the plasma and brain decreased between 5 and 32 h post-injection, indicating that accumulation in the brain upon repeated dosing at similar dose levels is unlikely (Figure 7).

### Antipsychotic and Procognitive Properties

Psychotomimetic compounds such as uncompetitive *N*-methyl-D-aspartate (NMDA) receptor antagonists (i.e., ketamine and PCP) induce schizophrenia-like symptoms in healthy volunteers and their administration to rodents serves as a model of psychosis.^44^ First- and second-generation antipsychotic medications with dopamine D_2_ and 5-HT_2A_R antagonism prevent PCP-induced increase in locomotor activity.^45^

**Figure 8:**
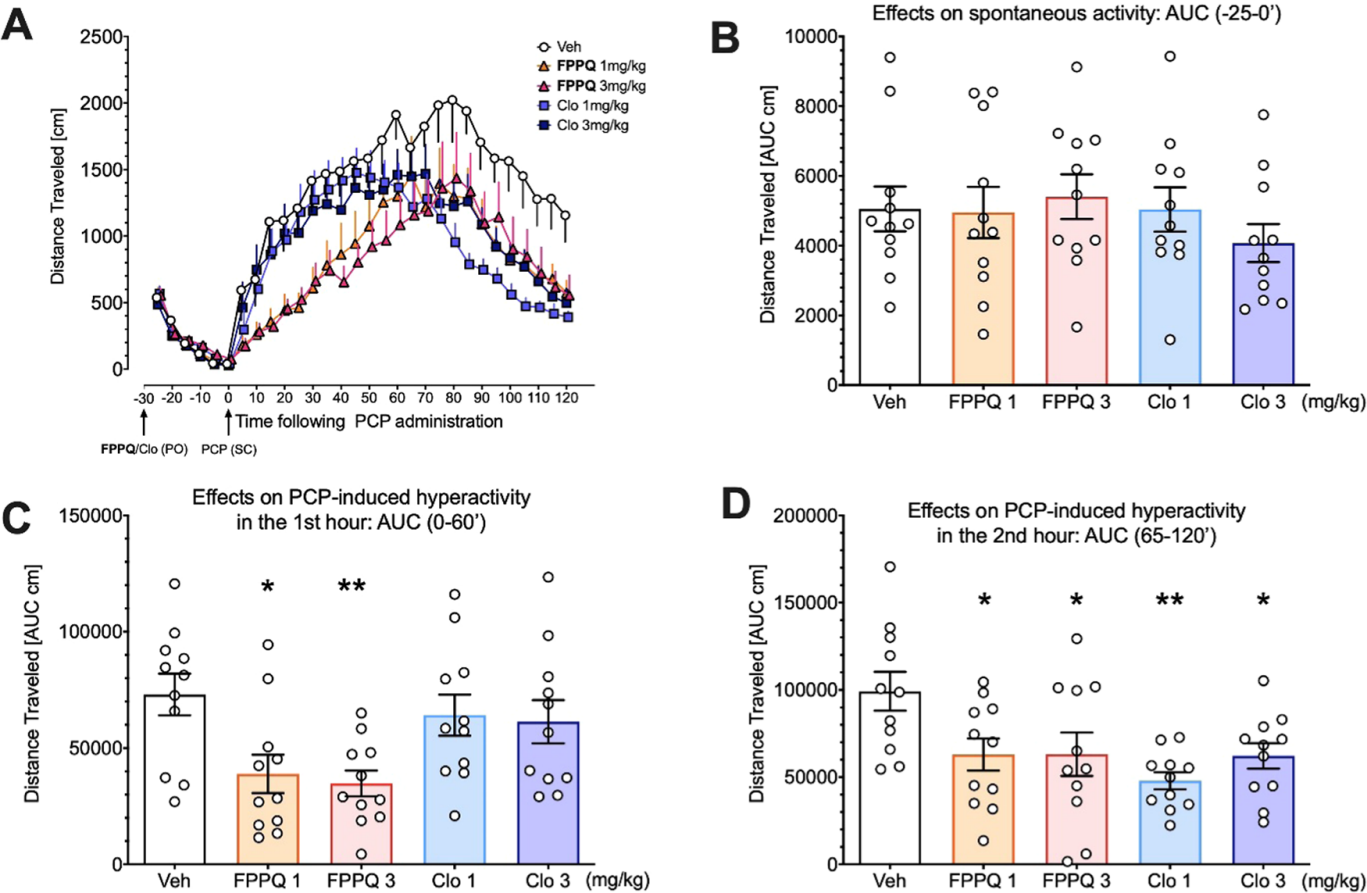
Effects of **FPPQ** and clozapine on PCP-induced hyperactivity (0–120 min following PCP administration) (A). Panel (B) shows no effects on spontaneous locomotor activity (−25 to 0 min before PCP administration) suggesting that neither the **FPPQ** compound nor clozapine produced sedative action at comparable doses. However, **FPPQ** but not clozapine attenuated PCP-induced hyperactivity at the 1st hour following PCP administration (C), while both compounds were active at the 2nd hour following PCP administration (D). Values present mean +, −, or ± SEM of 5 min epochs (A) or the area under the curve (AUC; **B, C, D**). Symbols: *p < 0.05 **p < 0.01 vs vehicle+PCP (Veh), Dunnett’s post hoc test. For each group, *N* = 11 rats.

We first examined the ability of **FPPQ** to affect PCP-induced hyperactivity in male Sprague–Dawley rats. The wildly used antipsychotic clozapine was used as a “positive” control. Neither **FPPQ** nor clozapine affected spontaneous activity analyzed with the use of Area Under the Curve (AUC) on −25 to 0 min before PCP administration, suggesting no sedative effects in the present experimental conditions (Figure 8B, *F*(4,50)=0.5951, NS).

As expected, PCP administration significantly increased locomotor activity (Figure 8A). Mixed-design ANOVA with treatment as between-subject factor and time as repeated factor revealed that treatment affected PCP-induced hyperactivity during 0-120 min following PCP administration (time × treatment interaction: *F*(92,1150)=2.184; *p* < 0.05).

Detailed analysis of the AUC activity data measured for the 0–60 and 65–120 min post-PCP treatment periods revealed that during the initial 0–60 min, treatment affected activity (*F*(4,50)=4.063; *p* < 0.01) and that **FPPQ** decreased PCP-induced hyperactivity at both doses (1 and 3 mg/kg) while clozapine at the same doses appeared to be ineffective (Figure 8C). During the second hour following PCP administration, treatment also affected activity (*F*(4,50)=4.125; *p* < 0.01) and both **FPPQ** and clozapine decreased PCP-induced hyperactivity at 1 and 3 mg/kg (Figure 8D).

Since clozapine acts as an antagonist at both 5-HT_3_ and 5-HT_6_Rs, we hypothesized that simultaneous blockade of these serotonin receptors might be responsible for the “anti-PCP” effects observed for **FPPQ**. We thus examined more directly whether dual 5-HT_6_R and 5-HT_3_R antagonistic activity as presented by **FPPQ** and by clozapine, could produce antipsychotic-like activity.

To this end, we first assessed the effects of SB399885, a 5-HT_6_R antagonist (which behaves as inverse agonist) alone on PCP-induced hyperactivity. It is known that the 5-HT_6_R antagonists produce no consistent antipsychotic-like effects, and could even potentiate amphetamine-induced hyperactivity.^46^ The goal of this experiment was to establish whether in PCP conditions the compound would produce similar or different effects.

**Figure 9:**
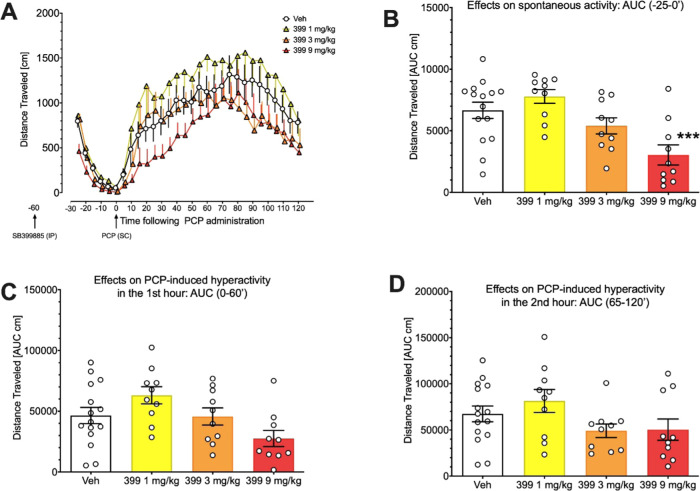
Effects of 5-HT_6_R antagonist SB399885 on spontaneous locomotor activity (-25 to 0 min before PCP administration) (B) and on PCP-induced hyperactivity (0–120 min following PCP administration) (A) suggest that the SB399885 compound at the highest dose of 9 mg/kg inhibited spontaneous activity. Pretreatment with SB399885 compound affected PCP-induced hyperactivity neither at the first (C) nor at the second (D) hour following PCP administration. Data are expressed as mean +, -, or ± SEM of 5 min epochs (A) and mean ± SEM of the area under curve; (AUC; B, C, D). For each group, *N* = 15–10 rats. Symbols: ***p < 0.001, Dunnet’s post hoc test vs. vehicle.

Figure 9A shows robust hyperactivity due to PCP administration. Mixed-design ANOVA demonstrated not significant interaction between time and SB399885 dose (*F*(69,943) = 0.632; NS) but significant effects of SB399885 dose (*F*(3,41)=3.467; *p* = 0.025). Analysis of the AUC at times -25 to 0 min (i.e., 30–60 min following SB399885 administration) revealed effects of treatment on spontaneous activity (*F*(3,41) = 8.079, *p* < 0.001) and its inhibition by SB399885 at 9 mg/kg, suggesting sedative-like action (Figure 9B).

Detailed analyses of SB399885 actions with the use of AUCs (Figure 9C,D) revealed however that this 5-HT_6_R antagonist did not affect PCP-induced hyperactivity. While ANOVA demonstrated that the treatments affected activity at the 1st hour (*F*(3,41)=3.907, *p* < 0.05), Dunnett’s post hoc test demonstrated no significant differences vs. vehicle. ANOVA for the 2nd hour was insignificant: *F*(3,41) = 2.140, *p* = 0.10.

Based on the results of this experiment, we attempted to examine the effects of a joint administration of the 5-HT_6_R antagonist SB399885 and 5-HT_3_R antagonist ondansetron on PCP-induced hyperactivity. The dose of SB399885 compound was set at 1 mg/kg as it certainly did not inhibit PCP hyperactivity (and even, insignificantly enhanced it, Figure 9A); see elsewhere.^47,48^ The dose of ondansetron (0.5 mg/kg) was chosen based on Pehrson et al.^49^ work and on du Jardin et al.,^50^ suggestion implicating that at 1.6 mg/kg, ondansetron is expected to produce 60% or greater occupancy at the 5-HT_3_R. We thus decided to use 0.5 mg/kg dose that would likely occupy ∼30% of 5-HT_3_R.

**Figure 10:**
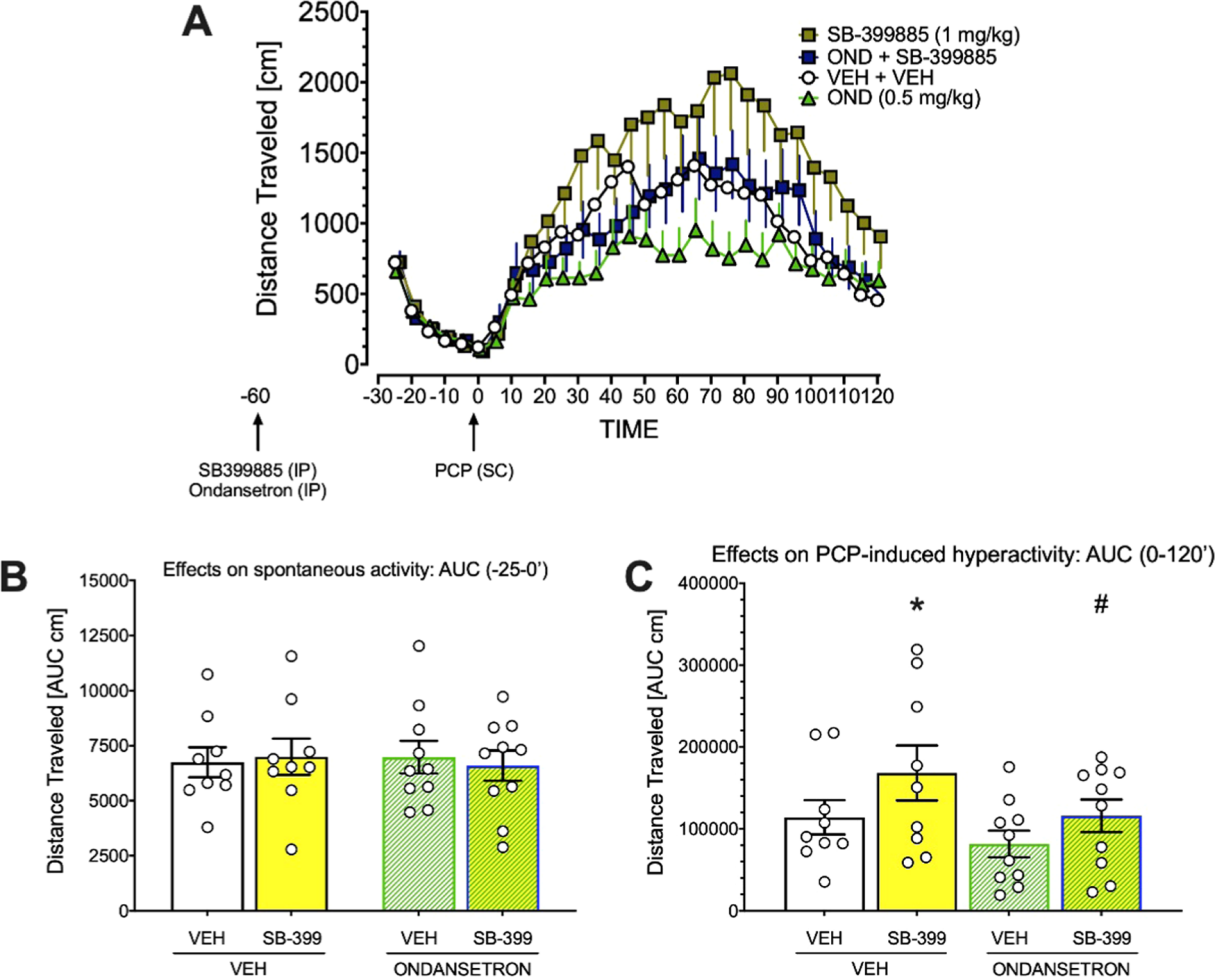
Effects of joint administration of the 5-HT_6_R antagonist SB399885 (1 mg/kg) and of the 5-HT_3_R antagonist ondansetron (0.5 mg/kg) on spontaneous locomotor activity (-25 to 0 min before PCP administration) (B) and on activity 0–120 min following PCP administration (A) suggest that neither compound affected spontaneous activity. (C) Pretreatment with SB399885 compound enhances PCP-induced hyperactivity compared with vehicle, and this enhancement is reduced by joint SB399885 and ondansetron administration. Symbols: *t(68)=2.179; *p* < 0.05 vs vehicle, # *t*(68)=2.158; *p* < 0.05 vs SB399885 only group, planned comparisons. Data are expressed as mean +, – or ± SEM of 5 min epochs (A) or the mean ± SEM of the AUC. For each group, the *N* = 9–10 rats.

As shown in Figure 10B, neither SB399885 at 1 mg/kg nor ondansetron at 0.5 mg/kg affected spontaneous activity (*F*(3,34) = 0.07, NS). However, inspection of the raw data (Figure 10A) suggested that while inhibition of 5-HT_6_Rs appears to enhance PCP-induced hyperactivity, the inhibition of 5-HT_3_Rs likely reduces it. Mixed-design ANOVA with the time as repeated factor and the treatment with both compounds as between-subject factors on the raw data presented in Figure 10B revealed significant effects of time (*F*(23,782) = 23.9443; *p* < 0.001), an interaction between time and ondansetron (*F*(23,782) = 1.9318; *p* < 0.01), an interaction between time and SB399885 (*F*(23,782)=2.2029, *p* < 0.001), but no interaction between time, ondansetron, and SB399885: (*F*(23,782) = 0.6881, NS).

As the a priori hypothesis was that the combined treatment with 5-HT_6_R and 5-HT_3_R antagonists would produce different effects than their individual actions and/or vehicle, we analyzed the SB399885-induced potentiation of PCP hyperactivity and its inhibition by ondansetron addition, using analyses of contrast coefficients^51^ on time-collapsed AUC data. These planned comparisons revealed that 5-HT_6_R antagonist enhanced hyperactivity was reduced in ondansetron+SB399885 group (Figure 10C).

While these results do not provide evidence that the co-administration of ondansetron with SB399885 produces antipsychotic-like effect, they do suggest that combined administration of antagonists of both 5-HT_6_R and 5-HT_3_R could alleviate 5-HT_6_R antagonist-induced potentiation of PCP-induced hyperactivity.

Finally, we assessed the interaction between another 5-HT_6_R antagonist CPPQ (which behaves as a neutral antagonist) and ondansetron on hyperactivity evoked by PCP. The dose of ondansetron (0.5 mg/kg) was the same as in the previous experiment while the doses of CPPQ (0.3, 1, and 3 mg/kg) were based on the previous report.^29^

**Figure 11:**
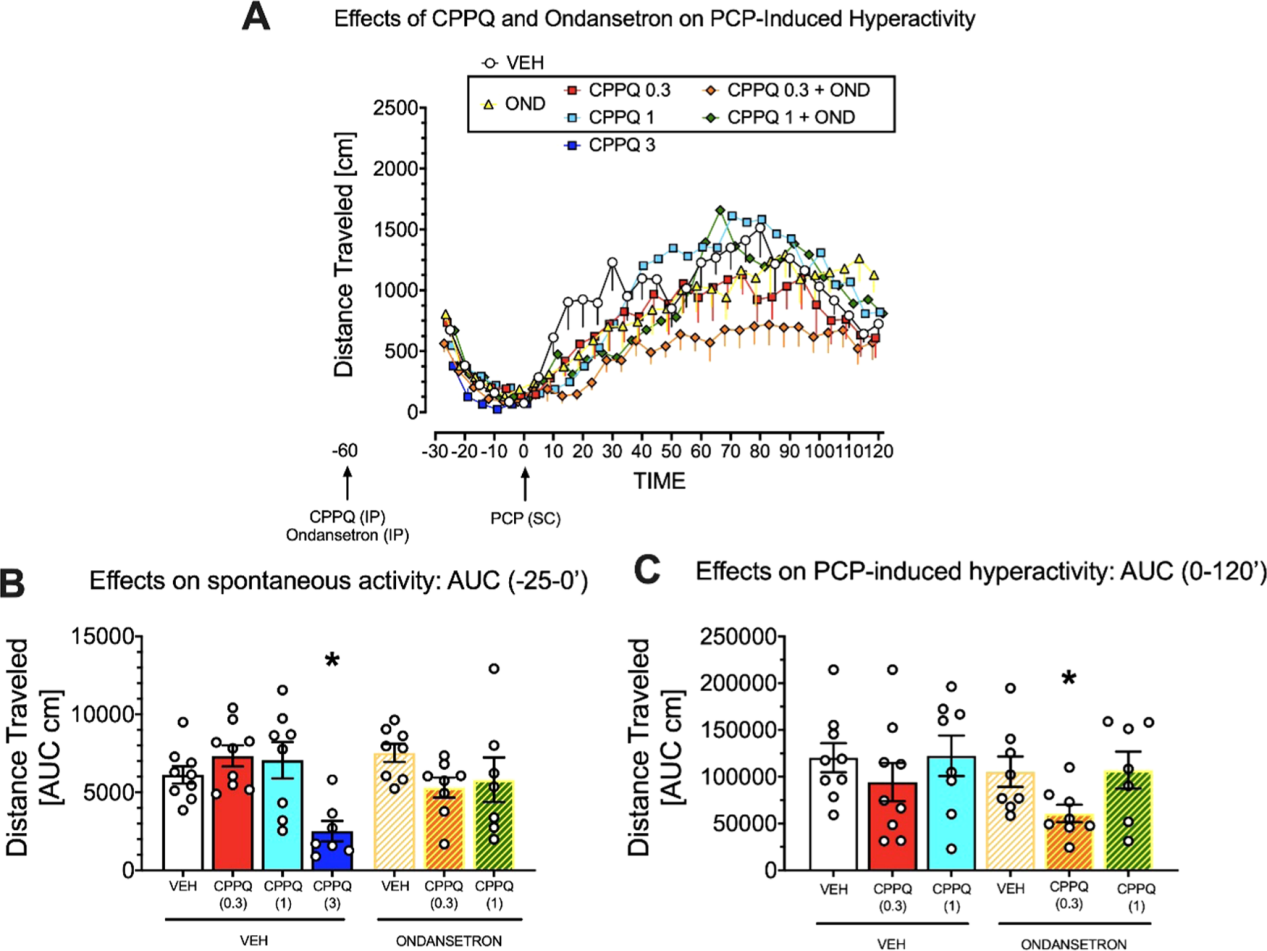
Effects of joint administration of 5-HT_6_R antagonist CPPQ and of 5-HT_3_R antagonist ondansetron on PCP-induced hyperactivity. (A) Mean – SEM raw data in 5 min epochs. (B) Mean ± SEM AUC spontaneous locomotor activity (−25 to 0 min before PCP administration) and 3 mg/kg CPPQ dose inhibited spontaneous activity (*p < 0.05 vs vehicle, Dunnet’s post hoc test); this group was not included in the final analyses. (C) Mean ± SEM 0-120 min following PCP administration AUC activity data; their analysis with contrast coefficients revealed that combined treatment with CPPQ (0.3 mg/kg) and ondansetron (0.5 mg/kg) inhibited PCP-induced hyperactivity compared with vehicle (*t(43) = 2.430; *p* = 0.019, planned comparisons test). For each group, *N* = 7–9 rats.

Analysis of the AUC at times -25 to 0 min (i.e., 30–60 min following CPPQ and ondansetron administration) revealed effects of treatment on spontaneous activity (*F*(6,49) = 3.920, *p* = 0.003) and its inhibition by CPPQ at 3 mg/kg, suggestive of sedative-like action of this dose (Figure 11A,B). Thus, data of the 3 mg/kg CPPQ group were not taken for further analyses.

Figure 11A shows robust hyperactivity due to PCP administration. Two-way ANOVA on 0–120 min AUC post-PCP activity data with CPPQ dose and ondansetron as between-subject factors revealed no effects of ondansetron (*F*(1,43)=2.153; NS), CPPQ dose (*F*(2,43)=2.864; *p* = 0.068), nor their interaction (*F*(2,43)=0.184; NS).

However, as the a priori hypothesis was that the combined treatment with 5-HT_6_R and 5-HT_3_R antagonists would produce different effects than their individual actions and/or vehicle, we analyzed 0–120 min AUC activity data with contrast coefficients. These planned comparisons revealed that combined treatment with CPPQ (0.3 mg/kg) and ondansetron (0.5 mg/kg) inhibited PCP-induced hyperactivity compared with vehicle (Figure 11C). Of note, CPPQ alone *per se* did not increase activity, in contrast to SB399885 (see Figure 10C).

**Figure 12:**
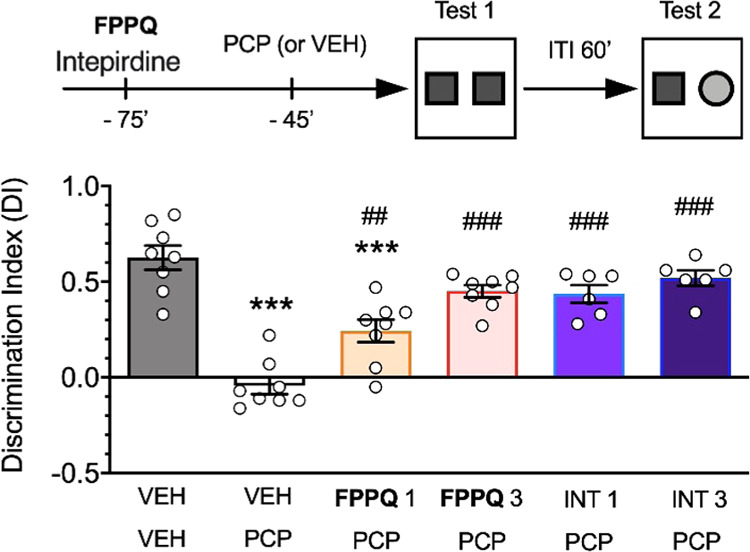
Effects of **FPPQ** on PCP-induced cognitive deficits in the NOR task suggest that like intepirdine, **FPPQ** prevents learning impairment and displays decent in vivo effects following oral administration in rats. Orally administered **FPPQ** and intepirdine (SB-742457) 30 min before PCP prevented memory deficits induced by phencyclidine (PCP, 5 mg/kg; N = 6–8 rats/group). Data are expressed as mean ± SEM of the discrimination index and drug doses, expressed as mg/kg, are shown in the legend below the abscissa. Symbols: VEH, vehicle; INT, intepirdine; PCP, phencyclidine; ***p < 0.001 vs control (VEH/VEH), #*p* < 0.05; ##*p* < 0.01 vs VEH/PCP, Tukey’s multiple comparison post hoc test.

A large body of evidence had indicated that cognitive impairment is a pervasive and core pathological component of schizophrenia. Consequently, cognitive impairments have become a high-priority area in antipsychotic development. To evaluate the impact of the investigated compounds on cognitive processes, the novel object recognition (NOR) task is one of the most frequently used models.^52,53^ In this test, **FPPQ** dose-dependently prevented PCP-induced short-term memory deficits when administered 30 min before PCP. The effects of **FPPQ** were similar to those produced by the 5-HT_6_R antagonist intepirdine (*F*(5,38) = 23.98; *p* < 0.0001; Figure 12) and by other 5-HT_6_R antagonists in other reports.^29,54^

It is also worth noting that the selective 5-HT_3_R antagonist ondansetron produced a procognitive effect in the NOR task,^50^ and also showed positive results on cognitive impairment in phase II clinical trials.^13^ In addition, the procognitive effect of the antidepressant drug vortioxetine results from its antagonistic properties at 5-HT_3_R in the GABAergic interneurons of the mPFC.^55,56^ Therefore, blockade of both 5-HT_3_R and 5-HT_6_R may contribute to the procognitive effect of FPPQ.

## Conclusions

Currently used treatments for schizophrenia can effectively control positive symptoms but with some exceptions they display a limited impact on cognitive deficits. Among serotonin receptors subtypes, the 5-HT_2A_R is a clinically validated target. Recent attention has been paid to 5-HT_3_R antagonists to support available treatments. Indeed, ondansetron, an antiemetic 5-HT_3_R antagonist revealed positive effects as adjunctive therapy of schizophrenia,^12,57^ ameliorating both negative symptoms and cognitive decline in patients. In parallel, 5-HT_6_R antagonists have emerged as promising tools to treat cognitive impairment. Specifically, both neutral antagonists and inverse agonists of 5-HT_6_R produce procognitive effects in preclinical and clinical settings.^24,58^ It is worth noting that neither 5-HT_3_R antagonists nor 5-HT_6_R antagonists improve the positive symptoms of psychoses, a feature that is also not addressed by antipsychotics with D_2_ receptor component. Our hybridization strategy proved to be successful in optimizing first-in-class dual-acting 5-HT_3_R/5-HT_6_R antagonist and extended the concept of rational multitarget drug discovery. Among the evaluated series, **FPPQ** displayed balanced low-nanomolar affinity at both receptors, behaved as a 5-HT_3_R antagonist and a neutral antagonist at 5-HT_6_R-dependent Gs signaling and had no influence on receptor-operated Cdk5-dependent neurite growth. **FPPQ** showed favorable selectivity over 87 targets, decent brain penetration, and safety profile, with no propensity to evoke off-target-related side effects. Ultimately, **FPPQ** reversed PCP-induced hyperactivity and displayed procognitive properties in the NOR task. Though respective contribution of blockade of 5-HT_3_R and 5-HT_6_R in antipsychotic-like effects of FPPQ remains to be established, these findings corroborate that combination of 5-HT_3_R antagonism and 5-HT_6_R antagonism, exemplified by FPPQ contribute to the effect observed in PCP-induced hyperactivity. Development of 5-HT_3_/5-HT_6_R antagonists represents a promising approach to respond to the persistent demand for higher efficacy and better compliance in treating drug-resistant schizophrenia symptoms.

## Experimental Section

### Synthesis General Information

The synthesis was carried out at ambient temperature, unless indicated otherwise. Organic solvents (from Sigma-Aldrich and Chempur) were of reagent grade and were used without purification. All reagents (Sigma-Aldrich, Fluorochem) were of the highest purity. Column chromatography was performed using silica gel Merck 60 (70–230 mesh ASTM). The UPLC purity of final compounds was verified by UV spectra and all compounds were confirmed to be ≥95% pure. Mass spectra were recorded on a UPLC-MS/MS system consisted of a Waters ACQUITY UPLC (Waters Corporation, Milford, MA) coupled to a Waters TQD mass spectrometer with electrospray ionization mode ESI-tandem quadrupole (for more information, see the Supporting Information). High-resolution MS measurements were carried out on a Bruker Impact II mass spectrometer (Bruker Corporation, Billerica). ^1^H NMR and ^13^C NMR spectra were recorded using JEOL JNM-ECZR500 RS1 (ECZR version) at 500 and 126 MHz, respectively, as well as Varian BB 200 spectrometer at 300 and 75 MHz, and are reported in ppm using deuterated solvent for calibration (CDCl_3_, methanol*-d*_4_ or DMSO-*d*_6_). The *J* values are given in Hertz (Hz). Elemental analysis for C, H, N, and S was performed on the elemental Vario EI III Elemental Analyser (Hanau, Germany). All values are reported as percentages, and were within ±0.4% of the calculated values.

Compounds **1**–**4** were obtained according to the previously reported procedure and the analytical data are in accordance with the literature.^29^

### General Procedure for Preparation of Compounds **5a**–**5b**

Compound **4** (0.35 g, 1.7 mmol, 1 equiv) was dissolved in acetonitrile followed by addition of amine (1.26 g, 6.8 mmol, 4 equiv). The reaction was heated in a microwave at 140 °C for 5h. The solvent was evaporated, and the crude product was purified by chromatography using silica gel with CH_2_Cl_2_/MeOH 9/1.5 (v/v) as a developing solvent.

#### *tert*-Butyl-(*S*)-3-((1*H*-pyrrolo[3,2-c]quinolin-4-yl)amino)pyrrolidine-1-carboxylate (**5a**)

Pale oil, 60% yield, *t*_R_ = 4.38, C_20_H_24_N_4_O_2_, MW 352.43. ^1^H NMR (300 MHz, CDCl_3_/methanol*-d*_4_) δ (ppm) 1.42 (s, 9H), 1.92–2.11 (m, 1H), 2.29–2.4 (m, 1H) 3.31–3.62 (m, 4H), 3.70–3.79 (m, 1H), 4.92 (bs, 1H), 6.54 (d, *J* = 3.1 Hz, 1H), 7.12 (d, *J* = 3.1 Hz, 1H), 7.12–7.23 (m, 1H), 7.36 (s, 1H), 7.77 (d, *J* = 8.5 Hz, 1H), 7.81 (dd, *J* = 7.8, *J* = 1.3 Hz, 1H). Monoisotopic mass 352.19, [M + H]^+^ 353.2. HRMS calcd for C_20_H_25_N_4_O_2_, 353.1978, found, 353.1978.

#### *tert*-Butyl-3-((1*H*-pyrrolo[3,2-c]quinolin-4-yl)amino)azetidine-1-carboxylate (**5b**)

Pale oil, 53% yield, *t*_R_ = 4.21, C_19_H_22_N_4_O_2_, MW 338.41. ^1^H NMR (300 MHz, CDCl_3_/ methanol*-d*_4_) δ (ppm) 1.22 (s, 9H), 1.28–1.41 (m, 2H), 4.40–4.46 (m, 2H), 4.48–4.56 (m, 1H), 6.80–6.87 (m, 1H), 7.08–7.12 (d, 1H), 7.21–7.26 (m, 1H), 7.28–7.32 (m, 1H), 7.42 (bs, 1H), 8.12–8.16 (m, 1H). Monoisotopic mass 338.12, [M + H]^+^ 339.1.

#### General Procedure for Preparation of Compounds **5c**–**5f**

Compound **4** (0.35 g, 1.7 mmol, 1 equiv) was suspended in a mixture of toluene (30 mL) and TEA (1.4 mL, 10.2 mmol, 6 equiv). Subsequently, an appropriate amine (2.4 mmol, 2 equiv, 0.27 mL) was added and the reaction was heated at 114 °C for 14 h. The reaction mixture was evaporated, and the remaining crude product was purified by chromatography on silica gel using CH_2_Cl_2_/MeOH 9/1 (v/v) as a developing solvent.

#### *tert*-Butyl (1-(1*H*-pyrrolo[3,2-c]quinolin-4-yl)pyrrolidin-3-yl)(methyl)carbamate (**5c**)

Pale oil, 61% yield, *t*_R_ = 4.88, C_21_H_26_N_4_O_2_, MW 366.47. ^1^H NMR (300 MHz, CDCl_3_) δ (ppm) 1.09 (s, 3H), 1.52 (s, 9H), 2.10–2.40 (m, 3H), 4.09–4.21 (m, 4H), 6.85–6.93 (m, 1H), 7.02–7.09 (m, 1H), 7.21–7.25 (m, 1H), 7.34–7.41 (m, 1H), 7.68–7.75 (m, 1H), 7.89–8.10 (m, 1H). Monoisotopic Mass 366.21, [M + H]^+^ 367.3.

#### 4-(4-Methylpiperazin-1-yl)-1*H*-pyrrolo[3,2-c]quinoline (**5d**)

Pale oil, 62% yield, *t*_R_ = 1.48, C_16_H_18_N_4_, MW 266.34. ^1^H NMR (500 MHz, CDCl_3_) δ (ppm) 2.35 (s, 3H), 2.51–2.61 (m, 4H), 3.81–3.92 (m, 4H), 6.62 (s, 1H), 7.12–7.24 (m, 2H), 7.25–7.40 (m, 1H), 7.75–7.80 (m, 2H), 9.77 (bs, 1H). ^13^C NMR (126 MHz, methanol*-d*_4_) δ ppm 42.5, 46.2, 52.6, 106.3, 108.9, 1141, 118.6, 121.0, 126.1, 126.2, 129.6, 133.4, 138.6, 150.6. Monoisotopic Mass 266.15, [M + H]^+^ 267.2. HRMS calcd for C_16_H_19_N_4_ 267.1610, found: 267.1610.

#### 4-(Piperazin-1-yl)-1*H*-pyrrolo[3,2-c]quinoline (**5e**)

##### Boc-derivative (*tert*-Butyl 4-(1*H*-pyrrolo[3,2-c]quinolin-4-yl)piperazine-1-carboxylate)

Pale oil, 63% yield, *t*_R_ = 4.33, C_20_H_24_N_4_O_2_, MW 352.43. ^1^H NMR (300 MHz, CDCl_3_) δ (ppm) 1.50 (s, 9H), 3.52–3.75 (m, 4H), 3.76–3.92 (m, 4H), 6.65 (d, *J* = 3.1 Hz, 1H), 7.15–7.25 (m, 2H), 7.31–7.42 (m, 1H), 7.83–7.91 (m, 2H), 9.75 (bs, 1H). ^13^C NMR (75 MHz, DMSO-*d*_6_) δ (ppm) 28.5, 47.7, 79.4, 103.8, 111.6, 116.3, 120.6, 122.6, 123.3, 126.7, 127.3, 136.73, 143.2, 154.5, 154.5. Monoisotopic Mass 352.19, [M + H]^+^ 353.4. HRMS calcd for 353.1978; found 353.1977.

##### Hydrochloride Salt

White solid, 67% yield, *t*_R_ = 1.40, C_15_H_17_ClN_4_, MW 288.78. ^1^H NMR (500 MHz, methanol*-d*_4_) δ (ppm) 3.58 (bs, 4H), 4.36 (bs, 4H), 7.14 (s, 1H), 7.56–7.63 (m, 2H), 7.70 (t, *J* = 7.3 Hz, 1H), 8.06 (d, *J* = 8.3 Hz, 1H), 8.23–8.27 (m, 1H). ^13^C NMR (126 MHz, methanol*-d*_4_) δ ppm 40.4, 42.9, 45.9, 106.4, 108.8, 114.1, 118.5, 121.0, 126.0, 126.2, 129.5, 133.3, 138.5, 150.8. Monoisotopic Mass 252.1, [M + H]^+^ 253.2. HRMS calcd for C_16_H_19_N_4_ 252.3210, found: 253.1456.

#### *tert*-Butyl-4-(1*H*-pyrrolo[3,2-c]quinolin-4-yl)-1,4-diazepane-1-carboxylate (**5f**)

Pale oil, 85% yield, *t*_R_ = 4.84, C_21_H_26_N_4_O_2_, MW 366.46. ^1^H NMR (300 MHz, CDCl_3_/methanol*-d*_4_) δ (ppm) 1.41 (s, 9H), 1.51–1.73 (m, 2H), 1.90–2.21 (m, 2H), 3.24–3.56 (m, 2H), 3.73 (bs, 1H), 4.01–4.34 (m, 3H), 6.70 (bs, 1H), 7.05–7.21 (m, 1H), 7.25–7.45 (m, 3H), 7.81 (t, *J* = 7.6 Hz, 1H). Monoisotopic Mass 366.21, [M + H]^+^ 367.2.

### General Procedure for the Preparation of Compounds **6**–**28**

Compounds **5a**–**5f** (0.28 mmol, 1 equiv) were dissolved in CH_2_Cl_2_ (5 mL) and BTPP (170 μL, 0.56 mmol, 2 equiv) was added. The mixture was placed in an ice bath, sulfonyl chloride (1.8 equiv) was added, and the reaction mixture was stirred for 3 h. Subsequently, the mixture was evaporated and the remaining crude product was purified by chromatography on silica gel. The Boc-protected derivatives were treated with 1 N HCl solution in MeOH to give HCl salts of secondary amines.

#### 1-(Phenylsulfonyl)-4-(4-methylpiperazin-1-yl)-1*H*-pyrrolo[3,2-c]quinoline hydrochloride (**6**)

White solid, 80% yield, *t*_R_ = 4.69, Mp = 127–129 °C, C_22_H_23_ClN_4_O_2_S, MW 442.96. ^1^H NMR (500 MHz, methanol*-d*_4_) δ (ppm) 3.06 (s, 3H), 3.50 (bs, 2H), 3.79 (bs, 2H), 4.04 (bs, 2H), 4.67 (bs, 2H), 7.40 (d, *J* = 3.7 Hz, 1H), 7.52–7.60 (m, 3H), 7.68–7.72 (m, 2H), 7.90–7.96 (m, 2H), 8.12 (d, *J* = 8.3 Hz, 1H), 8.29 (d, *J* = 4.0 Hz, 1H), 8.93–8.98 (m, 1 H).^13^C NMR (126 MHz, methanol*-d*_4_) δ ppm 43.5, 47.8, 53.6, 109.0, 115.0, 116.7, 120.8, 125.2, 127.1, 128.3, 130.9, 131.6, 131.8, 136.6, 137.9, 138.6, 151.8. Monoisotopic mass 406.15, [M + H]^+^ 407.3. HRMS calcd for C_22_H_22_N_4_O_2_S 407.1542; found: 407.1542.

#### 1-(Phenylsulfonyl)-4-(piperazin-1-yl)-1*H*-pyrrolo[3,2-c]quinoline dihydrochloride (**7**)

White solid, 90% yield, *t*_R_ = 4.51, Mp 130–132 °C, C_21_H_22_Cl_2_N_4_O_2_S, MW 465.40. ^1^H NMR (500 MHz, methanol*-d*_4_) δ (ppm) 3.48–3.55 (m, 4H), 4.17–4.27 (m, 4H), 7.37–7.40 (m, 1H), 7.51–7.55 (m, 3H), 7.65–7.75 (m, 2H), 7.85–7.90 (m, 2H), 8.12–8.15 (m, 1H), 8.25–8.35 (m, 1H), 8.98 (dd, *J* = 8.6, *J* = 0.9 Hz, 1H). ^13^C NMR (126 MHz, DMSO-*d*_6_) δ ppm 42.9, 46.8, 109.0, 114.1, 116.7, 123.7, 125.5, 126.0, 127.6, 128.2, 129.9, 130.3, 130.9, 136.1, 136.5, 137.1, 152.2. Monoisotopic Mass 392.13, [M + H]^+^ 393.3. HRMS calcd for C_21_H_20_N_4_O_2_S 393.1385; found 393.1387.

#### (*S*)-1-(Phenylsulfonyl)-*N*-(pyrrolidin-3-yl)-1*H*-pyrrolo[3,2-c]quinolin-4-amine dihydrochloride (**8**)

White solid, 80% yield, *t*_R_ = 4.78, Mp 220–222 °C, C_21_H_22_Cl_2_N_4_O_2_S, MW 465.39. ^1^H NMR (300 MHz, methanol*-d*_4_) δ (ppm) 2.43–2.47 (m, 1H), 2.59–2.77 (m, 1H), 3.46–3.52 (m, 2H), 3.53–4.01 (m, 2H), 5.18 (bs, 1H), 7.41–7.51 (m, 4H), 7.53–7.64 (m, 2H), 7.73–7.91 (m, 3H), 8.22 (m, 1H), 8.86 (d, *J* = 7.9 Hz, 1H). ^13^C NMR (75 MHz, DMSO-*d*_6_) δ ppm 31.2, 44.2, 49.1, 52.7, 108.9, 112.9, 115.9, 120.3, 123.7, 125.6, 126.4, 127.2, 130.2, 130.7, 132.8, 134.4, 135.3, 136.1, 138.5, 148.4. Monoisotopic Mass 392.13, [M + H]^+^ 393.1. HRMS calcd for C_21_H_20_N_4_O_2_S 392.1307, found 393.1322.

#### *N*-(Azetidin-3-yl)-1-(phenylsulfonyl)-1*H*-pyrrolo[3,2-c]quinolin-4-amine dihydrochloride (**9**)

White solid, 94% yield, *t*_R_ = 3.35, Mp 243–246 °C, C_20_H_20_Cl_2_N_4_O_2_S, MW 451.37. ^1^H NMR (300 MHz, DMSO-*d*_6_) δ (ppm) 4.31–4.32 (m, 1H), 4.78–5.16 (m, 4H), 6.92–6.94 (m, 1H), 7.10 (s, 2H), 7.31–7.38 (m, 4H), 7.54–7.57 (m, 1H), 7.81 (d, *J* = 8.3 Hz, 1H), 8.01 (d, *J* = 8.3 Hz, 1H), 8.20–8.22 (m, 1H).. Monoisotopic Mass: 378.12, [M + H]^+^ 379.2.

#### *N*-Methyl-1-(1-(phenylsulfonyl)-1*H*-pyrrolo[3,2-c]quinolin-4-yl)pyrrolidin-3-amine dihydrochloride (**10**)

White solid, 89% yield, *t*_R_ = 4.75, Mp 256–258 °C, C_22_H_24_Cl_2_N_4_O_2_S, MW 479.42. ^1^H NMR (500 MHz, DMSO-*d*_6_) δ (ppm) 2.49–2.63 (m, 2H), 3.11 (s, 3H), 3.76–3.91 (m, 1H), 4.16–4.21 (m, 1H), 4.23–4.41 (m, 2H), 4.61 (m, 1H), 5.78–5.94 (m, 1H), 6.81–6.90 (m, 1H), 7.10–7.24 (m, 1H), 7.26–7.41 (m, 3H), 7.47–7.55 (m, 1H), 7.68–7.77 (m, 2H), 7.79–7.81 (m, 1H), 7.87–7.92 (m, 1H). ^13^C NMR (126 MHz, DMSO-*d*_6_) δ ppm 29.5, 48.3, 52.3, 125.0, 126.0, 127.9, 130.9, 131.3, 132.7, 134.0. Monoisotopic Mass: 406.15, [M + H]^+^ 407.2.

#### 4-(1,4-Diazepan-1-yl)-1-(phenylsulfonyl)-1*H*-pyrrolo[3,2-c]quinoline dihydrochloride (**11**)

White solid, 95% yield, *t*_R_ = 5.72, Mp 247–248 °C, C_22_H_24_Cl_2_N_4_O_2_S, MW 513.87. ^1^H NMR (300 MHz, CDCl_3_/methanol*-d*_4_) δ (ppm) 2.25–2.47 (m, 2H), 3.28–3.47 (m, 2H), 3.60–3.75 (m, 2H), 4.15–4.25 (m, 2H), 4.26–4.35 (m, 2H), 6.76–6.81 (m, 1H), 7.18–7.25 (m, 1H), 7.35–7.38 (m, 1H), 7.50–7.68 (m, 3H), 7.73–7.75 (m, 1H), 7.75–7.80 (m, 1H), 8.00–8.12 (m, 1H), 8.25–8.30 (m, 1H), 8.75–8.82 (m, 1H). ^13^C NMR (126 MHz, DMSO-*d*_6_) δ ppm 24.8, 45.1, 46.8, 110.3, 113.0, 115.5, 123.7, 126.5, 127.4, 130.0, 132.9, 135.4, 136.2, 136.5, 138.7. Monoisotopic Mass 406.14. [M + H]^+^ 407.2.

#### 1-[(3-Fluorophenyl)sulfonyl]-4-(4-methylpiperazin-1-yl)-1*H*-pyrrolo[3,2-c]quinoline hydrochloride (**12**)

White solid, 88% yield, *t*_R_ = 4.95, Mp 176–177 °C, C_22_H_22_ClFN_4_O_2_S, MW 460.95. ^1^H NMR (500 MHz, methanol*-d*_4_) δ (ppm) 3.03 (s, 3H), 3.46 (bs, 2H), 3.70 (bs, 2H), 3.96 (bs, 2H), 4.64 (bs, 2H), 7.38 (d, *J* = 4.0 Hz, 1H), 7.42–7.49 (m, 1H), 7.55–7.63 (m, 2H), 7.68–7.74 (m, 3H), 8.09 (dd, *J* = 8.6, *J* = 0.9 Hz, 1H), 8.28 (d, *J* = 4.0 Hz, 1H), 8.93 (dd, *J* = 8.6, *J* = 0.6, 1H). ^13^C NMR (126 MHz, methanol*-d*_4_) δ ppm 42.4, 52.4, 108.4, 113.4, 114.6, 115.8, 119.5, 122.7, 124.0, 126.2, 130.7, 132.3, 135.0, 137.6, 138.5, 150.5, 160.7, 164.1. Monoisotopic Mass 424.14, [M + H]^+^ 425.1. HRMS calcd for C_22_H_22_N_4_O_2_S 425.1453; found 425.1450.

#### 1-[(3-Chlorophenyl)sulfonyl]-4-(4-methylpiperazin-1-yl)-1*H*-pyrrolo[3,2-c]quinoline hydrochloride (**13**)

White solid, 91% yield, *t*_R_ = 4.97, Mp 125–124 °C C_22_H_22_Cl_2_N_4_O_2_S, MW 477.40. ^1^H NMR (500 MHz, methanol*-d*_4_) δ (ppm) 3.03 (s, 3H), 3.38–3.61 (m, 2H), 3.63–3.78 (m, 2H), 3.81–4.18 (m, 2H), 4.52–4.68 (m, 2H), 7.39 (d, *J* = 4.0 Hz, 1H), 7.54 (t, *J* = 8.0 Hz, 1H), 7.56–7.63 (m, 1H), 7.65–7.71 (m, 1H), 7.71–7.78 (m, 1H), 7.84–7.90 (m, 1H), 7.95 (t, *J* = 1.9 Hz, 1H), 8.06–8.12 (m, 1H), 8.28 (d, *J* = 4.0 Hz, 1H), 8.93 (dd, *J* = 8.6, *J* = 0.9 Hz, 1H). ^13^C NMR (126 MHz, methanol*-d*_4_) δ ppm 46.4, 56.5, 112.5, 117.4, 119.8, 123.4, 127.8, 129.9, 130.3, 131.0, 134.9, 135.6, 138.8, 139.5, 139.8, 141.4, 142.4, 154.6. Monoisotopic Mass 440.11, [M + H]^+^ 441.2. HRMS calcd for C_22_H_22_ClN_4_O_2_S 441.1152; found: 441.1151.

#### 1-((4-Fluorophenyl)sulfonyl)-4-(4-methylpiperazin-1-yl)-1*H*-pyrrolo[3,2-c]quinoline hydrochloride (**14**)

Yellow oil, 80% yield, *t*_R_ = 4.94, C_22_H_22_ClFN_4_O_2_S, MW 460.95. ^1^H NMR (500 MHz, methanol*-d*_4_δ (ppm) 2.35 (s, 3H), 2.61–2.65 (m, 4H), 3.60–3.67 (m, 4H), 6.91–6.99 (m, 1H), 7.15–7.18 (m, 2H), 7.25–7.30 (m, 1H), 7.38–7.49 (m, 1H), 7.75–7.81 (m, 3H), 7.88–7.92 (m, 1H), 8.78 (d, *J* = 8.0 Hz, 1H). ^13^C NMR (126 MHz, DMSO-*d*_6_) δ ppm 46.3, 48.6, 55.1, 108.4, 115.1, 117.5, 118.0, 123.1, 123.8, 128.4, 129.1, 130.9, 133.9, 136.2, 145.3, 154.8, 164.8, 166.8. Monoisotopic Mass 424.14, [M + H]^+^ 425.3. HRMS calcd for C_22_H_22_N_4_O_2_S 425.1453; found 425.1452.

#### 1-[(2-Bromophenyl)sulfonyl]-4-(piperazin-1-yl)-1*H*-pyrrolo[3,2-c]quinoline dihydrochloride (**15**)

White solid, 92% yield, *t*_R_ = 4.65, Mp 158–160 °C, C_21_H_21_BrCl_2_N_4_O_2_S, MW 544.29. ^1^H NMR (300 MHz, CDCl_3_/methanol*-d*_4_) δ (ppm) 3.40–3.45 (m, 4H), 4.22–4.25 (m, 4H), 7.04–7.25 (m, 2H), 7.27–7.62 (m, 4H), 7.90–8.20 (m, 2H), 8.21–8.25 (m, 1H), 8.26–8.43 (m, 1H). ^13^C NMR (75 MHz, DMSO-*d*_6_) δ ppm 43.0, 46.8, 51.6, 109.1, 114.5, 117.3, 118.8, 121.4, 123.1, 127.2, 128.4, 130.4, 131.7. Monoisotopic Mass 470.04, [M + H]^+^ 471.2, 473.2. HRMS calcd for C_21_H_20_BrN_4_O_2_S 471.0487; found: 471.0490.

#### 1-[(2-Chlorophenyl)sulfonyl]-4-(piperazin-1-yl)-1*H*-pyrrolo[3,2-c]quinoline dihydrochloride (**16**)

White solid, 92% yield, *t*_R_ = 5.21, Mp 157–159 °C, C_21_H_21_Cl_3_N_4_O_2_S, MW 526.92. ^1^H NMR (300 MHz, methanol*-d*_4_) δ (ppm) 3.50–3.53 (m, 4H), 4.20–4.27 (m, 4H), 7.25–7.31 (s, 1H), 7.43–7.54 (m, 2H), 7.56–7.84 (m, 2H), 7.81 (m, 1H), 7.88 (m, 1H), 8.25 (m, 1H), 8.75 (m, 1H), 8.88 (s, 1H). ^13^C NMR (75 MHz, methanol*-d*_4_) δ ppm 42.8, 46.5, 108.5, 113.5, 115.9, 119.6, 124.1, 125.9, 127.1, 130.8, 131.6, 135.5, 135.8, 137.6, 138.5, 150.9. Monoisotopic Mass 426.09, [M + H]^+^ 427.3. HRMS calcd for C_21_H_20_ClN_4_O_2_S 427.0995; found: 427.0990.

#### 1-[(3-Fluorophenyl)sulfonyl]-4-(piperazin-1-yl)-1*H*-pyrrolo[3,2-c]quinoline dihydrochloride (**17**) **FPPQ**

White solid, 96% yield, *t*_R_ = 4.76, Mp 192–193 °C. Anal. calcd for C_21_H_21_Cl_2_FN_4_O_4_S × 2H_2_O: C: 48.56, H: 4.85, N: 10.79, S: 6.17; found: C: 48.57, H: 5.01, N: 10.71, S: 6.11. MW 519.41. ^1^H NMR (300 MHz, methanol*-d*_4_) δ ppm 3.64 (t, *J* = 5.0 Hz, 4H), 4.38 (t, *J* = 5.0 Hz, 4H), 7.31–7.51 (m, 2H) 7.53–7.65 (m, 2H) 7.72–7.78 (m, 3H), 8.18–8.21 (m, 1H), 8.28–8.31 (m, 1H), 8.88–8.91 (m, 1H). ^13^C NMR (75 MHz, DMSO-*d*_6_) δ ppm 42.8, 46.9, 109.4, 113.6, 115.2, 116.7, 119.3, 122.0, 123.6, 124.1, 125.8, 130.5, 133.4, 136.6, 137.6, 138.6, 150.7, 151.7, 160.5, 163.9. Monoisotopic Mass 410.12, [M + H]^+^ 411.1. HRMS calcd for C_21_H_19_FN_4_O_2_S 411.1296; found: 411.1292.

#### 1-[(3-Chlorophenyl)sulfonyl]-4-(piperazin-1-yl)-1*H*-pyrrolo[3,2-c]quinoline dihydrochloride (**18**)

White solid, 95% yield, *t*_R_ = 5.19, Mp 161–163 °C, Anal. calcd for C_21_H_21_Cl_3_N_4_O_2_S: C: 50.46, H: 4.23, N: 11.21, S: 6.42; found: C: 50.85, H: 4.64, N: 11.37, S: 6.82, MW 499.84. ^1^H NMR (300 MHz, CDCl_3_/methanol*-d*_4_) δ (ppm) 3.45 (bs, 4H), 4.28 (bs, 4H), 7.19 (s, 1H), 7.25–7.51 (m, 3H), 7.56–7.61 (m, 2H), 7.68 (s, 1H), 8.00 (s, 1H), 8.29 (d, *J* = 8.5 Hz, 1H), 8.75 (d, *J* = 8.2 Hz 1H). ^13^C NMR (75 MHz, DMSO-*d*_6_) δ (ppm) 42.8, 46.8, 109.4, 113.7, 116.8, 123.6, 125.7, 126.4, 127.2, 130.4, 132.7, 135.3, 136.1, 138.5, 151.8. Monoisotopic Mass 426.09, [M + H]^+^ 427.0, 429.0. HRMS calcd for C_21_H_20_ClN_4_O_2_S 427.0995; found: 427.0989.

#### 1-{[3-(Trifluoromethyl)phenyl]sulfonyl}-4-(piperazin-1-yl)-1*H*-pyrrolo[3,2-c]quinoline dihydrochloride (**19**)

White solid, 92% yield, *t*_R_ = 5.49, Mp 182–185 °C, C_22_H_21_Cl_2_F_3_N_4_O_2_S, MW 533.39. ^1^H NMR (300 MHz, methanol*-d*_4_) δ (ppm) 3.52–3.56 (m, 4H), 4.25–4.31 (m, 4H), 6.53 (m, 2H), 7.22 (m, 1H), 7.51–7.65 (m, 3H), 7.75 (t, *J* = 7.8 Hz, 1H), 8.10–8.21 (m, 2H), 9.15 (m, 1H). ^13^C NMR (75 MHz, DMSO-*d*_6_) δ (ppm) 43.0, 46.4, 109.3, 114.6, 117.3, 122.5, 123.3, 125.2, 129.5, 130.1, 131.7, 132.6, 136.5, 138.3. Monoisotopic Mass 460.12, [M + H]^+^ 461.0. HRMS calcd for C_22_H_20_F_3_N_4_O_2_S 461.1259; found: 461.1261.

#### 1-[(3-Methylphenyl)sulfonyl]-4-(piperazin-1-yl)-1*H*-pyrrolo[3,2-c]quinoline dihydrochloride (**20**)

White solid, 80% yield, *t*_R_ = 4.92, Mp 179–181 °C, C_22_H_24_Cl_2_N_4_O_2_S, MW 479.42. ^1^H NMR (300 MHz, methanol*-d*_4_) δ (ppm) 2.24–2.34 (m, 3H), 3.51–3.53 (m, 4H) 4.24–4.26 (m, 4H), 7.35 (d, *J* = 3.8 Hz, 2H), 7.46–7.48 (m, *J* = 15.0, 7.4 Hz, 1H), 7.50–7.51 (m, 1H), 7.68–7.75 (m, 3H), 8.11–8.21 (m, 1H), 8.25–8.26 (m, 1H), 8.88–8.91 (m, 1H). ^13^C NMR (75 MHz, methanol*-d*_4_) δ (ppm) 19.7, 42.7, 46.4, 107.9, 113.4, 115.4, 119.3, 124.3, 124.4, 125.9, 127.3, 129.7, 130.7, 134.9, 136.0, 136.6, 137.4, 140.8, 150.7. Monoisotopic Mass 406.15, [M + H]^+^ 407.1. HRMS calcd for C_22_H_23_N_4_O_2_S 407.1542; found: 407.1538.

#### 1-[(3-Methoxyphenyl)sulfonyl]-4-(piperazin-1-yl)-1*H*-pyrrolo[3,2-c]quinoline dihydrochloride (**21**)

White solid, 91% yield, *t*_R_ = 4.82, C_22_H_24_Cl_2_N_4_O_3_S, MW 495.42. ^1^H NMR (300 MHz, CDCl_3_/methanol*-d*_4_) δ (ppm) 3.49 (bs, 4H), 3.72 (s, 3H), 4.25 (bs, 4H), 7.18–7.25 (m, 1H), 7.27–7.30 (m, 2H), 7.36–7.41 (m, 2H), 7.52–7.59 (m, 1H), 7.68–7.75 (m, 1H), 8.09–8.12 (m, 1H), 8.24–8.27 (m, 1H), 8.96–9.00 (m, 1H).^13^C NMR (75 MHz, DMSO-*d*_6_) δ (ppm) 42.9, 46.8, 56.5, 109.1, 112.4, 114.1, 116.8, 119.5, 121.9, 123.8, 125.6, 130.0, 130.4, 132.2, 136.6, 138.1, 152.2, 160.3. Monoisotopic Mass 422.14, [M + H]^+^ 423.1. HRMS calcd for C_22_H_22_N_4_O_3_S 423.1491; found 423.1491.

#### 1-[(4-Fluorophenyl)sulfonyl]-4-(piperazin-1-yl)-1*H*-pyrrolo[3,2-c]quinoline dihydrochloride (**22**)

White solid, 95% yield, *t*_R_ = 4.65, Mp 172–174 °C, C_21_H_21_Cl_2_FN_4_O_2_S, MW 483.39. ^1^H NMR (300 MHz, CDCl_3_/methanol*-d*_4_) δ (ppm) 3.41–3.47 (m, 4H), 4.27–4.35 (m, 4H), 6.98–7.21 (m, 2H), 7.23–7.26 (m, 1H), 7.41–7.48 (m, 1H), 7.54–7.64 (m, 1H), 7.70–7.82 (m, 2H), 7.98–8.05 (m, 1H), 8.24–8.34 (m, 1H), 8.75–8.80 (m, 1H). ^13^C NMR (75 MHz, DMSO-*d*_6_) δ (ppm) 44.3, 49.3, 108.7, 113.2, 115.9, 118.4, 123.9, 131.4, 133.2, 134.5, 148.7, 157.5, 165.2, 167.2. Monoisotopic Mass 410.12, [M + H]^+^ 411.3. HRMS calcd for C_21_H_19_FN_4_O_2_S 411.1296; found 411.1293

#### 1-{[4-(Trifluoromethyl)phenyl]sulfonyl}-4-(piperazin-1-yl)-1*H*-pyrrolo[3,2-c]quinoline dihydrochloride (**23**)

White solid, 91% yield, *t*_R_ = 5.38, Mp 165–167 °C, C_22_H_21_Cl_2_F_3_N_4_O_2_S, MW 533.39. ^1^H NMR (300 MHz, methanol*-d*_4_) δ (ppm) 3.50–3.59 (m, 4H), 4.25–4.37 (m, 4H), 7.30–7.33 (m, 1H), 7.53 (t, *J* = 7.8 Hz, 1H), 7.67–7.75 (t, *J* = 7.8 Hz, 1H), 7.78–7.90 (m, 2H), 8.15–8.38 (m, 3H), 8.25–8.31 (m, 1H), 8.88–8.90 (m, 1H). ^13^C NMR (75 MHz, methanol*-d*_4_) δ (ppm) 42.7, 46.5, 108.7, 113.4, 115.9, 119.5, 123.9, 126.2, 127.1, 128.3, 130.7, 135.1, 135.8, 137.6, 140.5, 150.7. Monoisotopic Mass 460.12, [M + H]^+^ 461.3. HRMS calcd for C_22_H_20_F_3_N_4_O_2_S 461.1259; found: 461.1263.

#### 1-[(4-*iso*-Propylphenyl)sulfonyl]-4-(piperazin-1-yl)-1*H*-pyrrolo[3,2-c]quinoline dihydrochloride (**24**)

White solid, 79% yield, *t*_R_ = 5.62, Mp 212–214 °C, C_24_H_28_Cl_2_N_4_O_2_S, MW 507.47. ^1^H NMR (300 MHz CDCl_3_/methanol*-d*_4_) δ (ppm) 1.01–1.14 (m, 6H), 2.70–2.82 (m, 1H), 3.40–3.57 (m, 4H), 4.23–4.37 (m, 4H), 7.13–7.23 (m, 3H), 7.40 (t, *J* = 7.6 Hz, 1H), 7.51–7.68 (m, 3H), 7.98–8.02 (m, 1H), 8.33 (d, *J* = 8.2 Hz, 1H), 8.83 (d, *J* = 8.5 Hz, 1H). ^13^C NMR (75 MHz, CDCl_3_/methanol*-d*_4_) δ (ppm) 23.2, 34.2, 43.2, 47.1, 108.3, 113.4, 115.0, 120.0, 124.0, 126.2, 127.5, 128.1, 130.7, 133.7, 134.9, 137.7, 150.1, 157.5. Monoisotopic Mass 434.18, [M + H]^+^ 435.3. HRMS calcd for C_24_H_26_N_4_O_2_S 435.1855; found: 435.1854.

#### 1-[(3,4-Difluorophenyl)sulfonyl]-4-(piperazin-1-yl)-1*H*-pyrrolo[3,2-c]quinoline dihydrochloride (**25**)

White solid, 98% yield, *t*_R_ = 5.01, Mp 180–182 °C, C_21_H_20_Cl_2_F_2_N_4_O_2_S, MW 501.38. ^1^H NMR (300 MHz, CDCl_3_/methanol*-d*_4_) δ (ppm) 3.21–3.43 (m, 4H), 4.11–4.45 (m, 4H), 7.20–7.25 (m, 2H), 7.42–7.49 (m, 1H), 7.50–7.61 (m, 3H), 7.99 (d, *J* = 3.3 Hz, 1H), 8.31 (d, *J* = 8.0 Hz, 1H), 8.77 (d, *J* = 7.7 Hz, 1H). ^13^C NMR (75 MHz, DMSO-*d*_6_) δ ppm 43.0, 46.5, 109.3, 117.3, 123.5, 125.5, 127.7, 129.6, 130.2, 133.1, 133.8, 136.5, 137.1, 139.4. Monoisotopic Mass 428.11, [M + H]^+^ 429.2. HRMS calcd for C_21_H_19_F_2_N_4_O_2_S 429.1197; found: 429.1201.

#### 1-[(3,4-Dichlorophenyl)sulfonyl]-4-(piperazin-1-yl)-1*H*-pyrrolo[3,2-c]quinoline dihydrochloride (**26**)

White solid, 80% yield, *t*_R_ = 5.63, Mp 203–205 °C, C_21_H_20_Cl_4_N_4_O_2_S, MW 534.29. ^1^H NMR (300 MHz, CDCl_3_/methanol*-d*_4_) δ (ppm) 3.26–3.49 (m, 4H), 4.27–4.37 (m, 4H), 7.15–7.26 (m, 1H), 7.37–7.55 (m, 3H), 7.56–7.67 (m, 1H), 7.80 (s, 1H), 7.95–8.02 (m, 1H), 8.32 (d, *J* = 8.2 Hz, 1H), 8.77 (d, *J* = 8.2 Hz, 1H). ^13^C NMR (75 MHz, CDCl_3_/methanol*-d*_4_) δ ppm 42.7, 46.6, 108.6, 113.4, 115.4, 120.2, 123.7, 126.3, 126.6, 129.0, 130.6, 131.2, 132.0, 134.7, 135.9, 137.9, 140.7, 150.2. Monoisotopic Mass 460.05, [M + H]^+^ 461.2, 463.2. HRMS calcd for C_21_H_18_Cl_2_N_4_O_2_S 461.0606; found: 461.0605.

#### 1-[(2,5-Difluorophenyl)sulfonyl]-4-(piperazin-1-yl)-1*H*-pyrrolo[3,2-c]quinoline dihydrochloride (**27**)

White solid, 81% yield, *t*_R_ = 4.75 Mp 204–206 °C, C_21_H_20_Cl_2_F_2_N_4_O_2_S, MW 501.38. ^1^H NMR (300 MHz, CDCl_3_/methanol*-d*_4_) δ (ppm) 3.24–3.55 (m, 4H), 4.31–4.37 (m, 4H), 6.98–7.12 (m, 1H), 7.13–7.25 (m, 1H), 7.25–7.38 (m, 1H), 7.40–7.45 (m, 1H), 7.50–7.67 (m, 1H), 7.75–7.80 (m, 1H), 7.99–8.12 (m, 1H), 8.36 (d, *J* = 8.2 Hz, 1H), 8.63 (d, *J* = 8.2 Hz, 1H). ^13^C NMR (75 MHz, CDCl_3_) δ ppm 43.2, 44.3, 48.9, 53.5, 106.8, 115.2, 117.1, 119.2, 122.8, 123.9, 127.0, 128.1, 128.6, 136.5, 145.5, 154.1, 155.0, 156.7, 158.7. Monoisotopic Mass 428.11, [M + H]^+^ 429.2. HRMS calcd for C_26_H_27_F_2_N_4_O_4_S 429.1197; found: 429.1196.

#### 1-(Naphthalen-1-ylsulfonyl)-4-(piperazin-1-yl)-1*H*-pyrrolo[3,2-c]quinoline dihydrochloride (**28**)

White solid, 86% yield, *t*_R_ = 5.17, Mp 218–220 °C, C_25_H_24_Cl_2_N_4_O_2_S, MW 515.45. ^1^H NMR (300 MHz CDCl_3_/methanol*-d*_4_) δ (ppm) 3.36–3.56 (m, 4H), 4.21–4.42 (m, 4H), 7.18–7.26 (m, 2H), 7.35–7.44 (d, *J* = 6.2 Hz, 1H), 7.46–7.62 (m, 3H), 7.85 (d, *J* = 7.7 Hz, 1H), 7.95–8.12 (m, 3H), 8.24 (t, *J* = 8.1 Hz, 2H), 8.60 (d, *J* = 8.7 Hz, 1H). ^13^C NMR (75 MHz, CDCl_3_/methanol*-d*_4_) δ ppm 42.7, 46.6, 107.6, 113.1, 114.4, 119.5, 122.4, 123.8, 124.5, 126.1, 127.5, 127.8, 129.5, 129.7, 130.4, 130.5, 130.8, 131.2, 134.0, 134.7, 137.3, 138.0, 150.1. Monoisotopic Mass 442.15, [M + H]^+^ 443.3. HRMS calcd for C_25_H_23_N_4_O_2_S 443.1542; found: 443.1544.

### *In Silico* Simulations

#### Preparation of the Proteins

The construction of the 5-HT_6_R homology models has been described in detail elsewhere.^59^ The 5-HT_3_R co-crystallized with granisetron (PDB code 6NP0) was retrieved from the PDB database.^36^ Protein Preparation Wizard was used to assign bond orders, appropriate amino acid ionization states, and to check for steric clashes.

#### Molecular Docking

The three-dimensional structures of the ligands were prepared using LigPrep, and the appropriate ionization states at pH 7.4 ± 1.0 were assigned using Epik v5.0. The grids were generated (OPLS3 force field) by centering the grid box with a size of 20 Å on D3.32 (in case of 5-HT_6_R), and on W63 (for 5-HT_3_R). Flexible molecular docking was performed using Glide v8.5 at the standard precision (SP) level.

### In Vitro Pharmacological Evaluation

#### Cell Culture and Preparation of Cell Membranes for Radioligand Binding Assays

All of the experiments were carried out according to previously published procedures.^60−62^ In brief, HEK293 cells with stable expression of h5-HT_1A_, h5-HT_6_, h5-HT_7b_, and hD_2L_ receptors or CHO-K1 cells with a plasmid containing the sequence coding for the h5-HT_2A_R (PerkinElmer, # ES-313-C) were grown in Dulbecco’s modified Eagle medium containing 10% dialyzed fetal bovine serum and 500 μg/mL G418 sulfate. For membrane preparation, cells after reaching 90% confluence, were washed with phosphate-buffered saline (PBS), and pelleted by centrifugation (200 × g) in PBS containing 0.1 mM EDTA and 1 mM dithiothreitol.

#### Radioligand Binding Assays

The cell pellets were homogenized in assay buffer using a tissue homogenizer (Ultra Turrax IKAT25), centrifuged twice (35 000*g*, 15 min, 4 °C), and incubated (15 min, 37 °C) between centrifugation rounds. The buffers used were dedicated to a given type of receptor, and their composition was the same as in previously published articles.^60−62^ All assays were incubated in 96-well round-bottom microwell plates for 1 h at 37 °C. The exceptions were assays for 5-HT_1A_R and 5-HT_2A_R, which were performed at 24 °C and 27 °C, respectively. The total reaction volume was 200 μL. The incubation process was terminated by filtration through UniFilter-96 (PerkinElmer) plates with the FilterMate Universal Harvester (PerkinElmer, #C961962), and radioactivity retained on the filters was quantified on a MicroBeta counter for radiometric detection (PerkinElmer). For competitive studies, the assay samples contained as radioligands: 2.5 nM [^3^H]-8-OH-DPAT (PerkinElmer, #NET929001MC) for 5-HT_1A_R, 1 nM [^3^H]-ketanserin (PerkinElmer, #NET791250UC) for 5-HT_2A_R, 2 nM [^3^H]-LSD (PerkinElmer, # NET638250UC) for 5-HT_6_R, 0.8 nM [^3^H]-5-CT (PerkinElmer, #NET1188U100UC) for 5-HT_7_R, or 2.5 nM [^3^H]-raclopride (PerkinElmer, #NET975001MC) for D_2L_R. To evaluate the level of nonspecific signal 10 μM of 5-HT for 5-HT_1A_R and 5-HT_7_R, 20 μM of mianserin for 5-HT_2A_R, 10 μM of methiothepine for 5-HT_6_R and 10 μM of haloperidol for D_2L_R were used. Each compound was tested in triplicate at 7 concentrations in the range from 10^–10^ to 10^–4^ M. The inhibition constants (*K*_i_) were obtained from the Cheng–Prusoff model.^63^ The acquired results were presented as the mean of at least two independent experiments.

#### Evaluation of Functional Activity of 5-HT_6_Rs

Compounds were examined on 5-HT_6_R using their ability to inhibit cAMP production induced by 1 μM (EC_80_) 5-carboxamidotryptamine (5-CT). The level of cAMP was measured in 1321N1 cells expressing the *h*5-HT_6_R (PerkinElmer, #ES-316-CF). According to the manufacturer’s instructions, total cAMP was measured using the LANCE cAMP detection kit (PerkinElmer, #TRF0263). Cells were incubated with a mixture of compounds for 30 min at room temperature (RT) in a white polystyrene OptiPlate-384 (PerkinElmer, #6007299) microplate. After incubation, the reaction cells were lysed by the addition of 10 μL of cAMP detection buffer, including Eu-cAMP tracer and ULight-anti-cAMP working solution. The plate was incubated at RT for 1 h before measuring the signal with a Tecan multimode plate reader (Infinite M1000 Pro). Compounds were tested in triplicate at eight concentrations in the range from 10^–11^ to 10^–4^ M. *K*_b_ constants were calculated from Cheng–Prusoff equation^63^ adapted to functional assays.

#### Ex Vivo Evaluation of Functional Activity at 5-HT_3_R Functional Assay

Isolated guinea pig ileum was employed to test the affinity and the intrinsic activity of the investigated compounds for 5-HT_3_ receptors. The tissue was dissected from male guinea pigs previously deprived of food for 24 h but with free access to drinking water. A 15 cm ileum segment was excised from the small intestine of male guinea pigs and immersed into a Krebs solution (NaCl 120 mM, KCl 5.6 mM, MgCl_2_ 2.2 mM, CaCl_2_ 2.4 mM, NaHCO_3_ 19 mM, glucose 10 mM). After the first 5 cm length closest to the ileocaecal junction had been discarded 2 cm-long fragments were cut. Each segment of the ileum was placed in a 20 mL chamber of tissue organ bath system (Tissue Organ Bath System – 750 TOBS, DMT, Denmark) filled with the Krebs solution at 37° C, pH 7.4, with constant oxygenation (O_2_/CO_2_, 19:1), fixed by the lower end to a rod and by the upper end to the force–displacement transducer. The preparation was allowed to stabilize in organ baths for 60 min under a resting tension of 0.5 g, washing every 15 min with fresh Krebs solution. After the equilibration period, a cumulative concentration–response curve was constructed in each tissue for 5-HT (10 nM – 10 μM) by the method of van Rossum.^64^ The inhibitory effect of compounds was first evaluated by their influence (after 15 min of incubation with the tissue) on the contraction induced by single administration of 5-HT at the concentration of 3 μM and expressed as a percentage of inhibition of the maximal tension obtained with the contractile agent. Selected compounds were tested using an additional method. After establishment of the first 5-HT concentration–response curve, washing out of the tissue, and stabilization period, the same tissues were subsequently incubated with one of the concentrations of the tested compound for 15 min and the next cumulative concentration curve to 5-HT was obtained. Only one concentration of a studied compound was tested in each piece of tissue. Concentration–response curves were analyzed using GraphPad Prism 5.0 (GraphPad Software Inc., San Diego, CA) and the antagonistic properties were expressed as pD_2_′ or pA_2_. The Schild analysis was performed, and when the slope was not significantly different from unity, the pA_2_ value was determined (pA_2_—the negative log of molar concentration of the antagonist which reduces the effect of double dose of the agonist drug to that of a single dose). When the slope appeared to be significantly different from unity and the maximal response to 5-HT was not obtained, the pD_2_′ was calculated (pD_2_′—negative logarithm of the molar concentration of antagonist, which reduces the effect of an agonist to 50% of its maximum).

#### h5-HT_3_R Ion Channel Cell-Based Antagonist IonFlux Automated Patch Clamp Assay

The functional properties of the selected compound **FPPQ** on 5-HT_3_R were evaluated using an electrophysiological assay in CHO-K1 cells using IonFlux HT platform.

All recordings were obtained from a holding potential of -60 mV. To establish the baseline response, serotonin was added at the concentration corresponding to its EC_80_ value. Subsequently, the test compound was characterized in a dose–response protocol at the concentration ranges from 10^–6^ to 10^–11^ M, with 30 s preincubation, followed by the addition of 5-HT at its EC_80_ in the presence of the compound for 2 s.

Peak inward currents were measured in response to the serotonin additions in the presence of a single concentration of the compound. Obtained data have been normalized to the baseline peak current induced by the addition of EC_80_ serotonin for 2 s, according to eq 1
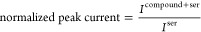
1Received data were analyzed using a four-parameter logistic equation in GraphPad Prism software. Experiment was performed Eurofins, France.

#### Determination of 5-HT_6_R Constitutive Activity at Gs Signaling

cAMP measurement was performed in NG108-15 cells transiently expressing 5-HT_6_R using the Bioluminescence Resonance Energy Transfer (BRET) sensor for cAMP, CAMYEL (cAMP sensor using YFP-Epac-RLuc).^65^ NG108-15 cells were co-transfected in suspension with 5-HT_6_R (or empty vector for Mock condition) and CAMYEL constructs, using Lipofectamine 2000, according to the manufacturer protocol, and plated in white 96-well plates (Greiner), at a density of 80 000 cells per well. Twenty-four hours after transfection, cells were washed with PBS containing calcium and magnesium. Coelanterazine H (Molecular Probes) was added at a final concentration of 5 μM, and left at room temperature for 5 min. BRET was measured using a Mithras LB 940 plate reader (Berthold Technologies).

#### Impact of Compounds on Neurite Growth

NG108-15 cells were grown in Dulbecco’s modified Eagle’s medium (DMEM) supplemented with 10% dialyzed fetal calf serum, 2% hypoxanthine/aminopterin/thymidine (Life Technologies), and antibiotics. Cells were transfected with plasmids encoding either cytosolic GFP or a GFP-tagged 5-HT_6_R in suspension using Lipofectamine 2000 (Life Technologies) and plated on glass coverslips. Six hours after transfection, cells were treated with either DMSO (control), **FPPQ**, or intepirdine (1 μM) for 24 h. Cells were fixed in 4% paraformaldehyde (PFA) supplemented with 4% sucrose for 10 min. PFA fluorescence was quenched by incubating the cells in PBS containing 0.1 M glycine, prior to mounting in ProLong Gold antifade reagent (Thermo Fisher Scientific). Cells were imaged using an AxioImager Z1 microscope equipped with epifluorescence (Zeiss), using a 20× objective for cultured cells, and neurite length (index of 5-HT_6_R constitutive activity as Cdk5 signaling) was assessed using the Neuron J plugin of the ImageJ software (NIH).

### Determination of Metabolic Stability in Rat and Human Liver Microsomes

Test compounds were prepared in phosphate-buffered saline (PBS) from 10 mM dimethyl sulfoxide (DMSO) solution so that the final incubation concentration was 1 μM. Pooled human (Invitrogen) or rat (Pharmidex Pharmaceutical Services Ltd) liver microsomes were diluted in PBS to allow for a 0.5 mg/mL total protein concentration in the assay. Incubations were started by adding NADPH (Sigma-Aldrich) solutions and were performed at 37 °C for various periods of time (0, 5, 15, 30, 45, 60, and 120 min). The reaction was stopped with the precipitation buffer (cold acetonitrile with 0.1% formic acid containing internal standard tolbutamide (Sigma-Aldrich), 400 μg/mL); this was used to precipitate proteins and release compound. The samples were vortexed and incubated for 10 min on ice and then centrifuged for 10 min at 15 000*g*. The resulting supernatants were transferred to vials and stored at −70 °C Supernatants were then analyzed by UHPLC-TOF MS. Assays were performed in triplicate in a total volume of 100 μL. Verapamil (Sigma-Aldrich) was used as a reference control.

#### Analysis

The samples were prepared for analysis by a fivefold dilution with 70/30 water/acetonitrile (25 μL of sample plus 100 μL of 70/30 water/acetonitrile). The samples were analyzed by high-resolution accurate mass UHPLC-TOF MS. The UPLC-MS system comprised an Agilent 1290 Infinity UHPLC pump with an Agilent 1290 Infinity HTS Autosampler, coupled with an Agilent 6550 iFunnel QToF mass spectrometer, equipped with a Waters Acquity BEH Phenyl UPLC column (50 × 2.1 mm^2^), 1.7 μm particle size. The system was controlled by MassHunter software vB.05.01. Gradient elution was employed with mobile phase components A and B being water/formic acid (0.1%, v/v) and acetonitrile/formic acid (0.1%, v/v), respectively. Initial conditions, from 0 to 0.3 min, were 2% B. Between 1.3 and 1.35 min %B was decreased to 2%, and this was maintained until the end of the run at 1.8 min. The flow rate was 0.4 mL/min, the injection volume was 5 μL, and the column was maintained at 50 °C. The mass spectrometer was operated in full scan mode, with positive ion electrospray data acquired over the *m*/*z* mass range 100–1000.

### Pharmacological and Safety Profile of FPPQ

The binding and safety profile of **FPPQ** was investigated using the SafetyScreen TM Panel (Eurofins) including enzymatic (*n* = 13) and binding assays (*n* = 74), (https://www.eurofinsdiscoveryservices.com/catalogmanagement/viewitem/SafetyScreen87-Panel/Panlabs/PP223#assayInfo). Additionally, we included the binding assays for the following receptors: serotonin 5-HT_4_ (ref (5)), 272000-HT_5A_ (ref (5)), 272100-HT_7_ (ref 272320), D_3_ (ref 219800), histamine H_3_ (ref 239820), sigma 1 (ref 278110), and enzymatic assays: CYP450, 1A2 (ref 2064), CYP450, 2C19 (ref 1772), CYP450, 2C9 (ref2066), CYP450, 2D6 (ref 1838), CYP450, 3A4 (ref 1769) to evaluate possible metabolic interactions.

### Determination of Agonist Effect of FPPQ for 5-HT_2B_ Receptors

Agonistic effect of compound **FPPQ** was determined as inhibition of 10^–6^ M serotonin using the HTRF technique. Experiment was performed in duplicate at Eurofins, France.

### Determination of Mutagenic Potential of FPPQ

Sodium azide (SA), 4-nitro-o-phenylenediamine (NPD, magnesium sulfate, sodium ammonium phosphate, D-glucose, D-biotin, sodium chloride, l-histidine HCl, l-tryptophane, dimethyl sulfoxide (DMSO), sodium phosphate-dibasic, citric acid monohydrate, potassium phosphate-dibasic, and sodium phosphate-monobasic were purchased from Sigma-Aldrich. Oxoid Nutrient Broth No. 2 (Oxoid Ltd.) and Agar-agar (Merck) were used as bacterial media.

#### Salmonella Mutagenic Assay

Mutagenic activity was tested by the Salmonella assay, using the *Salmonella Typhimurium* tester strains TA98, TA100, TA1535, and TA1537, kindly provided by Dr. T. Nohmi, Division of Genetics and Mutagenesis, National Institute of Hygienic Sciences, Tokyo, Japan, by the preincubation method. Selection of the strains was based on the testing and strain selection strategies of Mortelmans and Zeiger.^66^ The strains from frozen cultures were grown overnight for 12–14 h in Oxoid Nutrient Broth No. 2. Five different doses of test compounds were assayed. All of them were diluted in DMSO. The concentrations were selected on the basis of a preliminary toxicity test. The various concentrations of tested compounds were added to 300 μL of 0.2 M phosphate buffer (pH 7.4) and 60 μL of bacterial culture and then incubated at 37 °C for 20–30 min. After this time, 1200 μL of top agar was added to the mixture and poured on to a plate containing minimal agar. The plates were incubated at 37 °C for 48 h and the revertant colonies were counted manually. All experiments were analyzed in triplicate. Mutagenic activity is expressed as number of His+ induced revertants (mean ± standard deviation) for all tested doses. The standard mutagens used as positive controls were 4-nitro-o-phenylenediamine (0.25 μg/plate) for TA98 and TA1537, sodium azide (0.5 μg/plate) for TA100 and TA1535. DMSO served as the negative (solvent) control.

### Pharmacokinetic (PK) Profile of FPPQ

Lister Hooded (Envigo, U.K.) rats were administered with **FPPQ** (0.3, 1, and 3 mg/kg, *p.o*.) for assessment of serial plasma and terminal brain exposures for PK bio-analysis. Plasma levels were determined at 1, 2, 3, 4, 5, 8, 24, and 32 h after **FPPQ** administration (*n* = 3/time point). Blood samples were obtained by direct venipuncture from the tail and spun in a cooled centrifuge. Plasma aliquots (170 μL minimum) were stored frozen (-80 °C) until analysis. Brain levels were determined at 3, 4, 5, and 32 h after **FPPQ** administration. Brains were removed from the skull, briefly rinsed, hemisected, weighed, and stored frozen (-80 °C) until used. The samples were analyzed by UPLC-MS/MS (liquid chromatography-tandem mass spectrometry using electrospray ionization). The UPLC-MS/MS system comprised an Agilent 6410 triple quadrupole mass spectrometer coupled with an Agilent 1200 series UHPLC pump and autosampler. The system was controlled by MassHunter software vB.01.04. Sample analysis of **FPPQ** was carried out in positive ion electrospray mode for the following reaction monitoring transitions, precursor ion (*m*/*z*) = 411.1 and fragment ion (*m*/*z*) = 184.3. Chromatographic separations were achieved using a Kinetex C18 column (5 μm, 50 × 2.1 mm^2^, Phenomenex, U.K.) maintained at 50°C. The mobile phase consisted of water + 0.1% formic acid (solvent A) and acetonitrile + 0.1% formic acid (solvent B) programmed to linearly increase the proportion of solvent B as detailed: time after injection – 0 min (5% B), 0.3 (5% B), 1.9 min (95% B), 2.3 (95% B), 2.4 (5% B), 3.4 (5% B).

On the day of analysis, plasma samples were thawed and vortex mixed. Control plasma was spiked with SP14040 to create calibration standards. Aliquots (50 μL) of the samples were transferred to separate wells in a 96-well microtiter plate, to each of which was added 150 μL of IS solution. Blanks with no internal standard were prepared by the addition of either 150 μL of acetonitrile to 50 μL of control plasma. All samples were mixed on a rotary plate shaker (900 rpm, 20 min) and centrifuged (3000 rpm, 15 min). After centrifugation, aliquots (50 μL) of supernatant were transferred to separate wells in a 96-well microtiter plate containing water (100 μL) and mixed on a rotary plate shaker (450 rpm, 5 min) prior to analysis. On the day of analysis, brain samples were thawed, weighed and water added (1 mL/g tissue). Beads (zirconium oxide) were then added to the samples (1 g beads/g tissue) which were homogenized for 10 min at medium speed. Aliquots (50 μL) of homogenate brain samples were processed as described before for plasma aliquots.

### In Vivo Pharmacology

#### Animals

A total of 55, 45, 38, and 56 male Sprague–Dawley rats (Charles River, Germany) weighing 250–280 g on arrival were used in **FPPQ**, SB399885, SB399588+ondansetron, and CPPQ+ondansetron PCP hyperactivity studies, respectively. For the NOR test, 44 Sprague–Dawley rats were used. The animals were housed in a temperature-controlled (21 ± 1 °C) and humidity-controlled (40–50%) colony room under a 12/12 h light/dark cycle (lights on at 06:00 h). The rats were group-housed (5/cage) with free access to food and water. Rats were allowed to acclimatize for at least 7 days before the start of the experimental procedures. Behavioral testing was performed during the light phase of the light/dark cycle. The experiments were conducted in accordance with the European Guidelines for animal welfare (2010/63/EU) and were approved by the II Local Ethics Committee for Animal Experiments at the Maj Institute of Pharmacology, Polish Academy of Sciences, Krakow, Poland.

#### Spontaneous and PCP-Induced Hyperactivity

Both spontaneous and PCP-induced locomotor activity were measured automatically in Opto-Varimex-4 Auto-Tracks (Columbus Instruments, Ohio) located in sound-attenuated and ventilated boxes. The Auto-Track System sensed the motion with a grid of infrared photocells (16 beams per axis) surrounding the arena.

#### Drugs

Clozapine (Abcam Biochemicals, Cambridge, U.K.) was dissolved in 0.1 N HCl supplemented with distilled water to the appropriate volume (final pH = 5.0–6.0). PCP HCl (Sigma-Aldrich), ondansetron (Tocris, U.K.), SB399885 (Tocris, U.K.), **FPPQ**, and CPPQ ((*S*)-1-[(3-chlorophenyl)sulfonyl]-4-(pyrrolidine-3-yl-amino)-1*H*-pyrrolo[3,2-c]quinolone)^29,67^ were dissolved in distilled water. All compounds were administrated in a volume of 1 mL/kg.

#### Drugs Administration

Separate groups of animals were administered **FPPQ** (1 and 3 mg/kg), clozapine (1 and 3 mg/kg), or their vehicles PO, before being placed individually into the auto-tracks for 30 min of spontaneous locomotor activity measurement. In the other experiments, SB399885, ondansetron, CPPQ, or their combinations were administered IP 60 min before being placed in activity boxes. Following 30 min of spontaneous activity measurement, the animals were removed from the boxes, injected with phencyclidine hydrochloride (PCP) at a dose of 5 mg/kg (*SC)*, and then PCP-induced locomotor activity was measured for 120 min.

#### Data Analysis

The activity data collected every 5 min are presented as a raw readout as well as an Area Under Curve (AUC) of the distance traveled, in centimeters.

The measurements (30 min) preceding PCP administration indicate drug-induced effects on spontaneous locomotor activity. The second period (measured in the same animals at 0-120 min following PCP administration) indicates drug-induced alteration of PCP-induced hyperactivity.

Mixed-design two-way ANOVA with treatment(s) as between-subject factor and time as repeated measures factor on raw distance data, and separate one-way or two-way ANOVAs (on AUC data) followed by Dunnett’s multiple comparison, LSD or Tukey’s post hoc tests as well as analyses of contrast coefficients (Statistica 12 for Windows, IBM SPSS ver 26 for Mac) were used to assess the effects of compounds on activity. If not indicated otherwise, experimental design and drug doses were selected based on previous reports.^60^

#### Novel Object Recognition Test

Procedures were based on earlier studies by Popik et al.^52,68^ Rats were tested in a dimly lit (25 Lux) “open field” apparatus made of a dull gray plastic (66 × 56 × 30 cm^3^). After each measurement, the floor was cleaned and dried. The procedure lasting for 2 days consisted of the habituation to the test arena (without any objects) for 5 min. The test session comprising two trials separated by an intertrial interval (ITI) of 1 h was carried out on the next day. During the first trial (familiarization, T1) two identical objects (A1 and A2) were presented in the opposite corners of the open field, approximately 10 cm from the walls. During the second trial (recognition, T2), one of the A objects were replaced by a novel one so that the animals were presented with the A=familiar and B=novel objects. Both trials lasted for 3 min and the animals were returned to their home cages after T1. As the objects, the glass beakers filled with the gravel and the plastic bottles filled with the sand were used. The heights of the objects were comparable (∼12 cm) and the objects were heavy enough not to be displaced by the animals. The sequence of presentations and the location of the objects was randomly assigned to each rat. By definition, the animals explore the objects when looking, licking, sniffing, or touching the object while sniffing, but not leaning against, standing, or sitting on the object. Any rat exploring the two objects for less than 5 s within 3 min of T1 or T2 was eliminated from the study. Exploration time of the objects and the distance traveled were measured manually and using the Any-maze video tracking system, respectively. Based on exploration time (E) of two objects during T2, discrimination index (DI) was calculated according to the formula: DI = (EB–EA)/(EA+AB).

#### Experimental Design

Phencyclidine (PCP), used to attenuate learning, was administered at the dose of 5 mg/kg (IP) 45 min before familiarization phase (T1). The compounds were administrated *p.o*., 30 min before PCP (i.e., 1 h and 15 min before T1).

## Supporting Information Available

Experimental conditions for UPLC-MS, HRMS, and NMR analyses; ^1^H NMR spectra of intermediates; ^1^H NMR spectra and characterization of selected Boc-protected derivatives of final compounds; UPLC-MS, ^1^H NMR, and ^13^C NMR spectra of final compounds; synthesis and characterization of compound II; evaluation of binding mode of derivatives with different basic moieties against 5-HT_3_ and 5-HT_6_ receptors; off-target selectivity of **FPPQ**; and mutagenicity risk assessment for **FPPQ** (PDF)
5-HT_3_R crystal structure (PDB ID: 6NP0) (PDB)
5-HT_6_R homology model on the adrenergic β-2 template coordinate file (PDB)
SMILE strings of all shown molecules and corresponding biological activity (CSV)

## Supplementary Material

jm1c00224_si_001.pdf

jm1c00224_si_002.pdb

jm1c00224_si_003.pdb

## Notes

Analysis conducted using www.swissadme.ch resulted in no detectable PAINS—patterns for pyrroloquinolines **5d**, **5e**, and all of their sulfonyl derivatives **6**–**28**.

## Abbreviations Used

AUC: area under curve
BRET: bioluminescence resonance energy transfer
CL_int_: intrinsic clearance
5-CT: 5-carboxytryptamine
AcOH: acetic acid
ANOVA: analysis of variance
BTPP: (*tert*-Butylimino)tris(pyrrolidino)phosphorane
Cdk5: cyclin-dependent kinase 5
DI: discrimination index
GABA: γ aminobutyric acid
GPCR: G-protein-coupled receptor
*h*ERG: human ether-a-go-go related gene
5-HT: 5-hydroxytryptamine
LGIC: ligand-gated ion channel
L–R complex: ligand–receptor complex
MED: minimal effective dose
MeOH: methanol
mTOR: mechanistic target of rapamycin
MW: microwave
NOR: novel object recognition
PCP: phencyclidine
SEM: standard error of the mean
TEA: triethylamine
TR-FRET: time-resolved fluorescence resonance energy transfer
