# Supplementary material for: Structure-Based Design and Optimization of FPPQ, a Dual-Acting 5-HT3 and 5-HT6 Receptor Antagonist with Antipsychotic and Procognitive Properties
Source: J Med Chem. 2021 Sep 1;64(18):13279–98. doi: 10.1021/acs.jmedchem.1c00224 (PMC8474115; doi:10.1021/acs.jmedchem.1c00224)
Supplement: Supplementary file 1 — jm1c00224_si_001.pdf [file jm1c00224_si_001.pdf]

Supporting Information

Structure-Based Design and Optimization of FPPQ, a Dual-Acting 5-HT_3 and 5-HT_6 Receptor Antagonist With Antipsychotic and Pro-cognitive Properties

Paweł Zajdel,[a,*] Katarzyna Grychowska,[a] Szczepan Mogilski,[a] Rafał Kurczab,[b] Grzegorz Satała,[b] Ryszard Bugno,[b] Tomasz Kos,[b] Joanna Gołębiowska,[b] Natalia Malikowska-Racia,[b] Agnieszka Nikiforuk,[b] Severine Chaumont-Dubel,[c] Xavier Bantreil,[d] Maciej Pawłowski,[a] Jean Martinez,[d] Gilles Subra,[d] Frédéric Lamaty,[d] Philippe Marin,[c] Andrzej J. Bojarski,[b] and Piotr Popik[b,*]

[a] *Faculty of Pharmacy Jagiellonian University Medical College, 9 Medyczna Str., 30-688 Kraków, Poland*

[b] *Maj Institute of Pharmacology, Polish Academy of Sciences, 12 Smętna Str., 31-343 Kraków, Poland*

[c] *Institut de Génomique Fonctionelle, Université de Montpellier, CNRS, INSERM, 34094 Montpellier, France*

[d] *IBMM, Université de Montpellier, CNRS, ENSCM, 34095 Montpellier, France*

*Corresponding authors:
pawel.zajdel@uj.edu.pl
nfpopik@cyf-kr.edu.pl

Table of contents

Experimental conditions for UPLC-MS, HRMS and NMR analysis.. 3

Characterization data for selected Boc-protected derivatives of final compounds **7'–11'** and **15'–28'**. 4

^{1}H NMR spectra of intermediates **5a**–**5f**. 9

^{1}H NMR spectra of Boc-protected derivatives of final compounds **7'**, **15'**, **17'**–**22'** and **24'–28'** 12

UPLC-MS, ^{1}H NMR and ^{13}C NMR spectra of final compounds **6**–**28**.................................. 19

Synthesis and characterization of compoud **II**... 65

Scheme S1. Synthetic pathway leading to compound **II** 65

Table S1. Evaluation of the matching of derivatives with different modifications of the basic fragments to the 5-HT_3 and 5-HT_6 receptor binding sites... 68

Table S2. The off-target selectivity of **FPPQ** for 87 primary molecular targets[a].................... 69

Table S3. Mutagenicity risk assessment for **FPPQ** 73

Experimental conditions for UPLC-MS, HRMS and NMR analysis

Chromatographic separations were carried out using the Acquity UPLC BEH (bridged ethyl hybrid) C18 column; 2.1 × 100 mm, and 1.7 μm particle size, equipped with Acquity UPLC BEH C18 Van Guard pre-column; 2.1 × 5 mm, and 1.7 μm particle size. The column was maintained at 40°C, and eluted under gradient conditions from 95% to 0% of eluent A over 10 min, at a flow rate of 0.3 mL min^{-1}. Eluent A: water/formic acid (0.1%, v/v); eluent B: acetonitrile/formic acid (0.1%, *v/v*). Chromatograms were recorded using Waters eλ PDA detector. Spectra were analyzed in 200–700 nm range with 1.2 nm resolution and sampling rate 20 points/s. The UPLC purity of all the final compounds was confirmed to be 95% or higher.

MS detection settings of Waters TQD mass spectrometer were as follows: source temperature 150 °C, desolvation temperature 350°C, desolvation gas flow rate 600 L h-1, cone gas flow 100 L h-1, capillary potential 3.00 kV, cone potential 40 V. Nitrogen was used for both nebulizing and drying gas. The data were obtained in a scan mode ranging from 50 to 2000 m/z in time 1.0 s intervals. Data acquisition software was MassLynx V 4.1 (Waters).

HRMS analyses were obtained using electrospray ionization (ESI) in the positive ion mode. Mass accuracy was within 2 ppm error in full-scan mode. The optimized MS parameters were the following: ion spray voltage 4 kV; capillary temperature 240 ºC, dry gas flow rate 4 l/min. High-purity nitrogen as the nebulizing gas was used. Samples of 50 µM concentration were prepared from tested compounds using an eluent of acetonitrile + water (80:20) + 1% HCOOH.

All ^{1}H and ^{13}C NMR experiments were performed using JOEL JNM-ECZR500 RS1 (ECZR version) at 300 and 126 MHz, respectively as well as Varian BB 200 spectrometer at 300 and 75 MHz. Chemical shifts are given in parts per million (pmm), coupling constants (*J*) are reported in Hertz (Hz) and the splitting patterns are designated as follows:bs (broad singlet), s (singlet), d (doublet), t (triplet), q (quartet), dd (doublet of doublets), m (multiplet).

Characterization data for selected Boc-protected derivatives of final compounds 7’–11’ and 15’–28’.

Tert-butyl-4-(1-(phenylsulfonyl)-1H-pyrrolo[3,2-c]quinolin-4-yl)piperazine-1-carboxylate (**7'**)

Pale oil, 78% yield, t_R = 6.81, $C_{26}H_{28}N_4O_4S$, MW 492.59. ^{1}H NMR (300 MHz, $CDCl_3$) δ (ppm) 1.49 (s, 9H), 3.57–3.68 (m, 8H), 6.83 (d, *J* = 3.6 Hz, 1H), 7.27–7.31 (m, 1H), 7.41 (s, 2H), 7.43–7.54 (m, 2H), 7.67–7.74 (m, 2H), 7.82 (d, *J* = 7.2 Hz, 1H), 7.93 (d, *J* = 3.9 Hz, 1H), 8.80 (dd, *J* = 8.5, *J* = 1.3 Hz, 1H). Monoisotopic Mass 492.18, $[M+H]^+$ 493.3.

Tert-butyl-(S)-3-((1-(phenylsulfonyl)-1H-pyrrolo[3,2-c]quinolin-4-yl)amino)pyrrolidine-1-carboxylate (**8**’)

Pale oil, 78% yield, t_R = 6.52, $C_{26}H_{28}N_4O_4S$, MW 492.59. ^{1}H NMR (300 MHz, $CDCl_3$) δ (ppm) 1.47 (s, 9H), 1.98–2.07 (m, 1H), 2.34 (dd, *J* = 13.0, *J* = 6.0 Hz, 1H), 3.25–3.62 (m, 4H), 3.86 (dd, *J* = 11.3, *J* = 6.2 Hz, 1H), 4.91 (bs, 1H), 6.72 (d, *J* = 3.8 Hz, 1H), 7.20–7.25 (m, 2H), 7.34 (d, *J* = 7.9 Hz, 1H), 7.44–7.49 (m, 2H), 7.59 (d, *J* = 8.0 Hz, 1H), 7.73 (t, *J* = 1.9 Hz, 1H), 7.78 (d, *J* = 8.2 Hz, 1H), 7.87 (d, *J* = 3.6 Hz, 1H), 8.72 (dd, *J* = 8.6, *J* = 0.9 Hz, 1H). Monoisotopic Mass 492.18, $[M+H]^+$ 493.2.

Tert-butyl-3-((1-(phenylsulfonyl)-1H-pyrrolo[3,2-c]quinolin-4-yl)amino)azetidine-1-carboxylate (**9’**)

Pale oil, 75% yield, t_R = 6.58, $C_{25}H_{25}N_4O_4S$, MW 478.57. ^{1}H NMR (300 MHz, $CDCl_3$) δ (ppm) 1.49 (s, 9H), 3.36 (m, 1H), 3.90–4.16 (m, 4H), 6.86–6.93 (m, 2H), 7.02–7.10 (m, 3H), 7.21–7.24 (m, 2H), 7.37–7.40 (m, 1H), 7.70–7.75 (m, 2H), 7.90–8.11 (m, 1H). Monoisotopic Mass: 478.17, $[M+H]^+$ 479.2.

Tert-butyl-methyl(1-(1-(phenylsulfonyl)-1H-pyrrolo[3,2-c]quinolin-4-yl)pyrrolidin-3-yl)carbamate (**10**’)

Pale oil, 75% yield, t_R = 6.58, $C_{27}H_{30}N_4O_4S$, MW 506.62. ^{1}H NMR (300 MHz, $CDCl_3$) δ (ppm) 1.49 (s, 9H), 2.00–2.30 (m, 3H), 3.41 (s, 3H), 4.11–4.21 (m, 4H), 6.87–6.94 (m, 2H), 7.03–7.10 (m, 3H), 7.22–7.25 (m, 2H), 7.37–7.42 (m, 1H), 7.69–7.76 (m, 2H), 7.90–8.11 (m, 1H). Monoisotopic Mass: 506.20, $[M+H]^+$ 506.3.

Tert-butyl-4-(1-(phenylsulfonyl)-1H-pyrrolo[3,2-c]quinolin-4-yl)-1,4-diazepane-1-carboxylate (**11'**)

Pale oil, 75% yield, t_R = 6.52, $C_{27}H_{30}N_4O_4S$, MW 506.62. ^{1}H NMR (300 MHz, $CDCl_3$) δ (ppm) 1.50 (s, 9H), 2.10–2.22 (m, 2H), 3.25–3.50 (m, 2H), 3.70–3.80 (m, 2H), 3.92–4.20 (m, 4H), 6.89–6.97 (m, 2H), 7.00–7.08 (m, 3H), 7.21–7.28 (m, 2H), 7.40–7.48 (m, 1H), 7.70–7.77 (m, 2H), 7.95–8.12 (m, 1H).Monoisotopic Mass 506.20, $[M+H]^+$ 507.2.

Tert-butyl-4-(1-((2-bromophenyl)sulfonyl)-1H-pyrrolo[3,2-c]quinolin-4-yl)piperazine-1-carboxylate (**15'**)

Pale oil, 60% yield, t_R = 7.18, $C_{26}H_{27}BrN_4O_4S$, MW 571.49. ^{1}H NMR (300 MHz, $CDCl_3$) δ (ppm) 1.50 (s, 9H), 3.56–3.71 (m, 8H), 6.82 (d, J = 3.9 Hz, 1H), 7.16 (s, 1H), 7.35–7.45 (m, 3H), 7.60–7.66 (m, 1H), 7.78–7.86 (m, 1H), 7.99 (d, J = 3.9 Hz, 2H), 8.40 (dd, J = 8.6, J = 0.9 Hz, 1H). Monoisotopic Mass 570.09, $[M+H]^+$ 571.2, 573.2.

Tert-butyl-4-(1-((2-chlorophenyl)sulfonyl)-1H-pyrrolo[3,2-c]quinolin-4-yl)piperazine-1-carboxylate (**16'**)

Pale oil, 55% yield, t_R = 7.13, $C_{26}H_{27}ClN_4O_4S$, MW 527.04. ^{1}H NMR (300 MHz, $CDCl_3$) δ (ppm) 1.50 (s, 9H), 3.6–3.70 (m, 8H), 6.81 (d, J = 3.9 Hz, 1H), 7.15 (s, 1H), 7.33–7.42 (m, 3H), 7.61–7.64 (m, 1H), 7.77–7.86 (m, 1H), 8.01 (d, J = 3.9 Hz, 2H), 8.40 (dd, J = 8.6, J = 0.9 Hz, 1H). Monoisotopic Mass 526.14, $[M+H]^+$ 527.2.

Tert-butyl-4-(1-((3-fluorophenyl)sulfonyl)-1H-pyrrolo[3,2-c]quinolin-4-yl)piperazine-1-carboxylate (**17'**)

Pale oil, 93% yield, t_R = 7.24, $C_{26}H_{27}FN_4O_4S$, MW 510.58. ^{1}H NMR (300 MHz, $CDCl_3$) δ (ppm) 1.49 (s, 9H), 3.57–3.68 (m, 8H), 6.85 (d, J = 3.9 Hz, 1H), 7.16–7.24 (m, 1H), 7.28–7.34 (m, 1H), 7.35–7.43 (m, 2H), 7.46–7.53 (m, 2H), 7.83 (s, 1H), 7.89 (d, J = 3.9 Hz, 1H), 8.77 (dd, J = 8.6, J = 0.9 Hz, 1H). Monoisotopic Mass 510.17, $[M+H]^+$ 511.4.

Tert-butyl-4-(1-((3-chlorophenyl)sulfonyl)-1H-pyrrolo[3,2-c]quinolin-4-yl)piperazine-1-carboxylate ((**18'**)

Pale oil, 93% yield, t_R = 7.86, $C_{26}H_{27}ClN_4O_4S$, MW 527.03. ^{1}H NMR (300 MHz, $CDCl_3$) δ (ppm) 1.50 (s, 9H), 3.48–3.72 (m, 8H), 6.85 (d, J = 3.6 Hz, 1H), 7.28–7.32 (m, 2H), 7.43–7.50 (m, 2H), 7.51–7.53 (m, 1H), 7.72 (s, 1H), 7.80–7.82 (m, 1H), 7.89 (d, J = 3.9 Hz, 1H), 8.73–8.82 (m, 1H). Monoisotopic Mass 526.14, $[M+H]^+$ 527.0.

Tert-butyl-4-(1-((3-(trifluoromethyl)phenyl)sulfonyl)-1H-pyrrolo[3,2-c]quinolin-4-yl)piperazine-1-carboxylate (**19'**)

Pale oil, 83% yield, t_R = 7.68, $C_{27}H_{27}F_3N_4O_4S$, MW 560.59. ^{1}H NMR (300 MHz, $CDCl_3$) 1.49 (s, 9H), 3.54–3.72 (m, 8H), 6.86 (d, *J* = 3.9 Hz, 1H), 7.27–7.36 (m, 1H), 7.42–7.54 (m, 2H), 7.69–7.85 (m, 3H), 7.91 (d, *J* = 3.9 Hz, 1H), 8.09 (s, 1H), 8.79 (dd, *J* = 8.5, *J* = 1.3 Hz, 1H). Monoisotopic Mass 560.17, $[M+H]^+$ 561.1.

Tert-butyl-4-(1-(3-methylphenylsulfonyl)-1H-pyrrolo[3,2-c]quinolin-4-yl)piperazine-1-carboxylate (**20'**)

Pale oil, 83% yield, t_R = 7.68, $C_{27}H_{30}N_4O_4S$, MW 506.62. ^{1}H NMR (300 MHz, DMSO-d_6) δ ppm 1.49 (s, 9H), 2.30 (s, 3H), 3.50–3.64 (m, 8H), 7.04–7.22 (m, 1H), 7.25–7.30 (m, 1H), 7.39–7.54 (m, 3H), 7.66–7.68 (m, 1H), 7.70–7.77 (m, 2H), 8.01–8.14 (m, 1H), 8.64–8.69 (m, 1H) Monoisotopic Mass 506.20, $[M+H]^+$ 507.2.

Tert-butyl-4-(1-((3-methoxyphenyl)sulfonyl)-1H-pyrrolo[3,2-c]quinolin-4-yl)piperazine-1-carboxylate (**21'**)

Pale oil, 91% yield, t_R = 7.10, $C_{27}H_{30}N_4O_5S$, MW 522.62. ^{1}H NMR (300 MHz, $CDCl_3$) δ (ppm) 1.49 (s, 9H), 3.58–3.65 (m, 8H), 3.67 (s, 3H), 6.82 (d, *J* = 3.6 Hz, 1H), 6.99 (s, 1H), 7.21–7.24 (m, 1H), 7.25–7.29 (m, 2H), 7.30–7.34 (m, 1H), 7.45–7.52 (m, 1H), 7.81–7.86 (m, 1H), 7.90 (d, *J* = 3.9 Hz, 1H), 8.89 (dd, *J* = 8.5, *J* = 1.3 Hz, 1H). Monoisotopic Mass 522.19, $[M+H]^+$ 523.2.

Tert-butyl-4-(1-((4-fluorophenyl)sulfonyl)-1H-pyrrolo[3,2-c]quinolin-4-yl)piperazine-1-carboxylate (**22'**)

Pale oil, 95% yield, t_R = 7.08, $C_{26}H_{27}FN_4O_4S$, MW 510.58. ^{1}H NMR (300 MHz, $CDCl_3$) δ (ppm) 1.48 (s, 9H), 3.55–3.66 (m, 8H), 6.83 (d, *J* = 3.6 Hz, 1H), 6.99–7.07 (m, 2H), 7.25–7.32 (m, 1H), 7.43–7.53 (m, 1H), 7.69–7.77 (m, 2H), 7.80–7.82 (m, 1H), 7.90 (d, *J* = 3.9 Hz, 1H), 8.81 (dd, *J* = 8.6, *J* = 0.9 Hz, 1H). Monoisotopic Mass 510.17, $[M+H]^+$ 511.3.

Tert-butyl-4-(1-((4-(trifluoromethyl)phenyl)sulfonyl)-1H-pyrrolo[3,2-c]quinolin-4-yl)piperazine-1-carboxylate (**23’**)

Pale oil, 82% yield, t_R = 7.68, $C_{27}H_{27}F_3N_4O_4S$, MW 560.59. ^{1}H NMR (300 MHz, $CDCl_3$) 1.44 (s, 9H), 3.48–3.53 (m, 1H), 3.52 (bs, 1H), 3.50–3.61 (m, 1H), 3.57 (s, 5H), 7.17 (d, *J* = 3.9 Hz,

1H), 7.31–7.35 (m, 1H), 7.44–7.54 (m, 3H), 7.57–7.64 (m, 1H), 7.71–7.80 (m, 2H), 8.07 (d, *J* = 3.9 Hz, 1H), 8.66 (dd, *J* = 8.5, *J* = 0.9 Hz, 1H) Monoisotopic Mass 560.17, $[M+H]^+$ 561.1.

Tert-butyl-4-(1-((4-isopropylphenyl)sulfonyl)-1H-pyrrolo[3,2-c]quinolin-4-yl)piperazine-1-carboxylate (**24'**)

Pale oil, 79% yield, t_R = 8.05, $C_{29}H_{34}N_4O_4S$, MW 534.67. ^1H NMR (300 MHz, $CDCl_3$) δ (ppm) 1.15 (d, *J* = 6.9 Hz, 6H), 1.49 (s, 9H), 3.55–3.67 (m, 8H), 6.82 (d, *J* = 3.9 Hz, 1H), 7.18–7.24 (m, 2H), 7.31 (dd, *J* = 8.6, *J* = 1.4 Hz, 1H), 7.48 (d, *J* = 1.3 Hz, 1H), 7.61–7.68 (m, 2H), 7.80–7.83 (m, 1H), 7.92 (d, *J* = 3.6 Hz, 1H), 8.85 (dd, *J* = 8.5, *J* = 1.3 Hz, 1H). Monoisotopic Mass 534.23, $[M+H]^+$ 535.4.

Tert-butyl-4-(1-((3,4-difluorophenyl)sulfonyl)-1H-pyrrolo[3,2-c]quinolin-4-yl)piperazine-1-carboxylate (**25'**)

Pale oil, 80% yield, t_R = 8.60, $C_{26}H_{26}F_2N_4O_4S$, MW 528.57. ^1H NMR (300 MHz, $CDCl_3$) δ (ppm) 1.49 (s, 9H), 3.54–3.72 (m, 8H), 6.86 (d, *J* = 3.6 Hz, 1H), 7.30–7.38 (m, 1H), 7.39–7.45 (m, 1H), 7.46–7.56 (m, 3H), 7.78–7.90 (m, 2H), 8.70–8.74 (m, 1H). Monoisotopic Mass 528.16, $[M+H]^+$ 529.2.

Tert-butyl-4-(1-((3,4-dichlorophenyl)sulfonyl)-1H-pyrrolo[3,2-c]quinolin-4-yl)piperazine-1-carboxylate (**26'**)

Pale oil, 80% yield, t_R = 8.60, $C_{26}H_{26}Cl_2N_4O_4S$, MW 561.48. ^1H NMR (300 MHz, $CDCl_3$) δ (ppm) 1.47 (s, 9H), 3.56–3.70 (m, 8H), 6.86 (d, *J* = 3.6 Hz, 1H), 7.30–7.38 (m, 1H), 7.39–7.45 (m, 1H), 7.46–7.56 (m, 2H), 7.78–7.90 (m, 3H), 8.79 (dd, *J* = 8.5, *J* = 1.3 Hz, 1H). Monoisotopic Mass 560.11, $[M+H]^+$ 561.2, 563.2.

Tert-butyl-4-(1-((2,5-difluorophenyl)sulfonyl)-1H-pyrrolo[3,2-c]quinolin-4-yl)piperazine-1-carboxylate (**27'**)

Pale oil, 81% yield, t_R = 4.75, $C_{26}H_{26}F_2N_4O_2S$, MW 528.57. ^1H NMR (300 MHz, $CDCl_3$/methanol-d_4) δ (ppm) 1.50 (m, 9H), 3.51–3.60 (m, 8H), 6.75–6.78 (m, 1H), 6.92–7.08 (m, 1H), 7.24–7.26 (m, 1H), 7.27–7.35 (m, 1H), 7.49–7.52 (m, 1H), 7.75-7.84 (m, 2H), 7.90–7.97 (m, 1H), 8.62 (d, *J* = 8.2 Hz, 1H). Monoisotopic Mass 528.16, $[M+H]^+$ 529.2.

Tert-butyl-4-(1-(naphthalen-1-ylsulfonyl)-1H-pyrrolo[3,2-c]quinolin-4-yl)piperazine-1-carboxylate (**28'**)

Pale oil, 86% yield, t_R = 7.49, $C_{30}H_{30}N_4O_4S$, MW 542.65. ^{1}H NMR (300 MHz, $CDCl_3$) δ (ppm) 1.50 (s, 9H), 3.59–3.70 (m, 8H), 6.88 (d, *J*=3.85 Hz, 1H), 7.07–7.14 (m, 1H), 7.30–7.37 (m, 1H), 7.38–7.44 (m, 1H), 7.58–7.71 (m, 2H), 7.73–7.84 (m, 2H), 7.87–7.93 (m, 1H), 7.98–8.04 (m, 2H), 8.50 (dd, *J* = 8.6, *J* = 0.9 Hz, 1H), 8.58 (d, *J* = 8.5 Hz, 1H). Monoisotopic Mass 542.20, $[M+H]^+$ 543.4.

^{1}H NMR spectra of intermediates 5a–5f.

Tert-butyl-(S)-3-((1H-pyrrolo[3,2-c]quinolin-4-yl)amino)pyrrolidine-1-carboxylate (**5a**)

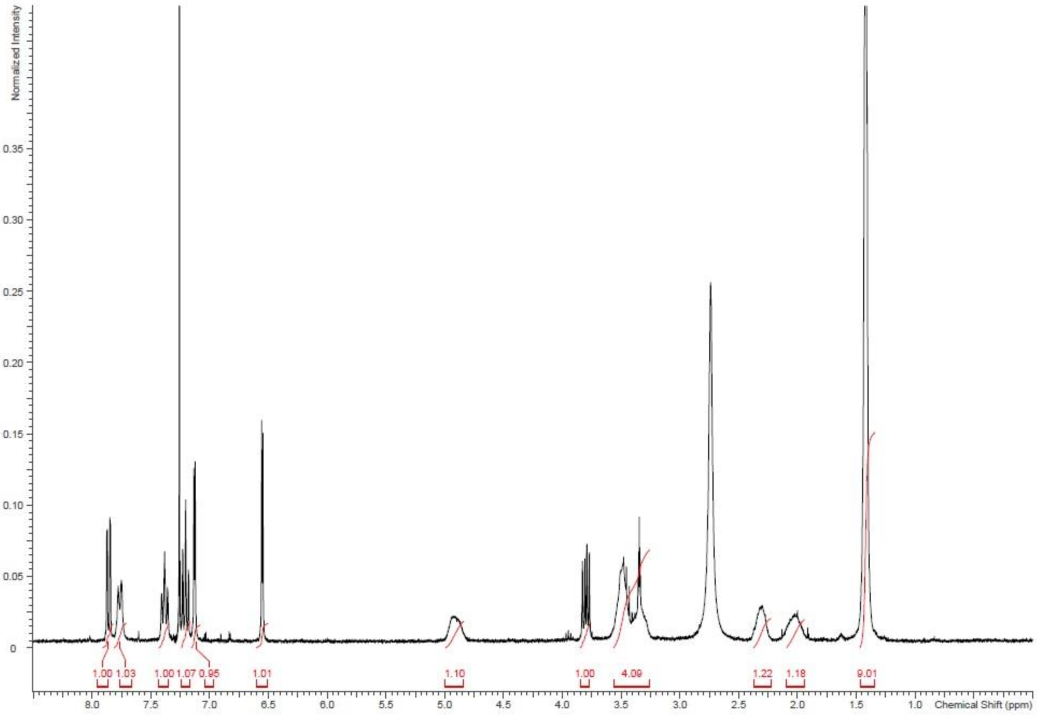

Tert-butyl-3-((1H-pyrrolo[3,2-c]quinolin-4-yl)amino)azetidine-1-carboxylate (**5b**)

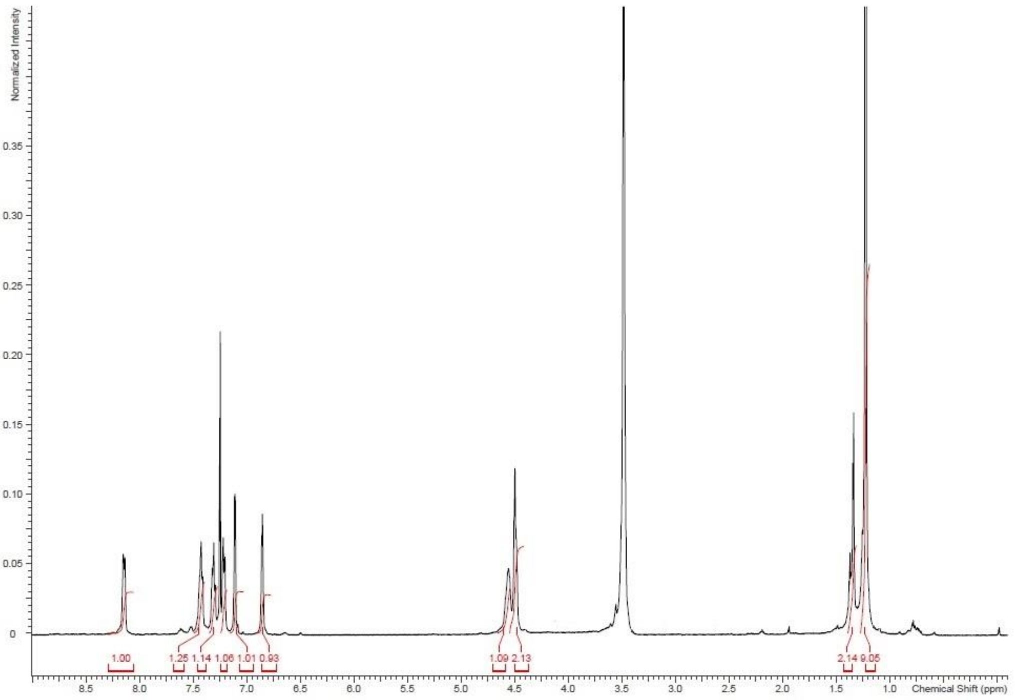

Tert-butyl (1-(1H-pyrrolo[3,2-c]quinolin-4-yl)pyrrolidin-3-yl)(methyl)carbamate (**5c**)

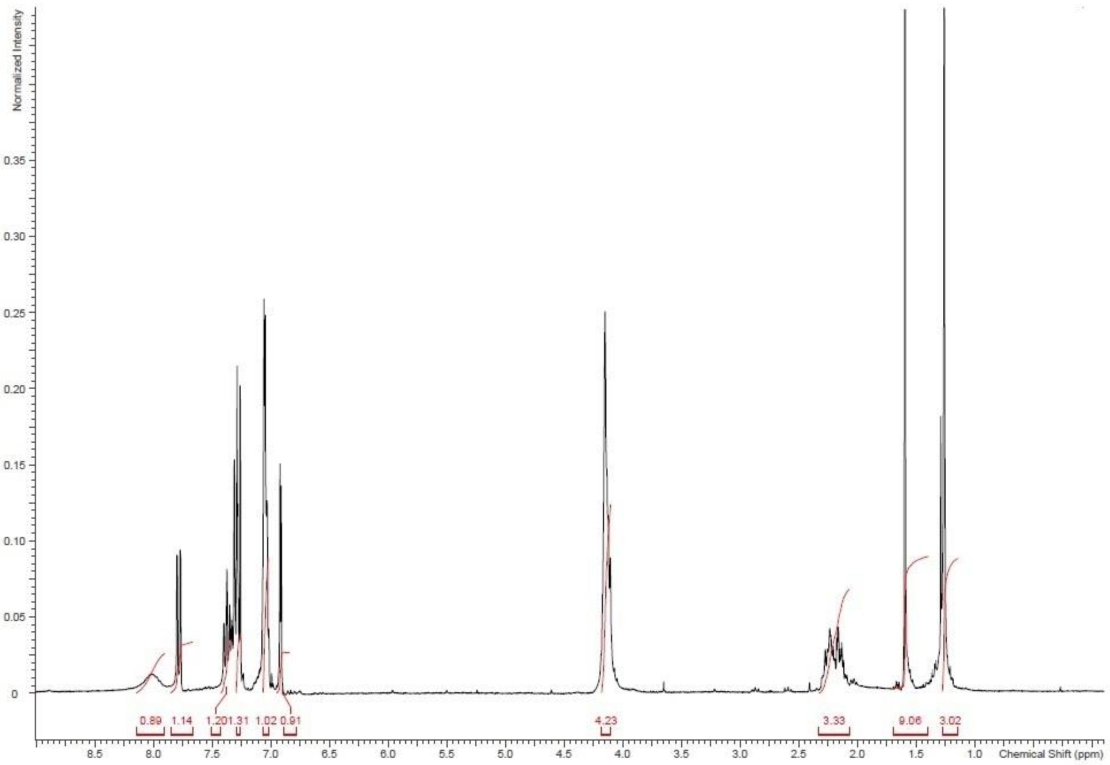

4-(4-Methylpiperazin-1-yl)-1H-pyrrolo[3,2-c]quinoline (**5d**)

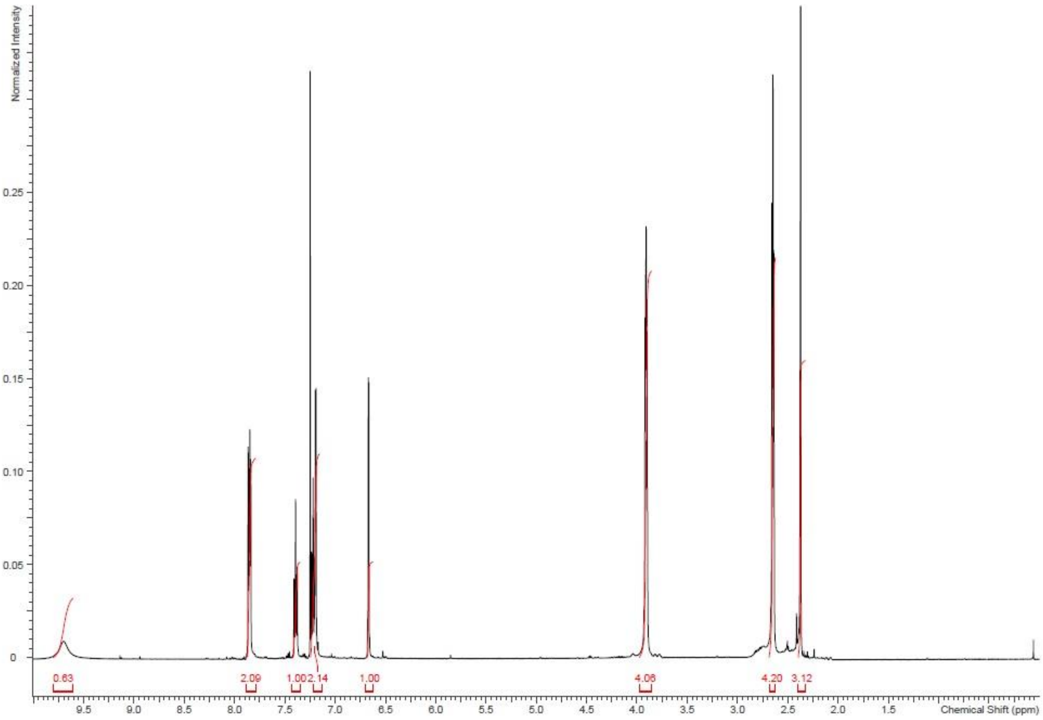

Tert-butyl 4-(1H-pyrrolo[3,2-c]quinolin-4-yl)piperazine-1-carboxylate (**5e**)

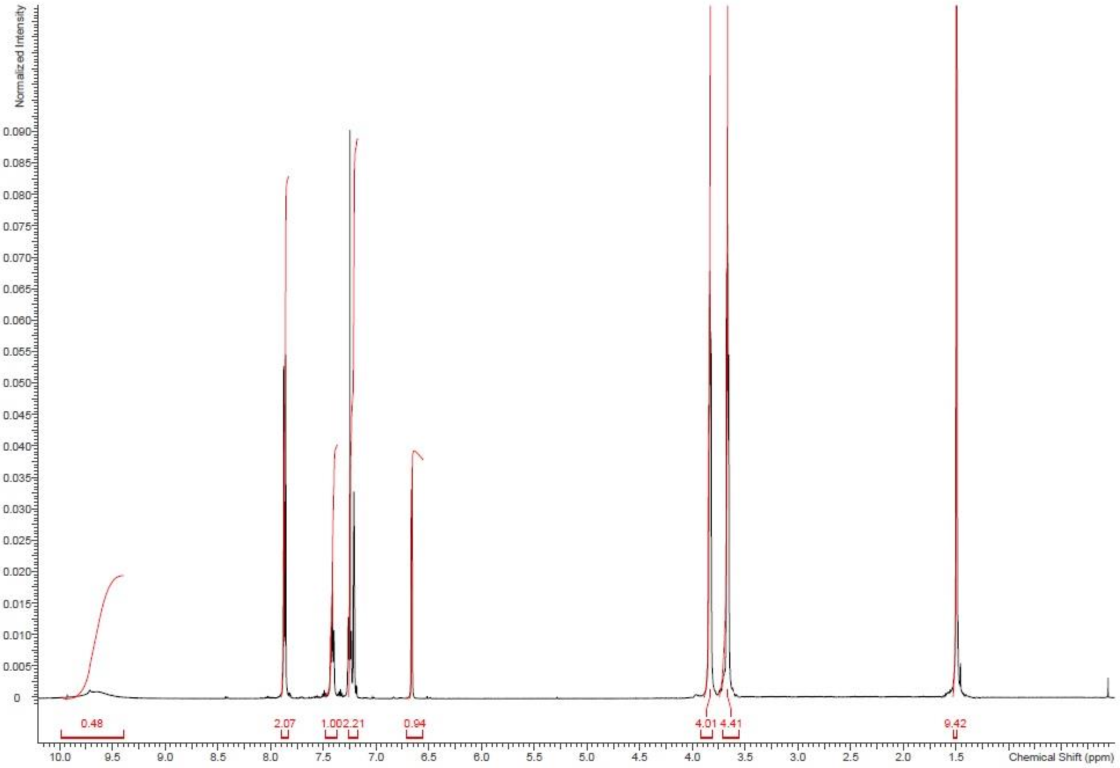

Tert-butyl-4-(1H-pyrrolo[3,2-c]quinolin-4-yl)-1,4-diazepane-1-carboxylate (**5f**)

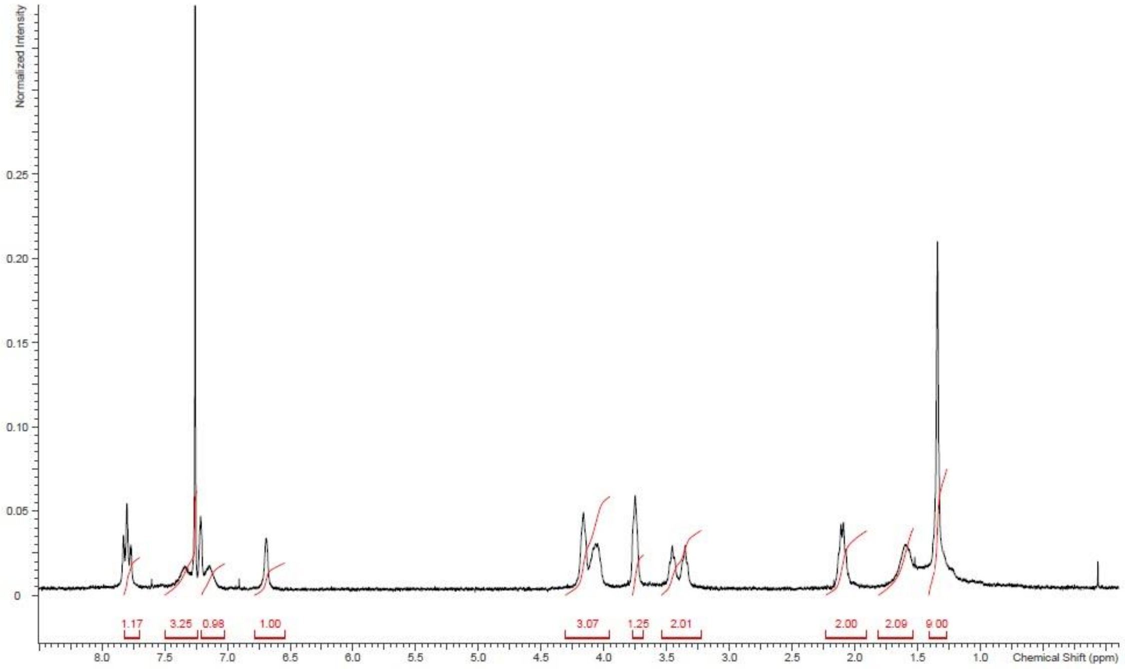

^{1}H NMR spectra of Boc-protected derivatives of final compounds 7’, 15’, 17’–22’ and 24’–28’

Tert-butyl-4-(1-(phenylsulfonyl)-1H-pyrrolo[3,2-c]quinolin-4-yl)piperazine-1-carboxylate (**7'**)

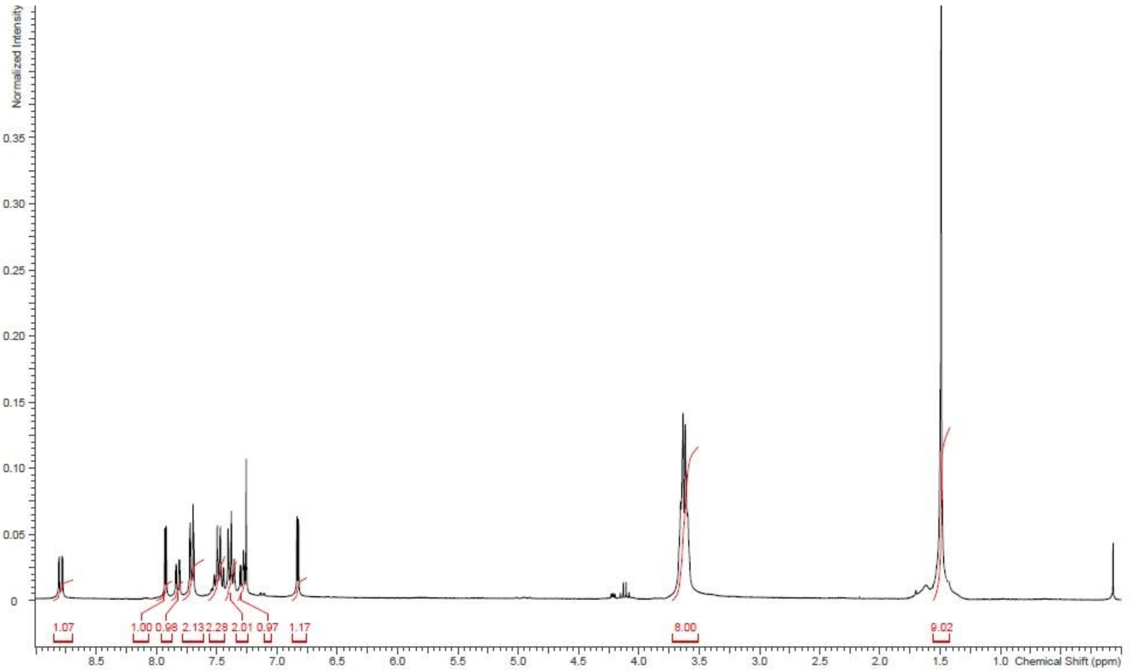

Tert-butyl-4-(1-((2-bromophenyl)sulfonyl)-1H-pyrrolo[3,2-c]quinolin-4-yl)piperazine-1-carboxylate (**15'**)

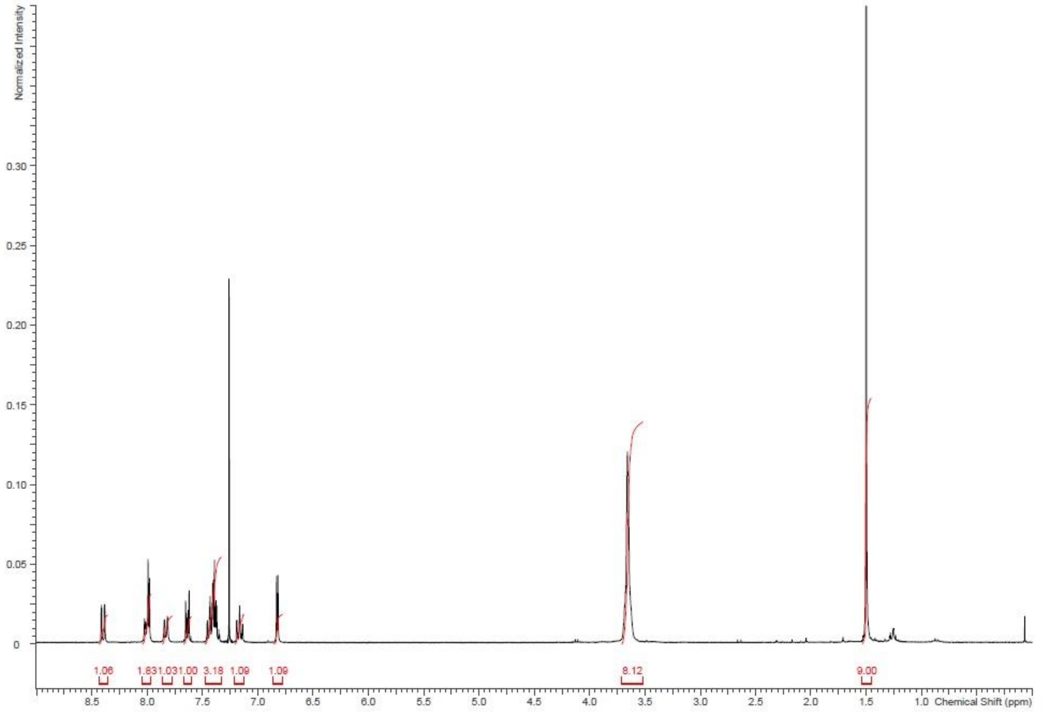

Tert-butyl-4-(1-((3-fluorophenyl)sulfonyl)-1H-pyrrolo[3,2-c]quinolin-4-yl)piperazine-1-carboxylate (**17'**)

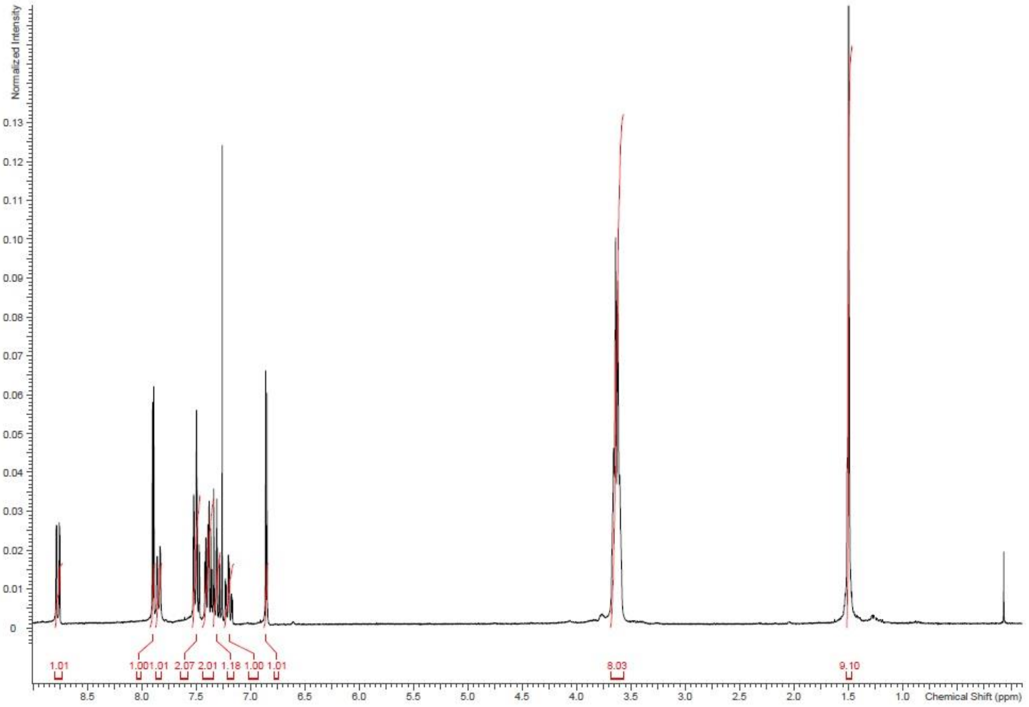

Tert-butyl-4-(1-((3-chlorophenyl)sulfonyl)-1H-pyrrolo[3,2-c]quinolin-4-yl)piperazine-1-carboxylate (**18'**)

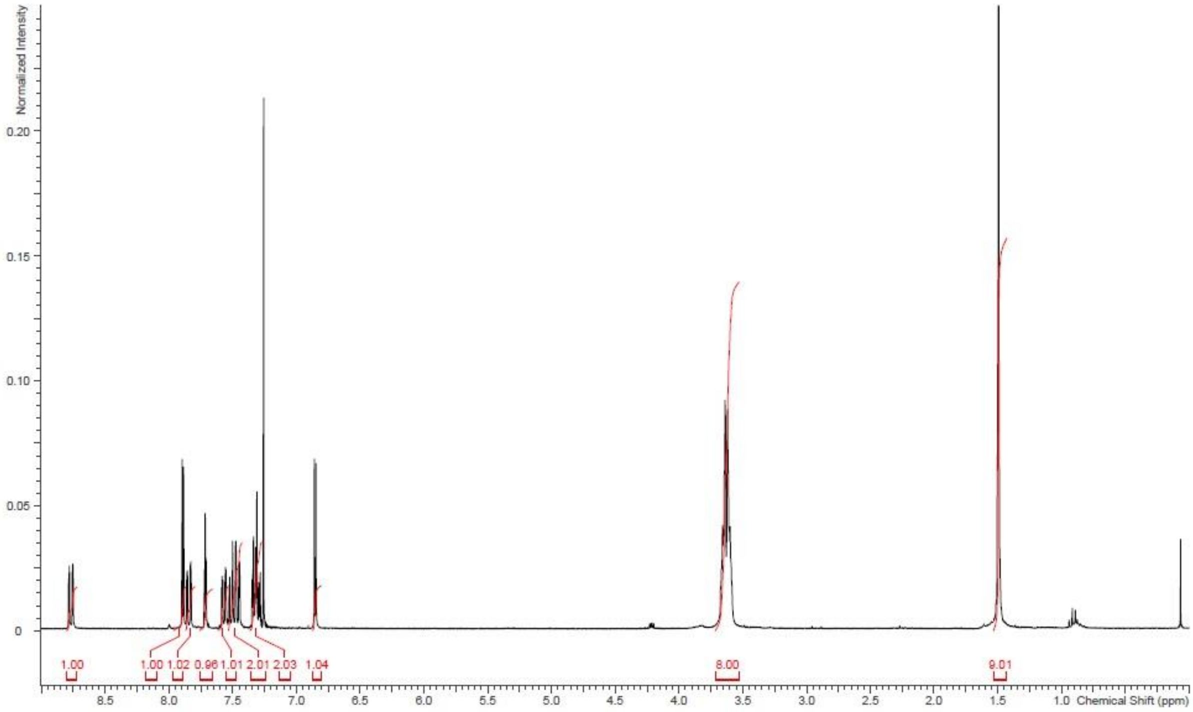

Tert-butyl-4-(1-((3-(trifluoromethyl)phenyl)sulfonyl)-1H-pyrrolo[3,2-c]quinolin-4-yl)piperazine-1-carboxylate (**19'**)

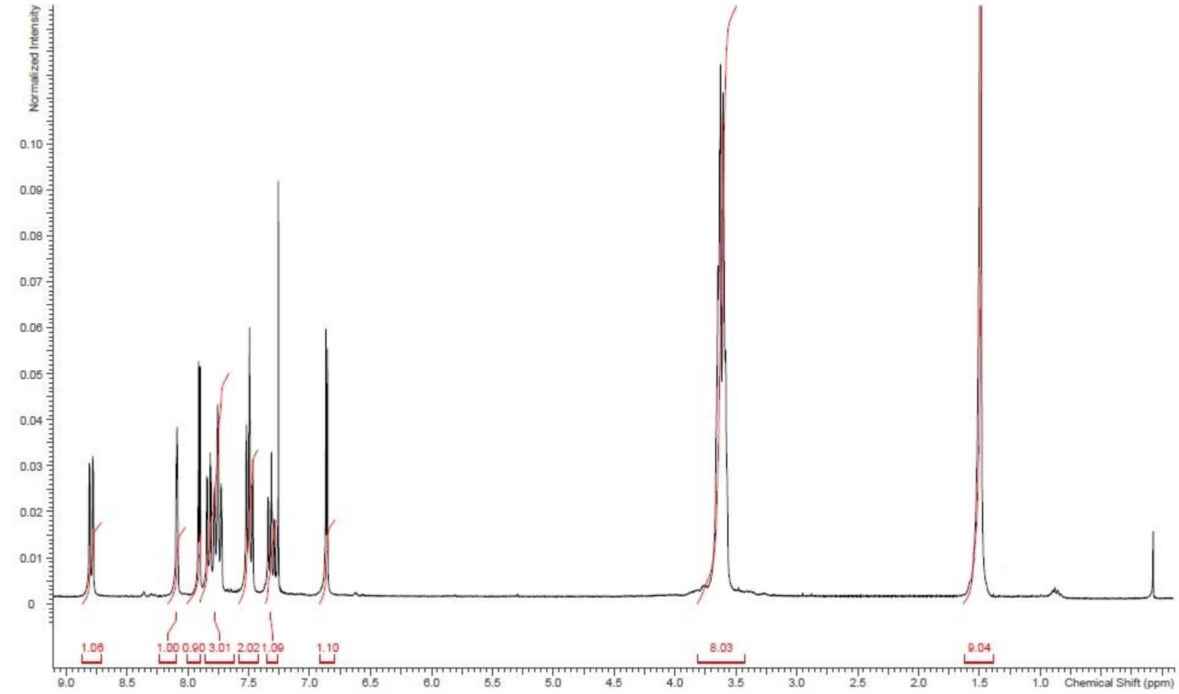

Tert-butyl-4-(1-(3-methylphenylsulfonyl)-1H-pyrrolo[3,2-c]quinolin-4-yl)piperazine-1-carboxylate (**20'**)

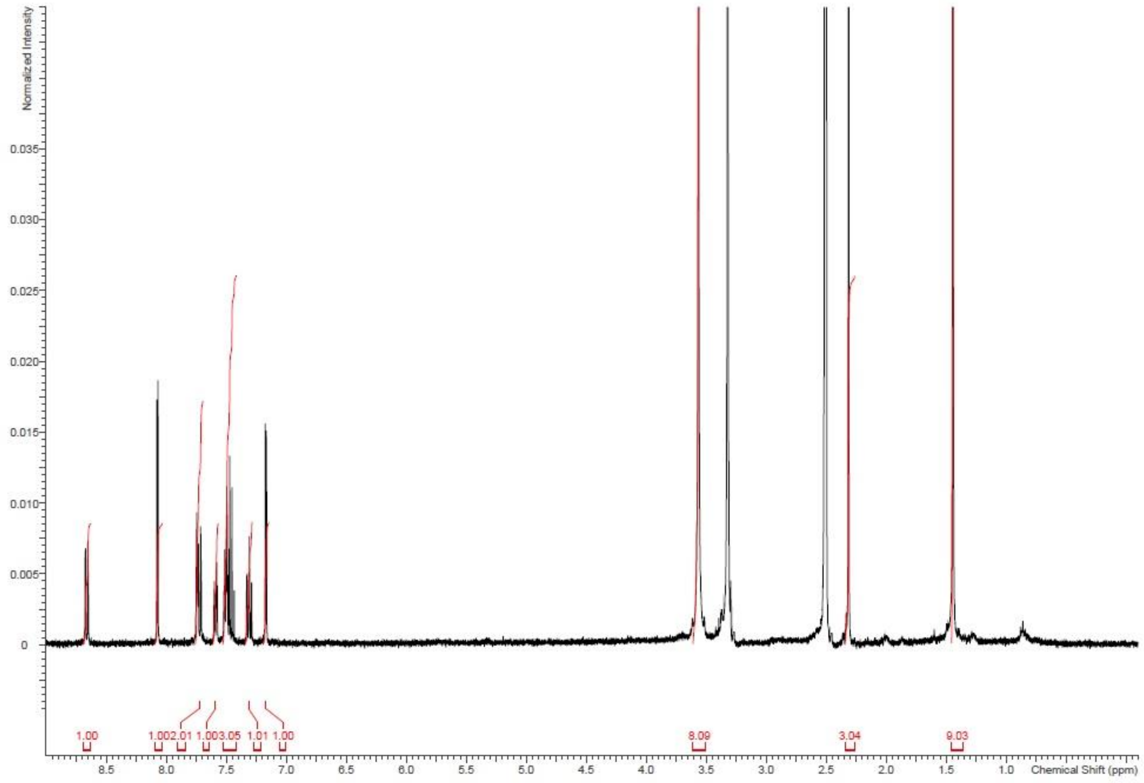

Tert-butyl-4-(1-((3-methoxyphenyl)sulfonyl)-1H-pyrrolo[3,2-c]quinolin-4-yl)piperazine-1-carboxylate (**21'**)

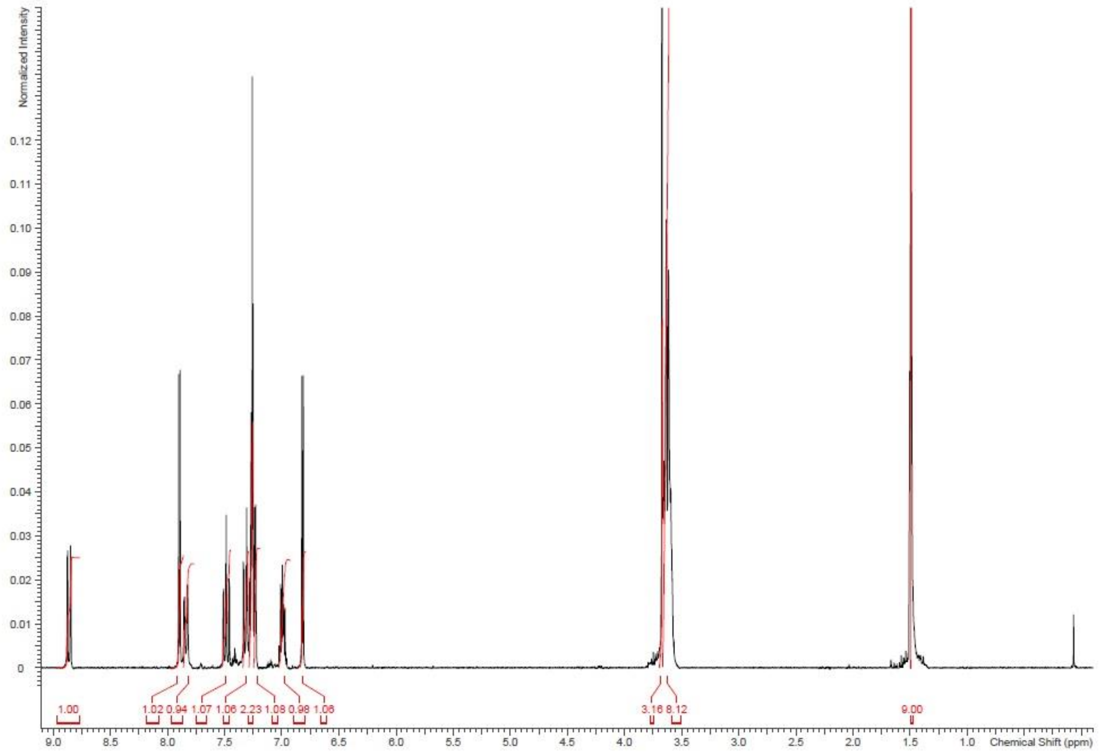

Tert-butyl-4-(1-((4-fluorophenyl)sulfonyl)-1H-pyrrolo[3,2-c]quinolin-4-yl)piperazine-1-carboxylate (**22'**)

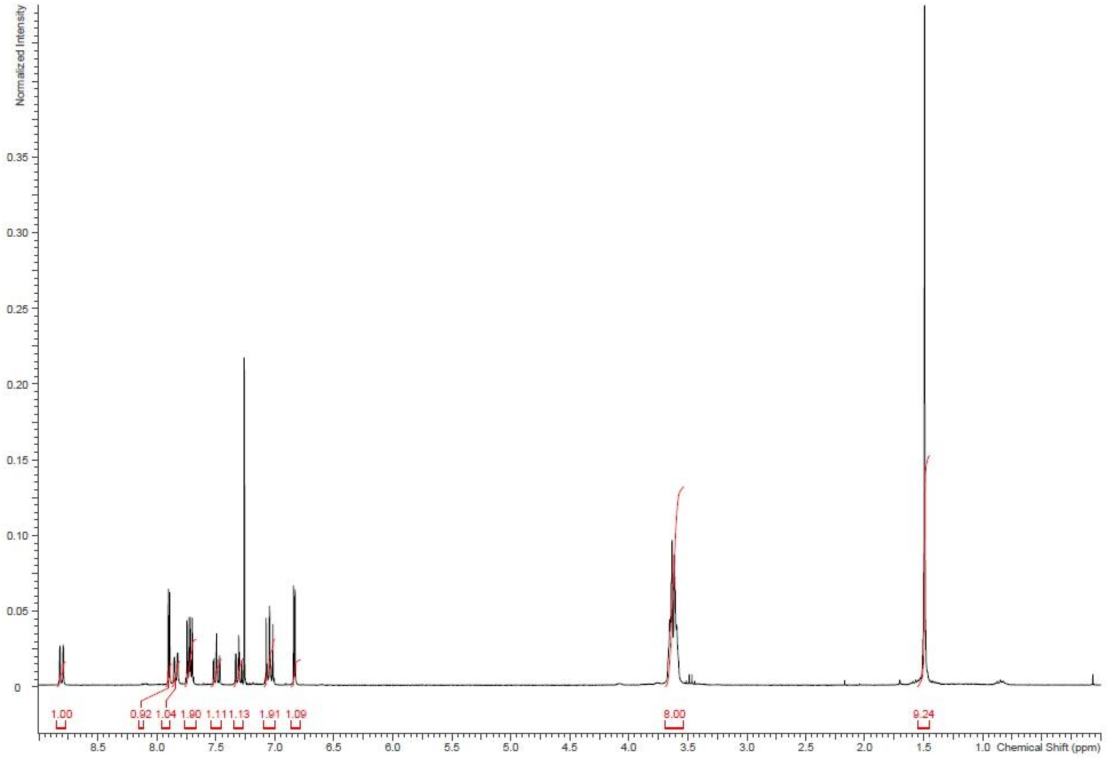

Tert-butyl-4-(1-((4-isopropylphenyl)sulfonyl)-1H-pyrrolo[3,2-c]quinolin-4-yl)piperazine-1-carboxylate (**24'**)

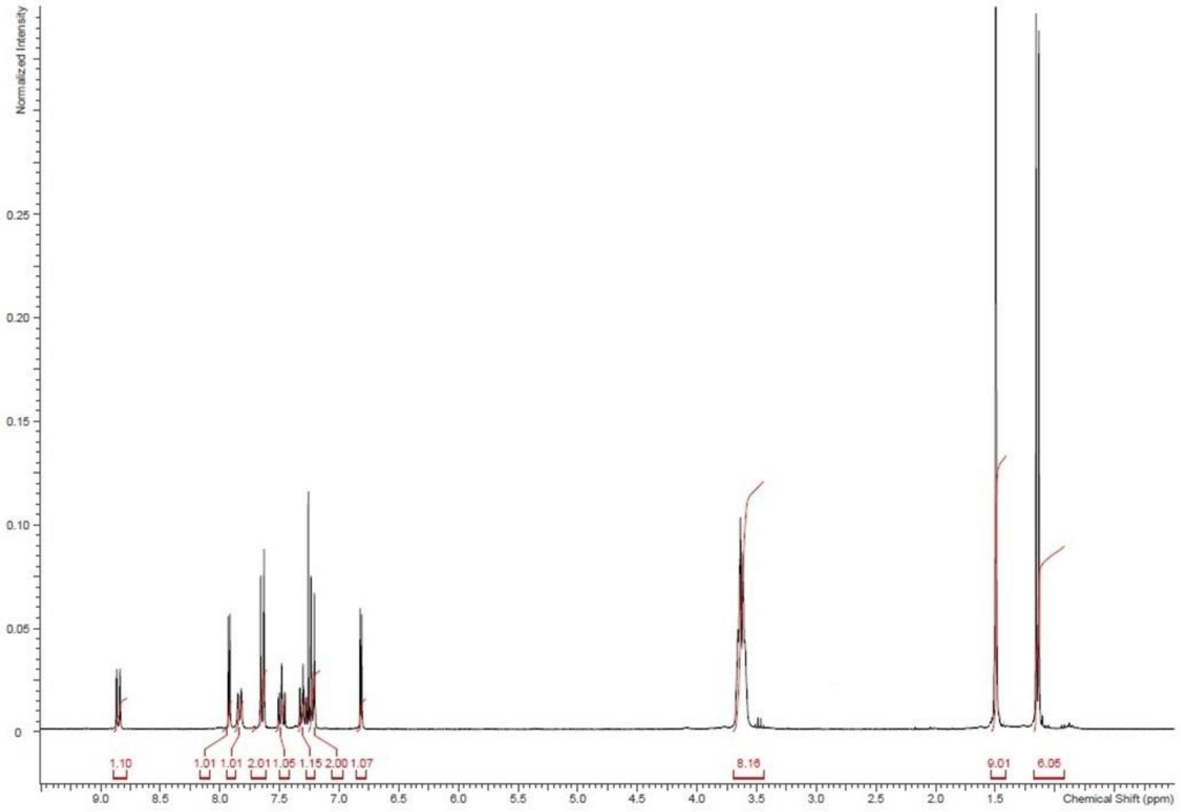

Tert-butyl-4-(1-((3,4-difluorophenyl)sulfonyl)-1H-pyrrolo[3,2-c]quinolin-4-yl)piperazine-1-carboxylate (**25'**)

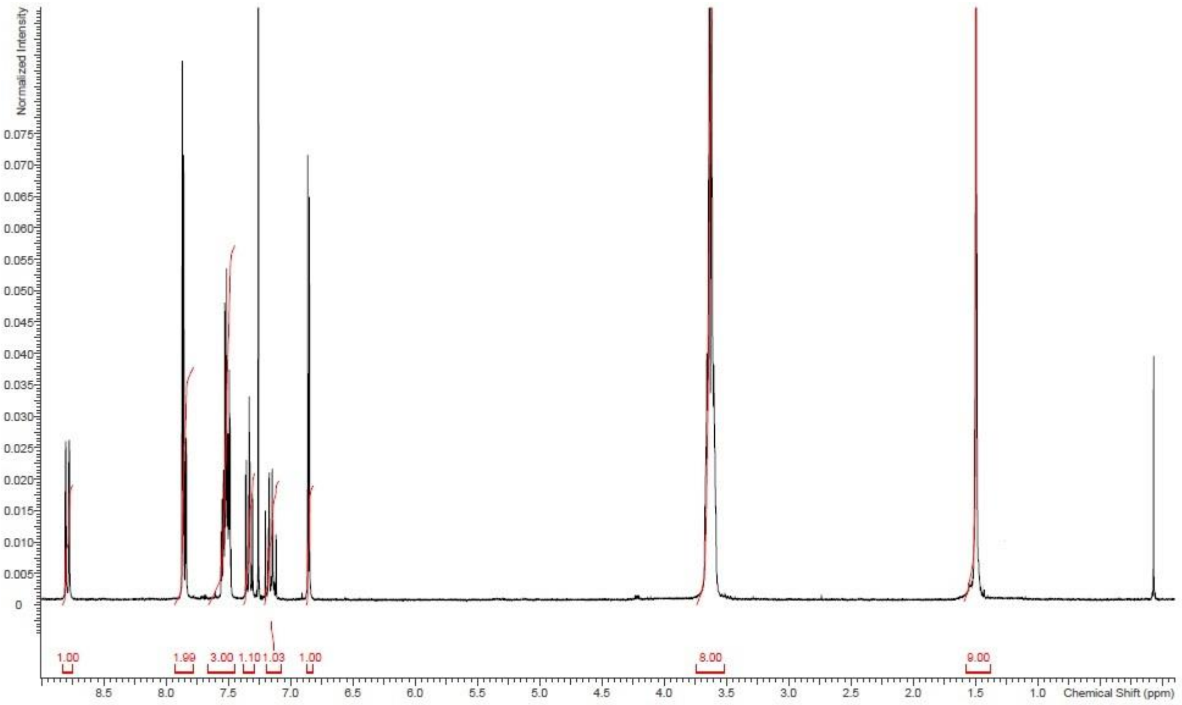

Tert-butyl-4-(1-((3,4-dichlorophenyl)sulfonyl)-1H-pyrrolo[3,2-c]quinolin-4-yl)piperazine-1-carboxylate (**26'**)

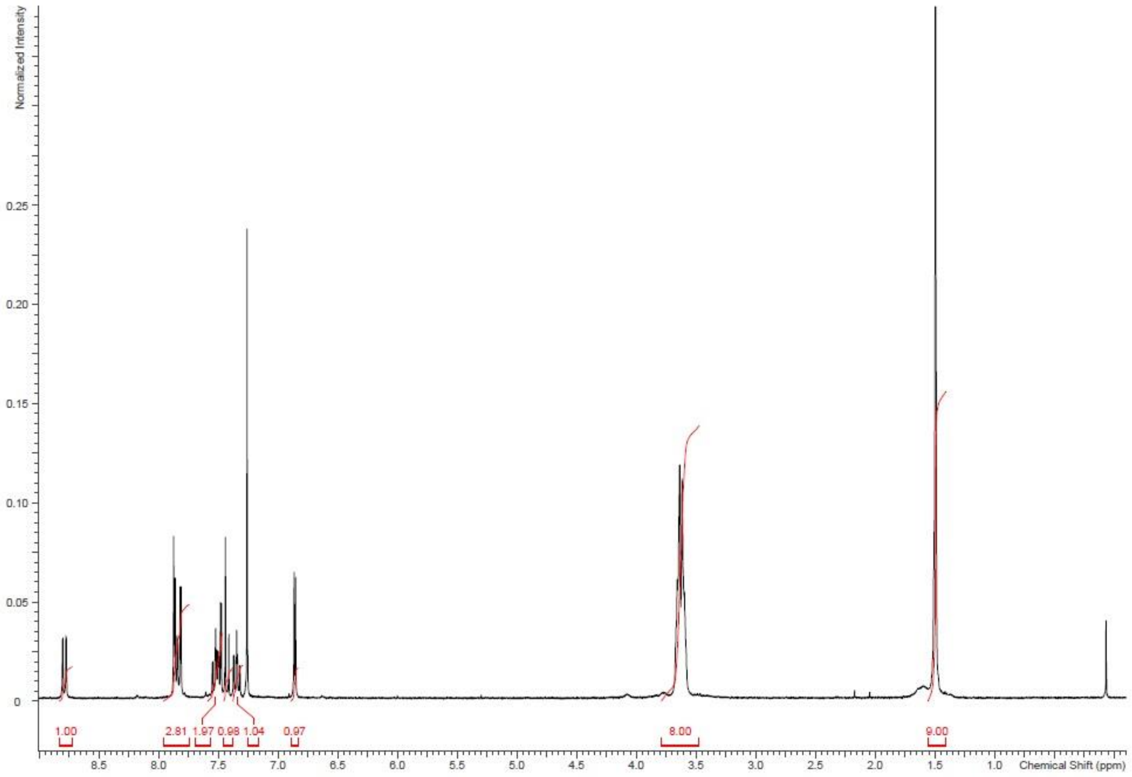

Tert-butyl-4-(1-((2,5-difluorophenyl)sulfonyl)-1H-pyrrolo[3,2-c]quinolin-4-yl)piperazine-1-carboxylate (**27'**)

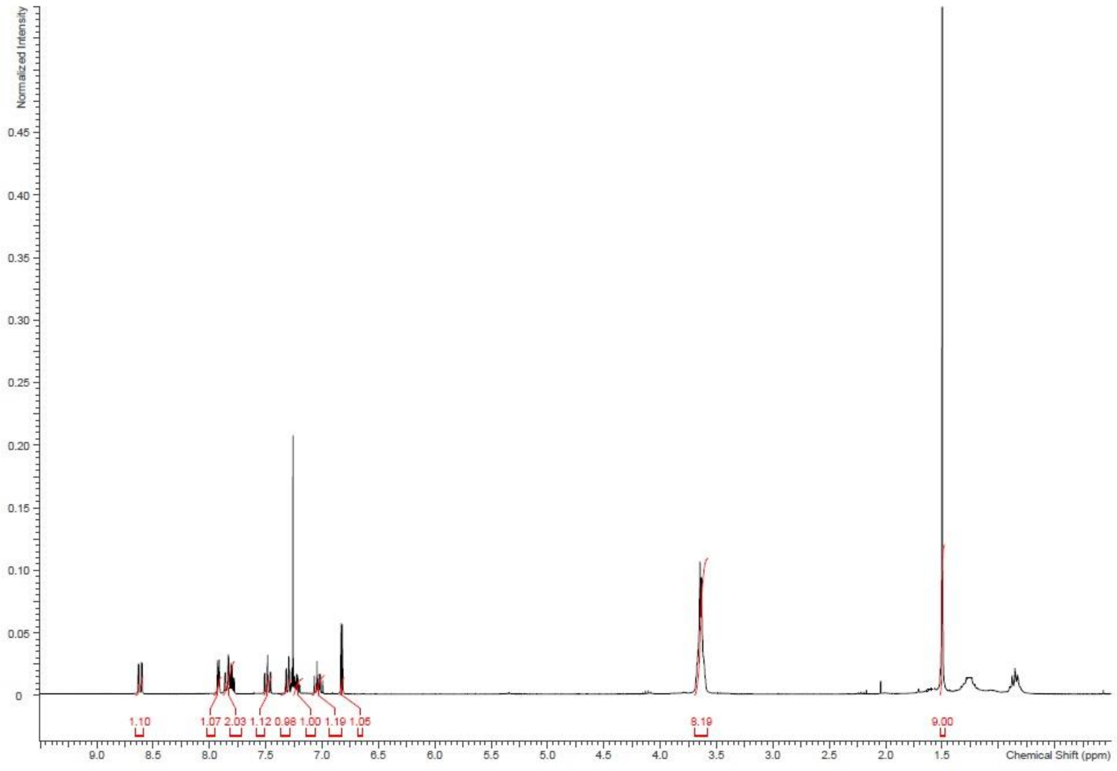

Tert-butyl-4-(1-(naphthalen-1-ylsulfonyl)-1H-pyrrolo[3,2-c]quinolin-4-yl)piperazine-1-carboxylate (**28'**)

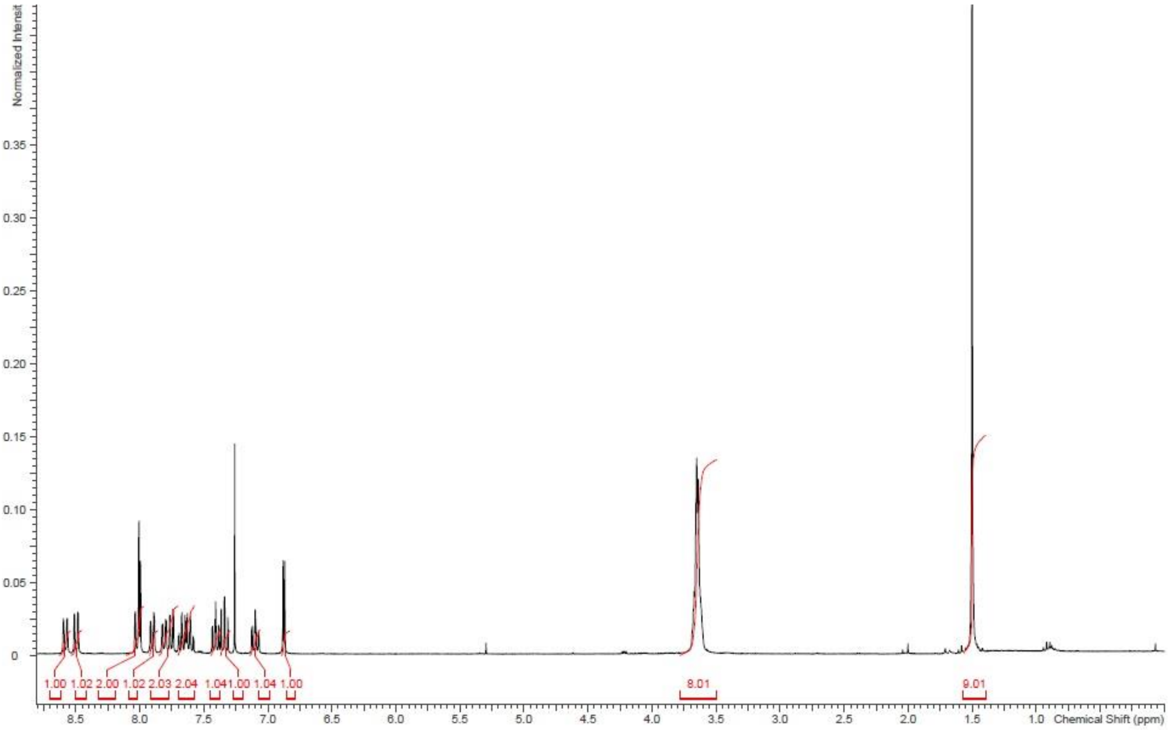

UPLC-MS, ^{1}H NMR and ^{13}C NMR spectra of final compounds 6–28

1-(Phenylsulfonyl)-4-(4-methylpiperazin-1-yl)-1H-pyrrolo[3,2-c]quinoline hydrochloride (**6**)

UPLC-MS

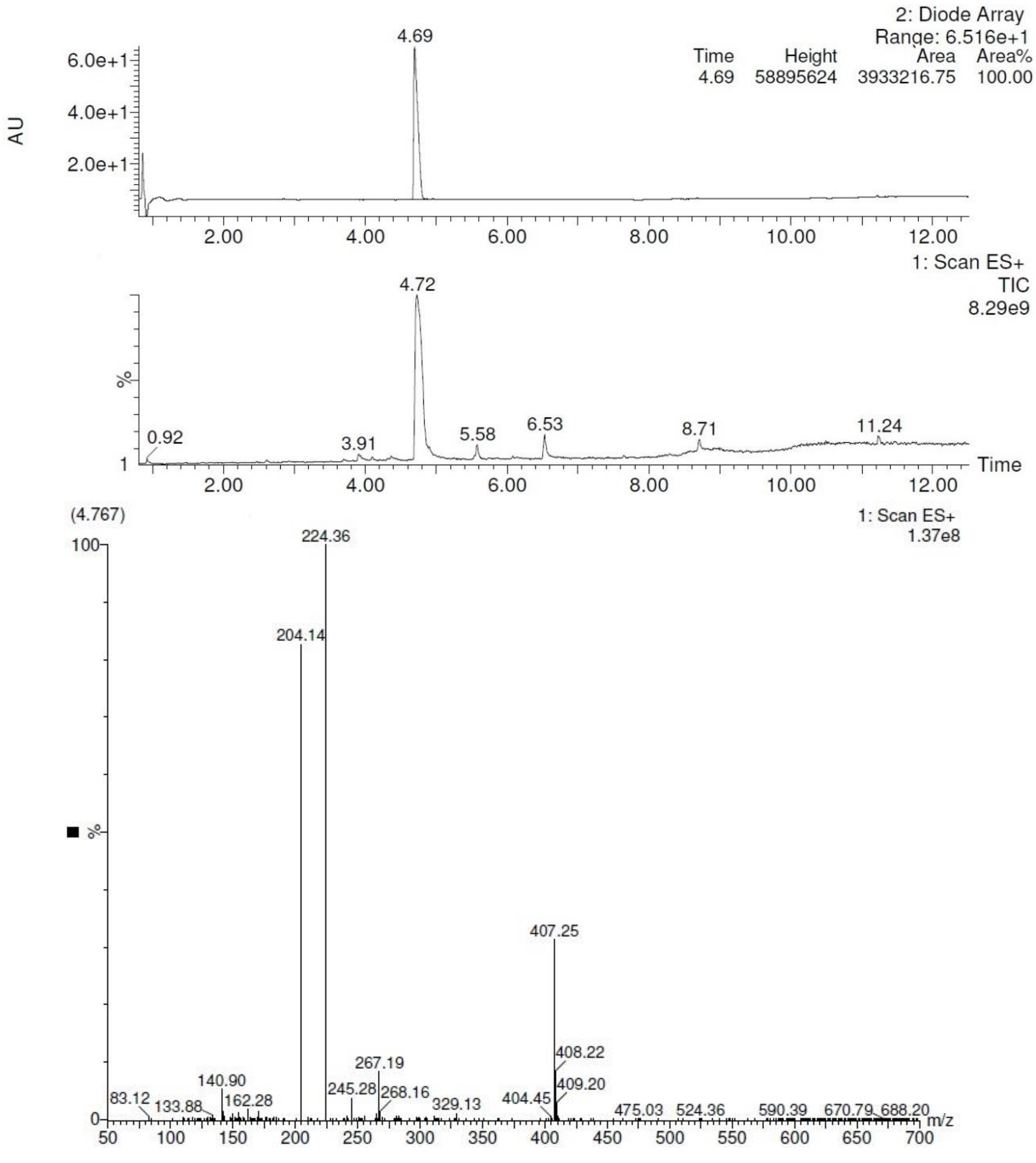

^{1}H NMR

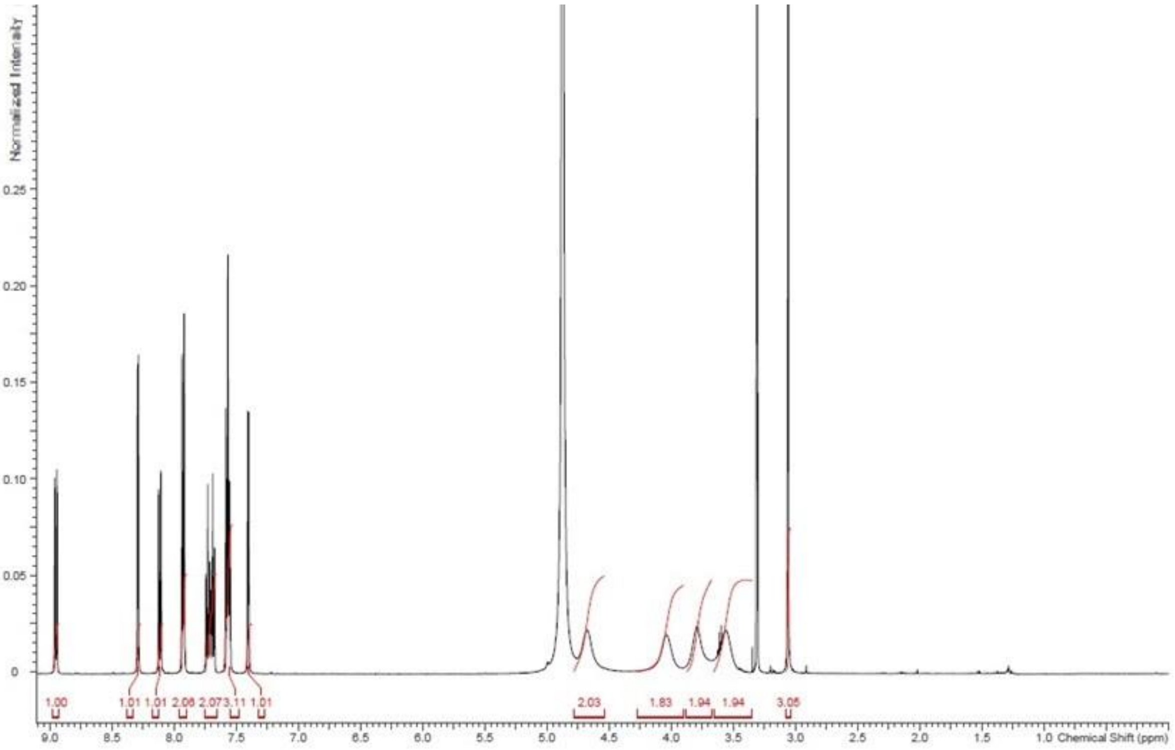

^{13}C NMR

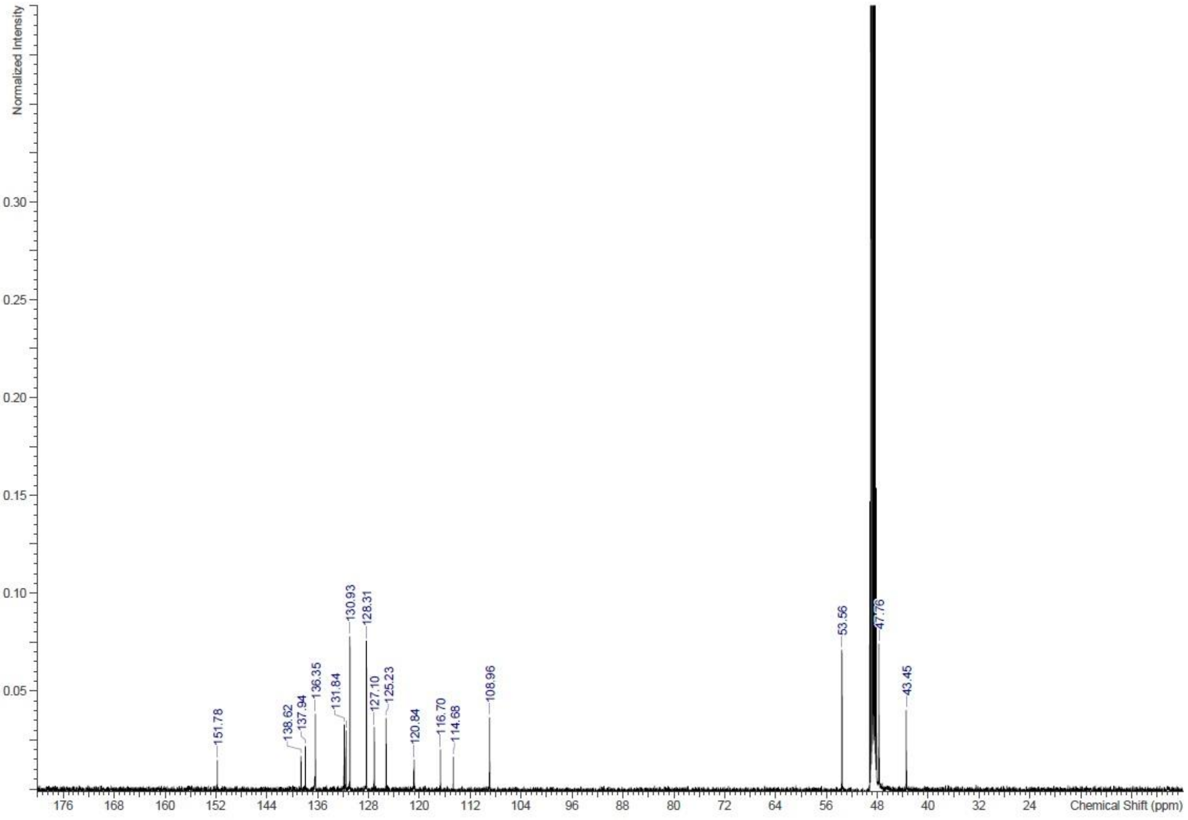

1-(Phenylsulfonyl)-4-(piperazin-1-yl)-1H-pyrrolo[3,2-c]quinoline dihydrochloride (**7**)

UPLC-MS

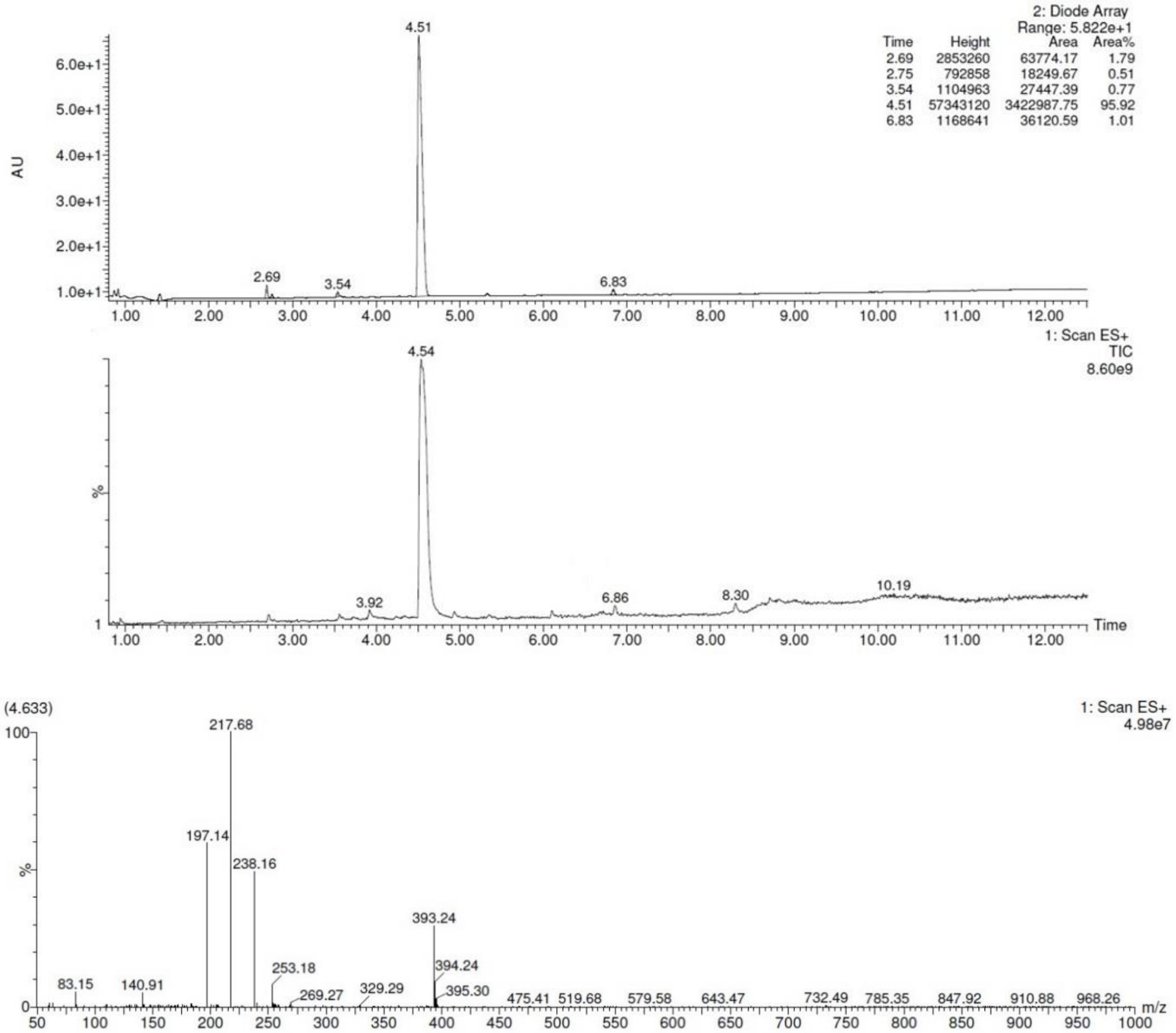

^1H NMR

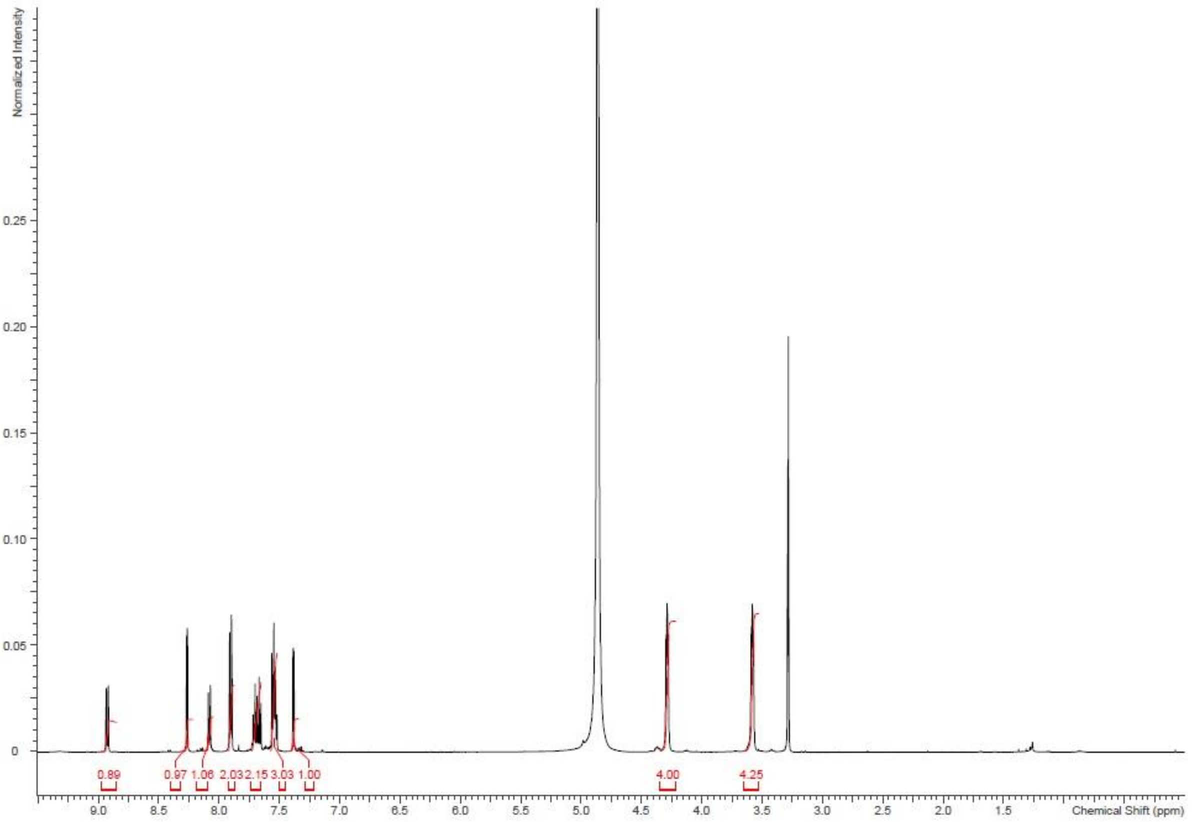

^{13}C NMR

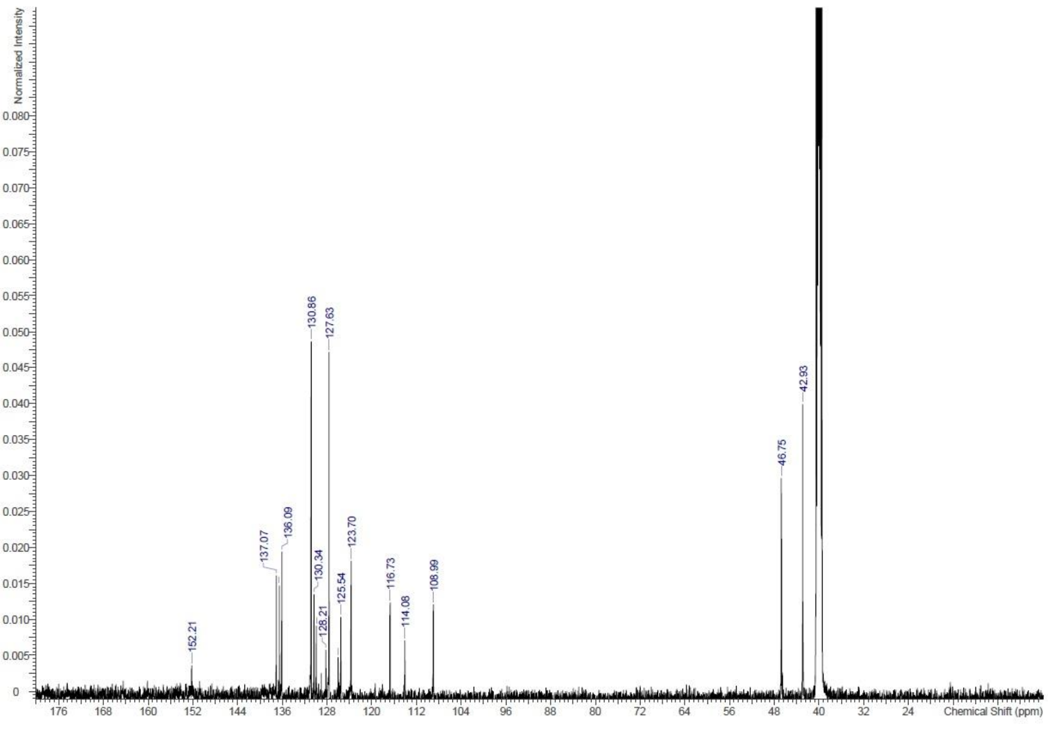

(S)-1-(Phenylsulfonyl)-N-(pyrrolidin-3-yl)-1H-pyrrolo[3,2-c]quinolin-4-amine dihydrochloride (**8**)

UPLC-MS

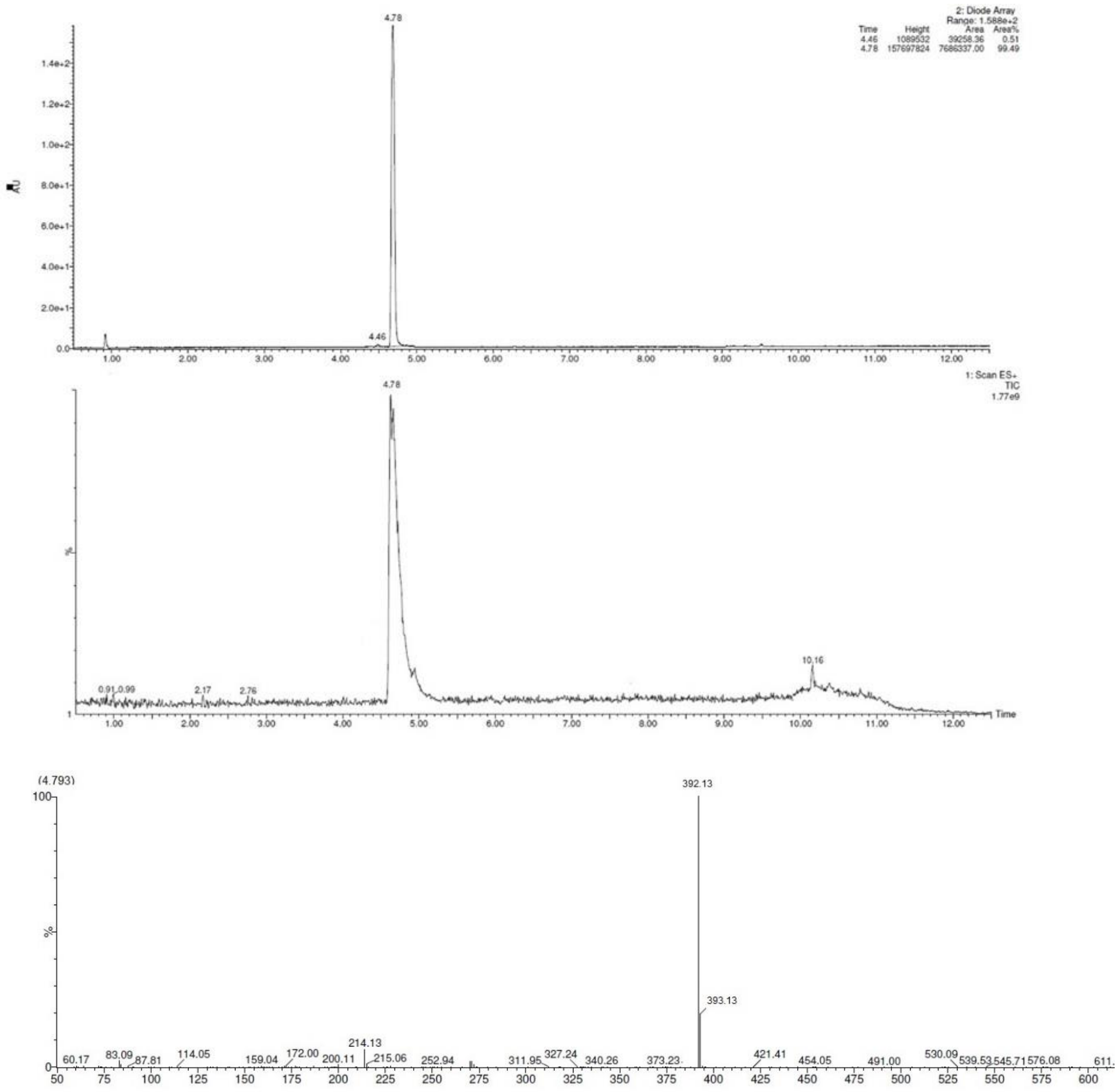

^{1}H NMR

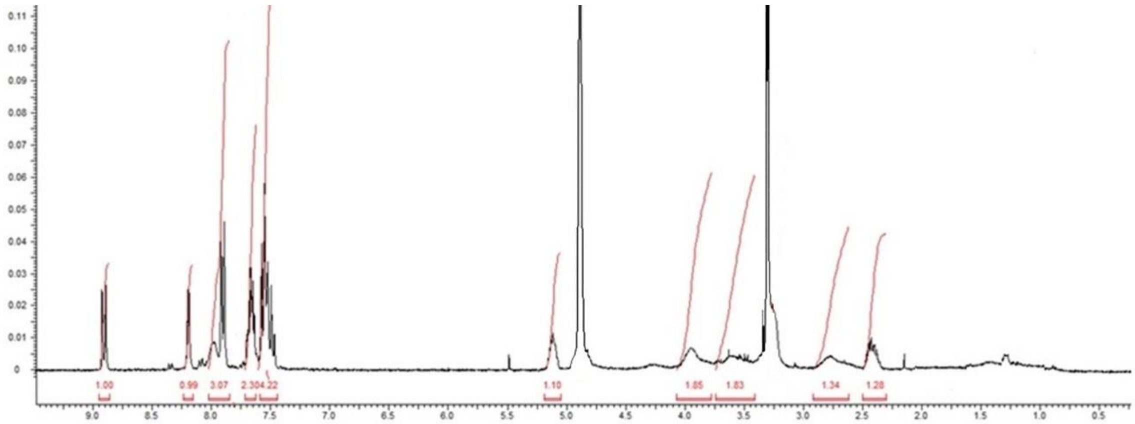

^{13}C NMR

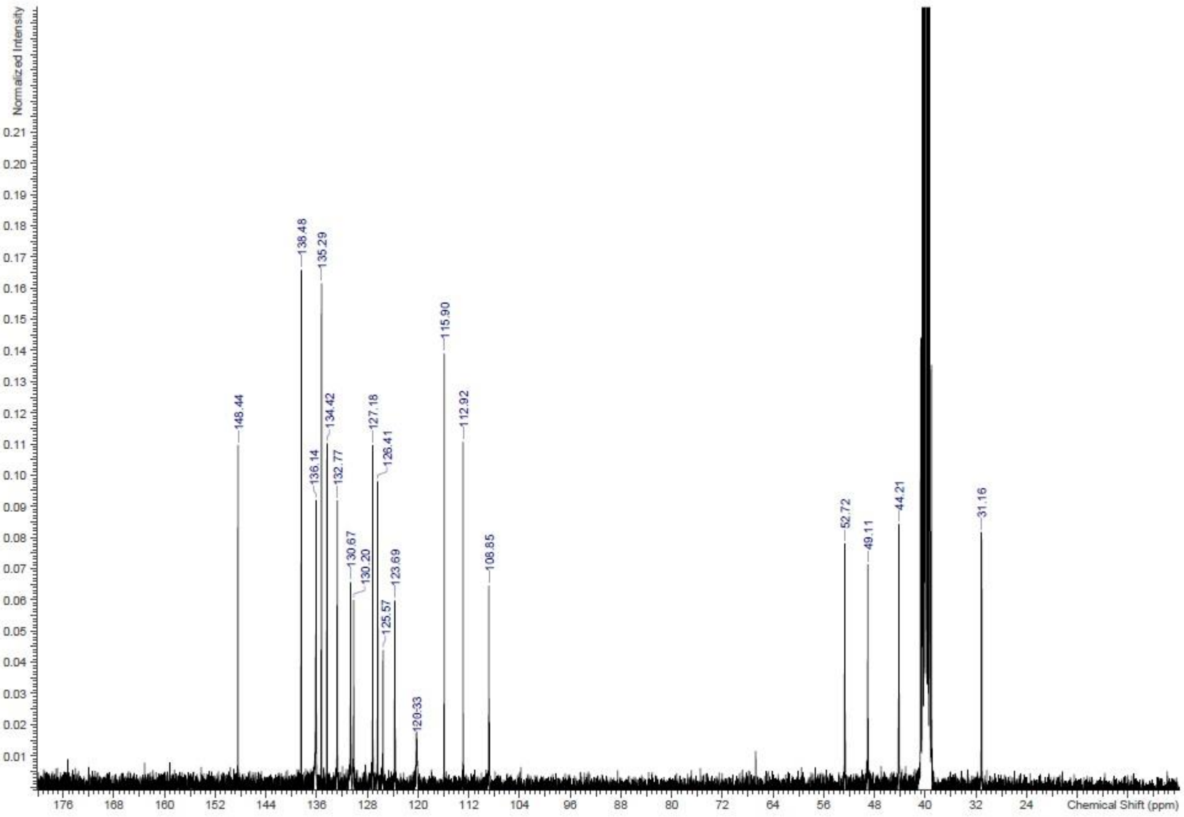

N-(Azetidin-3-yl)-1-(phenylsulfonyl)-1H-pyrrolo[3,2-c]quinolin-4-amine dihydrochloride (**9**)

UPLC-MS

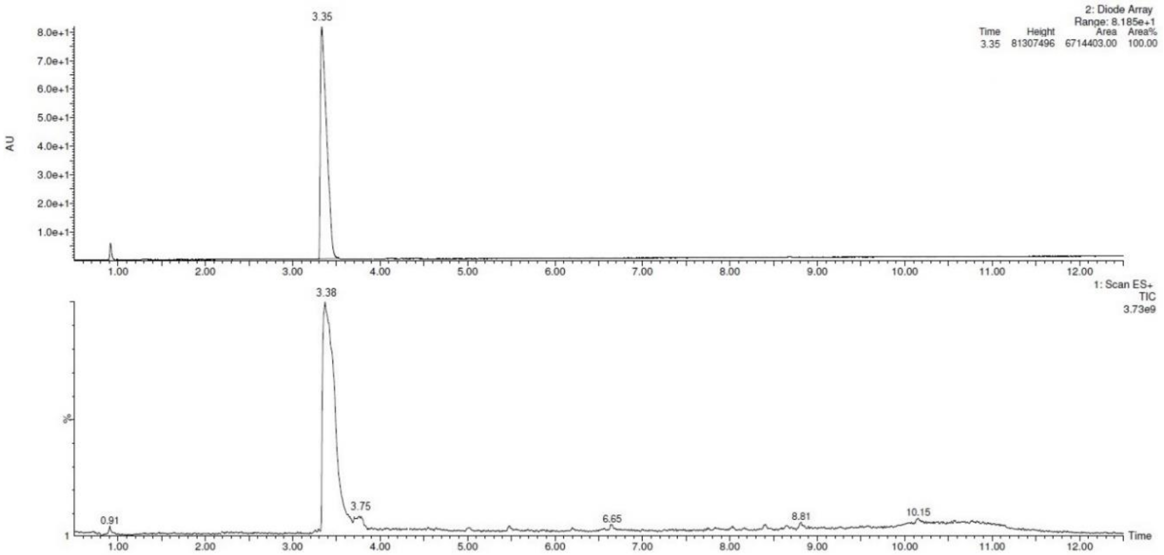

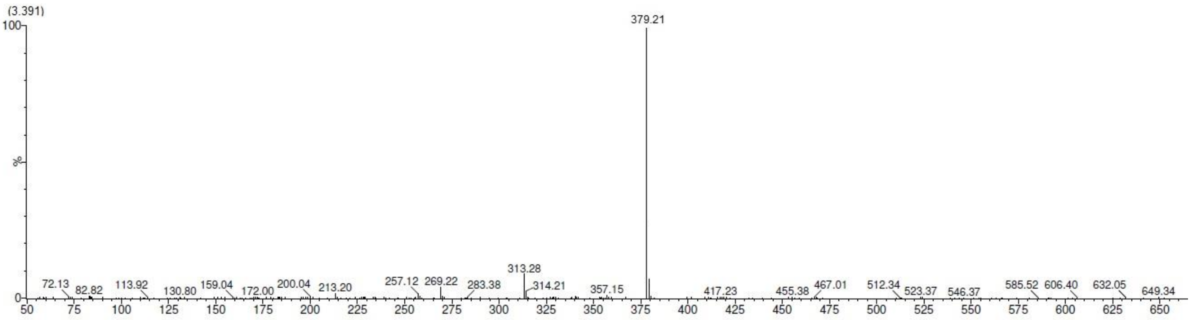

^{1}H NMR

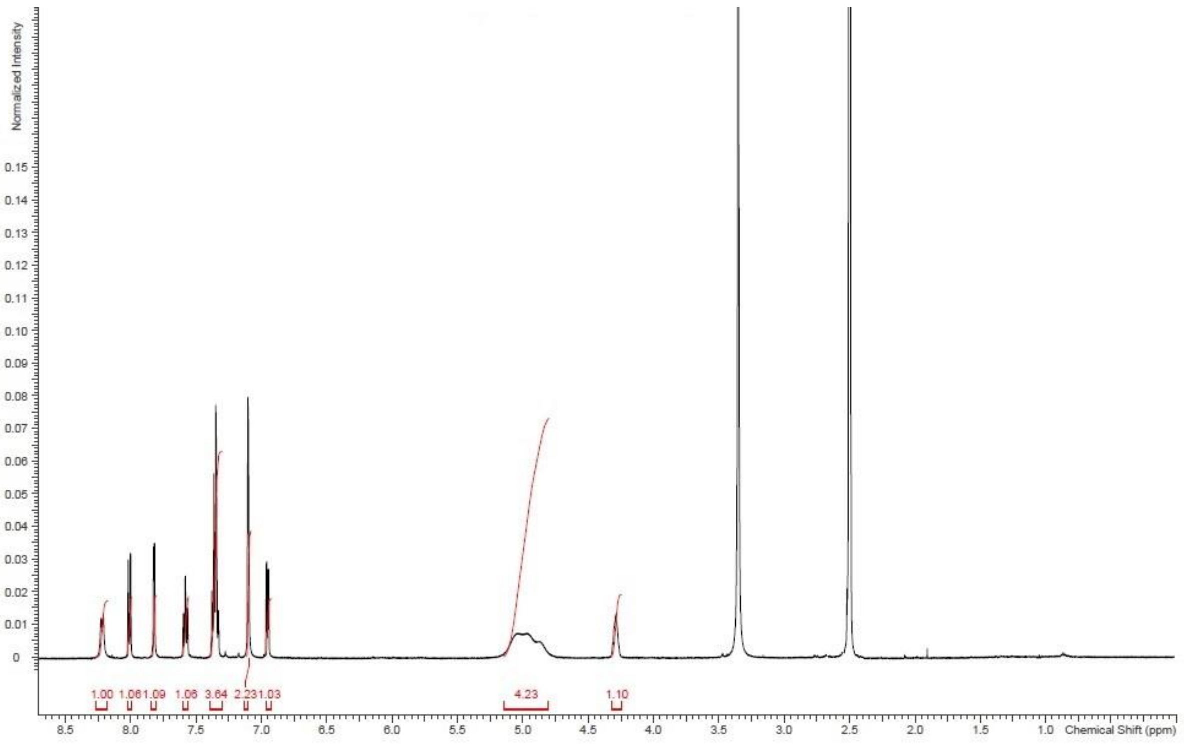

Normalized Intensity
Chemical Shift (ppm)
1.00
1.06
1.09
1.06
3.64
2.23
1.03
4.23
1.10

N-Methyl-1-(1-(phenylsulfonyl)-1H-pyrrolo[3,2-c]quinolin-4-yl)pyrrolidin-3-amine dihydrochloride (**10**)

UPLC-MS

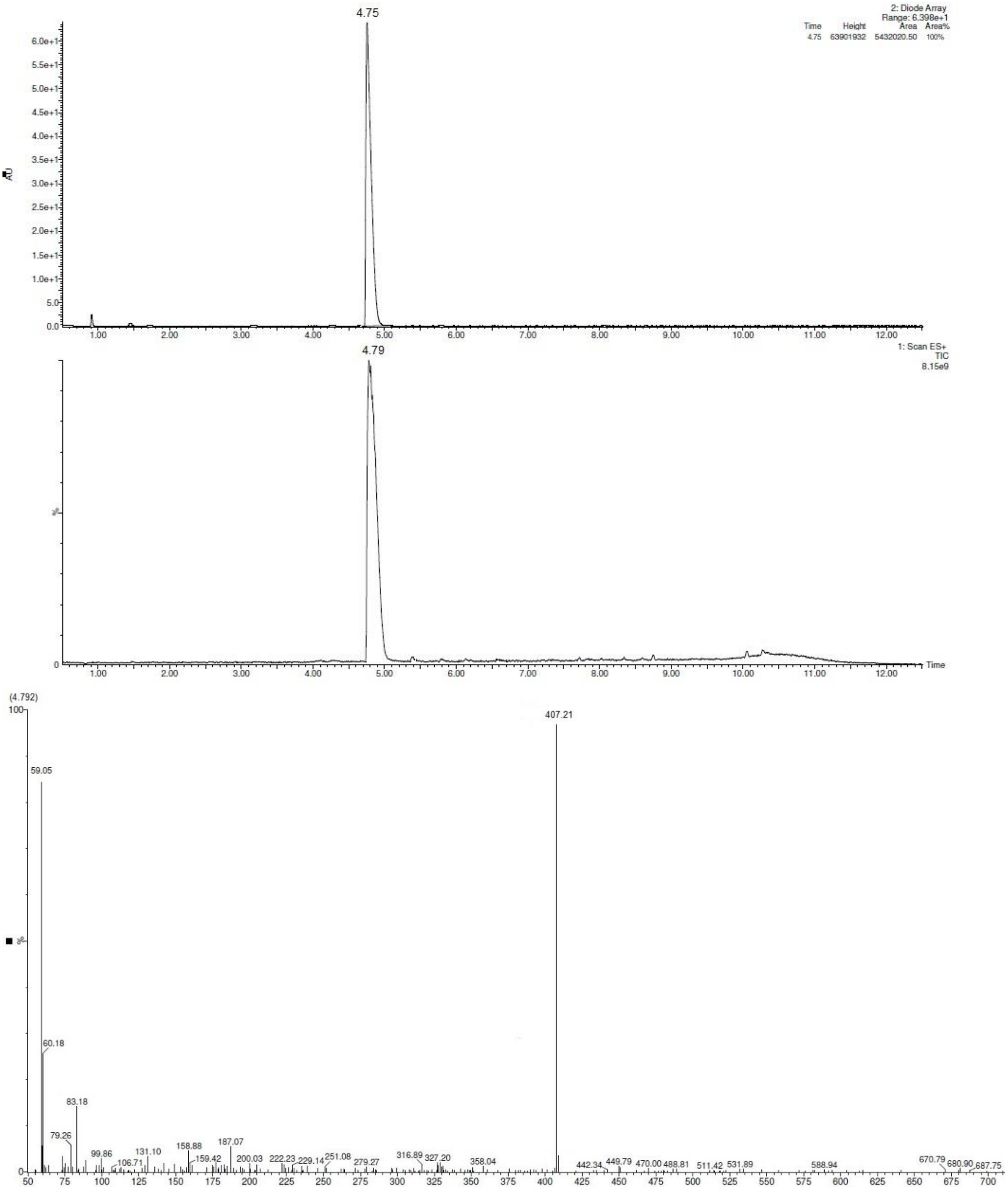

^{1}H NMR

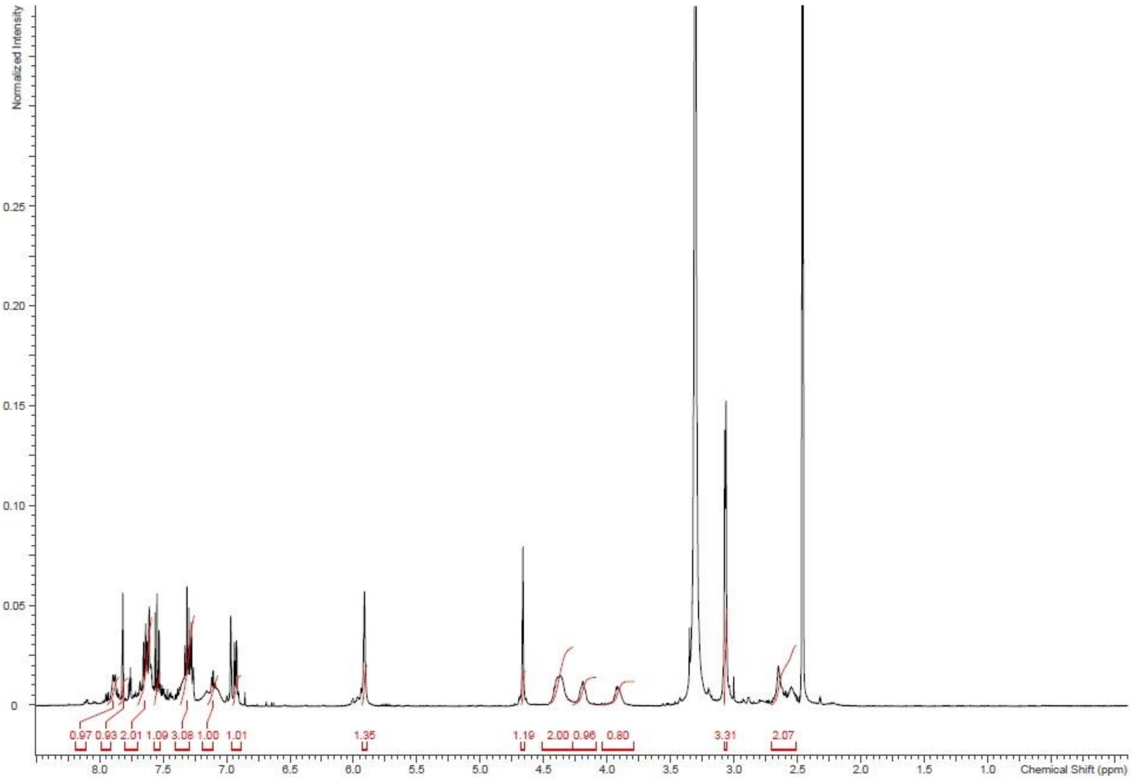
Normalized Intensity
0.25
0.20
0.15
0.10
0.05
0
0.97 0.93 2.01 1.09 3.08 1.00 1.01
1.35
1.19 2.00 0.96 0.80
3.31
2.07
8.0 7.5 7.0 6.5 6.0 5.5 5.0 4.5 4.0 3.5 3.0 2.5 2.0 1.5 1.0
Chemical Shift (ppm)

4-(1,4-Diazepan-1-yl)-1-(phenylsulfonyl)-1H-pyrrolo[3,2-c]quinoline dihydrochloride (**11**)

UPLC-MS

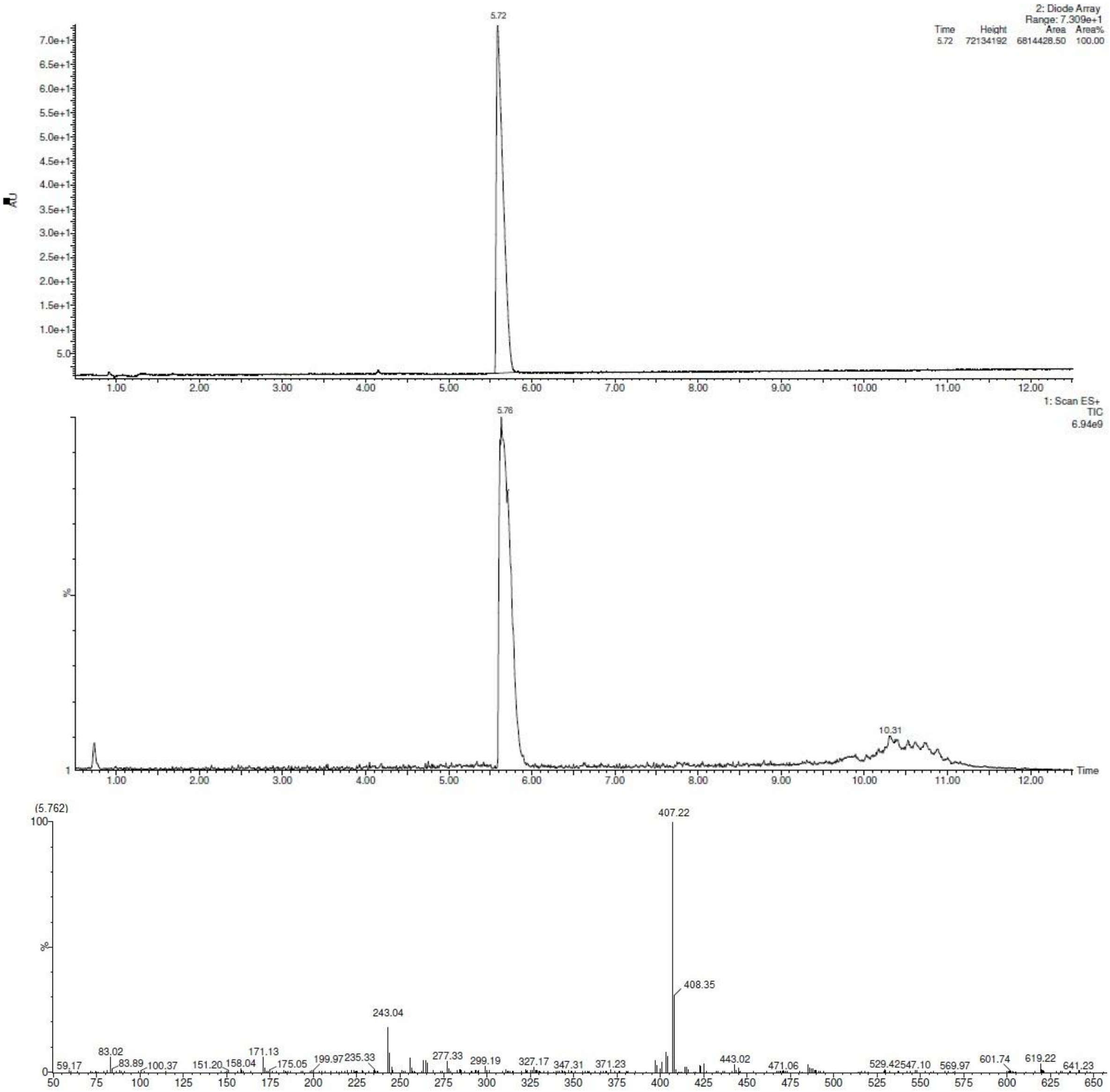

^{1}H NMR

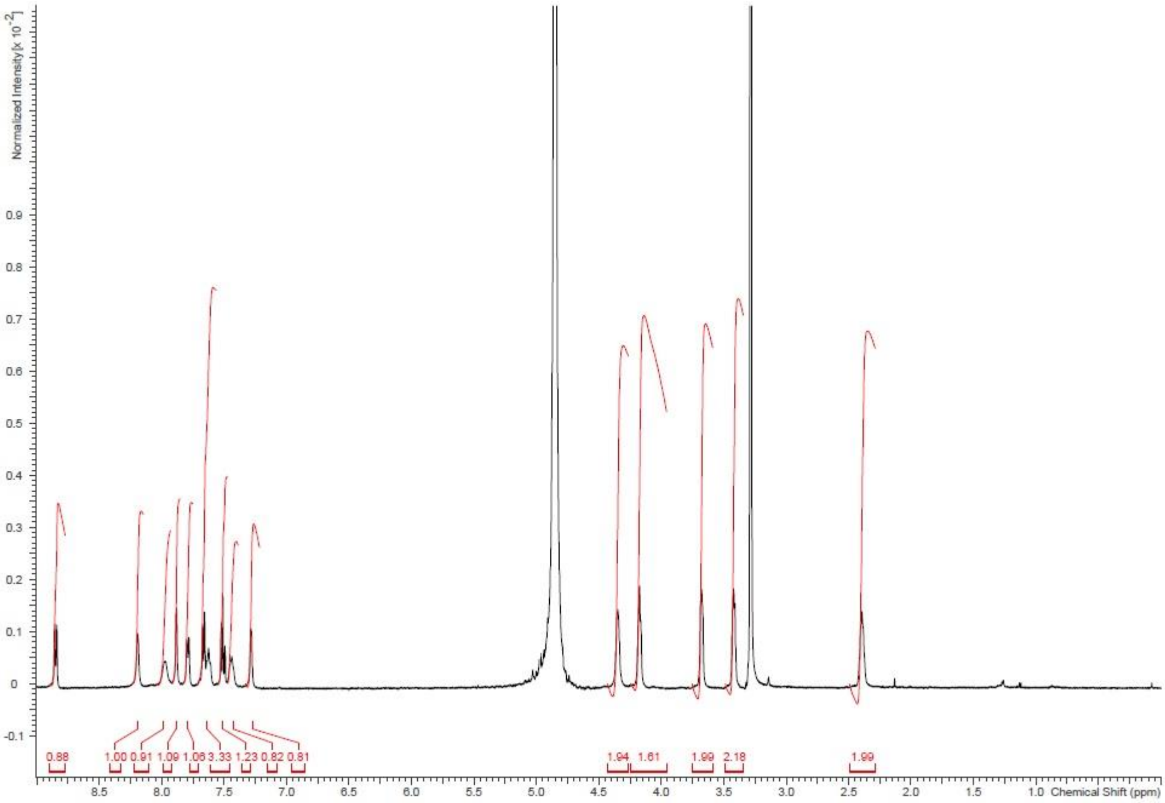

^{13}C NMR

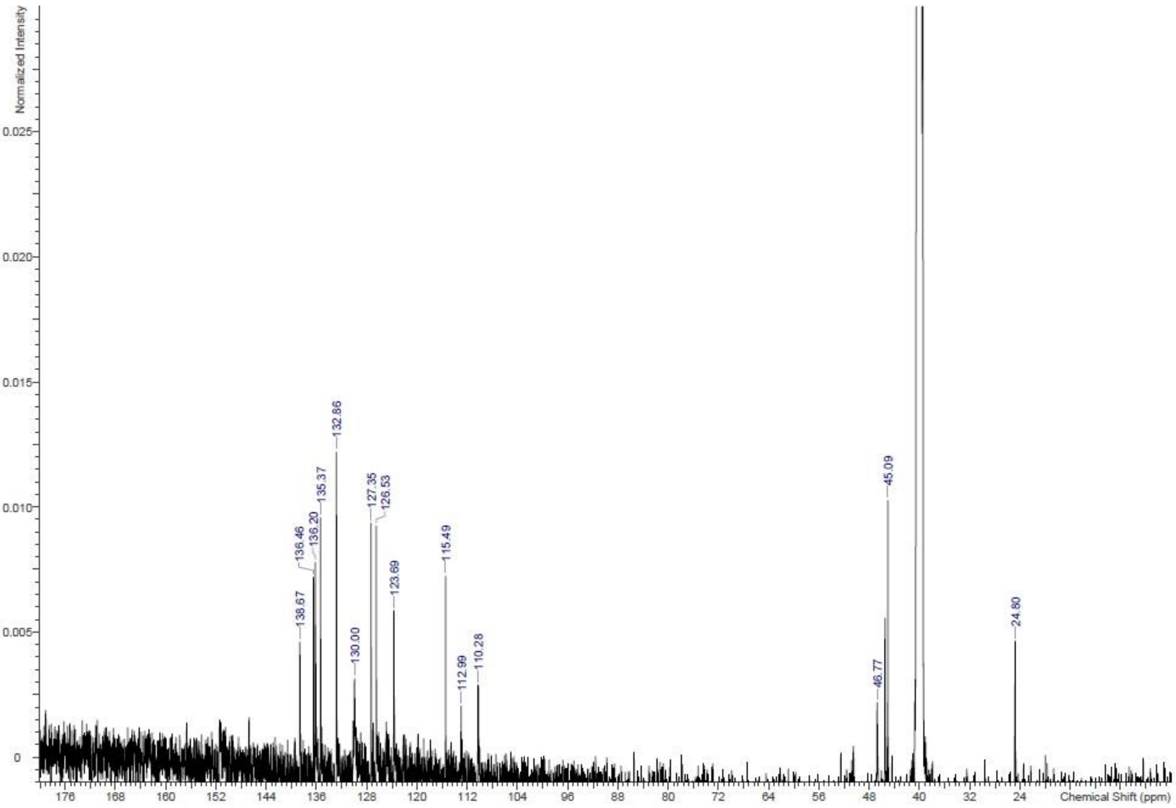

1-[(3-Fluorophenyl)sulfonyl]-4-(4-methylpiperazin-1-yl)-1H-pyrrolo[3,2-c]quinoline hydrochloride (**12**)

UPLC-MS

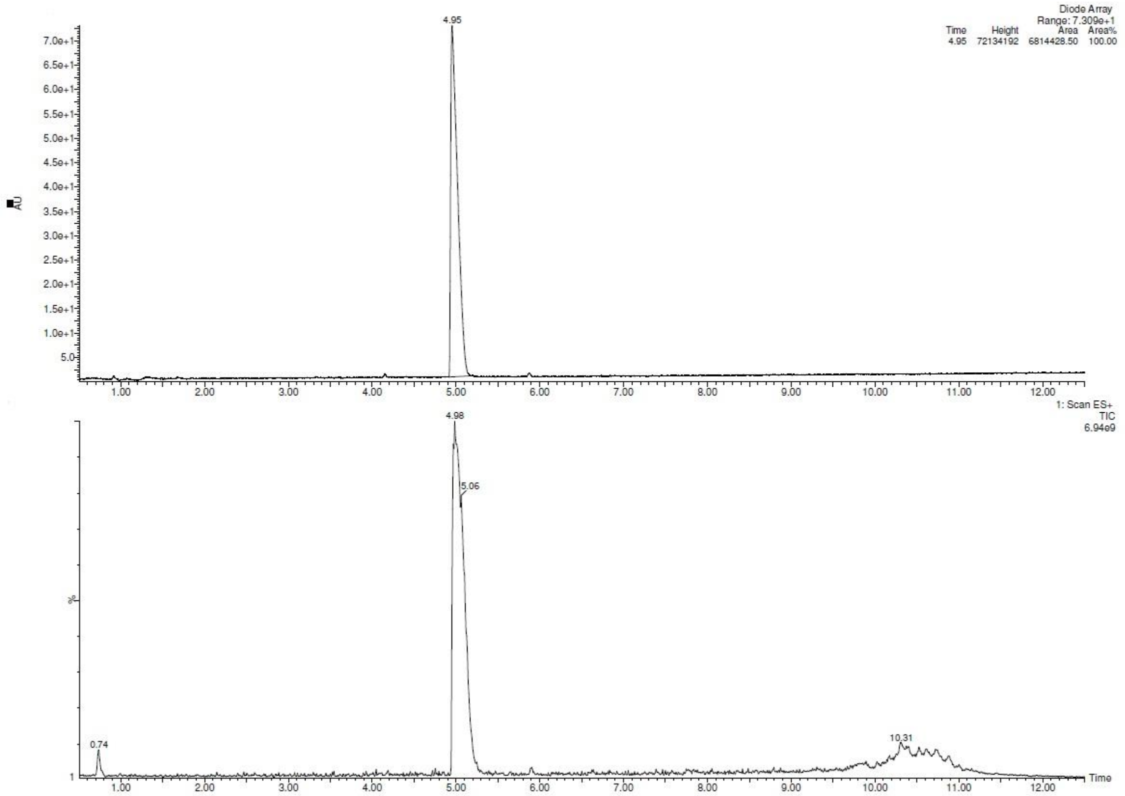

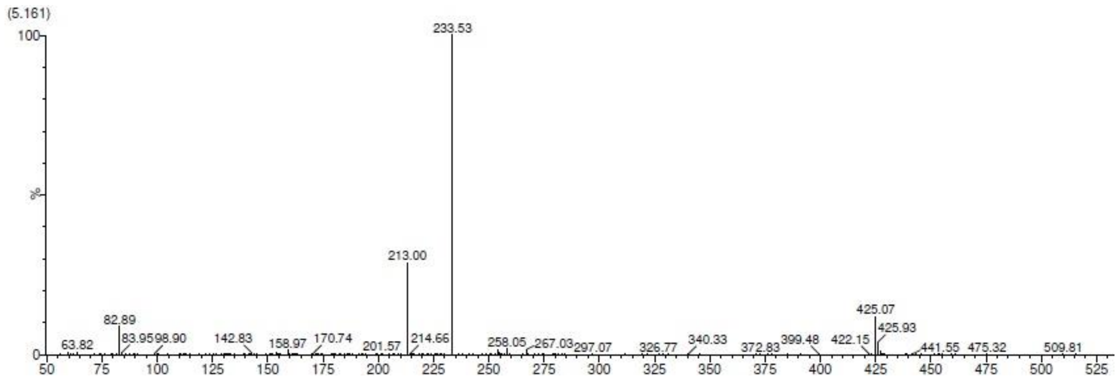

^{1}H NMR

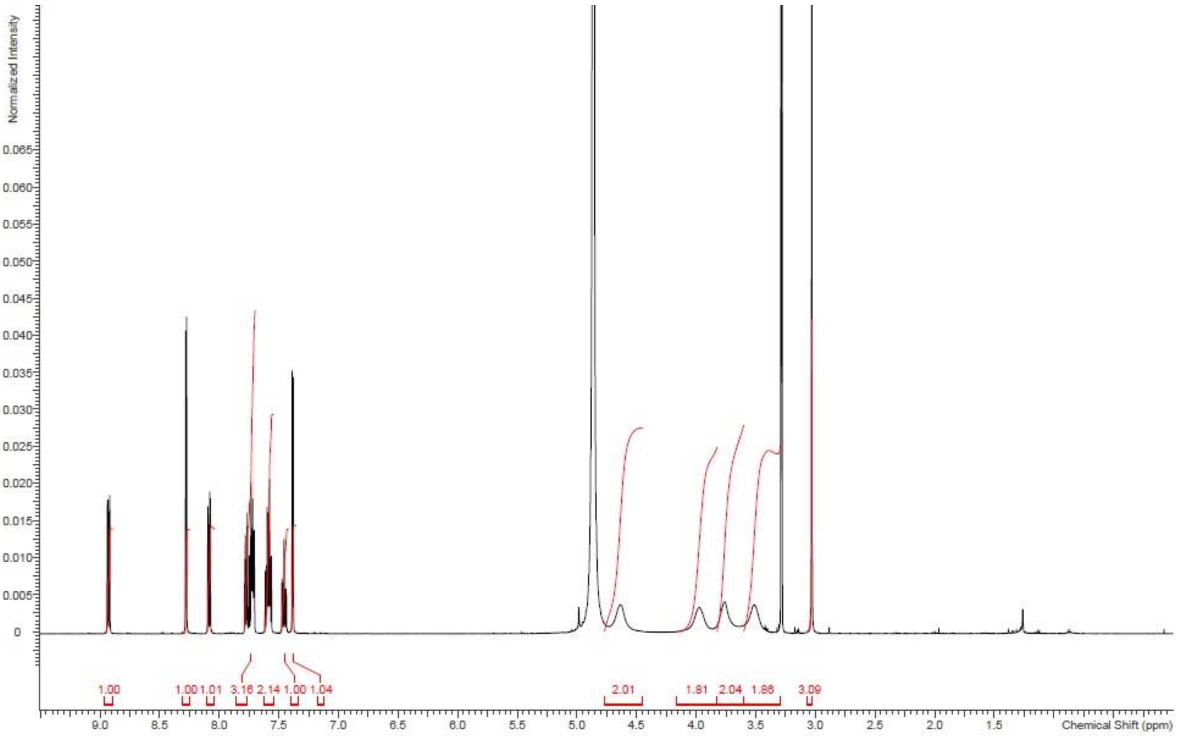

^{13}C NMR

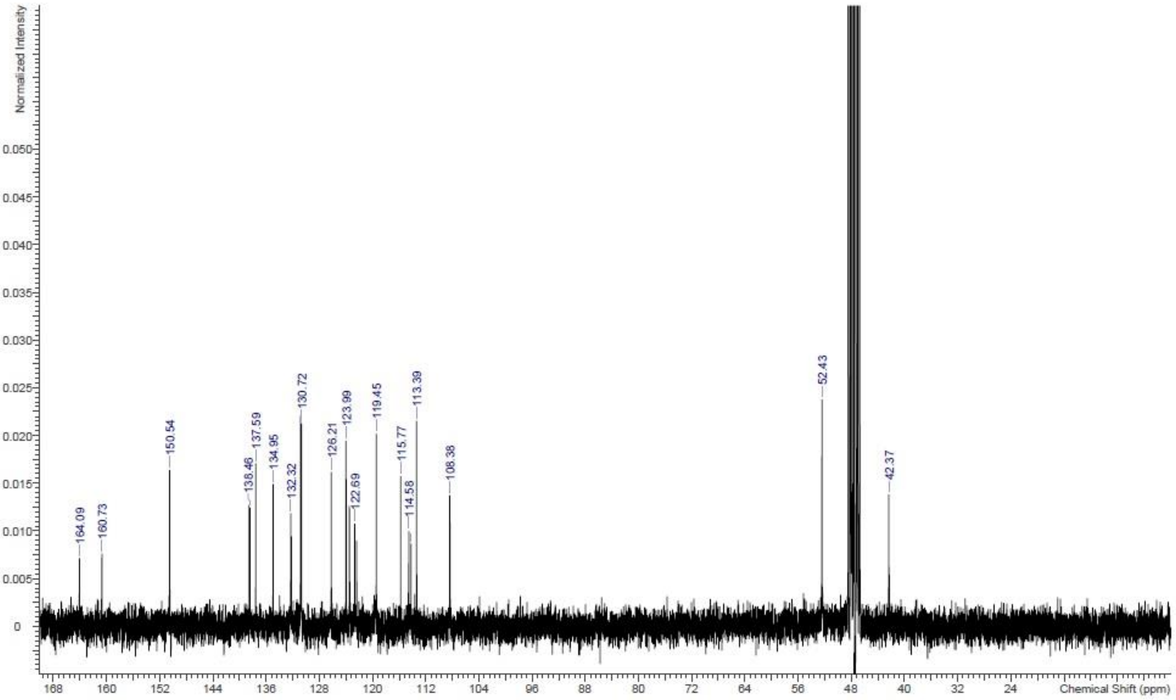

1-[(3-Chlorophenyl)sulfonyl]-4-(4-methylpiperazin-1-yl)-1H-pyrrolo[3,2-c]quinoline hydrochloride (**13**)

UPLC-MS

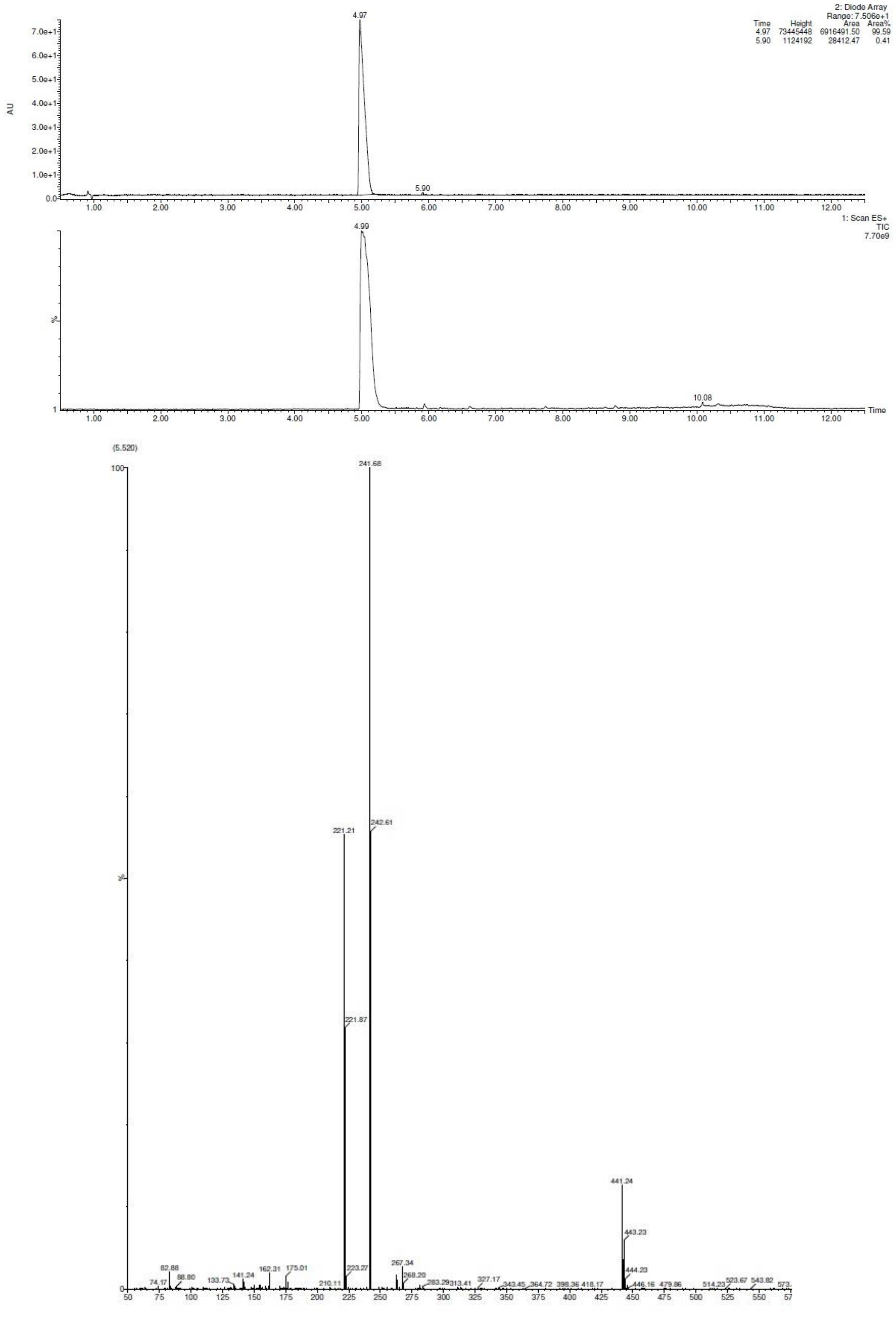

^{1}H NMR

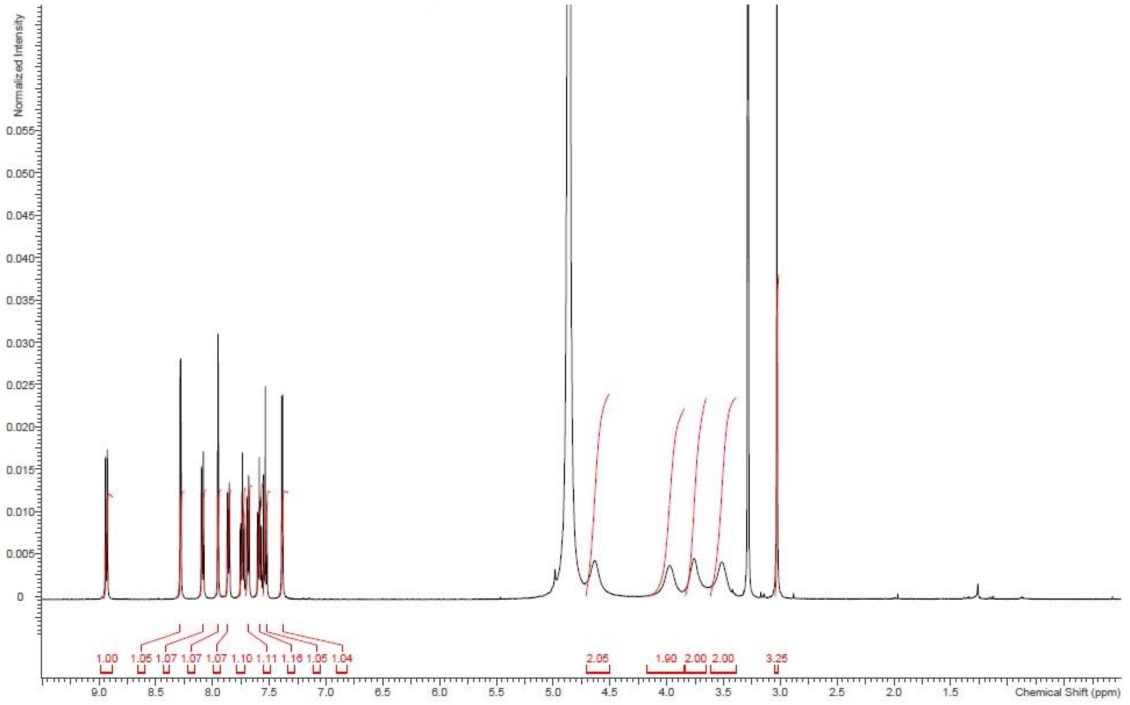

^{13}C NMR

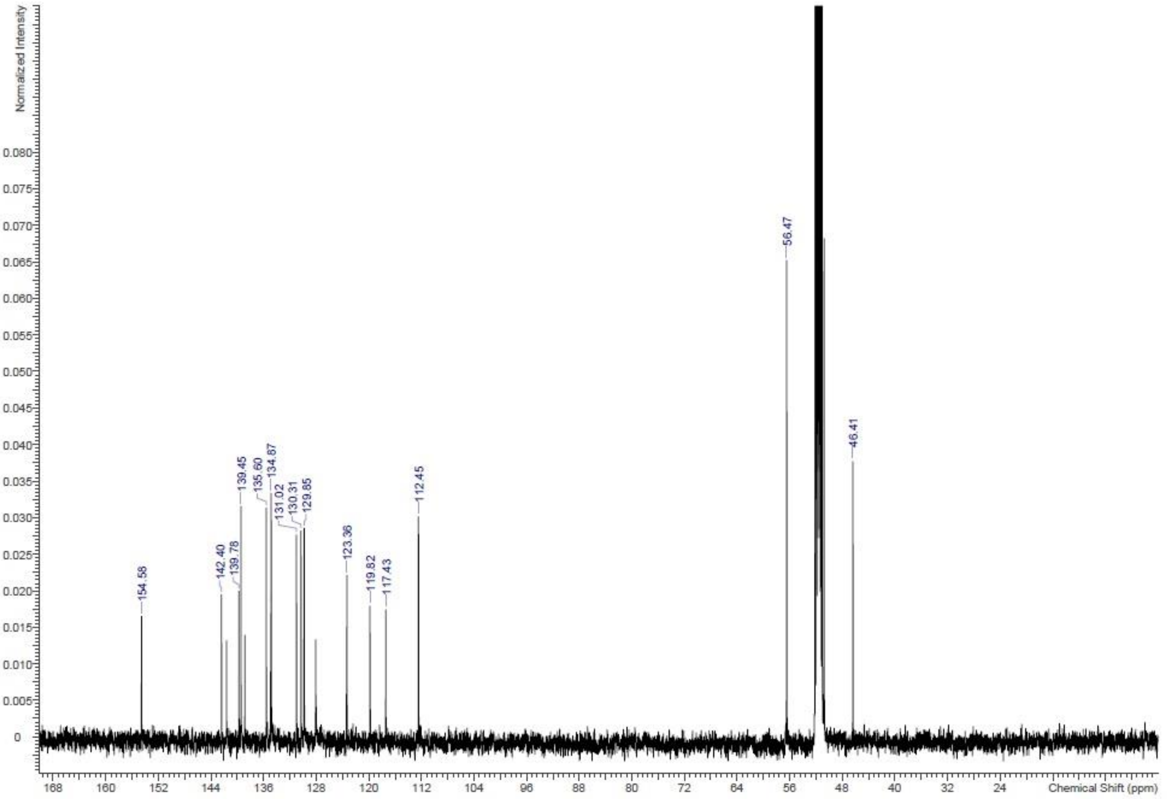

1-((4-Fluorophenyl)sulfonyl)-4-(4-methylpiperazin-1-yl)-1H-pyrrolo[3,2-c]quinoline hydrochloride (**14**)

UPLC-MS

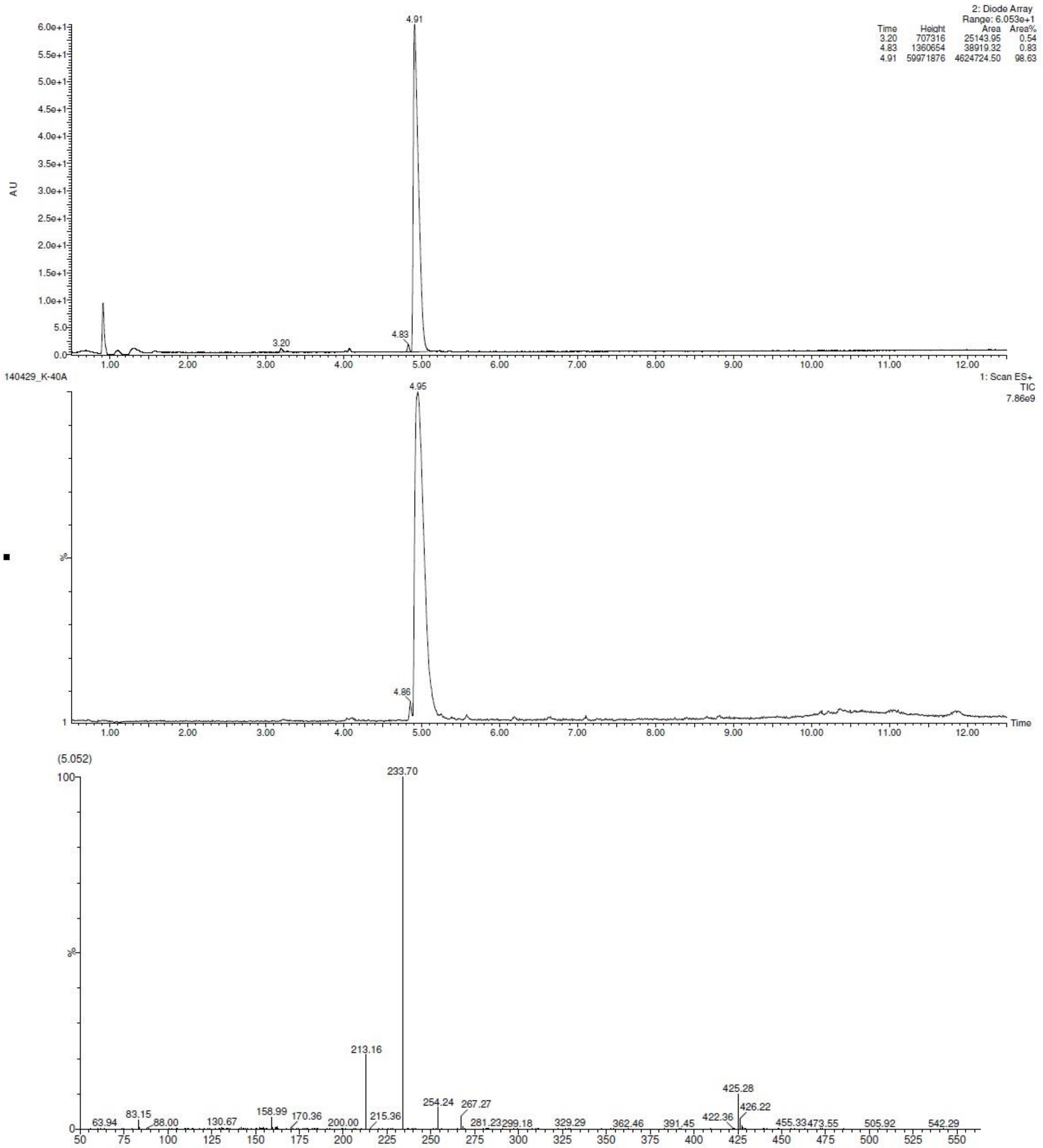

^1H NMR

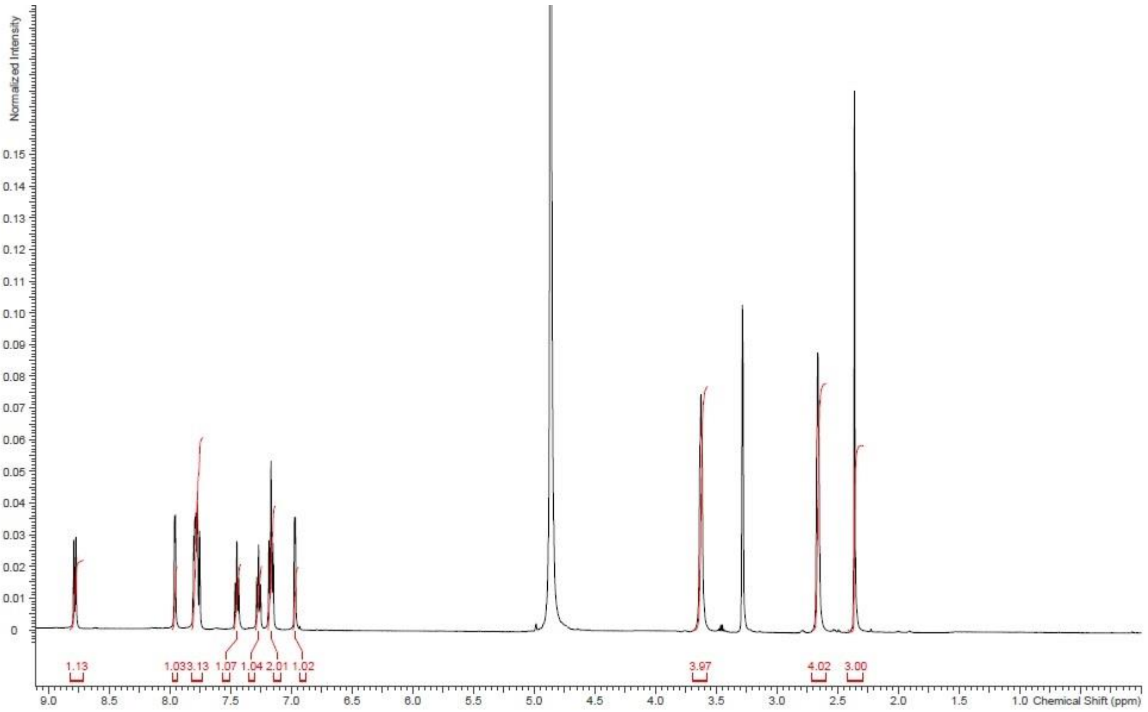

^{13}C NMR

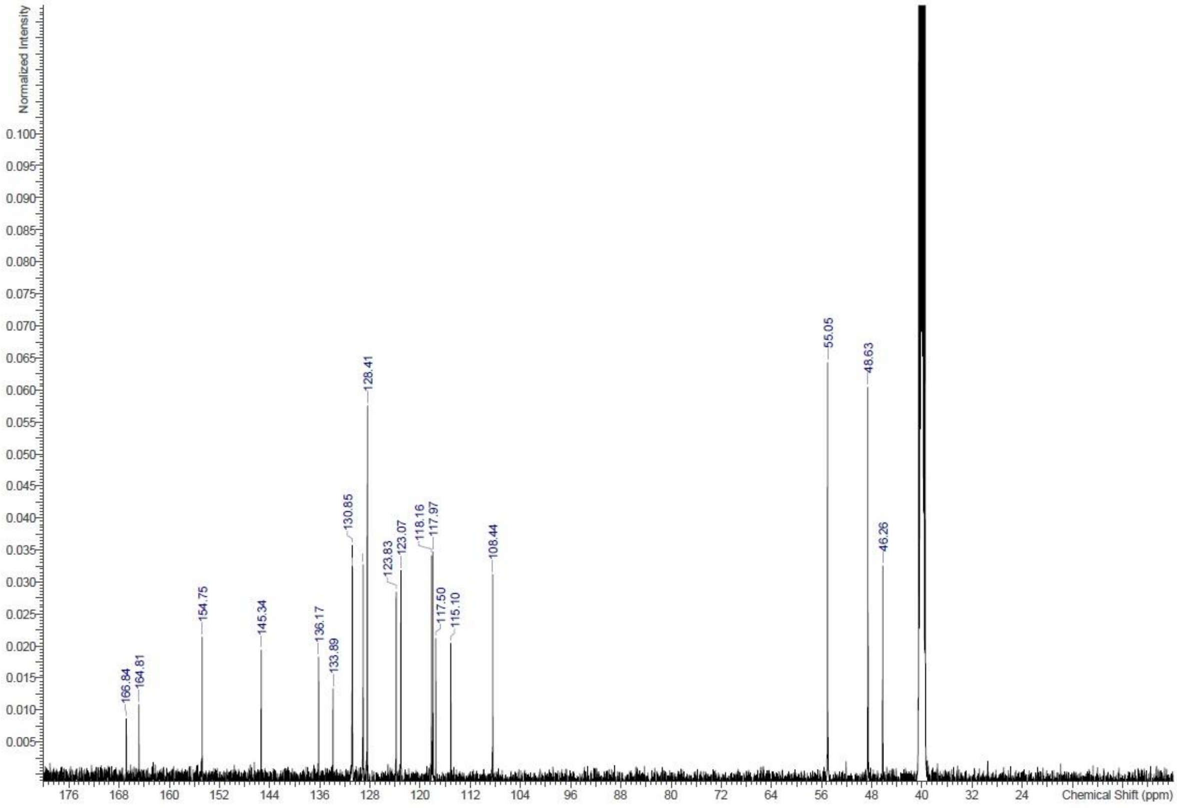

1-[(2-Bromophenyl)sulfonyl]-4-(piperazin-1-yl)-1H-pyrrolo[3,2-c]quinoline dihydrochloride (**15**)

UPLC-MS

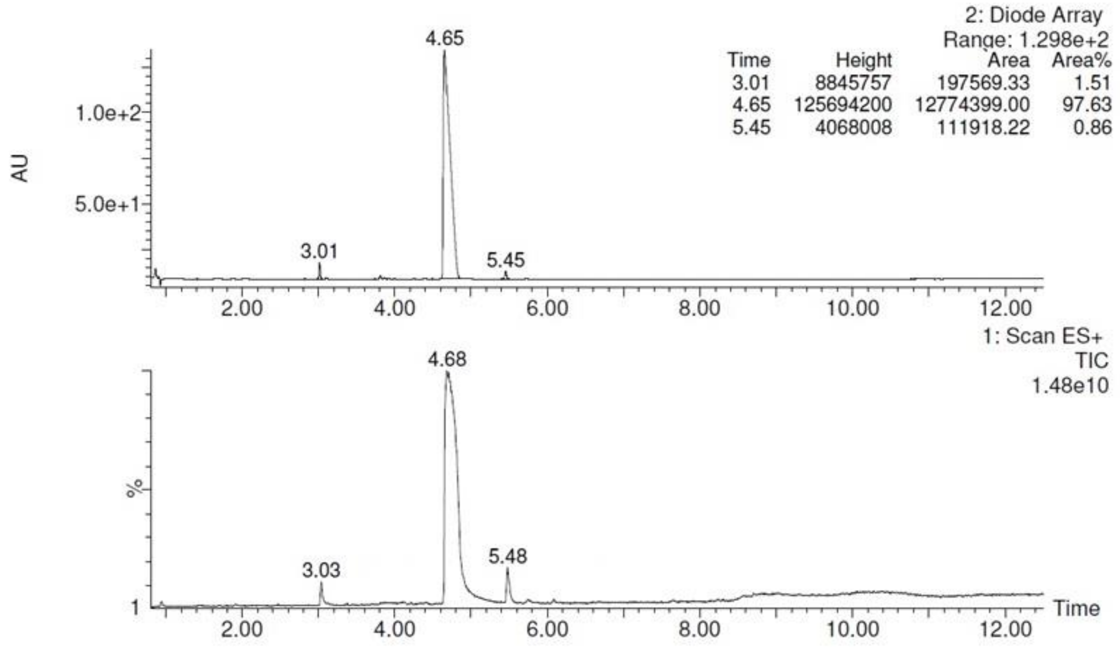

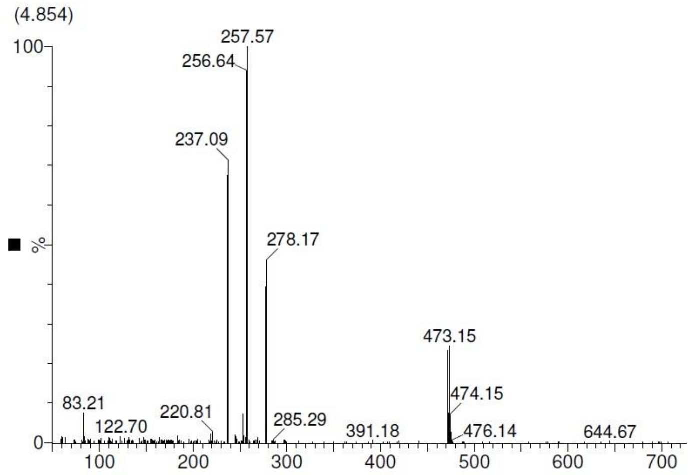

^{1}H NMR

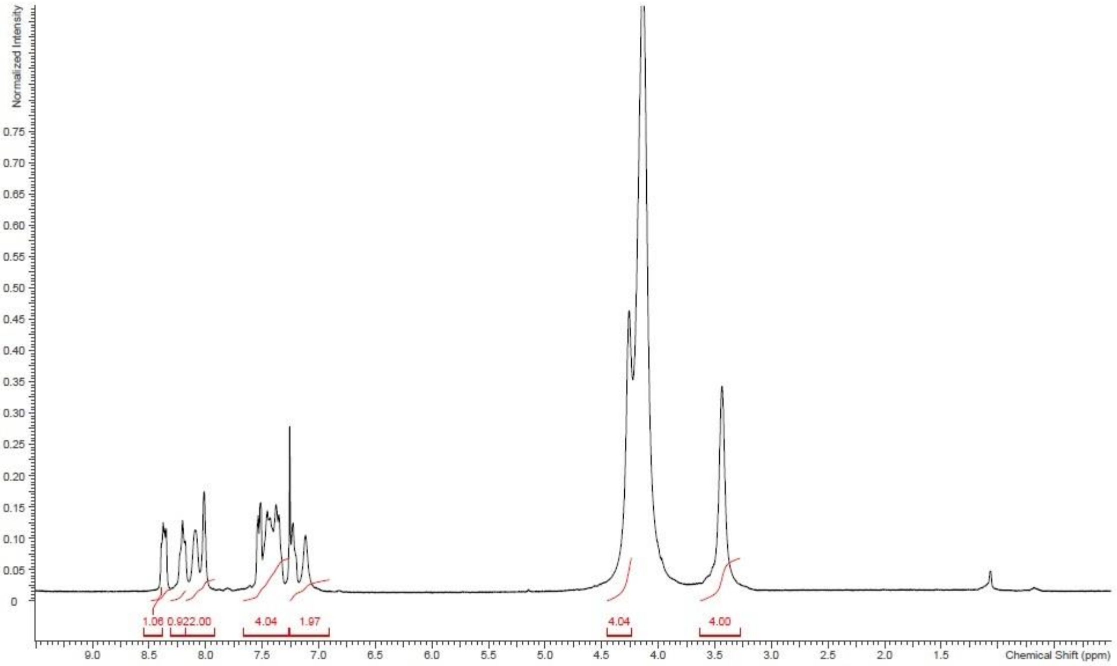

^{13}C NMR

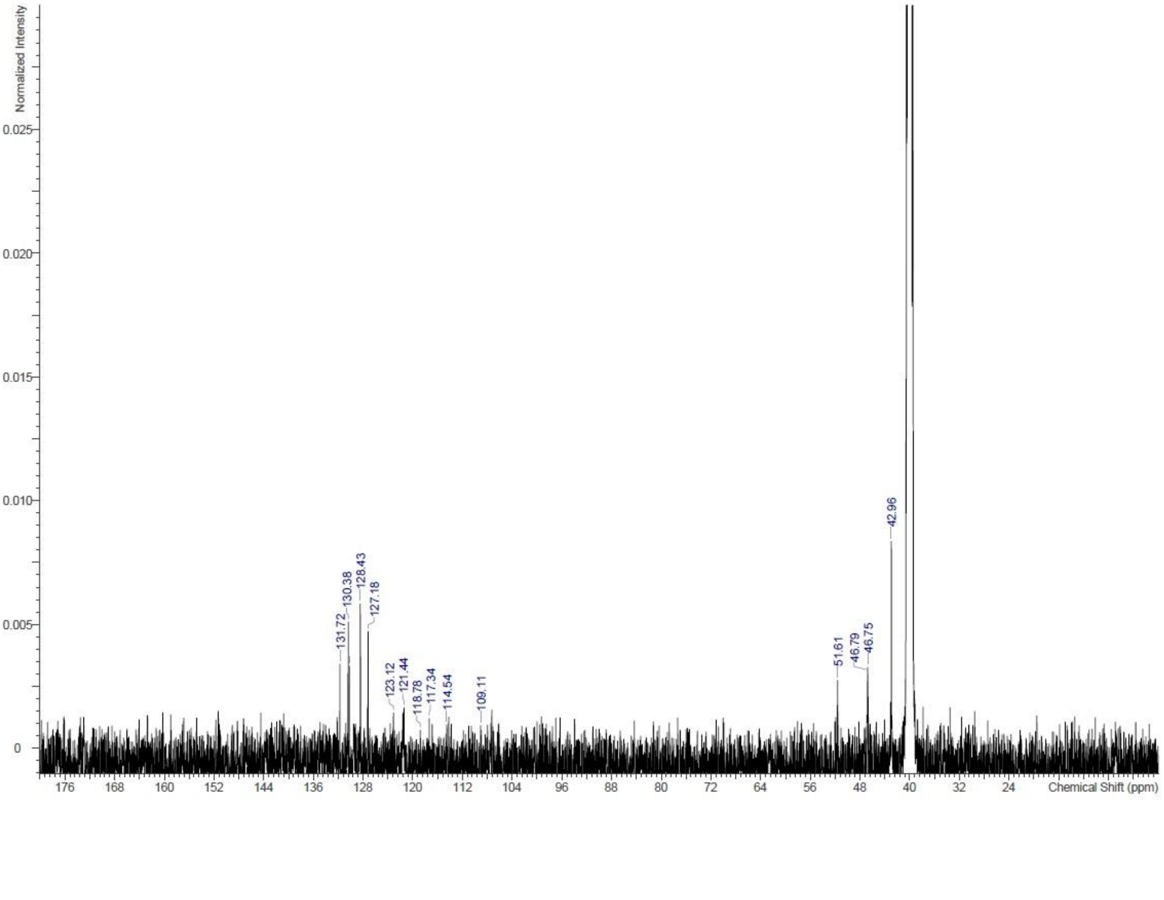

1-[(2-Chlorophenyl)sulfonyl]-4-(piperazin-1-yl)-1H-pyrrolo[3,2-c]quinoline dihydrochloride (**16**)

UPLC-MS

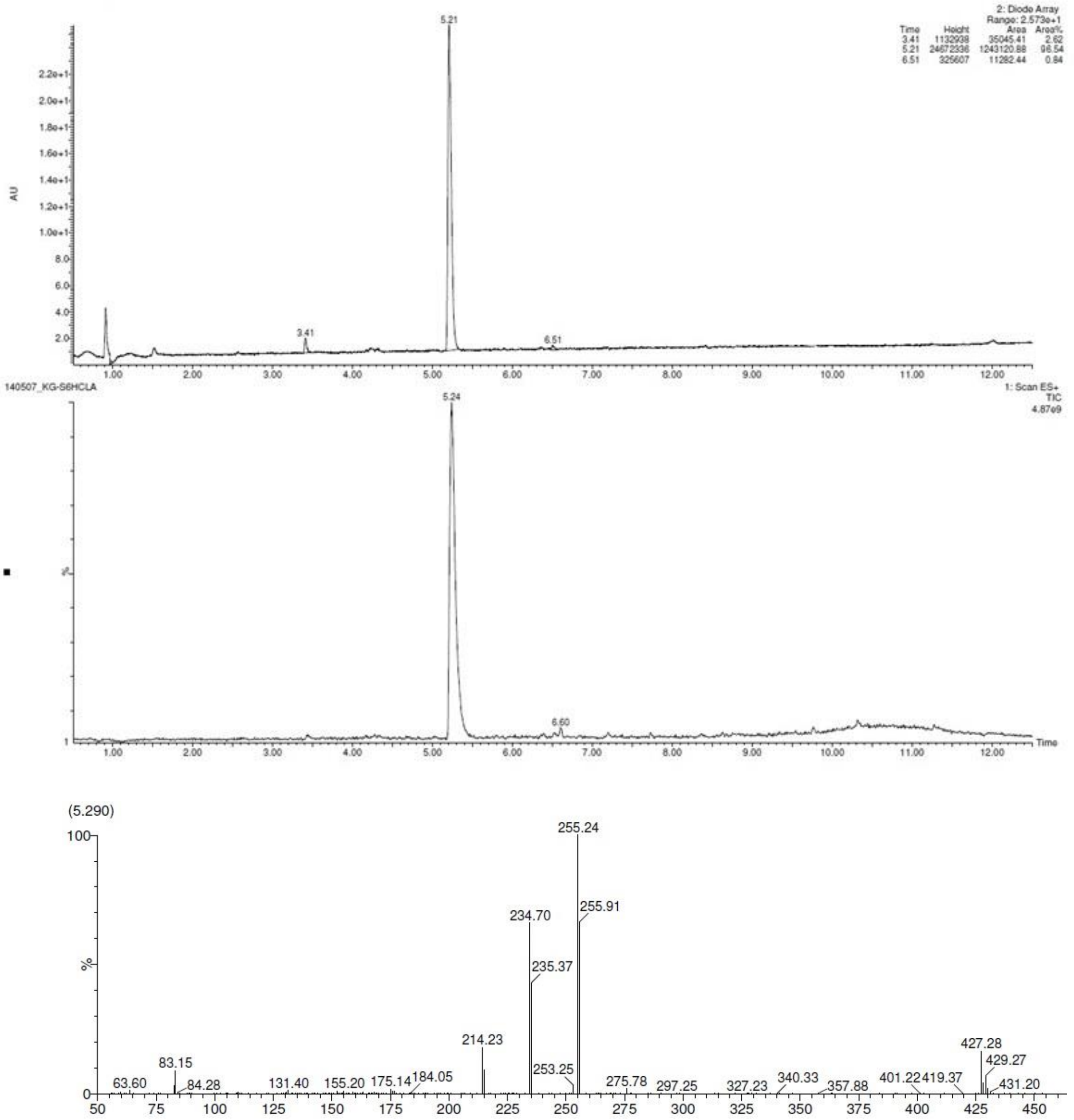

^{1}H NMR

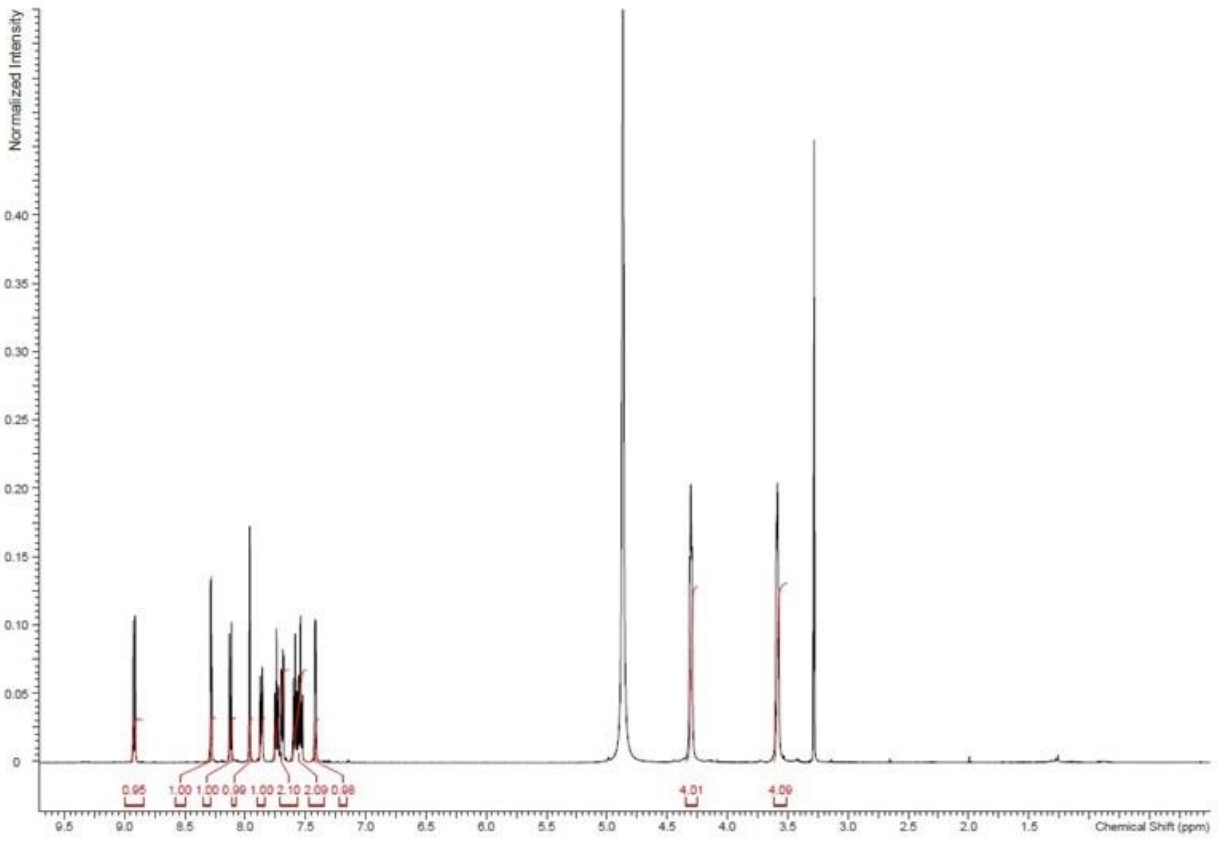

^{13}C NMR

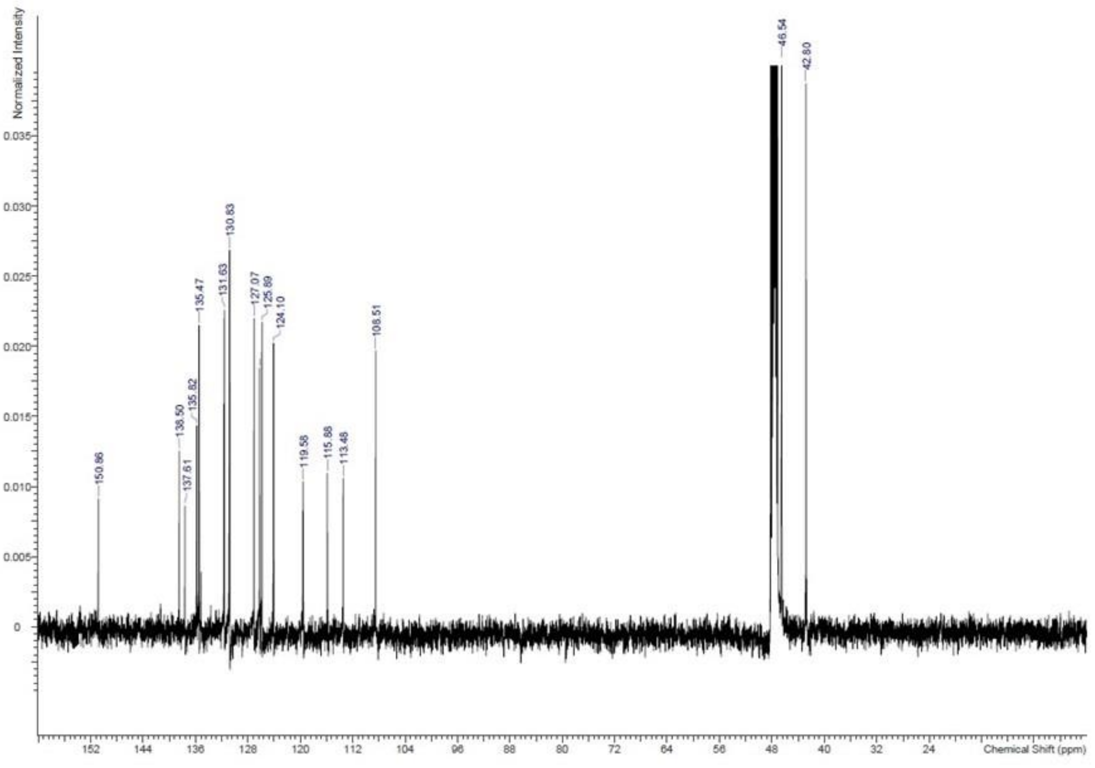

1-[(3-Fluorophenyl)sulfonyl]-4-(piperazin-1-yl)-1H-pyrrolo[3,2-c]quinoline dihydrochloride (**17**) **FPPQ**

UPLC-MS

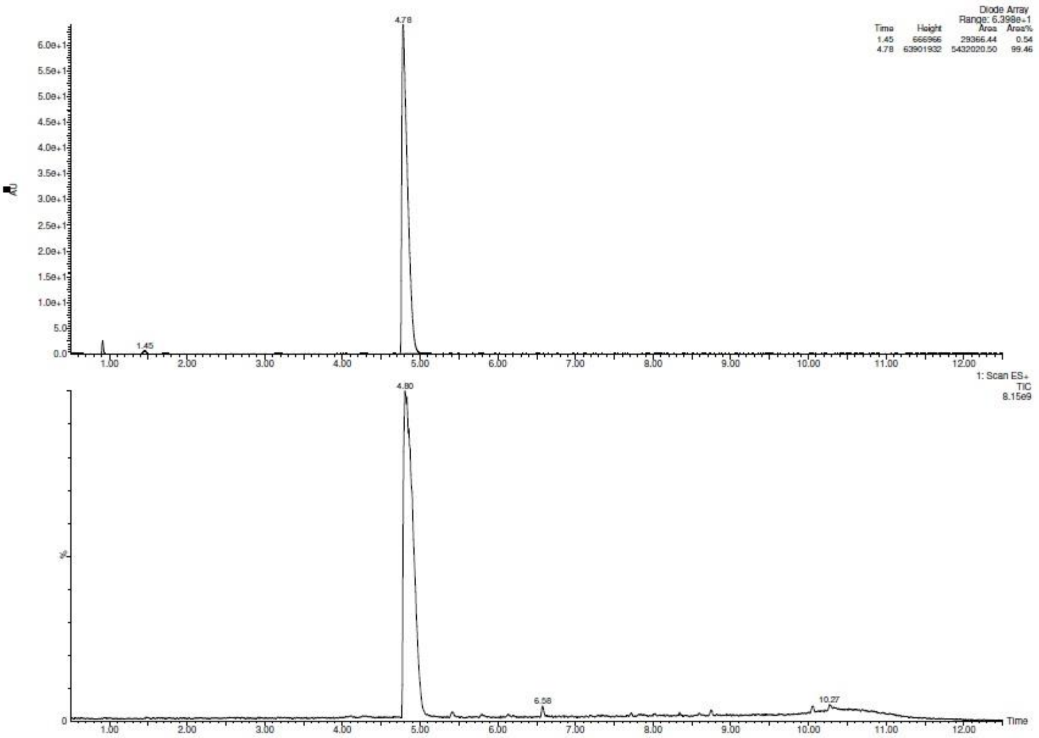

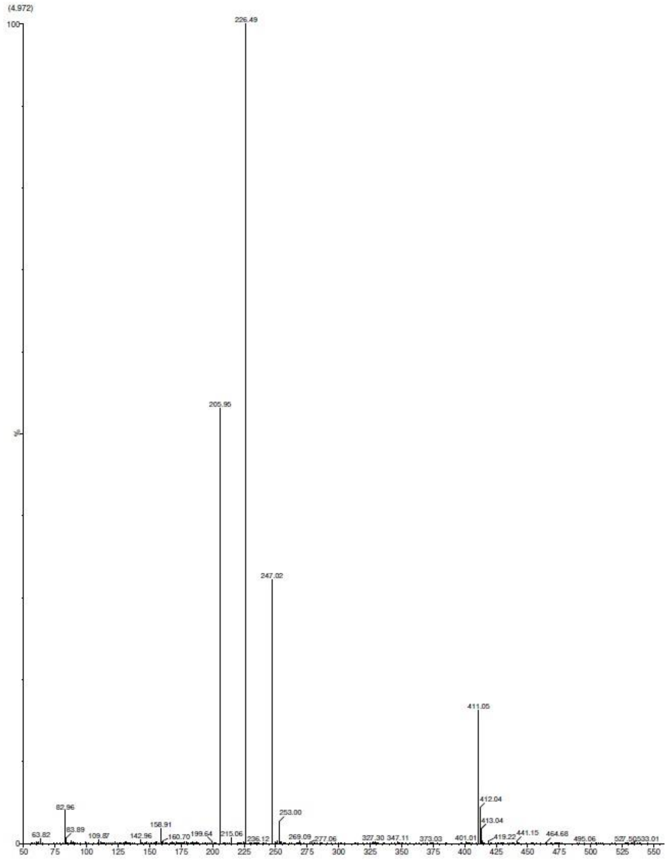

^{1}H NMR

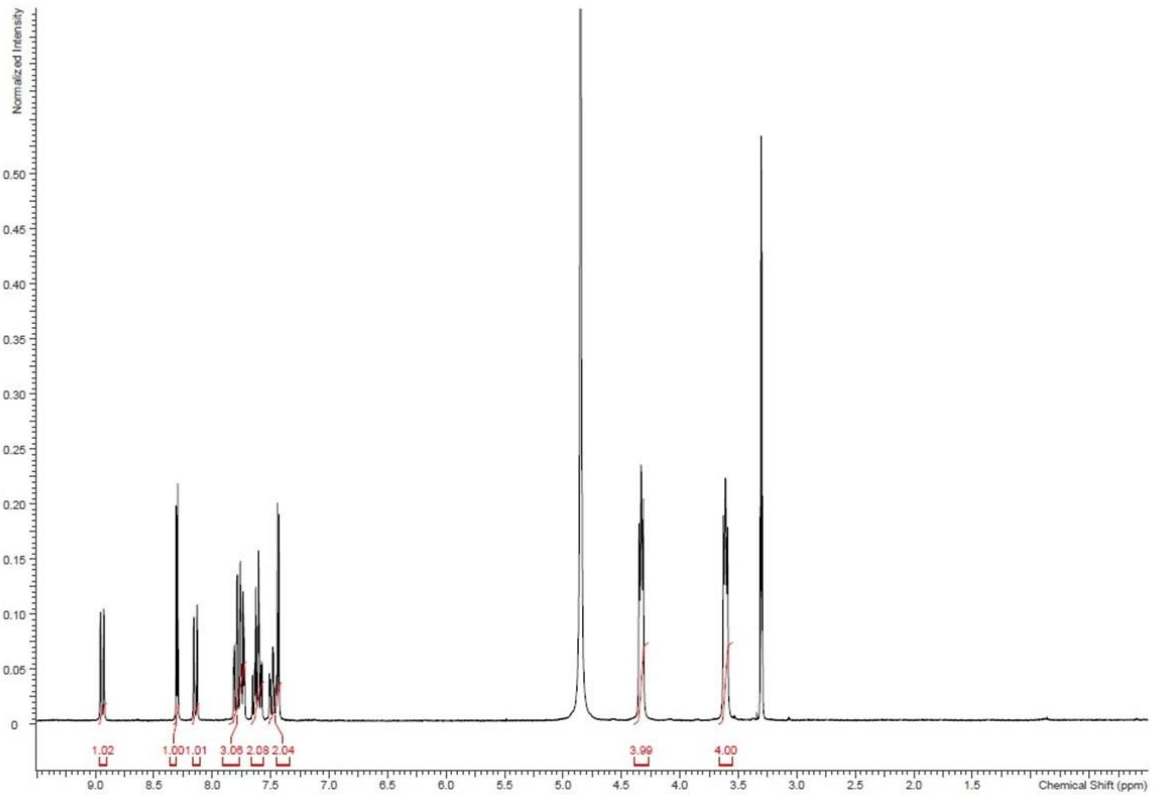

^{13}C NMR

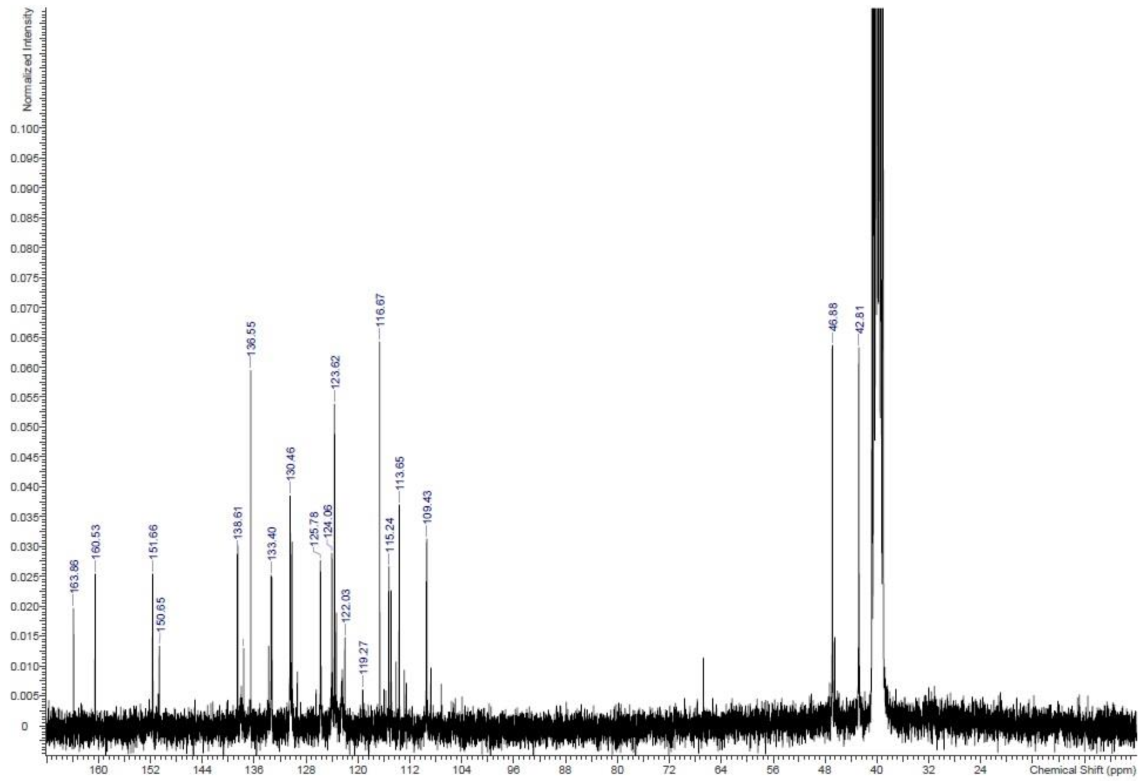

1-[(3-Chlorophenyl)sulfonyl]-4-(piperazin-1-yl)-1H-pyrrolo[3,2-c]quinoline dihydrochloride (**18**)

UPLC-MS

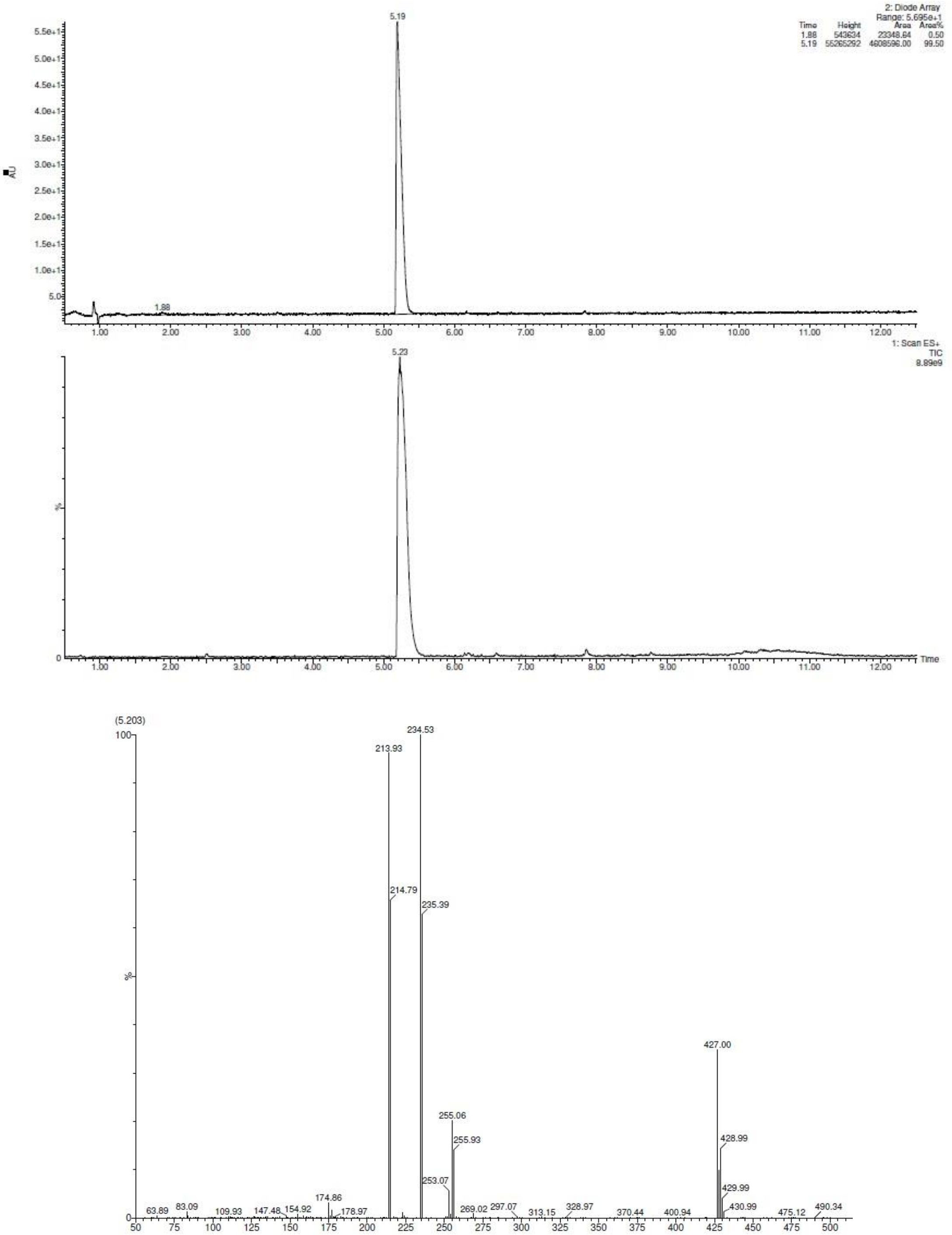

^{1}H NMR

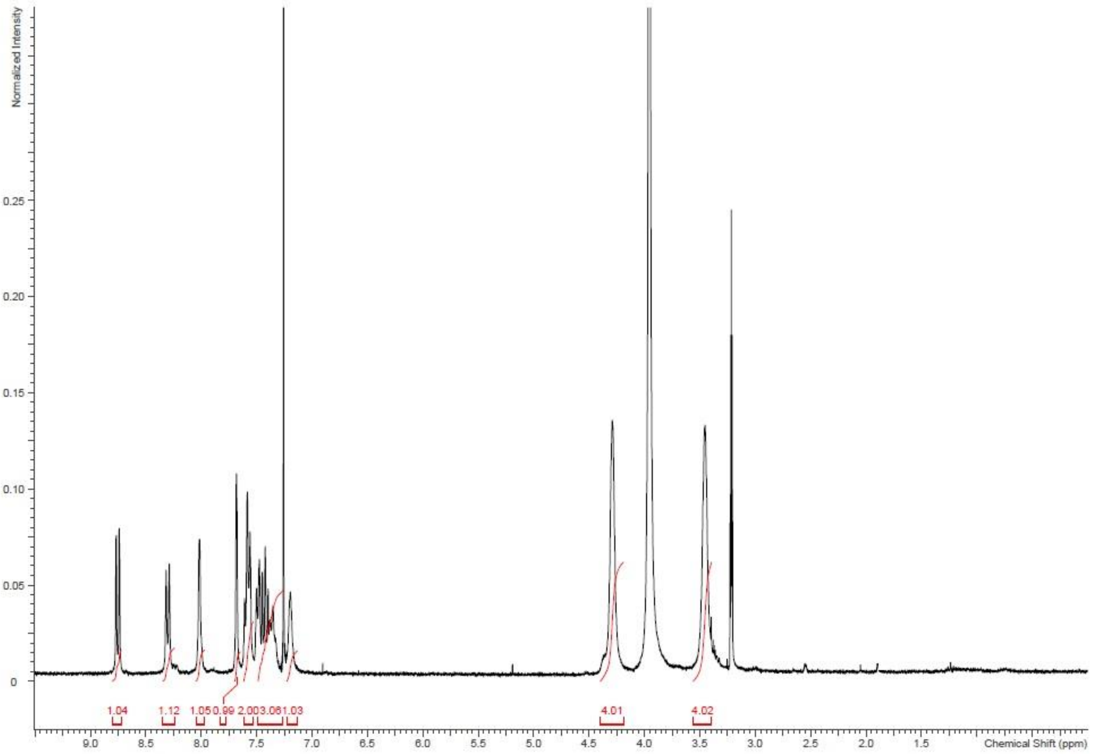

^{13}C NMR

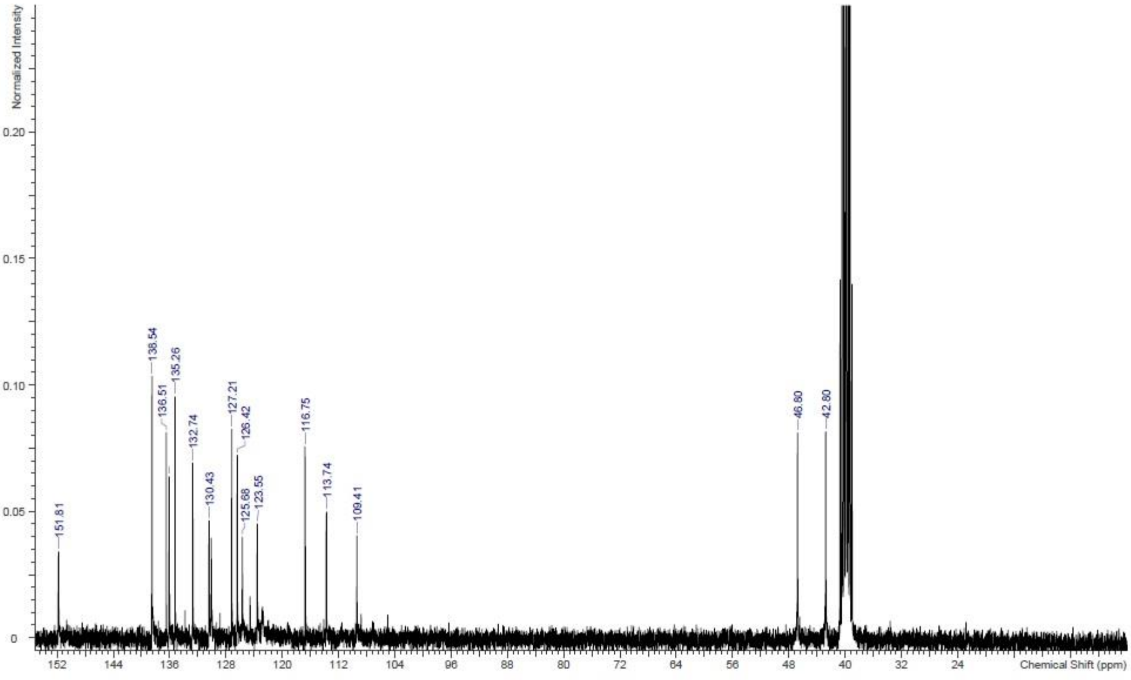

1-{[3-(Trifluoromethyl)phenyl]sulfonyl}-4-(piperazin-1-yl)-1H-pyrrolo[3,2-c]quinoline dihydrochloride (**19**)

UPLC-MS

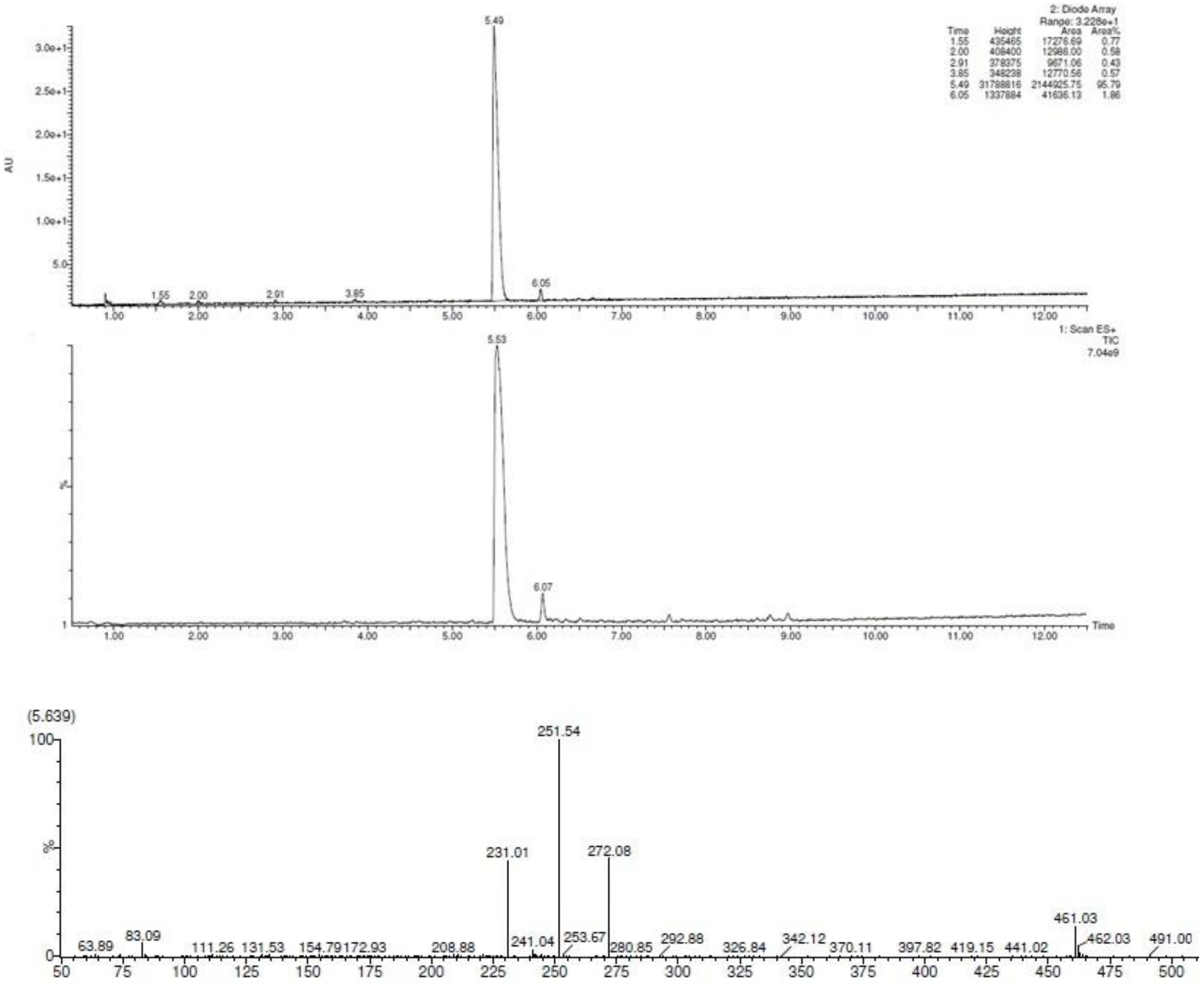

^{1}H NMR

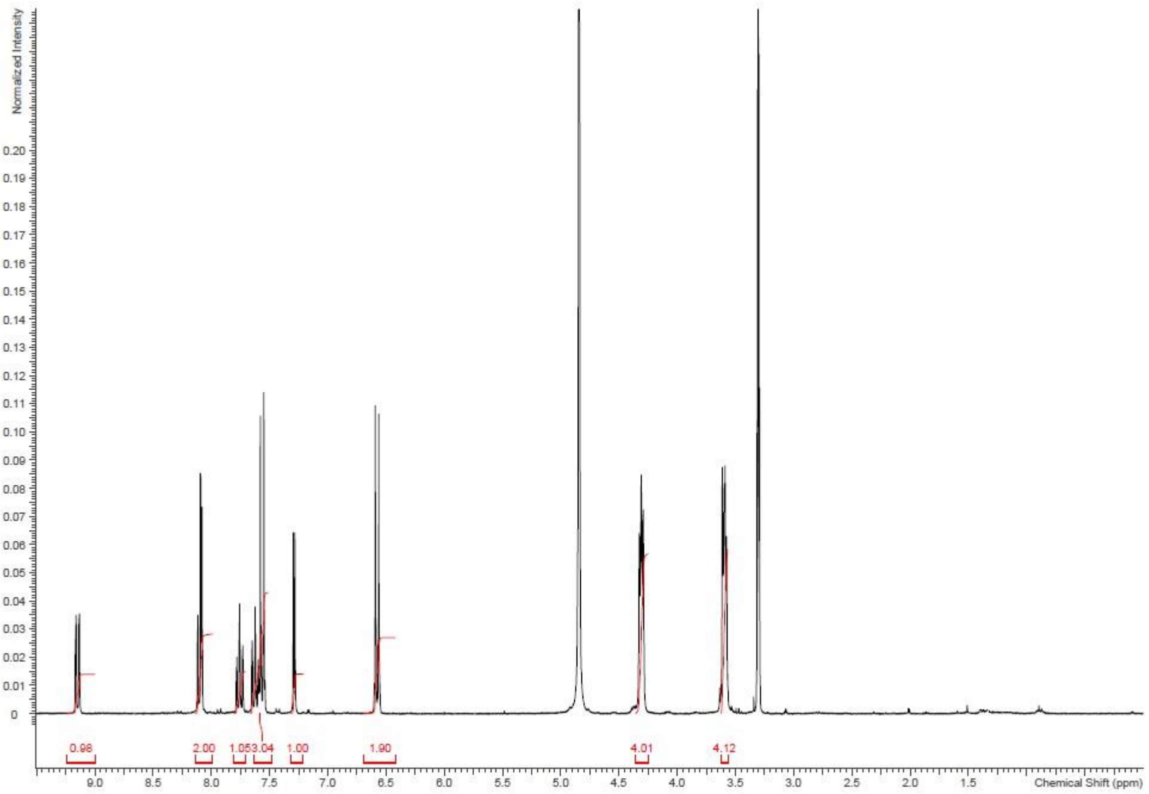

^{13}C NMR

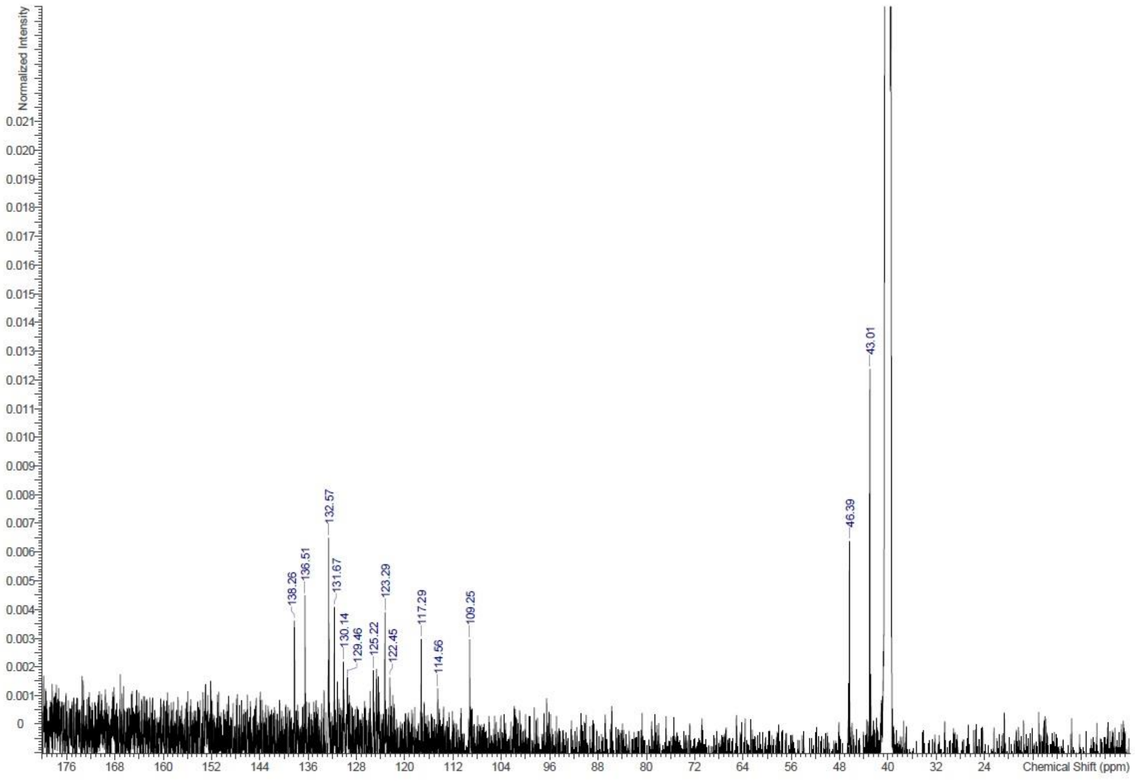

1-[(3-Methylphenyl)sulfonyl]-4-(piperazin-1-yl)-1H-pyrrolo[3,2-c]quinoline dihydrochloride (**20**)

UPLC-MS

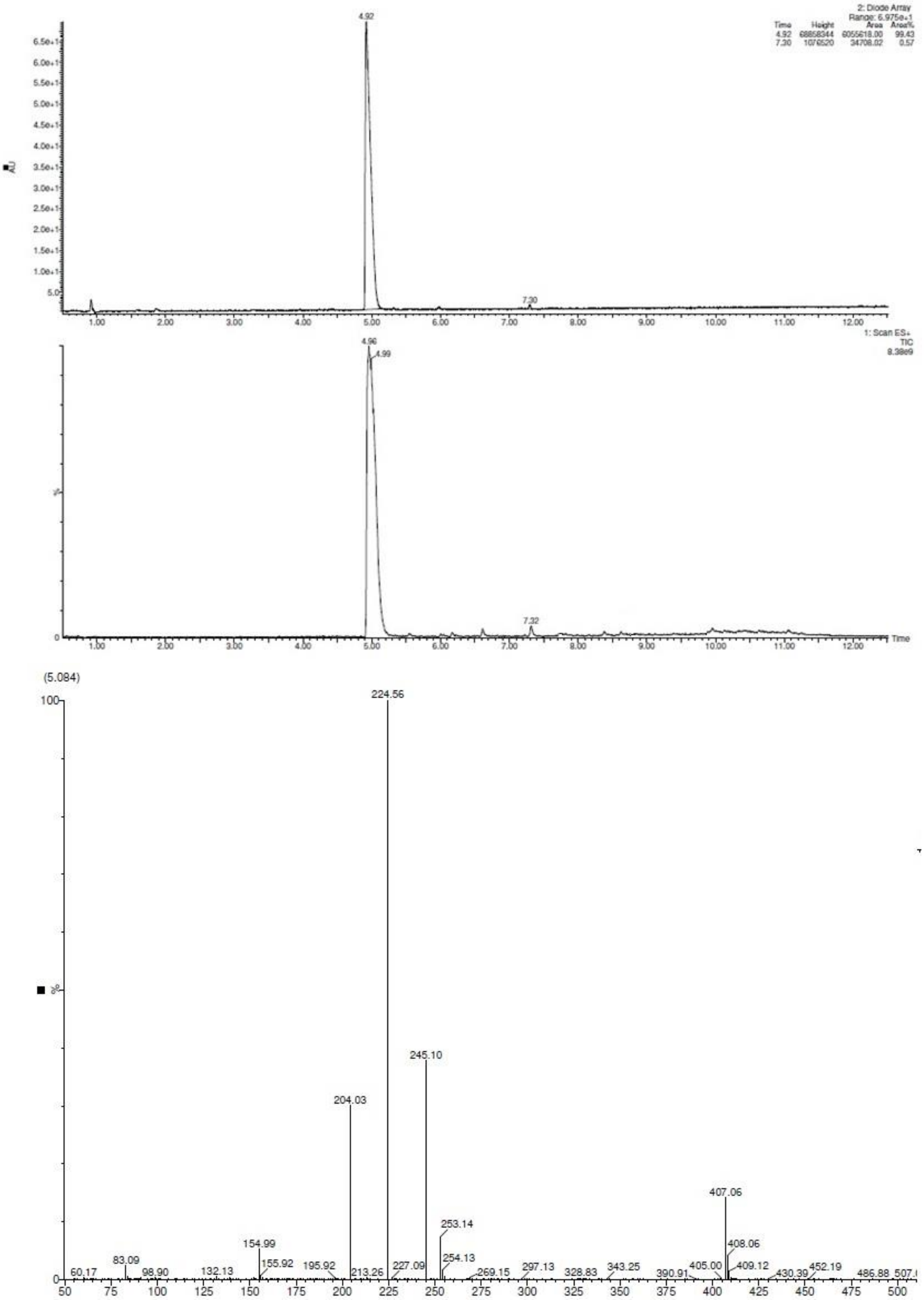

^1H NMR

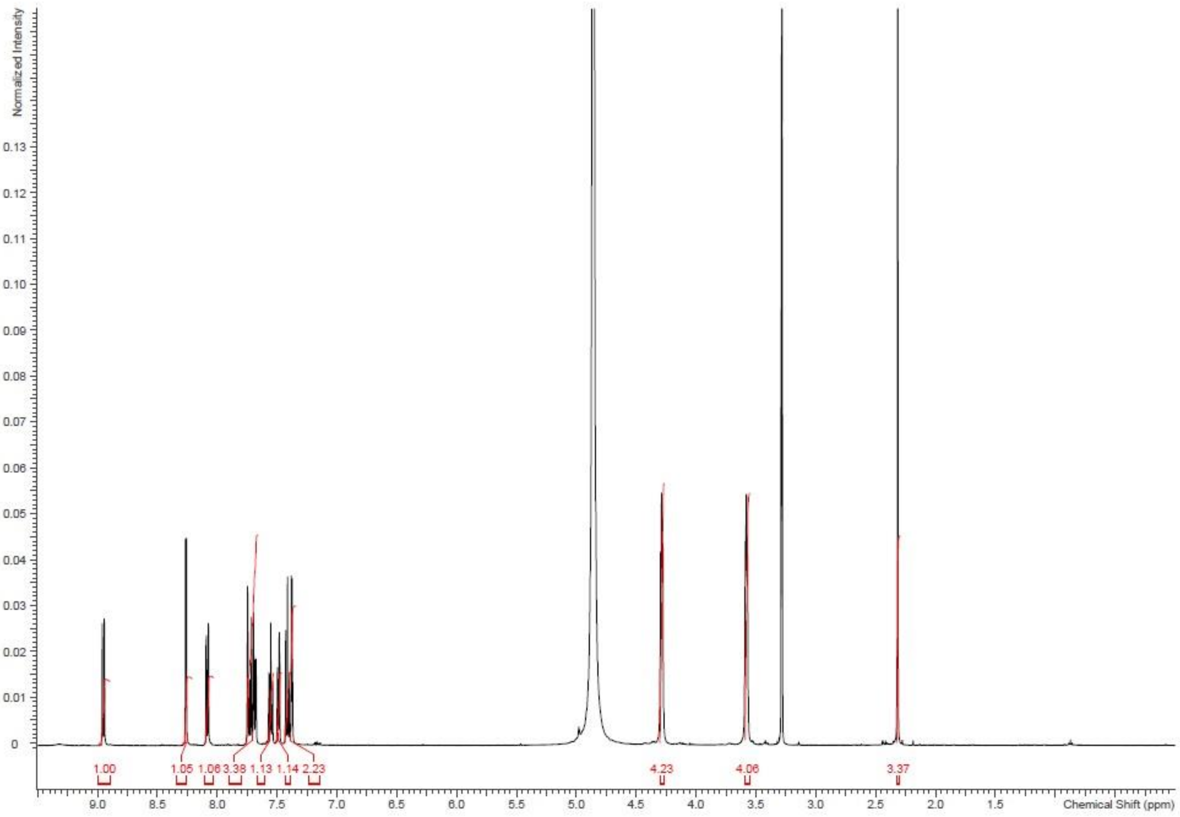

^{13}C NMR

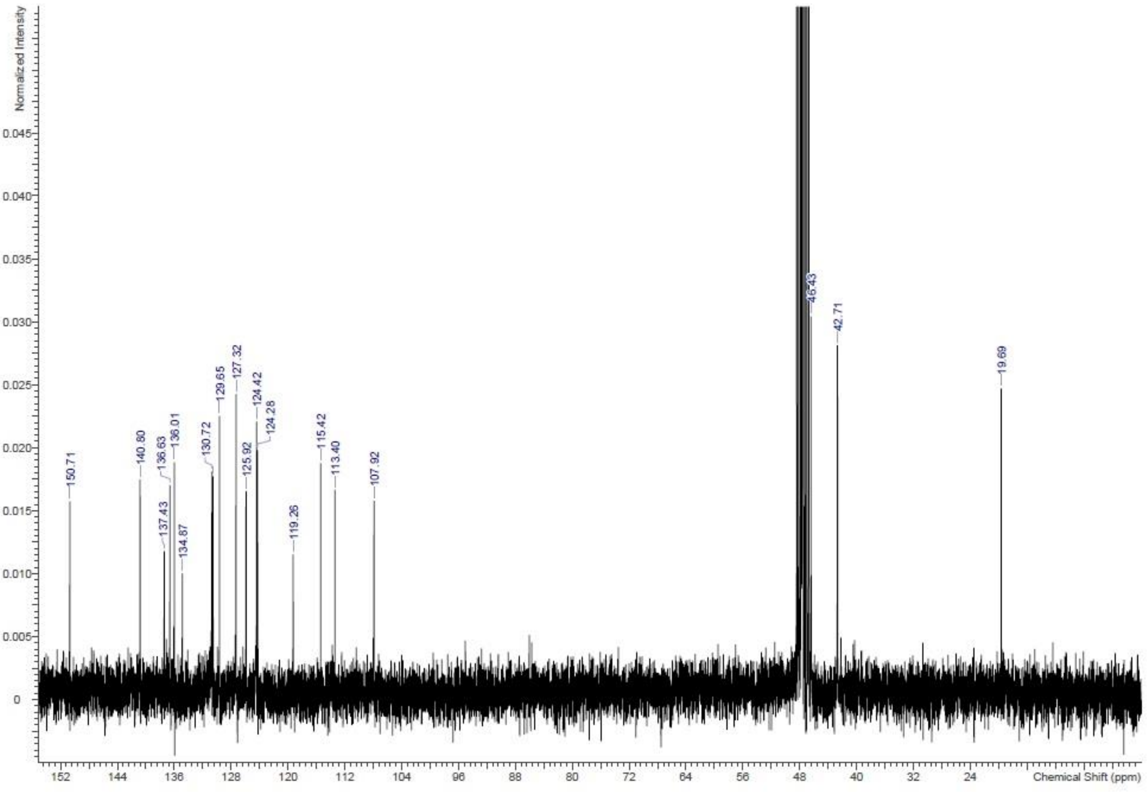

1-[(3-Methoxyphenyl)sulfonyl]-4-(piperazin-1-yl)-1H-pyrrolo[3,2-c]quinoline dihydrochloride (**21**)

UPLC-MS

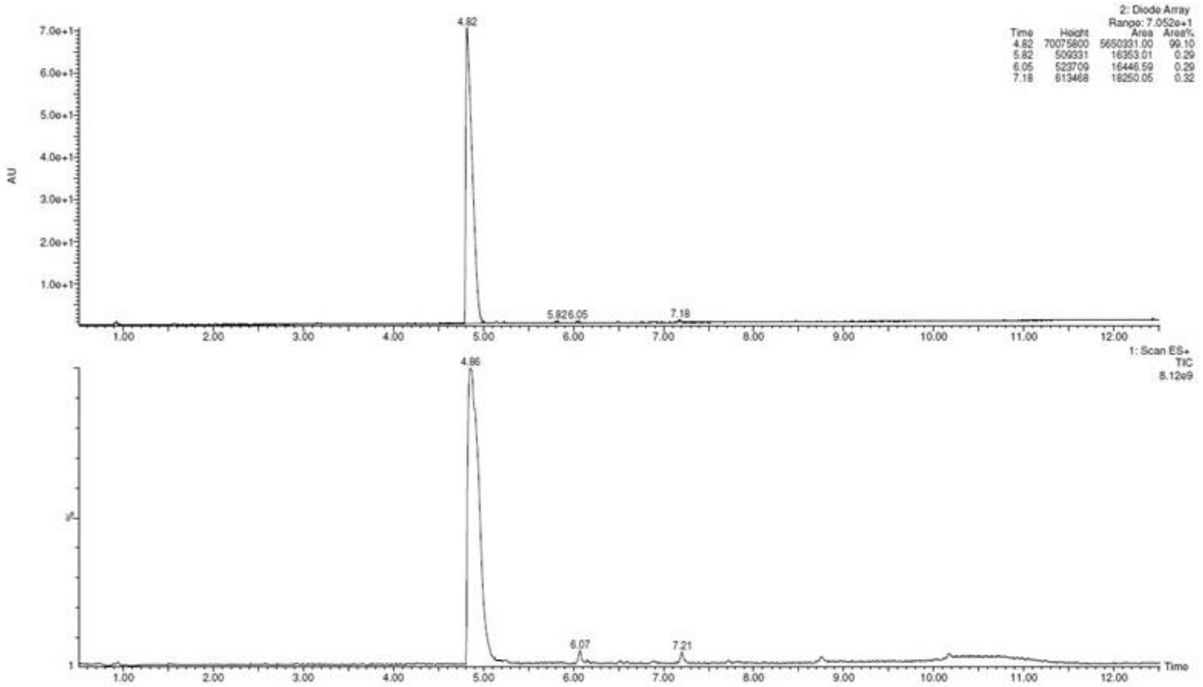

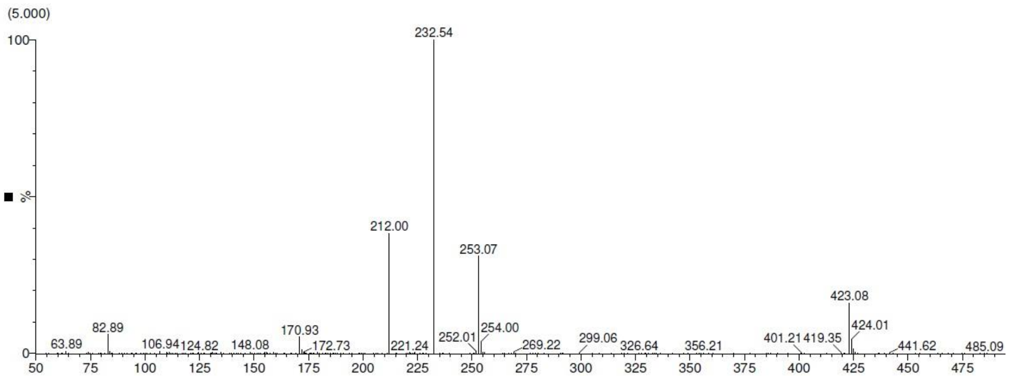

^{1}H NMR

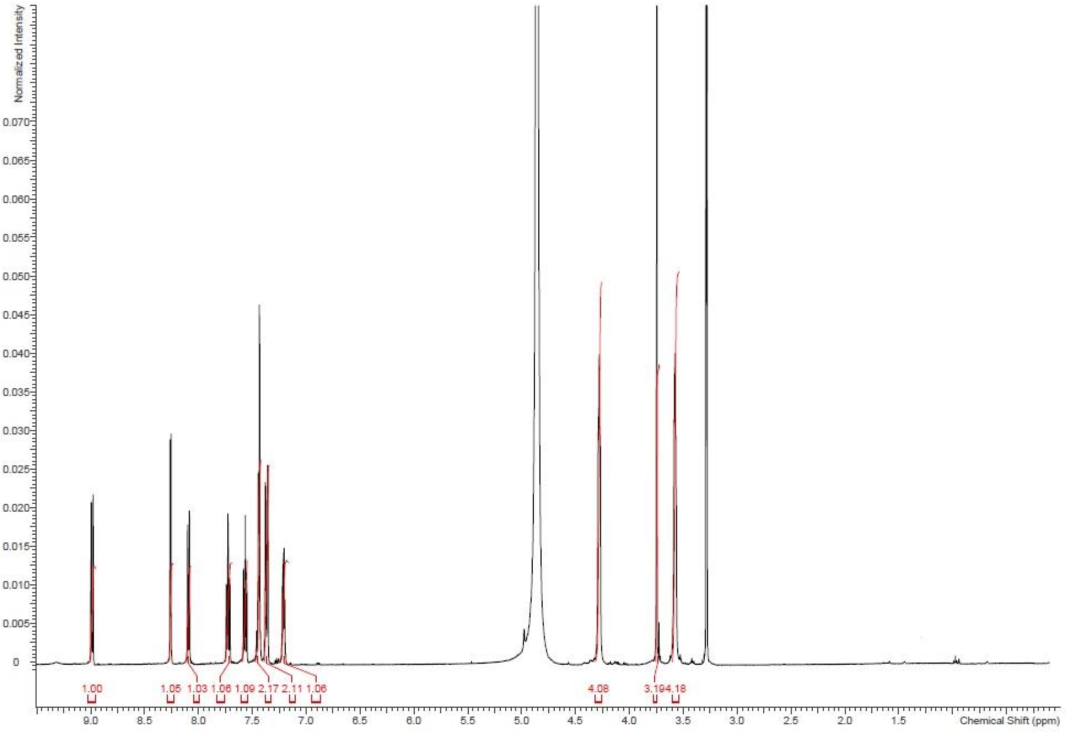

^{13}C NMR

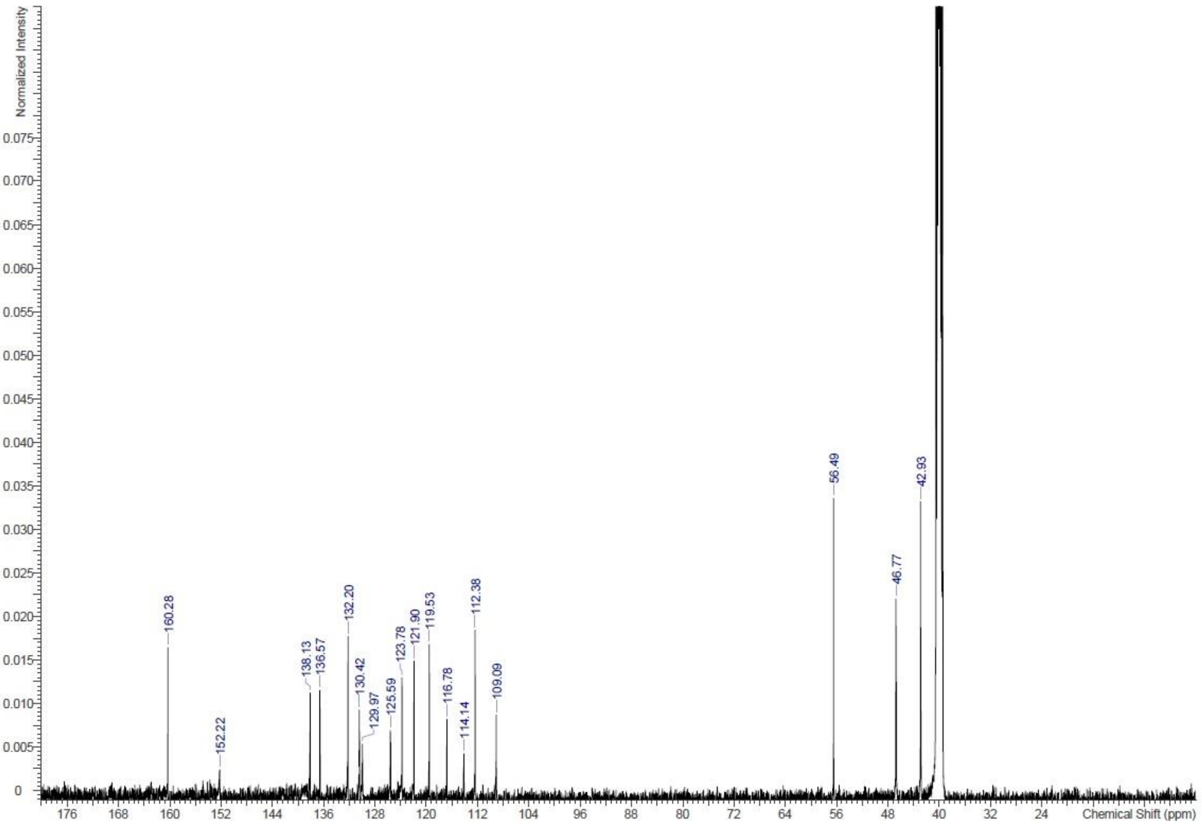

1-[(4-Fluorophenyl)sulfonyl]-4-(piperazin-1-yl)-1H-pyrrolo[3,2-c]quinoline dihydrochloride (**22**)

UPLC-MS

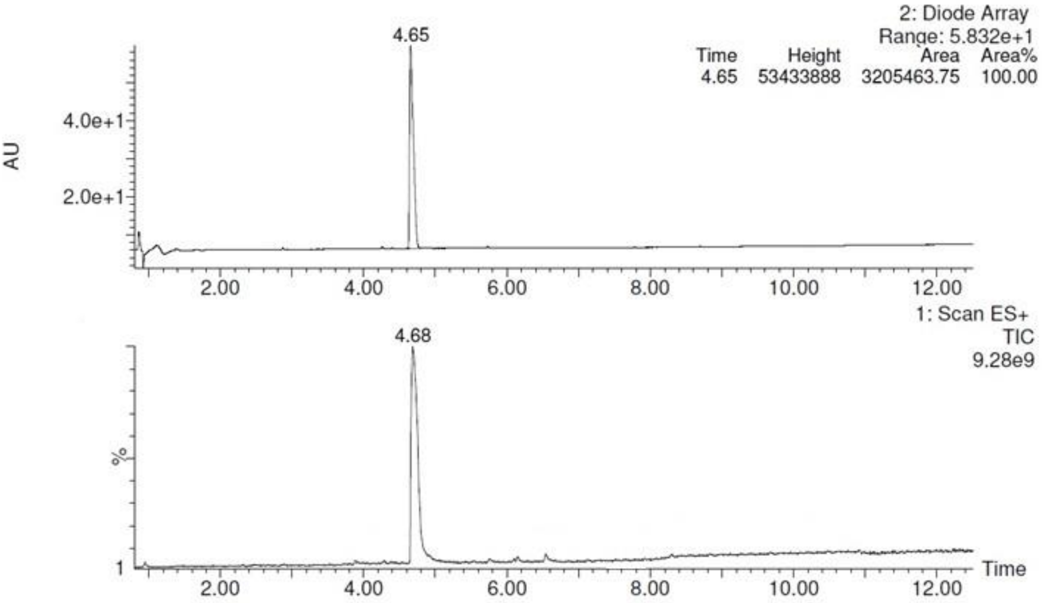

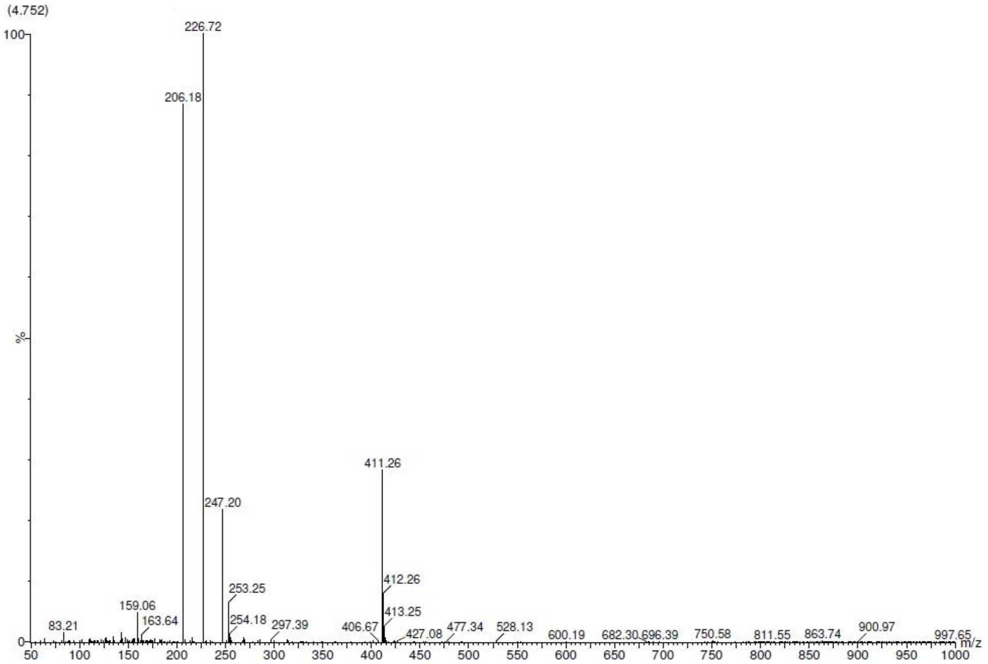

^{1}H NMR

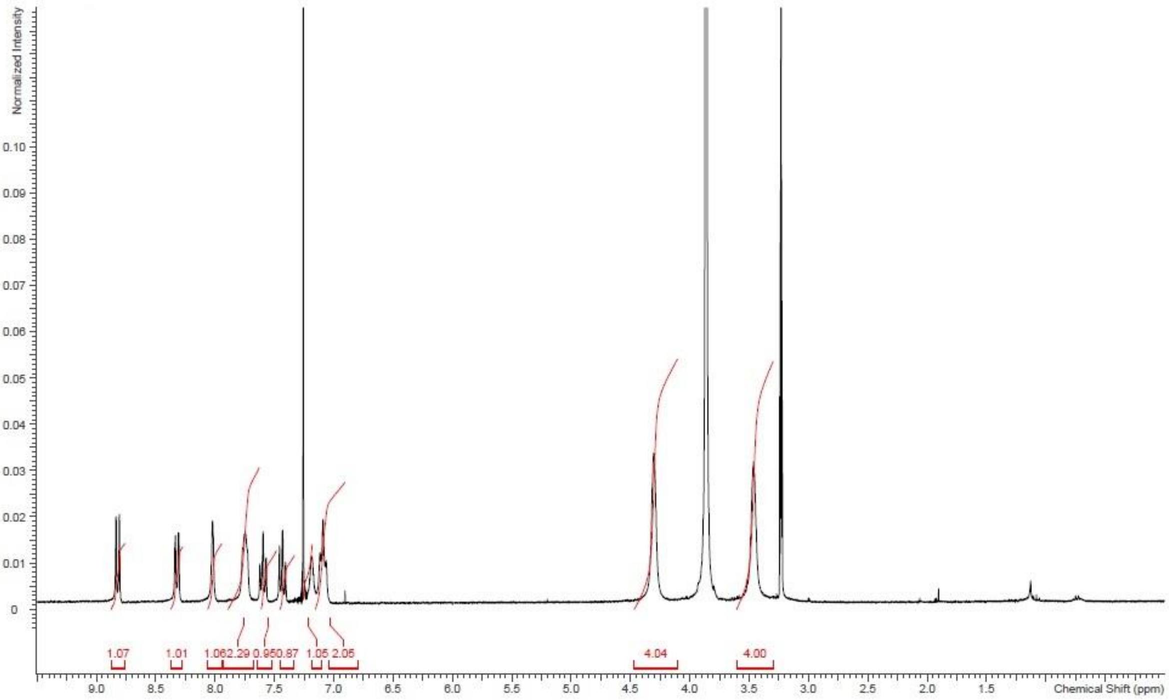

^{13}C NMR

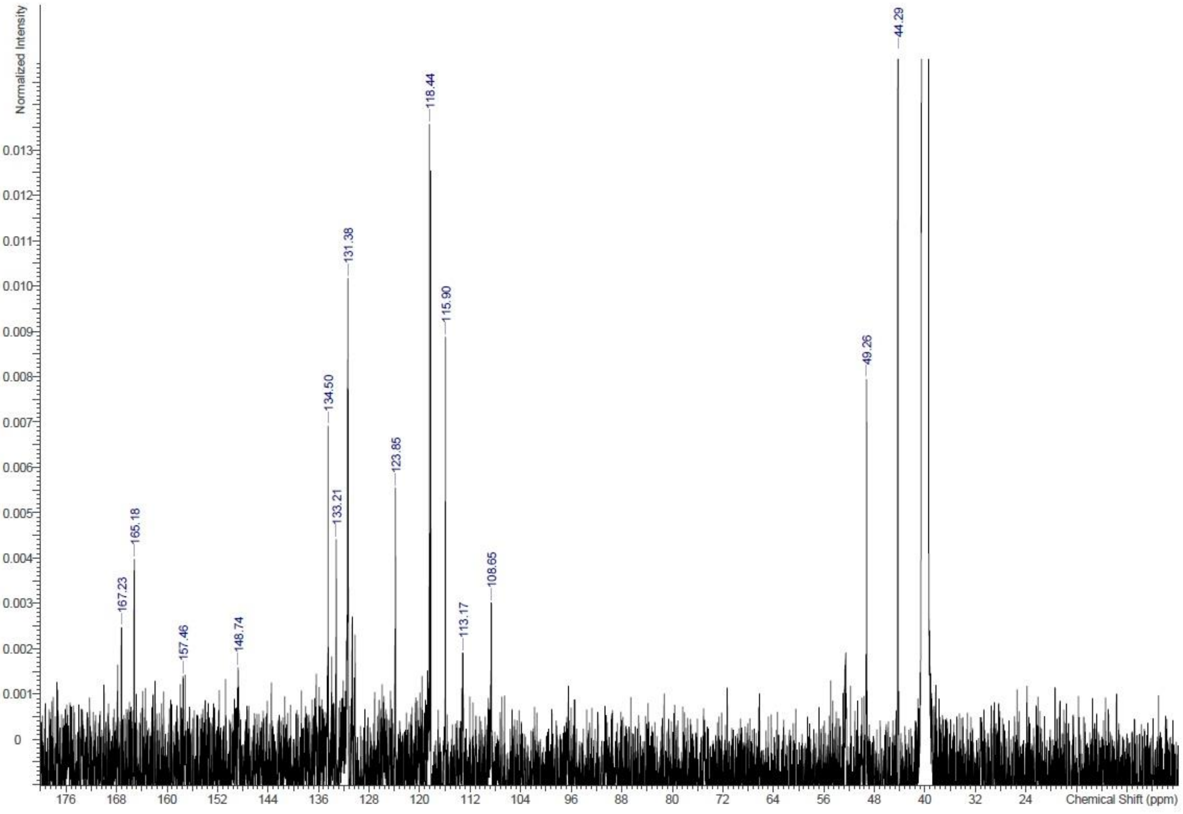

1-{[4-(Trifluoromethyl)phenyl]sulfonyl}-4-(piperazin-1-yl)-1H-pyrrolo[3,2-c]quinoline dihydrochloride (**23**)

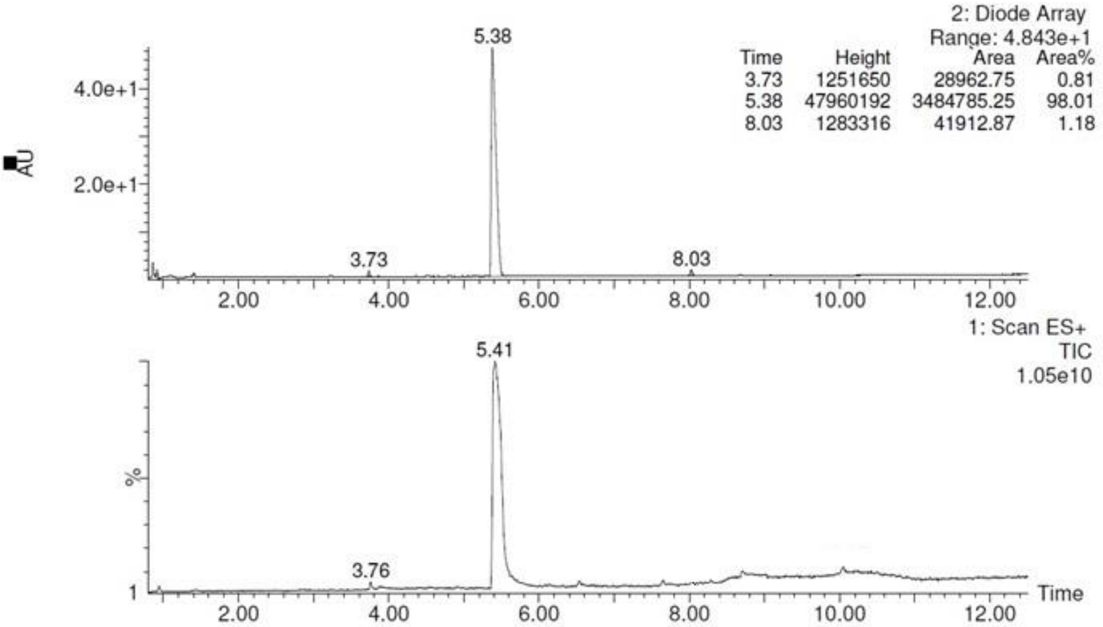

UPLC-MS

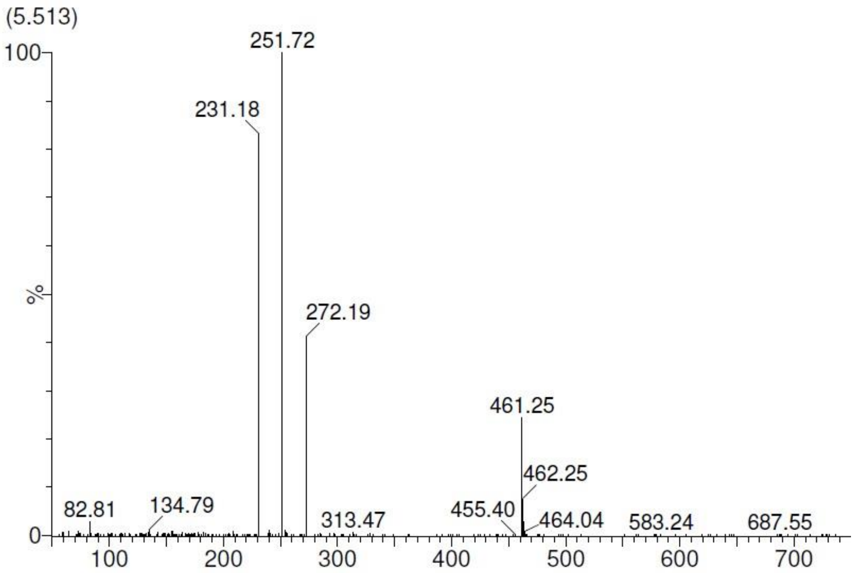

^{1}H NMR

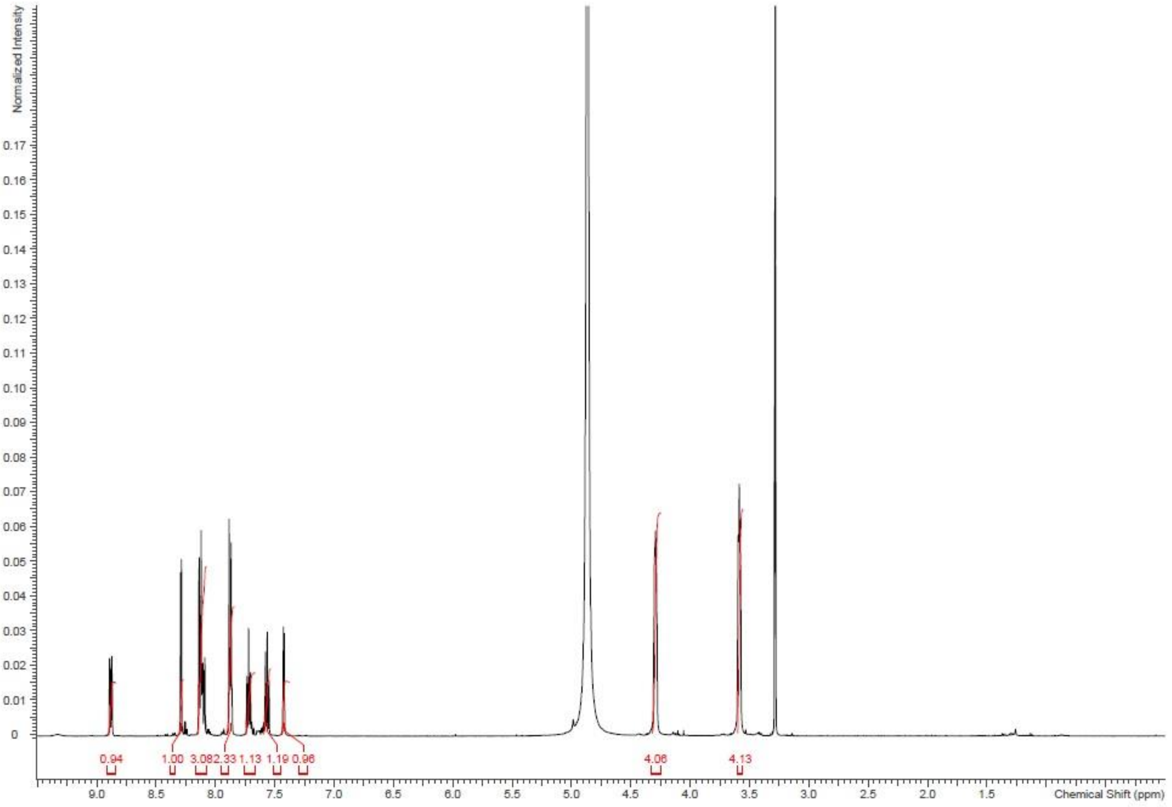

^{13}C NMR

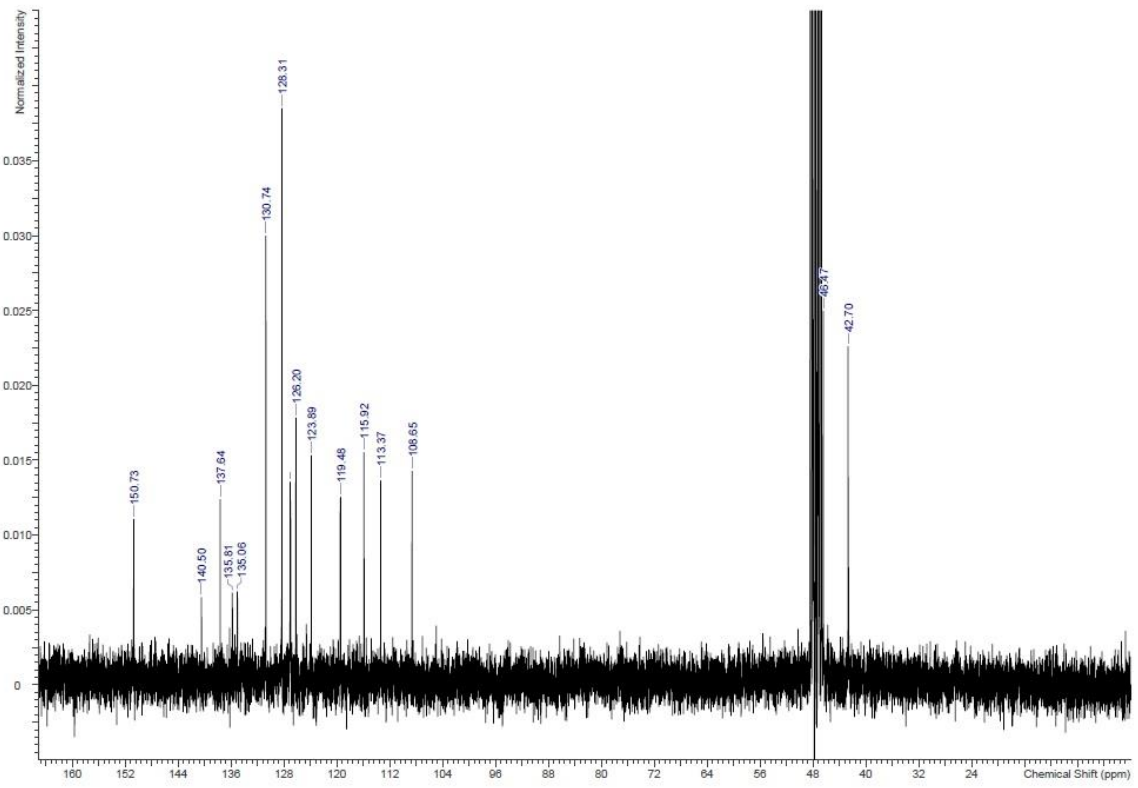

1-[(4-iso-Propylphenyl)sulfonyl]-4-(piperazin-1-yl)-1H-pyrrolo[3,2-c]quinoline dihydrochloride (**24**)

UPLC-MS

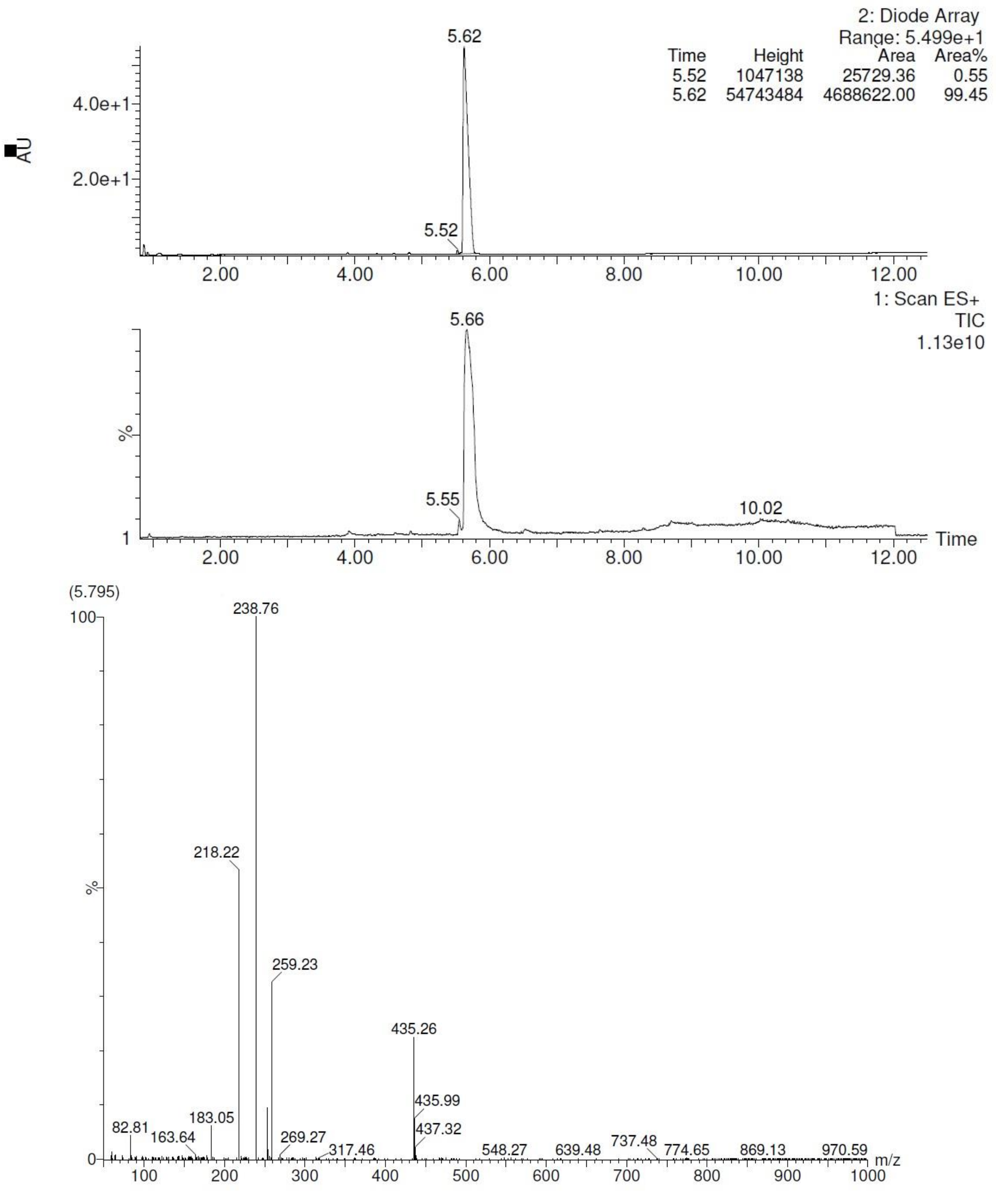

^1H NMR

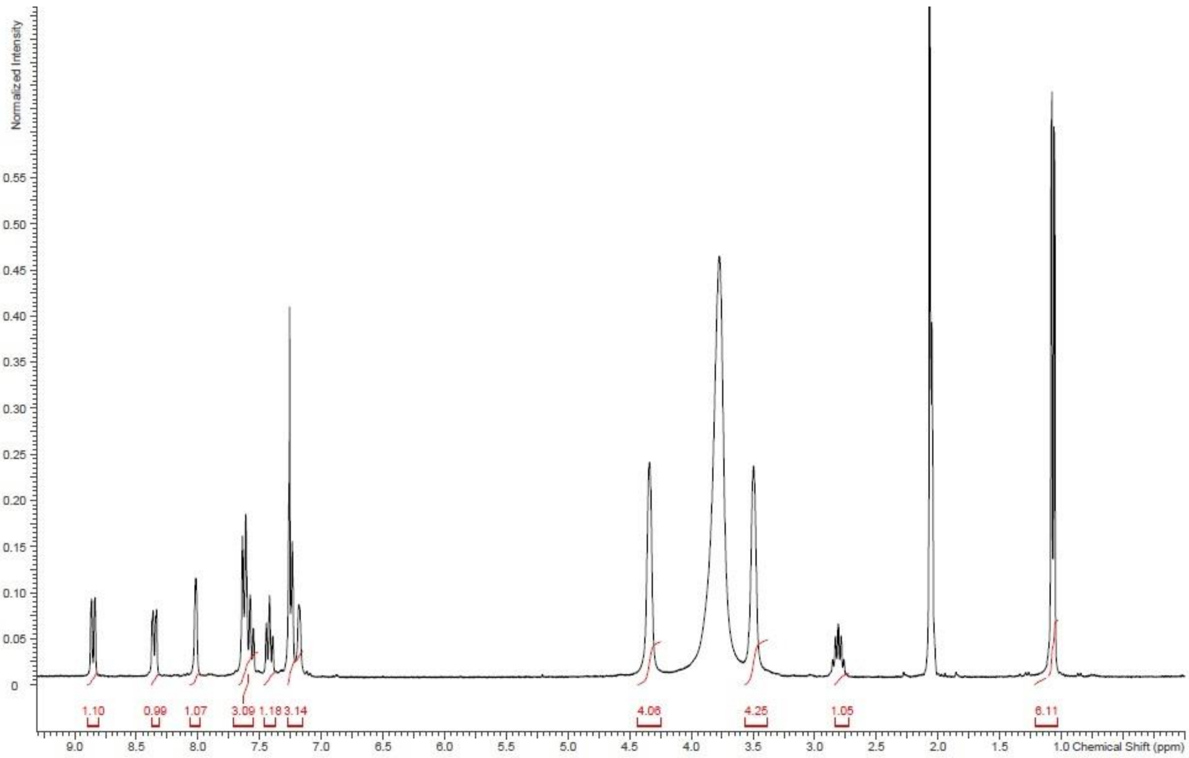

^{13}C NMR

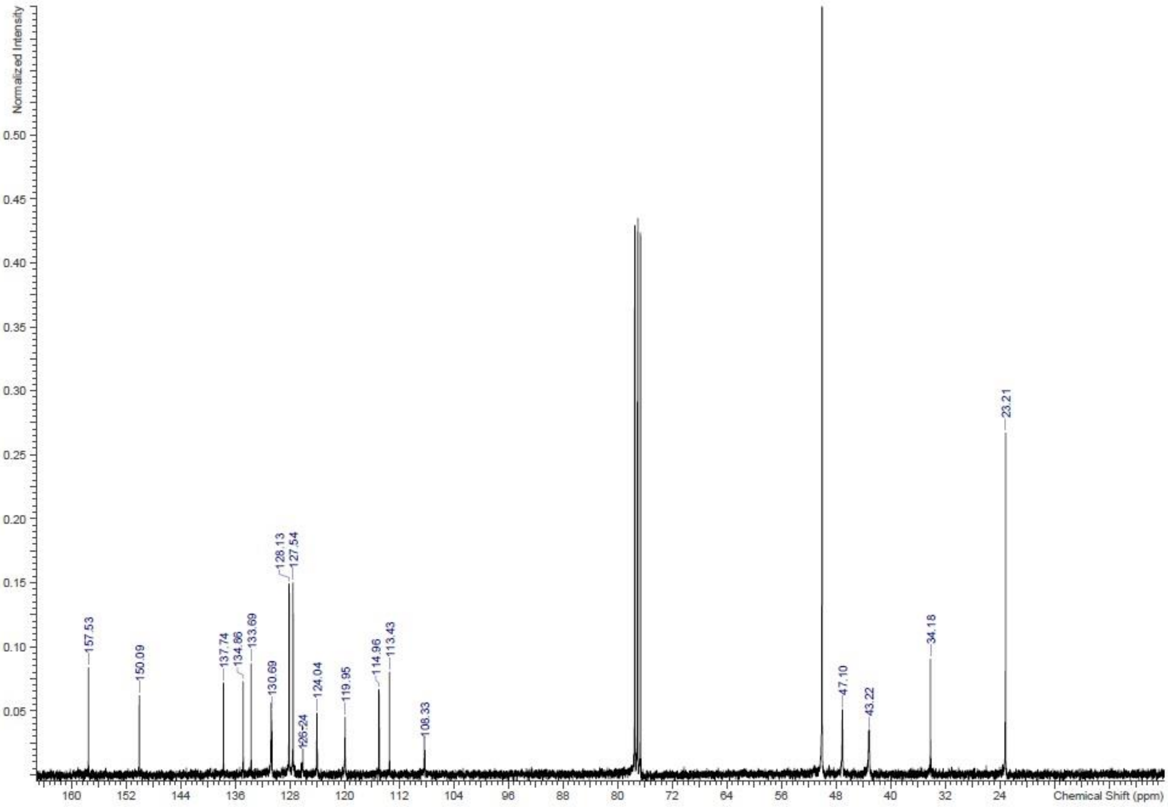

1-[(3,4-Difluorophenyl)sulfonyl]-4-(piperazin-1-yl)-1H-pyrrolo[3,2-c]quinoline dihydrochloride (**25**)

UPLC-MS

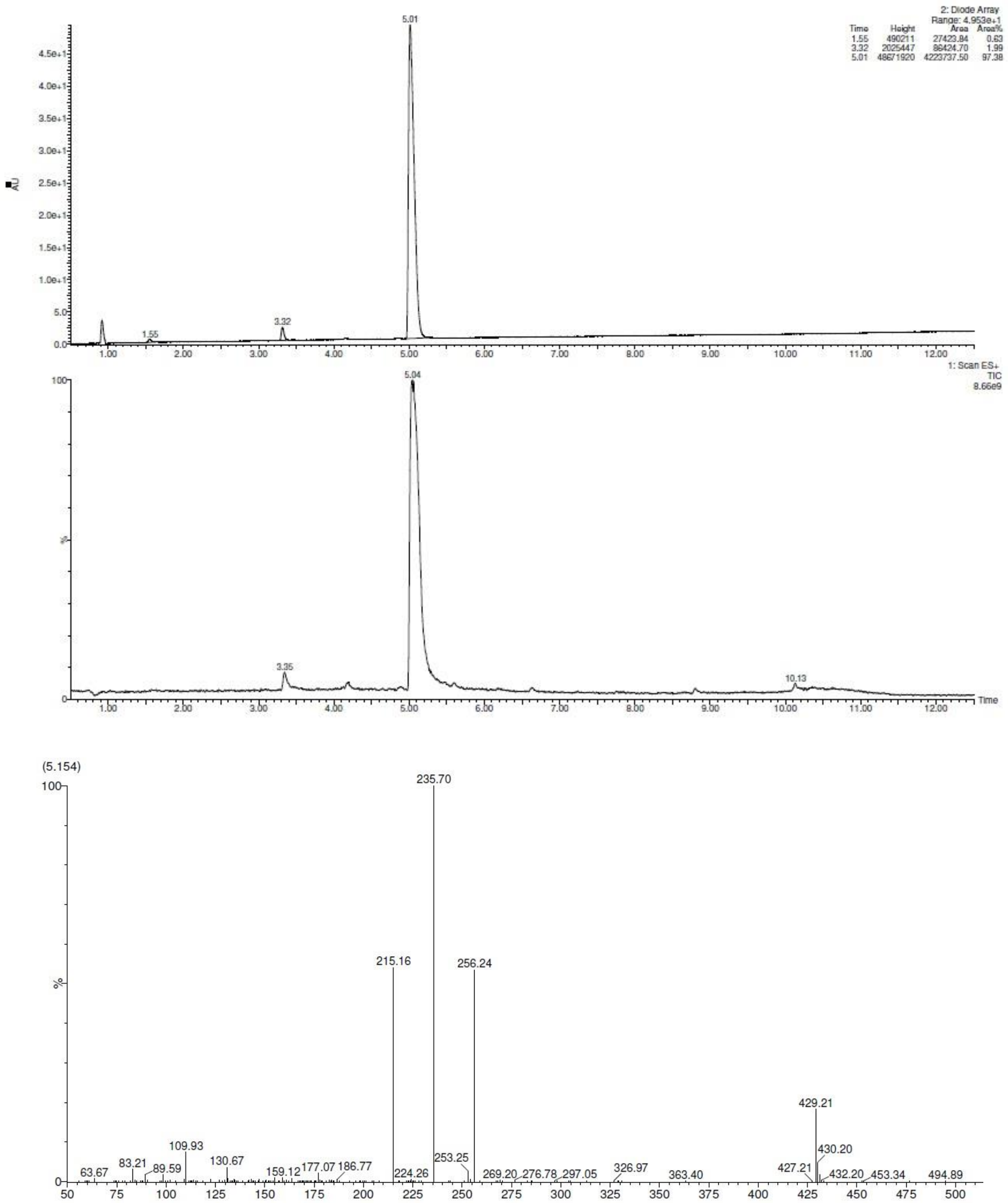

^{1}H NMR

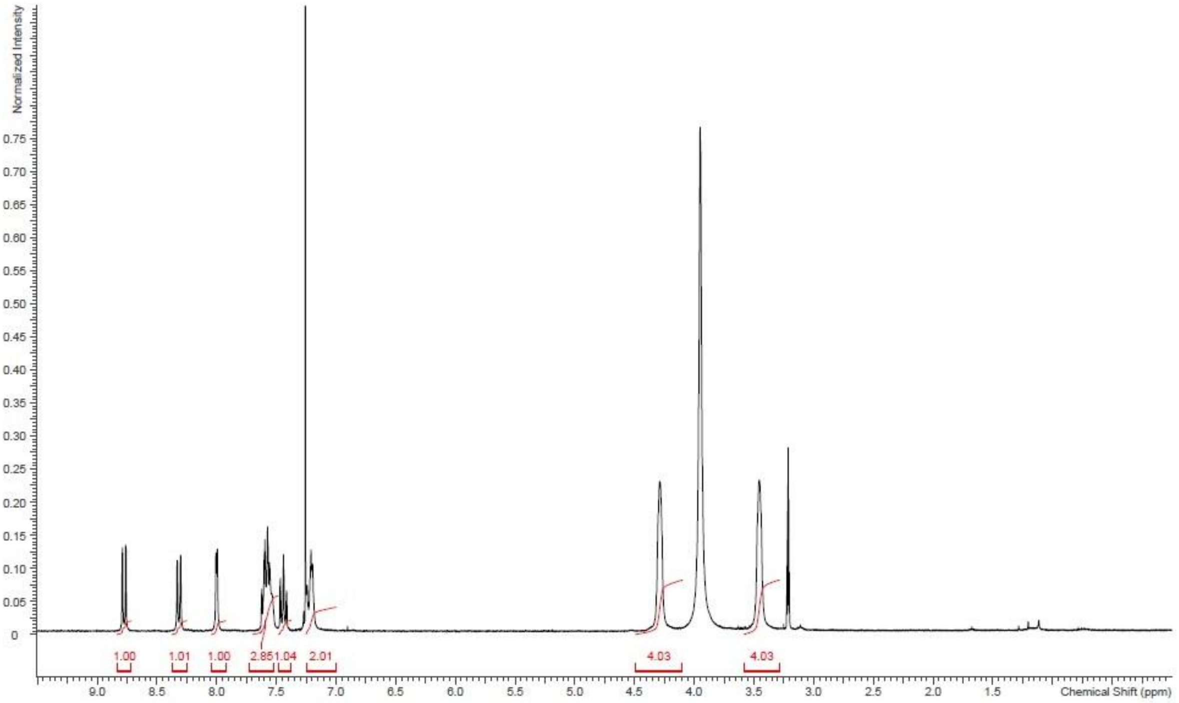

^{13}C NMR

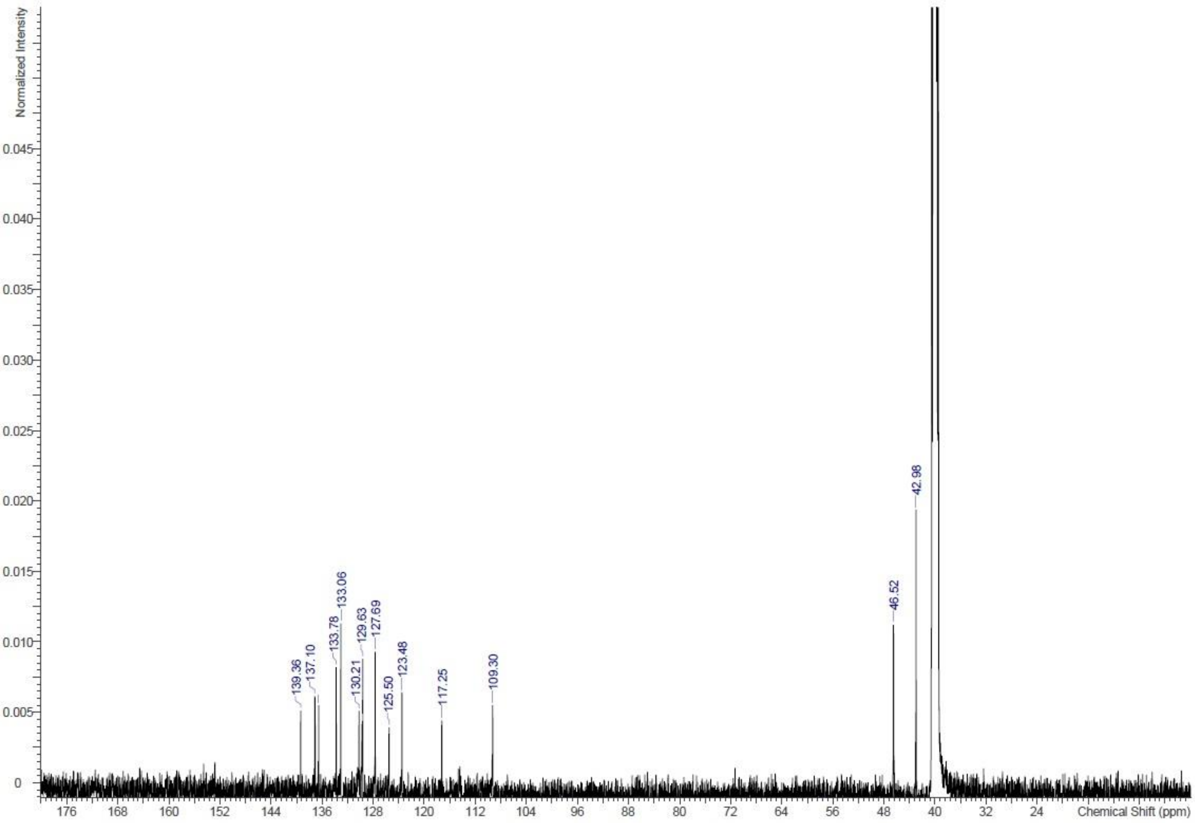

1-[(3,4-Dichlorophenyl)sulfonyl]-4-(piperazin-1-yl)-1H-pyrrolo[3,2-c]quinoline dihydrochloride (**26**)

UPLC-MS

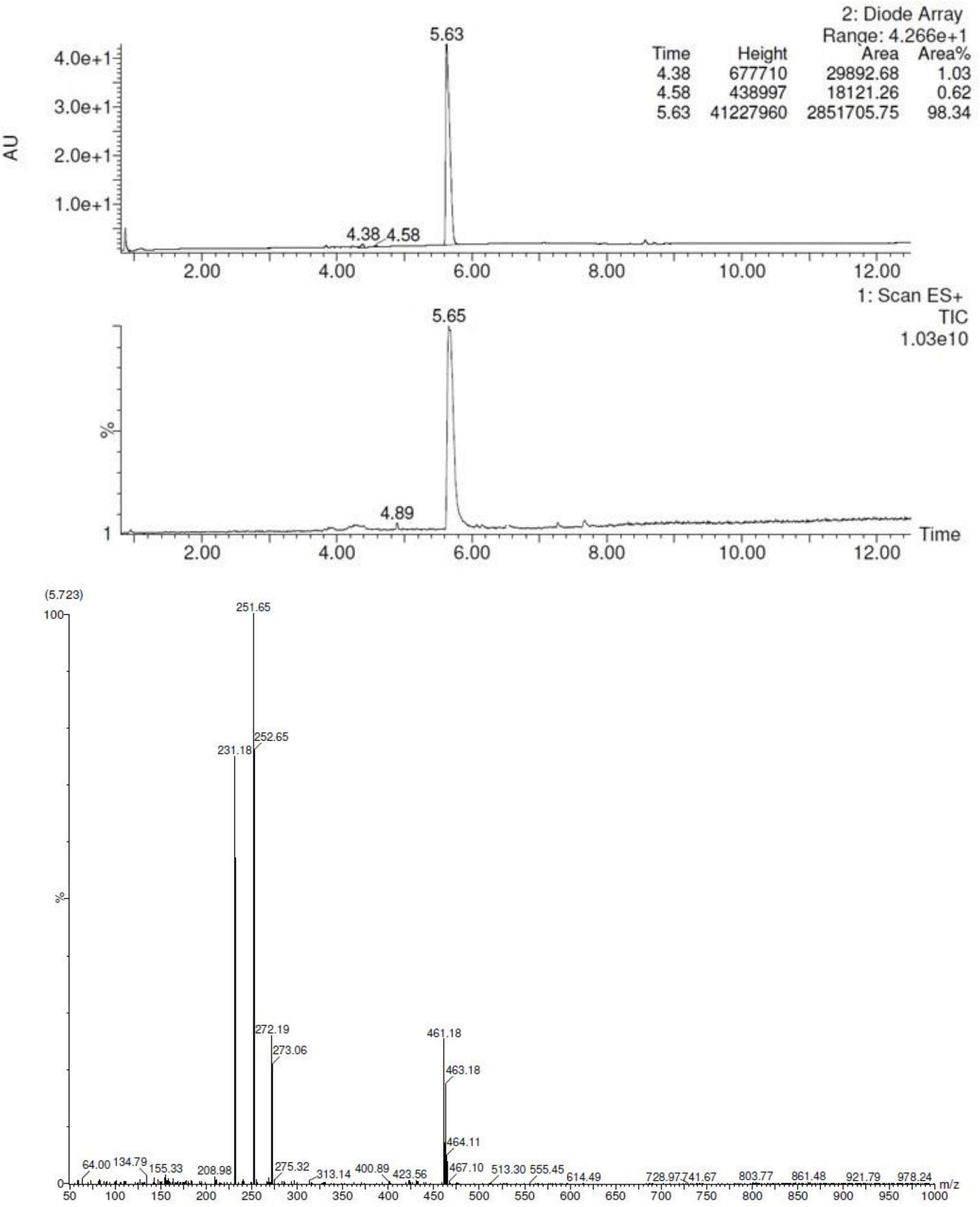

^{1}H NMR

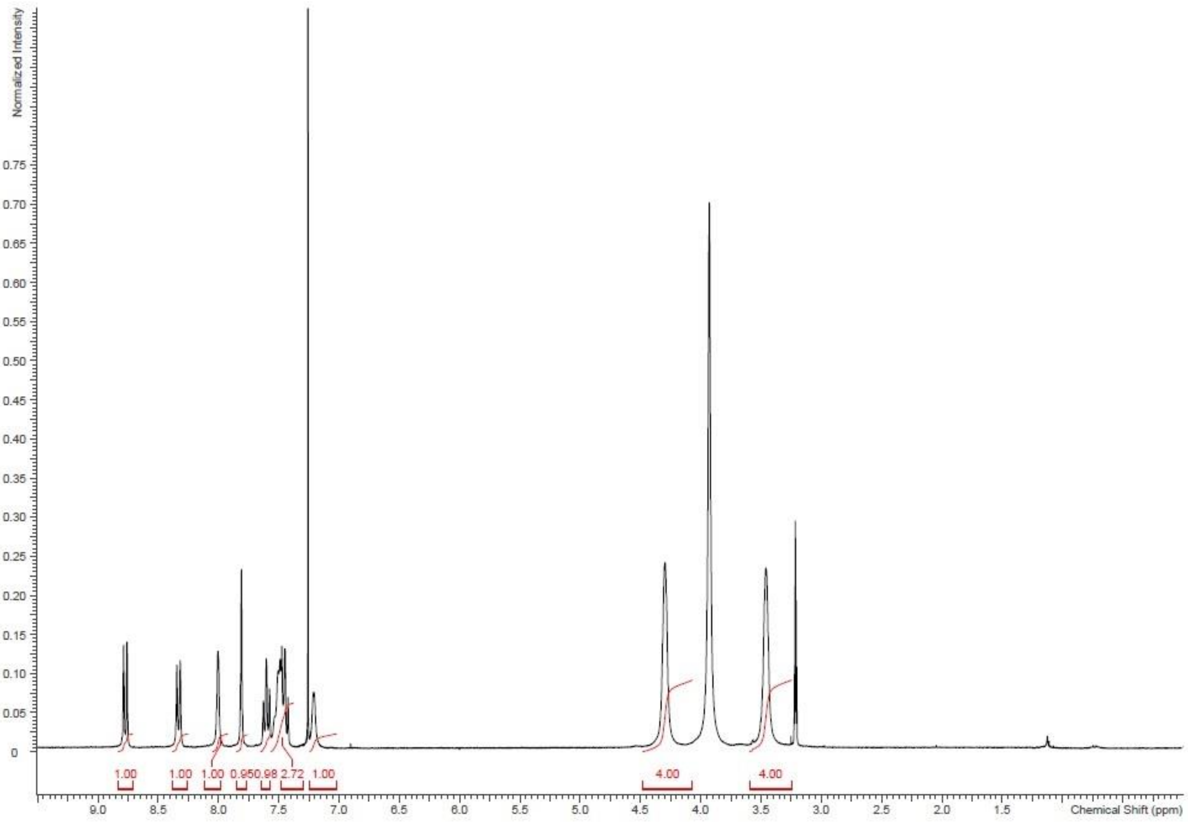

^{13}C NMR

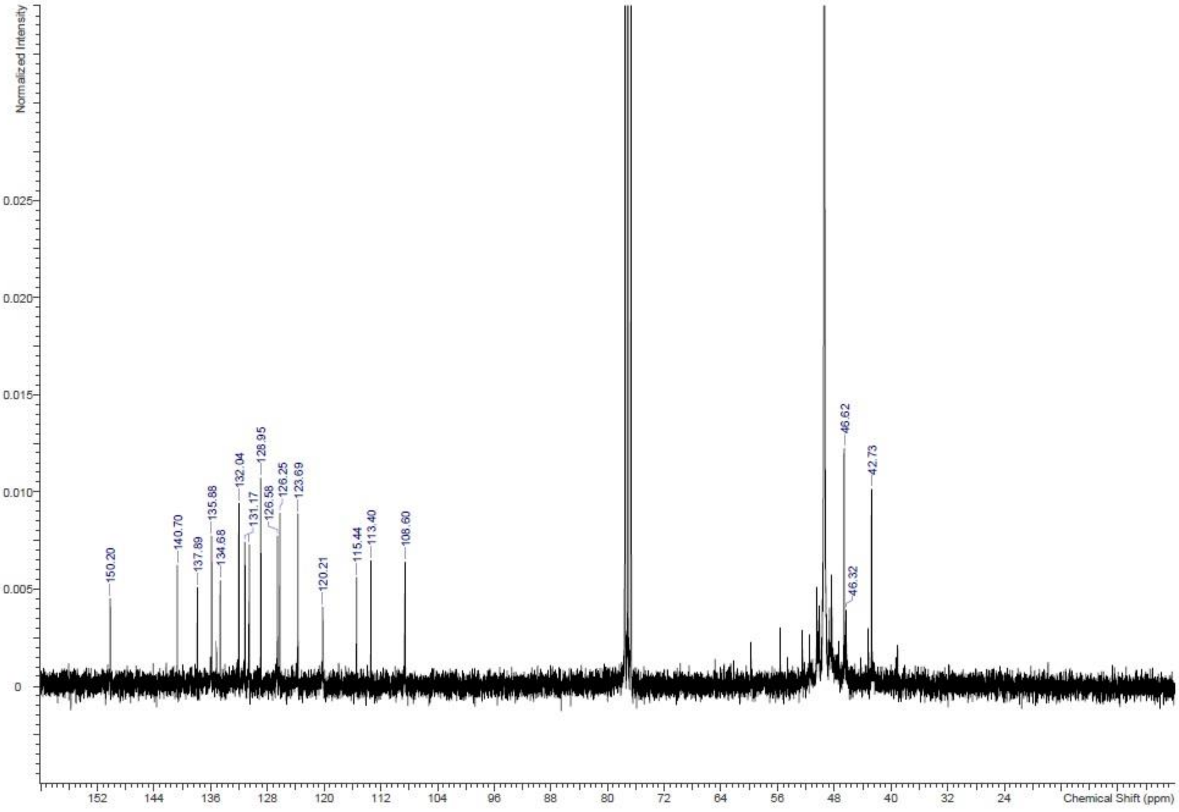

1-[(2,5-Difluorophenyl)sulfonyl]-4-(piperazin-1-yl)-1H-pyrrolo[3,2-c]quinoline dihydrochloride (**27**)

UPLC-MS

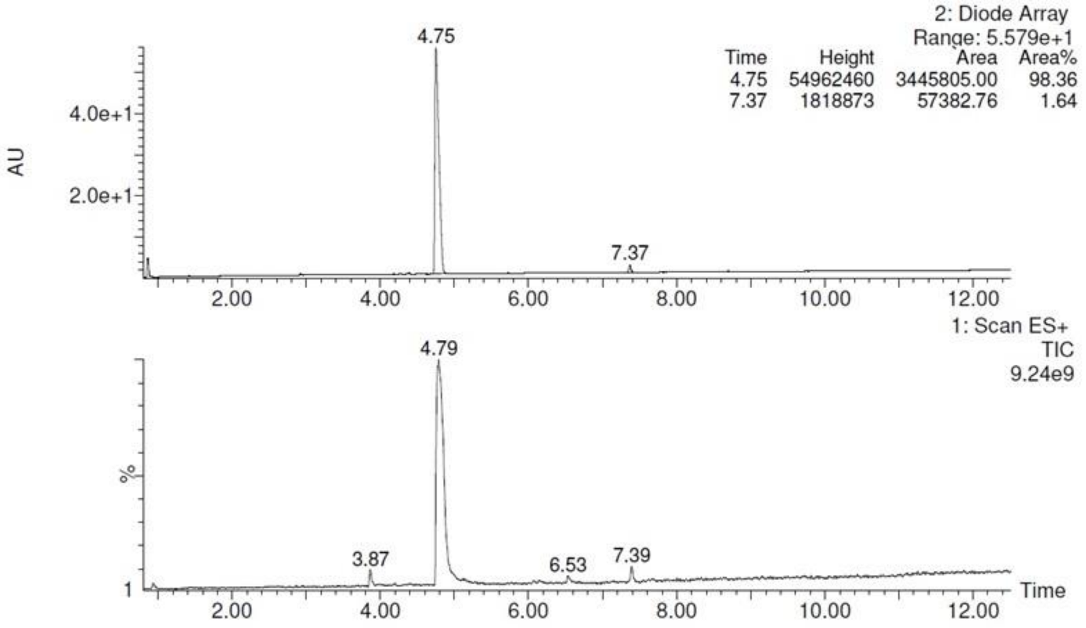

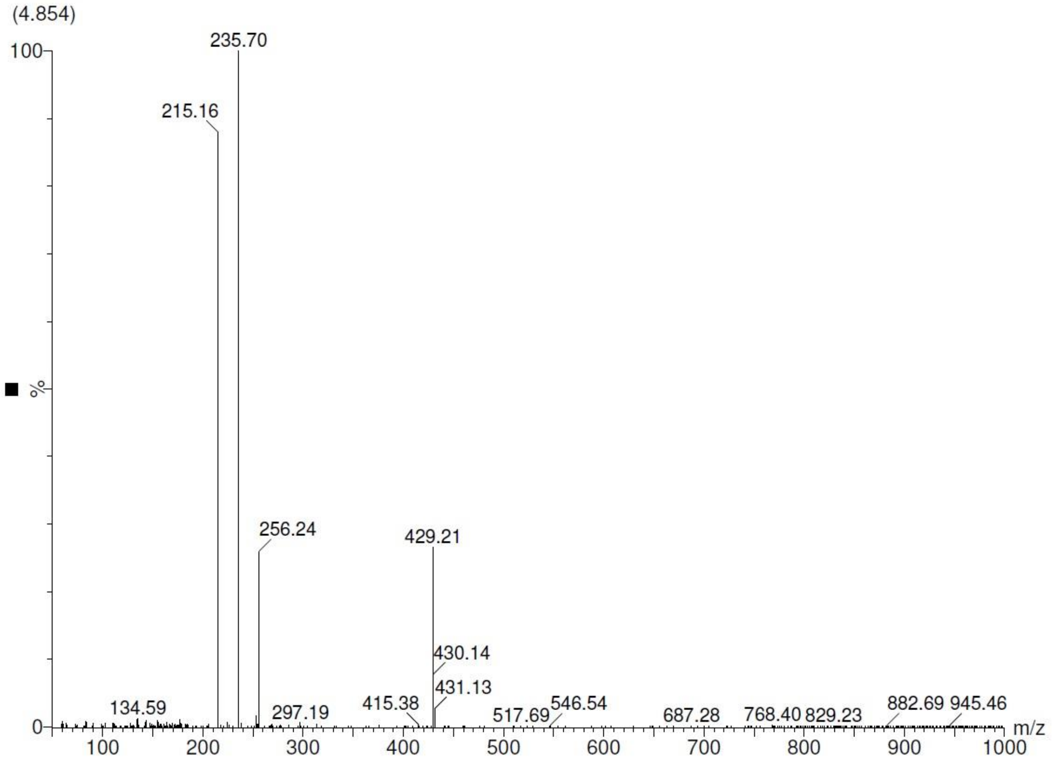

^{1}H NMR

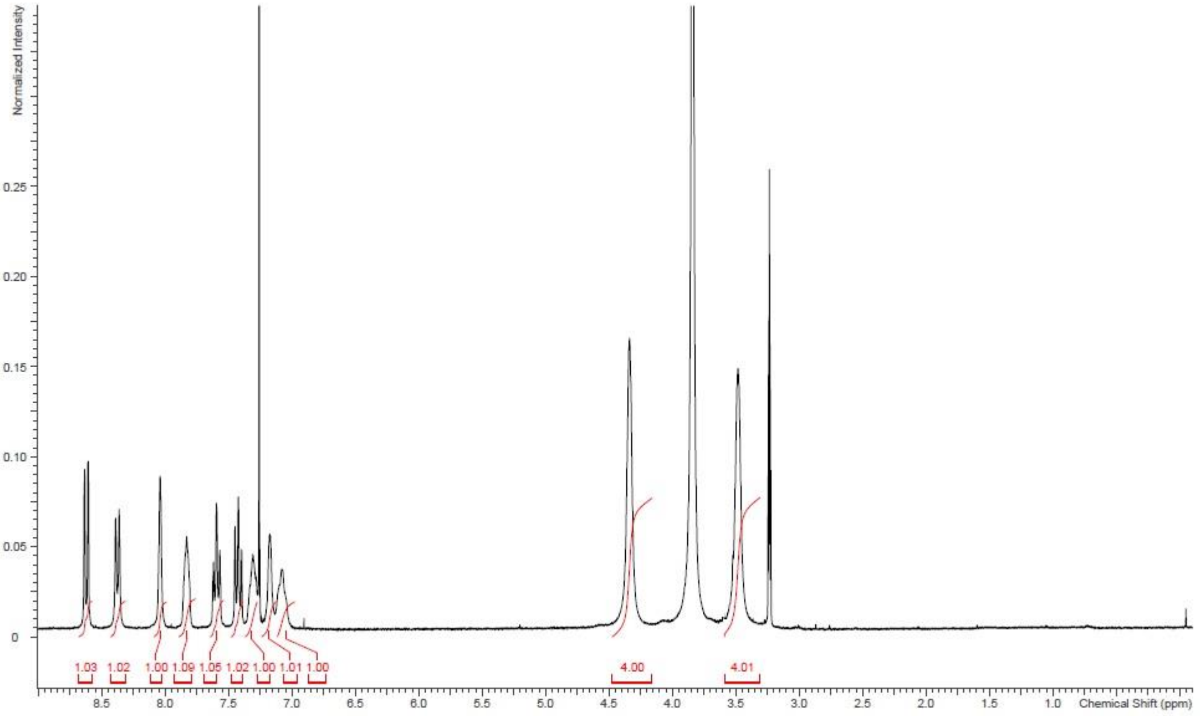

^{13}C NMR

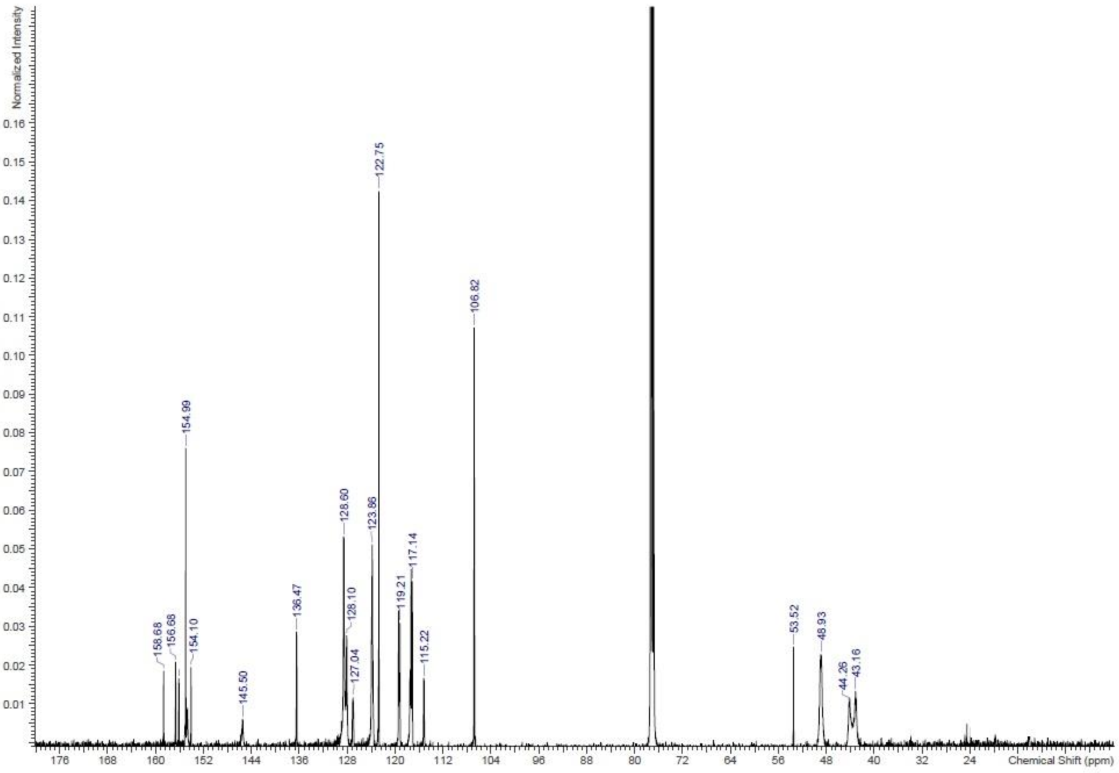

1-(Naphthalen-1-ylsulfonyl)-4-(piperazin-1-yl)-1H-pyrrolo[3,2-c]quinoline dihydrochloride (**28**)

UPLC-MS

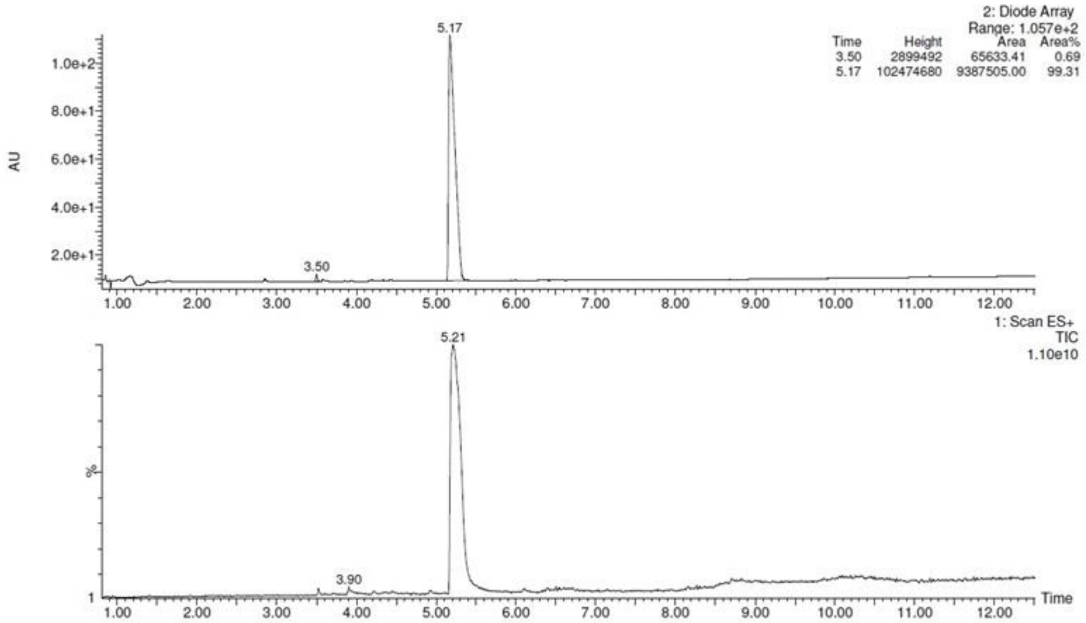

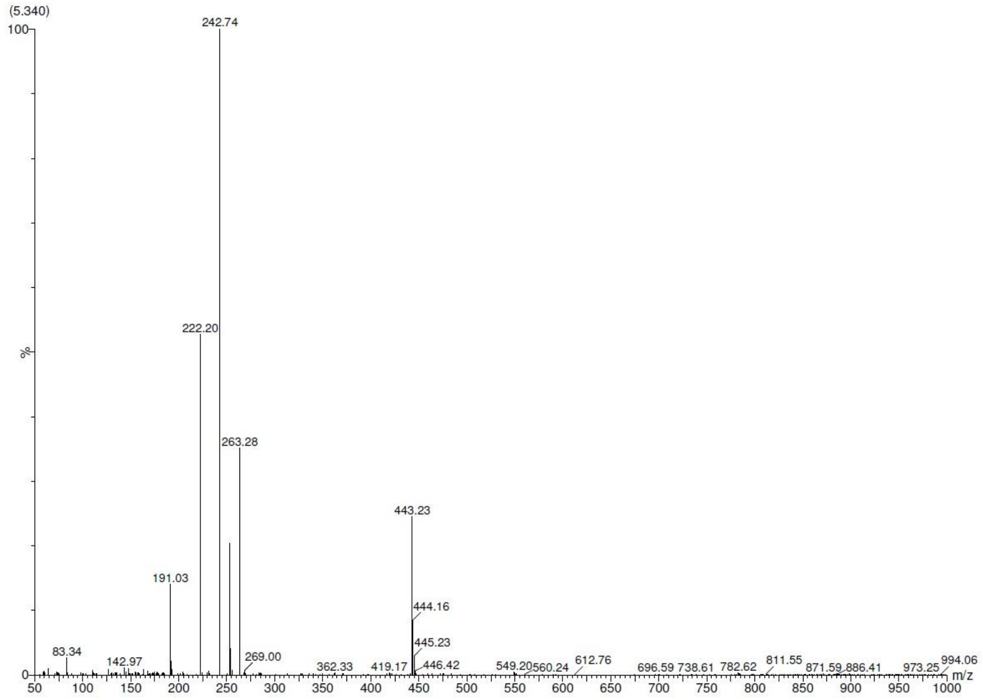

^{1}H NMR

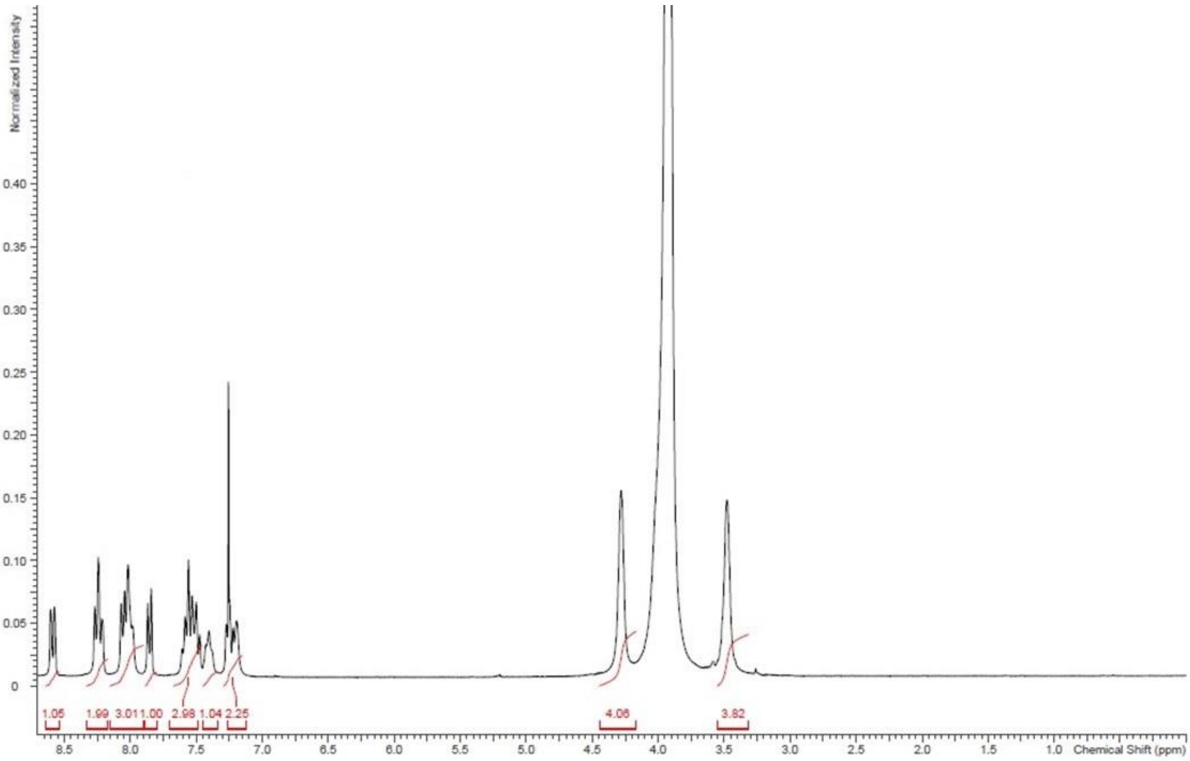

^{13}C NMR

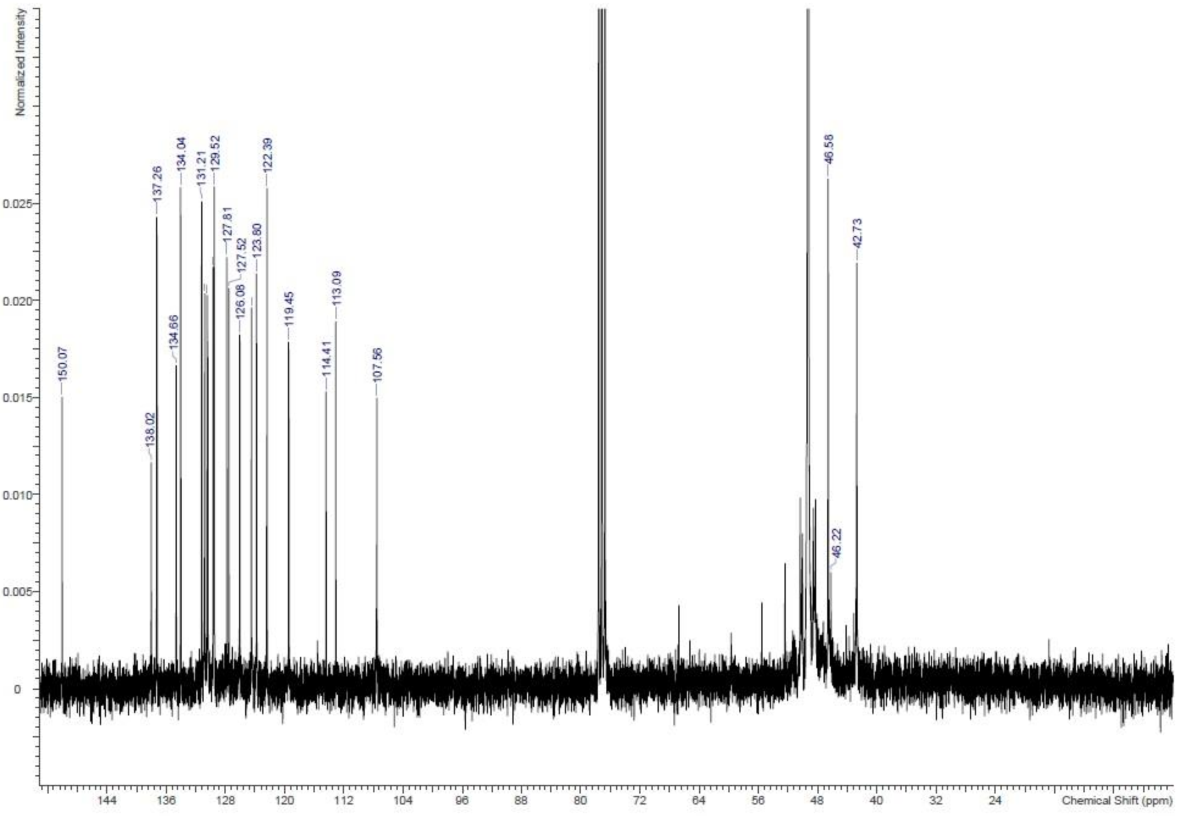

Synthesis and characterization of compoud II

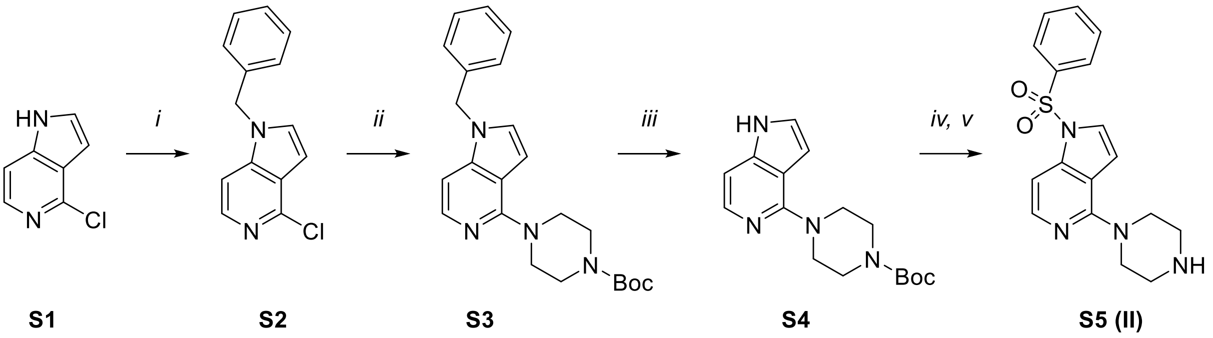

Scheme S1. Synthetic pathway leading to compound **II**. (*i*) Benzyl bromide, BTTP, CH_2Cl_2, 38 °C, 12 h; (*ii*) Boc-piperazine, TEA, MeCN, 180 °C MW, 5 h; (*iii*) K-O*t*Bu, DMSO, 90 °C; (*iv*) Benzenesulfonyl chloride, BTPP, CH_2Cl_2, 0°C → rt, 3 h; (*v*) 1M HCl/MeOH, rt, 5 h.

Hydrochloride salt: White solid, 70% yield, t_R = 3.37, $C_{17}H_{19}ClN_4O_2S$, MW 378.88. ^{1}H NMR (500 MHz, methanol-d_4) δ ppm 3.44–3.52 (m, 3H), 3.46–3.46 (m, 1H), 3.47–3.47 (m, 1H), 4.07 (bs, 4H), 7.25 (d, *J* = 4.0 Hz, 1H), 7.54–7.65 (m, 2H), 7.70–7.76 (m, 1H), 7.91 (d, *J* = 6.0 Hz, 2H), 8.05 (d, *J* = 4.0 Hz, 1H), 8.10 (dd, *J* = 8.6, *J* = 1.2 Hz, 2H), 8.10–8.10 (m, 1H). ^{13}C NMR (75 MHz, methanol-d_4) δ (ppm) 42.6, 45.6, 103.0, 108.2, 115.4, 127.3, 128.0, 129.9, 131.5, 135.4, 136.9, 141.7, 149.9. Monoisotopic Mass 342.12, $[M+H]^+$ 343.2

1-(Phenylsulfonyl)-4-(piperazin-1-yl)-1*H*-pyrrolo[3,2-*c*]pyridine (II)

UPLC-MS

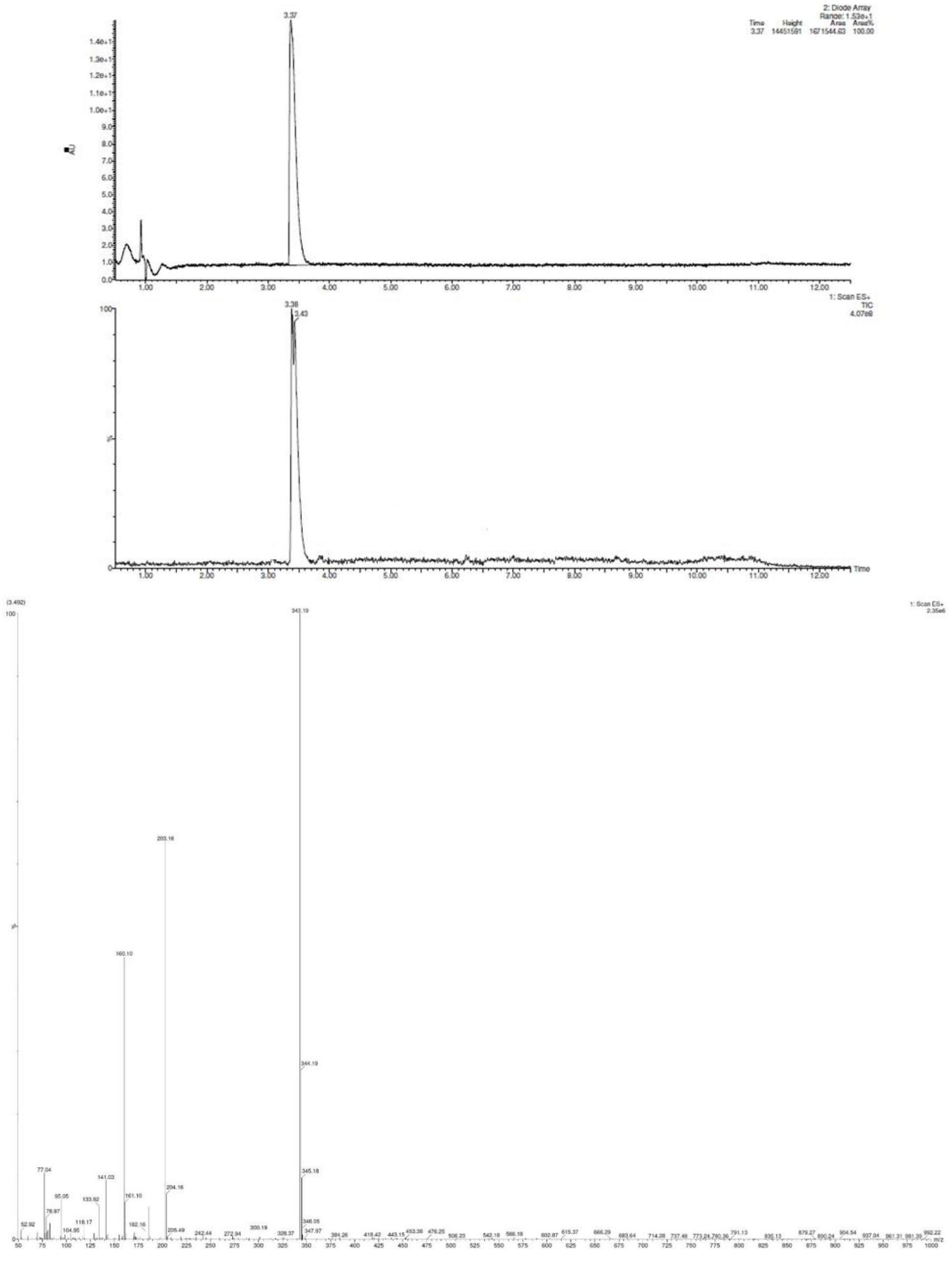

^{1}H NMR

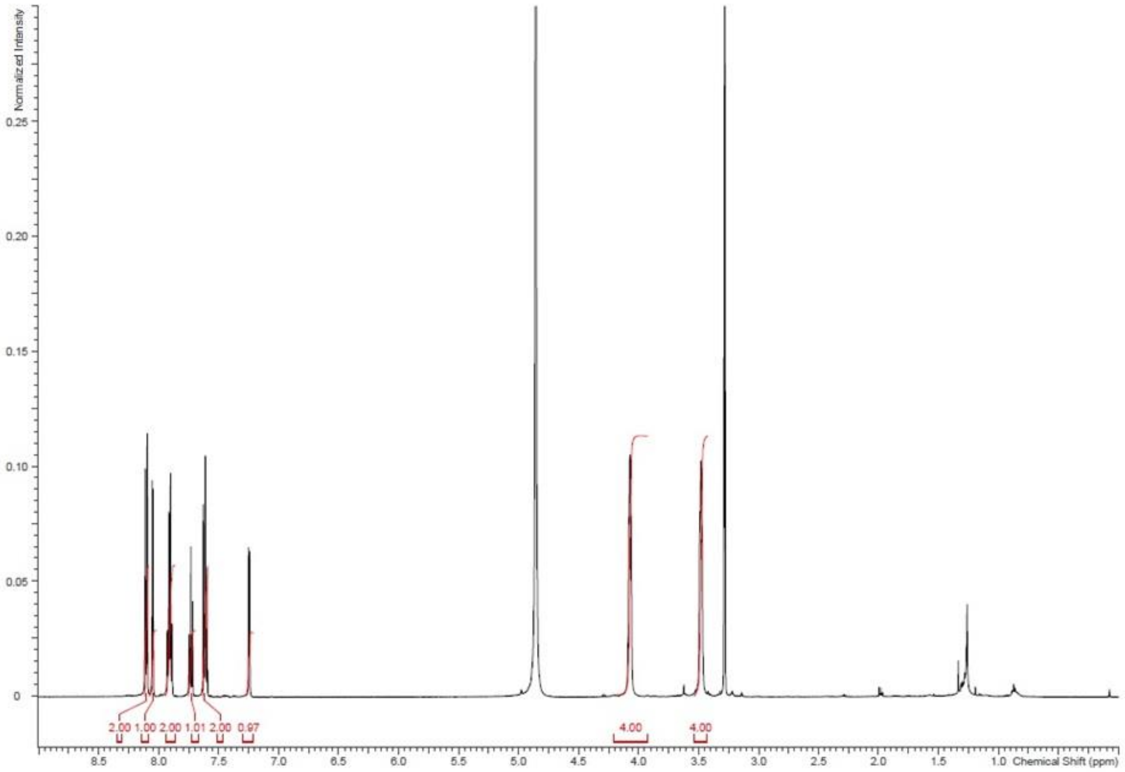

^{13}C NMR

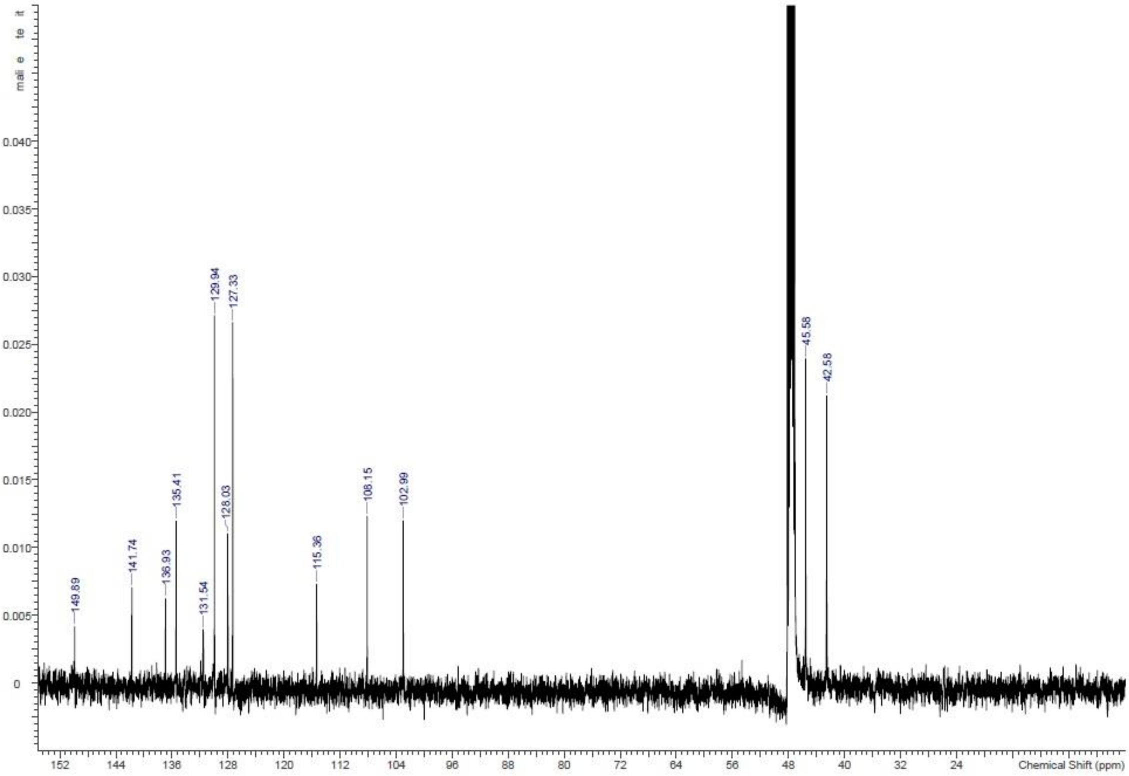

Table S1. Evaluation of the matching of derivatives with different modifications of the basic fragments to the 5-HT_3 and 5-HT_6 receptor binding sites.

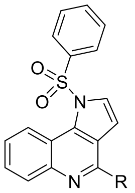

Compd	R	Fit score[a]	
		**5-HT3R**	**5-HT6R**
**6**	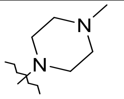	1	1
**7**	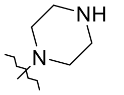	1	1
**8**	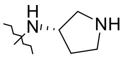	-1	1
**9**	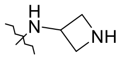	-1	1
**10**	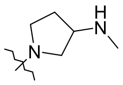	1	1
**11**	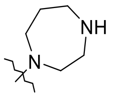	1	1
**S6**	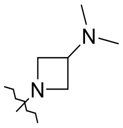	-1	-1
**S7**	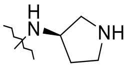	-1	1
**S8**	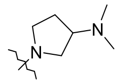	1	-1

[a] The parameter describes how well a given derivative adapts to the active site and reproduces key interactions observed in complexes of reference ligands with a given receptor (possible values: "1" - means very good fitting and creation of key interactions; "0" - means no docking of the given structure; "-1" - means wrong fitting to the binding pocket and/or no key interactions). The scores were obtained on the basis of visual inspection of ligand-receptor complexes obtained by molecular docking.

Table S2. The off-target selectivity of **FPPQ** for 87 primary molecular targets[a]

Assay	Species	Conc.	%inh	IC_{50} [µM]	K_i [µM]
Na^+/K^+ ATPase	pig	10 µM	1		
Acetylcholinesterase	hum	10 µM	11		
COX-1	human	10 µM	-6		
COX-2	human	10 µM	-13		
MAO-A	human	10 µM	12		
MAO-B	human	10 µM	21		
ACE	rabbit	10 µM	-9		
Cathepsin G	human	10 µM	-1		
PDE3	human	10 µM	10		
PDE4	human	10 µM	24		
Protein Set/Thr Kinase	rat	10 µM	-20		
Protein Tyr Kinase, Insulin receptor	human	10 µM	-6		
Protein Tyr Kinase, LCK	human	10 µM	6		
Adenosine A_1	human	10 µM	29		
Adenosine A_{2A}	human	10 µM	1		
Adrenergic α_{1A}	rat	10 µM	34		
Adrenergic α_{1B}	rat	10 µM	29		
Adrenergic α_{1D}	human	10 µM	24		
Adrenergic α_{2A}	human	10 µM[b]	98	0.22	0.11
		10 µM	97		
		1 µM	80		
		0.1 µM	32		
		10 nM	5		
Adrenergic α_{2B}	human	10 µM[b]	53	10.1	4.61
		100 µM	108		
		10 µM	43		
		1 µM	-4		
		0.1 µM	1		
Adrenergic β_1	human	10 µM[b]	102	0.30	0.17
		10 µM[b]	100		
		1 µM	75		
		0.1 µM	27		
		10 nM	-3		
Adrenergic β_2	human	10 µM[b]	99	0.12	0.085
		10 µM	103		
		1 µM	93		
		0.1 µM	43		
		10 nM	9		
Androgen (Testosterone)	human	10 µM	13		
Angiotensin AT_1	human	10 µM	-4		
Bradykinin B_2	human	10 µM	0		
Ca Channel L-Type, Benzothiazepine	rat	10 µM[b]	92	1.22	1.08
		10 µM	83		
		1 µM	46		
		0.1 µM	15		
		10 nM	-3		

**Assay**	**Species**	**Conc.**	**%inh**	**IC_{50} [µM]**	**K_i [µM]**
		10 µM[b]	78		
		10 µM	89		
Ca Channel L-Type, Dihydropyridine	rat	1 µM	60	0.78	0.50
		0.1 µM	3		
		10 nM	-4		
		10 µM[b]	85		
		10 µM	78		
Ca Channel L-Type, Phenylalkylamine	rat	1 µM	48	1.36	1.32
		0.1 µM	9		
		10 nM	-2		
Ca Channel N-Type	human	10 µM	11		
Cannabinoid CB_1	human	10 µM	35		
Cannabinoid CB_2	human	10 µM	-21		
Chemokine CCR1	human	10 µM	-6		
Chemokine CCR2 (IL-$8R_B$)	human	10 µM	21		
Dopamine D_1	human	10 µM	27		
Dopamine D_{2L}	human	10 µM	46		
Dopamine D_{2S}	human	10 µM	46		
		10 µM[b]	90		
		10 µM	89		
Dopamine D_3	human	1 µM	63	0.63	0.21
		0.1 µM	14		
		10 nM	-2		
Endothelin ET_A	human	10 µM	3		
Estrogen ERα	human	10 µM	22		
$GABA_A$ Chloride channel, TBOB	rat	10 µM	8		
$GABA_A$ Flunitrazepam, Central	rat	10 µM	8		
$GABA_A$ Ro-15-1788, Hippocampus	rat	10 µM	-5		
$GABA_{B1A}$	human	10 µM	13		
Glucocorticoid	human	10 µM	8		
Glutamate AMPA	rat	10 µM	8		
Glutamate Kainate	rat	10 µM	-2		
Glutamate Metabotropic, $mGlu_5$	human	10 µM	16		
Glutamate NMDA, Agonism	rat	10 µM	-2		
Glutamate NMDA, Glycine	rat	10 µM	-1		
Glutamate NMDA, Phencyclidine	rat	10 µM	4		
Glutamate NMDA, Polyamine	rat	10 µM	2		
Glycine, Strychnine-sensitive	rat	10 µM	-1		
		10 µM[b]	98		
		10 µM	97		
Histamine H_1	human	1 µM	67	0.46	0.22
		0.1 µM	19		
		10 nM	4		
Histamine H_2	human	10 µM	45		
Histamine H_3	human	10 µM	26		
Leukotriene, Cysteinyl $CysLT_1$	human	10 µM	10		
Melanocortin MC_1	human	10 µM	10		
Melanocortin MC_4	human	10 µM	27		

Assay	Species	Conc.	%inh	IC_{50} [µM]	K_i [µM]
		10 µM[b]	57		
		100 µM	98		
Muscarinic M_1	human	10 µM	54	7.89	1.94
		1 µM	13		
		0.1 µM	3		
Muscarinic M_2	human	10 µM	5		
Muscarinic M_3	human	10 µM	43		
Muscarinic M_4	human	10 µM	8		
Neuropeptide Y Y_1	human	10 µM	37		
Nicotinic Acetylcholine	human	10 µM	-6		
Nicotinic Acetylcholine α1, Bungarotoxin	human	10 µM	11		
Opiate σ_1 (OP1, DOP)	human	10 µM	7		
		10 µM[b]	64		
		100 µM	99		
Opiate κ (OP2, KOP)	human	10 µM	55	8.18	3.27
		1 µM	8		
		0.1 µM	5		
		10 µM[b]	61		
		100 µM	97		
Opiate µ (OP3, MOP)	human	10 µM	59	7.08	2.88
		1 µM	11		
		0.1 µM	3		
Platelet Activating Factor (PAF)	human	10 µM	14		
Potassium Channel [K_{ATP}]	human	10 µM	1		
		10 µM[b]	101		
		10 µM	107		
Potassium Channel [hERG]	human	1 µM	32	1.15	0.94
		0.1 µM	9		
		10 nM	9		
PPARγ	human	10 µM	28		
Progesterone PR-B	human	10 µM	21		
		10 µM[b]	91		
		10 µM	87		
Serotonin 5-HT_{1A}	human	1 µM	53	0.86	0.49
		0.1 µM	16		
		10 nM	-2		
		10 µM[b]	92		
		10 µM	90		
Serotonin 5-HT_{1B}	human	1 µM	47	1.17	0.89
		0.1 µM	4		
		10 nM	5		
Serotonin 5-HT_{2A}	human	10 µM	50		
		10 µM[b]	97		
		10 µM	96		
Serotonin 5-HT_{2B}	human	1 µM	71	0.26	0.17
		0.1 µM	31		
		10 nM	13		

Assay	Species	Conc.	%inh	IC_{50} [µM]	K_i [µM]
Serotonin $5\text{-}HT_{2C}$	human	10 µM[b]	93	1.58	0.83
		10 µM	94		
		1 µM	32		
		0.1 µM	-6		
		10 nM[b]	-5		
Serotonin $5\text{-}HT_3$	human	10 µM[b]	101	0.00412	0.00093
		1 µM	100		
		0.1 µM	99		
		10 nM	76		
		1 nM	14		
Serotonin $5\text{-}HT_4$	guinea pig	10 µM	30		
Serotonin $5\text{-}HT_{5A}$	human	10 µM[b]	61	5.58	2.87
		100 µM	98		
		10 µM	64		
		1 µM	15		
		0.1 µM	1		
Serotonin $5\text{-}HT_7$	human	10 µM[b]	67	5.01	2.87
		100 µM	93		
		10 µM	65		
		1 µM	21		
		0.1 µM	-1		
Sigma σ_1	human	10 µM[b]	51	8.33	3.50
		100 µM	89		
		10 µM	51		
		1 µM	18		
		0.1 µM	7		
Sodium Channel, Site 2	rat	10 µM[b]	93	0.78	0.71
		10 µM	94		
		1 µM	47		
		0.1 µM	23		
		10 nM[b]	9		
Tachykinin NK_1	human	10 µM[b]	58	13.70	9.92
		100 µM	92		
		10 µM	40		
		1 µM	10		
		0.1 µM	0		
Adenosine Transporter	guinea pig	10 µM	12		
Dopamine Transporter (DAT)	human	10 µM[b]	98	1.37	1.09
		10 µM	101		
		1 µM	31		
		0.1 µM	6		
		10 nM	2		
GABA Transporter	rat	10 µM	3		
Norepinephrine Transporter (NET)	human	10 µM[b]	96	1.25	1.24
		10 µM	94		
		1 µM	42		
		0.1 µM	8		
		10 nM	1		
Serotonin Transporter (SERT)	human	10 µM	18		
Vasopressin V_{1A}	human	10 µM	-18		

[a] Performed at Eurofins. [b] Compound tested in additional batch.

Table S3. Mutagenicity risk assessment for **FPPQ**

**Compd**	**Conc. [μg/ml]**	**Number of revertants**[a]			
		**TA-100**	**TA-1535**	**TA-98**	**TA-1537**
**17** (**FPPQ**)	16	6 ± 1	10 ± 4	10 ± 3	8 ± 2
	8	8 ± 3	9 ± 1	8 ± 3	7 ± 3
	4	8 ± 3	8 ± 4	10 ± 2	5 ± 1
	0.4	5 ± 2	10 ± 1	8 ± 4	5 ± 2
	0.2	5 ± 2	6 ± 3	4 ± 2	3 ± 3

[a]Mean ±SEM of number of revertants/plate for each tester strain.
